# Oxetane Synthesis via Alcohol C–H Functionalization

**DOI:** 10.1021/jacs.3c04891

**Published:** 2023-07-18

**Authors:** Subhasis Paul, Dario Filippini, Filippo Ficarra, Heorhii Melnychenko, Christopher Janot, Mattia Silvi

**Affiliations:** † The GSK Carbon Neutral Laboratories for Sustainable Chemistry, University of Nottingham, Jubilee Campus, Nottingham NG7 2TU, United Kingdom; ‡ School of Chemistry, University of Nottingham, University Park, Nottingham NG7 2RD, United Kingdom; § Chemical Development, Pharmaceutical Technology and Development, Operations, AstraZeneca, Macclesfield, SK10 2NA, United Kingdom

## Abstract

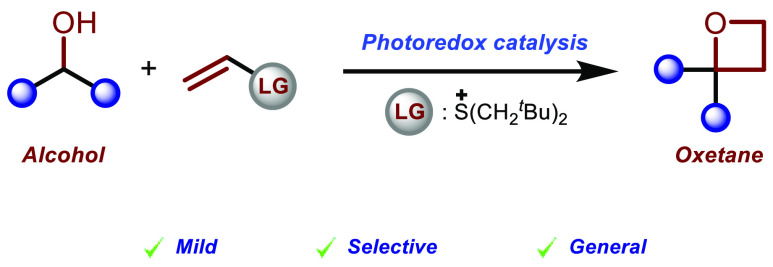

Oxetanes are strained heterocycles with unique properties that have triggered significant advances in medicinal chemistry. However, their synthesis still presents significant challenges that limit the use of this class of compounds in practical applications. In this Letter, we present a methodology that introduces a new synthetic disconnection to access oxetanes from native alcohol substrates. The generality of the approach is demonstrated by the application in late-stage functionalization chemistry, which is further exploited to develop a single-step synthesis of a known bioactive synthetic steroid derivative that previously required at least four synthetic steps from available precursors.

Oxetanes (Scheme 1a) have received considerable interest in recent years as a result of their unique chemical and physical properties.^1^ These strained^2^ heterocycles are known to possess structural rigidity, low lipophilicity, high H-bonding acceptor ability,^3^ and have been observed to possess enhanced metabolic stability compared with other related oxygen heterocycles.^4^ Thus, oxetanes are of increasing importance in drug design^1,3^ and can be found in numerous relevant bioactive molecules.^5^ Despite this privileged role, the use of oxetanes in agrochemicals and pharmaceuticals is often hampered by their synthesis, which poses significant challenges (Scheme 1b).^1^ A traditional strategy to access oxetanes is the Paternò–Büchi [2 + 2] cycloaddition (Scheme 1b, left).^6^ Despite being an established process in photochemistry, this process is often complicated by both reactivity and selectivity factors, which are known to be substrate-dependent.^7^ Furthermore, the typical requirement for UV light irradiation can be problematic and may lead to significant side product formation. While the use of lower-energy visible light irradiation has been recently reported in Paternò–Büchi reactions,^8^ only specific classes of substrates have been demonstrated to react under these conditions. Because of the scope limitations mentioned above, the most commonly used methodology to construct oxetanes is the intramolecular Williamson synthesis (Scheme 1b, center).^9,1^ However, the synthesis of starting materials bearing an alcohol and a leaving group in a 1,3-relationship often requires cumbersome multistep synthetic sequences. Although strategies to generate such species *in situ* from more common precursors have been explored, e.g., from epoxides (Scheme 1b, right) or from structurally simple ketones,^10^ the scope of such methodologies is generally limited, and in most cases, a tedious sequence of synthetic steps is still required to obtain the target oxetanes from available precursors. These drawbacks considerably limit the structural diversity of the oxetane products accessible.

**Scheme 1:**
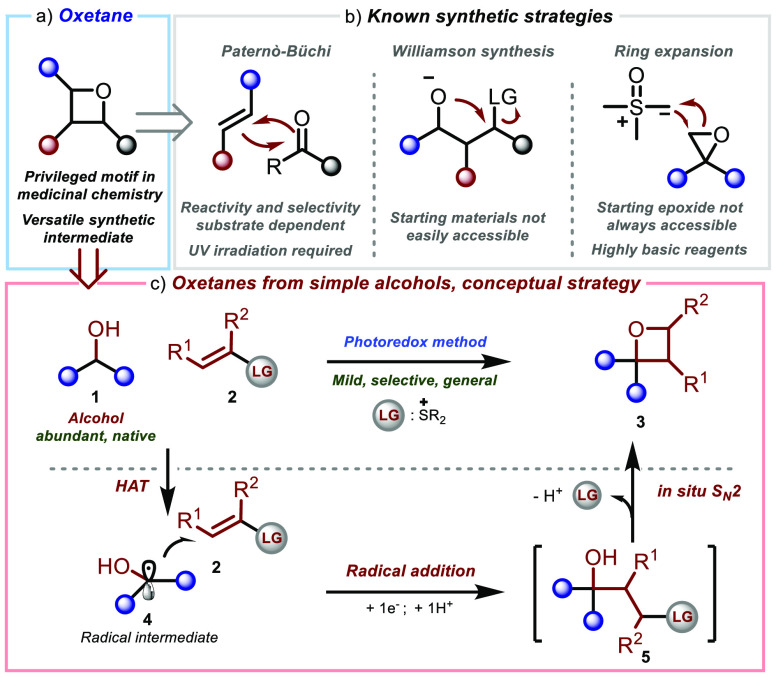
(a) Oxetane Functionality and Applications, (b) Examples of Current Oxetane Synthesis Methods and Limitations, and (c) Planned Strategy: Synthesis of Oxetanes from Alcohols and Conceptual Plan

In contrast to the established methodologies listed above, which rely on carbonyl, epoxides, or ad hoc designed alcohol starting materials, in this report we describe a methodology that introduces a novel synthetic disconnection to access oxetanes from inactivated alcohols via selective C–H functionalization (Scheme 1c, top). Such a process is expected to significantly extend the range of accessible oxetane structures, thereby allowing direct access to these strained heterocycles from a native alcohol functionality that is ubiquitous in organic molecules.^11^

In our conceptual plan (Scheme 1c, bottom), we envisioned the selective radical generation in α-position of an alcohol substrate **1** via H atom transfer (HAT)^12−14^ to give **4**, followed by addition to a suitable alkene **2** to afford intermediate **5**. If it possessed sufficient leaving group (LG) ability, then the LG functionality within intermediate **5** would promote an *in situ* cyclization to give oxetane **3**. In such a strategy, the functional group LG within radical trap **2** would play the critical, dual role of modulating the alkene electronics to ensure polarity matching in the key addition of nucleophilic radical **4**(15) while at the same time presenting an excellent leaving group ability to promote a challenging 4-*exo*-*tet* S_N_2 cyclization.^16^ Our group^17^ has recently demonstrated that vinyl sulfonium ions^18^ readily participate in radical conjugate addition reactions, thereby giving highly reactive adducts that are prone to undergo intermolecular S_N_2 reactions with nucleophiles.^17,19^ Given its excellent leaving group ability,^20^ we envisioned that the cationic sulfonium functionality would provide alkene **2** with the unique combination of properties required to successfully realize the plan in Scheme 1c.

Through exploitation of the ability of quinuclidine to selectively abstract hydrogens in alcohol substrates^12,13^ under photoredox^21^ conditions, we commenced our investigation by irradiating an acetonitrile solution of 2 equiv of cyclohexanol **1a** and 1 equiv of diphenyl vinyl sulfonium triflate **2a**(22,18) in the presence of catalytic iridium complex {Ir[dF(CF_3_)ppy]_2_(dtbpy)}PF_6_ (1 mol %), tetrabutyl ammonium dihydrogen phosphate (25 mol %),^12a^ and quinuclidine (10 mol %). After irradiation, KO^*t*^Bu was added to the vessel, and the reaction mixture was stirred at 60 °C. Analysis of the reaction mixture revealed the presence of traces of the desired oxetane **3a** with a low mass balance due to various unidentified decomposition pathways (Table 1, entry 1). In consonance with our previous studies,^17^ neopentyl-substituted structure **2b** was found to be unique in promoting the radical process (see the Supporting Information for full optimization details and comparison with other vinyl sulfonium systems), thereby giving the desired oxetane **3a** in excellent 97% yield (entry 2). Although a milder base (K_3_PO_4_) could be used in place of KO^*t*^Bu to promote cyclization, a higher temperature (80 °C) and extended cyclization time were required, which led to the desired product **3a** in reduced 72% yield (entry 3). Thus, KO^*t*^Bu was selected as the optimal base. In order to use this methodology to functionalize valuable complex alcohols, the stoichiometry of the reaction was adjusted to use compound **1a** as the limiting reagent. Under these conditions, product **3a** was obtained in quantitative yield using a slight excess (1.5 equiv) of vinyl sulfonium ion **2b** (entry 4). Finally, replacement of the iridium photocatalyst with 5 mol % of the more affordable organic photocatalyst 4CzIPN^23^ had no impact on the efficiency of the process and afforded the final spiro-oxetane **3a** in quantitative yield (entry 5, 74% yield of isolated material because of the volatility of **3a**). As expected for a radical process, by omitting light and performing the reaction in the presence of a radical inhibitor, we did not observe the formation of product **3a** (entries 6 and 7).

**Table 1:**
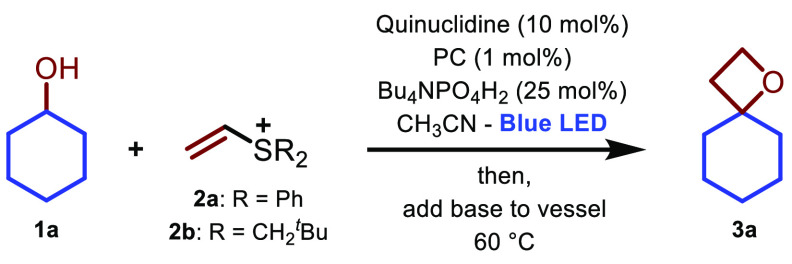
Optimization Studies

entry^a^	alkene	photocatalyst (PC)	base	**3a** (%)^b^
1	**2a**	Ir[dF(CF_3_)(ppy)_2_dtbpy]^+^	KO*^t^*Bu	traces
2	**2b**	Ir[dF(CF_3_)(ppy)_2_dtbpy]^+^	KO*^t^*Bu	97
3^c^	**2b**	Ir[dF(CF_3_)(ppy)_2_dtbpy]^+^	K_3_PO_4_	72
4^d^	**2b**	Ir[dF(CF_3_)(ppy)_2_dtbpy]^+^	KO*^t^*Bu	99
5^d^^,^^e^	**2b**	4CzIPN	KO*^t^*Bu	99 (74)
6^d^^–^f	**2b**	4CzIPN	KO*^t^*Bu	0
7^d^^,^^e^^,^^g^	**2b**	4CzIPN	KO*^t^*Bu	0

a Unless otherwise noted, reactions were carried out irradiating 2 equiv of **1a** and 1 equiv of **2** using the conditions as in header scheme and 2 equiv of base at a 0.1 mmol scale; see the Supporting Information for full optimization details. A PF_6_^–^ counterion is intended for the iridium photocatalyst, and a TfO^–^ is intended for sulfonium salts.

b NMR yield using dibromomethane as an internal standard. In parentheses is the isolated yield of a 0.2 mmol scale reaction.

c After base addition, the reaction was heated at 80 °C for 41 h.

d Stoichiometry: 1 equiv of **1a**, 1.5 equiv of **2b**.

e 5 mol % of PC was used.

f Reaction performed with TEMPO (1 equiv).

g Performed in the dark.

With the optimized conditions in hand, we explored the generality of the process by exposing different alcohol substrates to our reaction conditions. A variety of six-membered ring heterocycles, including ethers or acetals, which may present issues of HAT site selectivity,^24,14^ readily undergo the desired reaction to afford spirocyclic products **3b** and **3c** in high yields. Sulfone functionalities are tolerated, as shown by the good yield obtained for the oxetane product **3d**. For this entry, overstoichiometric zinc chloride^12b^ was used in place of tetrabutyl ammonium hydrogen phosphate, and reaction conditions were revised to ensure high starting material conversion (see the Supporting Information for details). Piperidine-derived oxetane **3e** can also be obtained in high yields under the same conditions, with the Bz protecting group chosen over a Boc protecting group to ensure full HAT site selectivity.^12b^ 1,4-Substituted cyclohexanol carrying an ester functionality successfully undergoes the desired reaction, thereby leading to the final product **3f** in moderate yield and diastereoselectivity. For this entry, the use of potassium phosphate as a base was preferred over the corresponding *tert*-butoxide to minimize undesired ester hydrolysis (see the Supporting Information for details). Spirocyclic oxetanes with different ring sizes and strains can be successfully accessed, with both macrocycle **3g** and highly strained spirocycle **3h** obtained in high yields. Attracted by the known synthetic interest in oxetane polyspirocyclic structures,^1c^ we successfully applied our methodology to construct compounds **3i**–**3k**, which feature an oxetane ring connected with variously strained *N*-heterocycles via two contiguous spirocenters. The moderate to high yields obtained further demonstrate the versatility of this methodology to access products presenting extreme strain energy. The construction of spirocyclic oxetanes is also feasible within different bicyclic and polycyclic architectures, with norborneol, adamantanol, and aza-byciclooctane heterocyclic alcohol substrates leading to the desired products **3l**, **3m**, and **3n** in respective 71, 50, and 78% yield. Remarkably, oxetanes **3l** and **3n** are obtained with full diastereoselectivity. A variety of linear secondary alcohols undergo the desired reactivity to afford products **3o**–**3q**, which bear benzylic or ether functionalities featuring weak hydridic C–H bonds that could potentially undergo competitive side-reactivity. Versatile nitrile functional groups can be incorporated within the product structures, with oxetane product **3r** obtained in a synthetically useful 57% yield. Primary alcohols are also suitable substrates, which provide access to a variety of oxetanes containing ethers, protected alcohols, and linear chains (**3s**–**3v**). Lower yields (42–56%) are obtained in these products when compared with the corresponding oxetanes derived from secondary alcohol substrates, with minor amounts of vinyl alcohol byproducts generated via a competing E2 elimination pathway occurring in the corresponding sulfonium intermediates. This observation suggests that the reduced yields observed are ascribable to a slower cyclization due to a reduced Thorpe–Ingold effect^25^ rather than to a lower efficiency of the radical addition step. As a limitation of this method, benzylic, allylic, and propargylic alcohols do not lead to the desired products; see the Supporting Information for more details.

We then investigated the introduction of further substitution in the oxetane products by subjecting the corresponding propenyl sulfonium to the reaction conditions, which resulted in obtaining β-methyl-substituted oxetane **3w** in moderate yield. For this substrate, an enhanced loading of the more robust ZnCl_2_-promoted catalytic system is required to ensure starting material conversion. The introduction of alkyl or aryl substituents in the α-position of the oxetane ring is also possible, and desired products **3x** and **3y** can be obtained from the corresponding alkenyl sulfonium ions in moderate yields. For these entries, undesired E2 elimination and Grob fragmentation^26^ are important competing pathways, which can be minimized by operating an *in situ* solvent exchange to hexamethylphosphoramide (HMPA) or 1,3-Dimethyl-3,4,5,6-tetrahydro-2(1H)-pyrimidinone (DMPU) after irradiation and using MeMgBr as base to promote cyclization^27^ (see the Supporting Information for more details).

Finally, the oxetane synthesis described in this report can be successfully applied in late-stage functionalization and chemical modification of complex alcohols. For example, pregnenolone, which bears an alkene and a ketone functionality, undergoes the desired process to give oxetane **3z** in 44% yield and full diastereoselectivity. Galactose-derived oxetane **3aa** can also be obtained as a single diastereoisomer from the corresponding commercially available primary alcohol after a simple chromatographic purification of the reaction mixture.

Oxetane-containing steroid **3ab** (Scheme 2a, bottom) is known for its interesting biological activity in reversing the effects of desoxycorticosterone acetate on urinary sodium and potassium and inhibiting the stimulatory effects of estrogens on the growth of uterus.^28b^ However, the synthesis of this compound, as well as that of other related oxetane-containing steroid analogues, requires a multistep synthetic approach and the use of highly basic reagents. These drawbacks have considerably hampered research on oxetane steroid derivatives in drug design. The most recent synthesis of **3ab** involves four chemical steps from androstenedione:^28a^ respectively, the protection of the carbonyl cyclohexenone core as an enamine (more established protecting groups proved to be unsuccessful), a sulfonium ylide-mediated Corey–Chaykovsky epoxidation,^29^ a sulfoxonium ylide epoxide ring expansion to oxetane,^10^ and finally deprotection to afford desired product **3ab** (Scheme 2a, bottom). In contrast to this lengthy synthetic sequence, by simply submitting native testosterone to our reaction conditions, the desired oxetane **3ab** can be obtained in a single synthetic step with full diastereoselectivity (Scheme 2b, bottom), thereby demonstrating the potential of the synthetic methodology described in this report in medicinal chemistry applications.

**Scheme 2:**
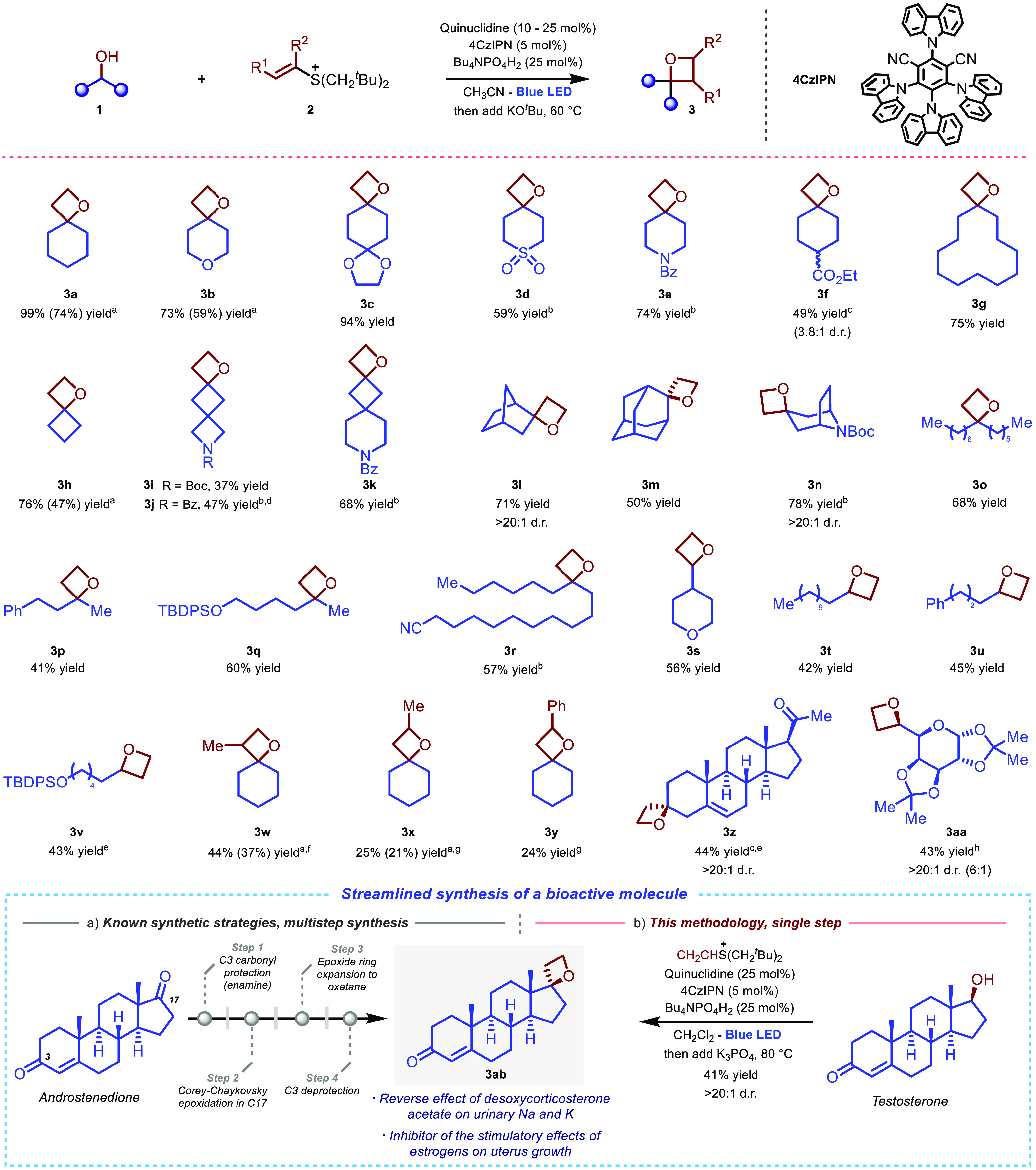
Reaction Scope and Example of Application of This Methodology in the Synthesis of a Known Bioactive Molecule Volatile material; value reported as NMR yield against CH_2_Br_2_ or phenanthrene as internal standards; the yield of isolated product after chromatographic purification is in parentheses. Reaction carried out using 2.2 equiv of ZnCl_2_, 1.5 equiv of K_3_PO_4_, 1 mol % {Ir[dF(CF_3_)ppy]_2_(dtbpy)}PF_6_, and 30 mol % of quinuclidine; see the Supporting Information for full experimental details. K_3_PO_4_ was used as base with heating at 80 °C. Reaction performed at a 0.1 mmol scale. Reaction performed in dichloromethane. Reaction carried out using 2.2 equiv of ZnCl_2_, 1.5 equiv of K_3_PO_4_, 3 mol % {Ir[dF(CF_3_)ppy]_2_(dtbpy)}PF_6_, and 75 mol % of quinuclidine; see the Supporting Information for full experimental details. After irradiation, *in situ* solvent exchange to HMPA or DMPU, and MeMgBr was used as base. dr value refers to isolated product after chromatographic purification; the dr observed in the reaction crude mixture is in parentheses. Unless otherwise noted, reactions are carried out at the 0.2 mmol scale, yields refer to isolated material after chromatographic purification, and dr is determined via ^1^H NMR analysis of the crude reaction mixture. TfO^–^ or BF_4_^–^counterions are intended for vinyl sulfoniums. See specific entry details in the Supporting Information.

As depicted in Scheme 3, luminescence quenching studies and electrochemical studies (see the Supporting Information for more details) suggest that this process proceeds through reductive quenching of photoexcited 4CzIPN* to mediate the formation of a quinuclidinium radical cation.^12^ This species undergoes HAT with activated H-bonded alcohol **1**,^12a^ which leads to nucleophilic radical **4** that quickly adds to **2** to give radical cation **6**.^17^ Single-electron reduction of this intermediate closes the photoredox cycle and generates a transient ylide that quickly undergoes protonation to give **5**. KO*^t^*Bu-promoted intramolecular S_N_2 furnishes oxetane **3**.

**Scheme 3:**
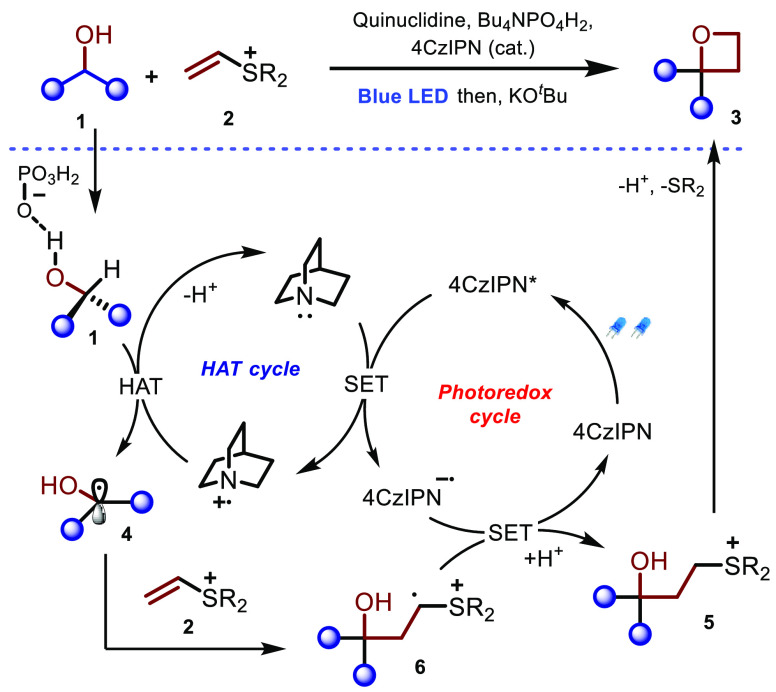
Proposed Reaction Mechanism

In conclusion, this report describes a versatile and practical methodology for the direct conversion of inactivated sp^3^ alcohols into oxetanes. The chemistry is general, occurs under remarkably mild conditions, is applicable to the functionalization of unmodified complex molecules, and can streamline synthetic routes toward bioactive molecules, as demonstrated by the one-step conversion of testosterone into the bioactive steroid **3ab**. The novel methodology presented in this report is expected to find various applications in synthesis and medicinal chemistry.

## Supporting Information Available

Spectral data for all compounds; additional experimental details; materials; and methods, including photographs of experimental setup (PDF)
