# Supplementary material for: Oxetane Synthesis via Alcohol C–H Functionalization
Source: J Am Chem Soc. 2023 Jul 18;145(29):15688–94. doi: 10.1021/jacs.3c04891 (PMC10375527; doi:10.1021/jacs.3c04891)

Supporting Information for

Oxetane Synthesis via Alcohol C-H Functionalization

Subhasis Paul,[§] Dario Filippini,[§] Filippo Ficarra,[§] Heorhii Melnychenko,[§] Christopher Janot[‡] and Mattia Silvi[§]

§ The GSK Carbon Neutral Laboratories for Sustainable Chemistry, University of Nottingham, Jubilee Campus, Nottingham, NG7 2TU, UK; School of Chemistry, University of Nottingham, University Park, Nottingham, NG7 2RD, UK.

‡ Chemical Development, Pharmaceutical Technology and Development, Operations, AstraZeneca, Macclesfield, SK10 2NA, UK.

Correspondence to: mattia.silvi@nottingham.ac.uk

Table of Contents

1.1. Solvents, reagents, and starting materials S2
1.2. Chromatography and instrumental analysis S2
1.3. Naming of compounds S2
1.4. LEDs S3
2. Synthesis of the photocatalysts and novel starting materials S3
2.1. Synthesis of 4CzIPN S3
2.2. Synthesis of dineopentyl(vinyl)sulfonium triflate (**2b**) S4
2.3. Synthesis of dineopentyl(prop-1-en-1-yl)sulfonium triflate (**2g**) and dineopentyl(prop-1-en-2-yl)sulfonium triflate (**2h**) S6
2.4. Synthesis of dineopentyl(1-phenylvinyl)sulfonium tetrafluoroborate (**2i**) S8
2.5. Synthesis of (2-hydroxy-7-azaspiro[3.5]nonan-7-yl)(phenyl)methanone (**1k**) S8
2.6. Synthesis of 6-hydroxy-2-azaspiro[3.3]heptan-2-yl)(phenyl)methanone (**1j**) S9
2.7. Synthesis of 17-bromoheptadecan-7-ol S10
2.8. Synthesis of 12-hydroxyoctadecanenitrile (**1r**) S11
2.9. Synthesis of 6-((*tert*-butyldiphenylsilyl)oxy)hexan-2-ol (**1q**) S11
3. Optimization studies S12
3.1. Investigation of the effect of the sulfonium structure S12
3.2. Investigation of the effect of standard reaction parameters S12
3.3. Investigation of the $ZnCl_2$ – quinuclidine system S13
3.4. Optimization studies on primary alcohol substrates S14
3.5. Investigation of substituted vinyl systems S15
4. General procedures & product characterization S16
General procedure A S16
General Procedure B S17
4.1. Reaction set-up for irradiation of mixtures with blue LEDs S18
4.2. Reaction products and characterization S20
4.3. Elucidation of the stereochemistry of **3aa** S37
5. Scope limitations S39
6. Mechanistic investigations S39
6.1. Experiment carried out in the presence of TEMPO as radical inhibitor S39
6.2. Fluorescence quenching studies S40
6.3. Electrochemical studies S42
7. References and Notes S44
8. NMR Spectra S45

1.1. Solvents, reagents, and starting materials

All air and water-sensitive reactions were carried out in oven-dried glassware under argon atmosphere using standard Schlenk manifold technique. The solvents were degassed when needed by bubbling argon for ten minutes. Bulk solutions were evaporated under reduced pressure using a Büchi rotary evaporator. All solvents were commercially supplied or provided by the communal stills of the School of Chemistry, University of Nottingham. Commercially available compounds were purchased from Sigma Aldrich, Alfa Aesar, Acros, Fluorochem, TCI chemicals and used as received. Dried solvents were purchased from Acros Organic, Extra Dry over molecular sieves, AcroSeal®.

Compounds 11-bromoundecanal[1], 2-bromo-2-phenylethyl acetate[2], **1e**[3], **1v**,[4] **2a**,[5] **2c**,[5] **2d**,[6] **2e**,[7] were synthesized following reported procedures.

1.2. Chromatography and instrumental analysis

Flash column chromatography (FCC) was carried out using Sigma-Aldrich silica gel LC60A-40 (63 µm). THF and Et_2O were distilled prior to use. All reactions were followed by thin-layer chromatography (TLC) when practical, using Merck Kieselgel 60 F_{254} fluorescent treated silica which was visualised under UV light, by staining with aqueous basic potassium permanganate, phosphomolybdic acid or with ninhydrin solution.

^{1}H-NMR, ^{13}C-NMR and **^{19}F-NMR** spectra were recorded using Bruker broadband prodigy cryoprobe AV(III)500HD 500 MHz and Bruker AV(III)400HD 400 MHz spectrometers. Chemical shifts (δ) are given in parts per million (ppm) and coupling constants (J) are given in hertz (Hz). The ^{1}H-NMR spectra are reported as follows: ppm (multiplicity, coupling constants, number of protons). High resolution mass spectra (**HRMS**) were recorded on a Bruker MicrOTOF II by Electrospray Ionisation (ESI) or on an Agilent 7890B gas chromatography system coupled with a Jeol AccuTOF GCx by Electron Ionisation (EI). **IR** spectra were recorded on a Bruker Vertex 70 FT-IR ATR as a thin film. Only selected absorption maxima (ν_{max}) are reported in wavenumbers (cm^{-1}). **Melting points** were recorded in degrees Celsius (°C), using a Stuart melting point SMP 20 microscope apparatus and are reported uncorrected. Optical rotation (**$[\alpha]_D^{25}$**) was recorded on an Anton Paar MCP 100 at 25 °C, in chloroform, with a concentration of 1g/100mL. **Fluorescence spectra** were recorded using a Edinburgh Instruments FLS980, equipped with a 450 W continuous xenon lamp (Xe1). **Cyclic voltammetry** experiments were carried out using a CH Instruments Model 600E potentiostat.

1.3. Naming of compounds

Compound names are generated by ChemDraw 20.0 software (PerkinElmer), following the IUPAC nomenclature.

1.4. LEDs

Irradiation of reaction mixtures was performed using Kessil lamp A160WE Tuna Blue Saltwater LED Light 40W set to blue at maximum intensity. Lamp emission is observed to decay over long usage due to LED chip deterioration. To ensure good reproducibility of the results, we recommend using new lamps.

2. Synthesis of the photocatalysts and novel starting materials

2.1. Synthesis of 4CzIPN

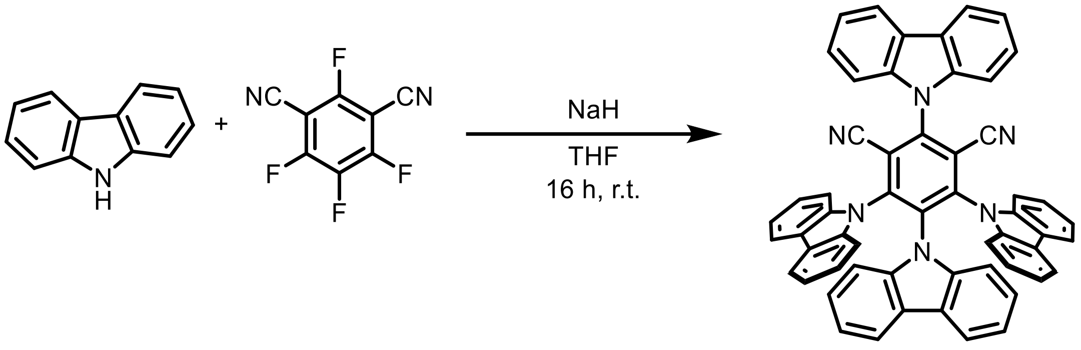

4CzIPN

4CzIPN was synthesised through a modification of a known procedure.[8]

NaH (60% in oil; 15.0 mmol; 600 mg) was slowly added to a stirred solution of 9-carbazole (10.0 mmol; 1.67 g) in dry THF (40 mL) under argon atmosphere at room temperature. After 30 min, tetrafluoroisophthalonitrile (2.00 mmol; 400 mg) was added. After stirring at room temperature for 16 h, 200 μL of distilled water were added to the mixture to quench the excess NaH. Then the solvent was evaporated under reduced pressure. The yellow powder was solubilized in dichloromethane and celite was added. The solvent was removed again under reduced pressure and the resulting powder was dry-loaded and purified through column chromatography (SiO_2; gradient 75:25 to 1:1 hexane:DCM) to afford 4CzIPN (1.2 g; 76%) as a bright yellow fine powder. **R_f** (1:1 hexane:DCM) 0.6; **^{1}H NMR** ($CDCl_3$, 500 MHz) δ (ppm): 8.23 (d, *J* = 7.7 Hz, 2H), 7.81 – 7.62 (m, 8H), 7.49 (ddd, *J* = 8.0, 6.8, 1.5 Hz, 2H), 7.33 (d, *J* = 7.7 Hz, 2H), 7.23 (dd, *J* = 6.8, 2.1 Hz, 4H), 7.12 – 7.05 (m, 8H), 6.89 – 6.78 (m, 4H), 6.64 (t, *J* = 7.8 Hz 2H); **^{13}C NMR** ($CDCl_3$, 126 MHz) δ (ppm): 145.4, 144.8, 140.1, 138.3, 137.1, 134.9, 127.1, 125.9, 125.1, 124.9, 124.7, 124.0, 122.6, 122.1, 121.6, 121.1, 120.6, 119.8, 116.5, 111.8, 110.1, 109.6, 109.6.

The spectra match the ones reported.[9]

2.2. Synthesis of dineopentyl(vinyl)sulfonium triflate (2b)

Dineopentyl(vinyl)sulfonium triflate (**2b**) was synthesized as reported in Scheme S1.

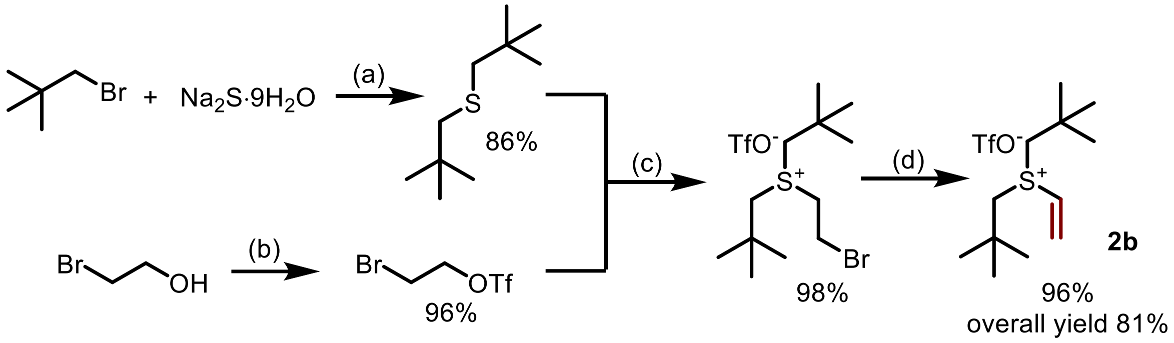

Scheme S1: Synthetic route for dineopentyl(vinyl)sulfonium triflate (a) tributylhexadecylphosphonium bromide (10 mol%), H_2O, 80 °C; (b) Tf_2O, Py, DCM, -20 °C to r.t.; (c) DCM, r.t.; (d) $KHCO_3$, THF:H_2O (2:1), r.t.

Synthesis of dineopentyl sulfide

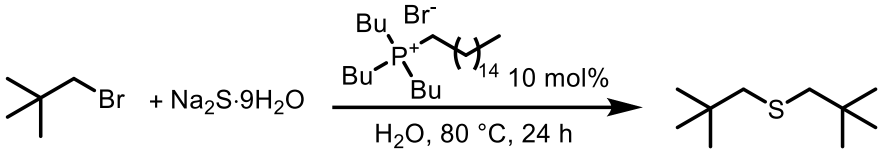

Dineopentyl sulfide was synthesised through a modification of a known procedure.[10]

$Na_2S·9H_2O$ (0.7 equiv.; 140 mmol; 33.6 g) and tributylhexadecylphosphonium bromide (0.1 equiv.; 20 mmol; 10.2 g) were loaded in a 250 mL round bottom flask equipped with a reflux condenser and dissolved in 60 mL of degassed water (degassed by argon sparging for 30 min). Neopentyl bromide (1.0 equiv.; 200 mmol; 25.2 mL) was added and the mixture was heated up to 80 °C for 24 h while stirring vigorously vigorous stirring. The mixture was cooled down to room temperature and the organic layer was separated and vacuum distilled (108 °C at 50 mbar). To further remove traces of water, after the distillation the compound was passed through a Pasteur filled with anhydrous $MgSO_4$ to obtain the product (15.0 g; 86%) as a transparent oil.

^{1}H NMR ($CDCl_3$, 500 MHz) δ (ppm): 2.45 (s, 4H), 0.98 (s, 18H); **^{13}C NMR** ($CDCl_3$, 126 MHz) δ (ppm): 50.1, 32.8, 29.1.

The spectra match the ones reported.[11]

Synthesis of 2-bromoethyl trifluoromethanesulfonate

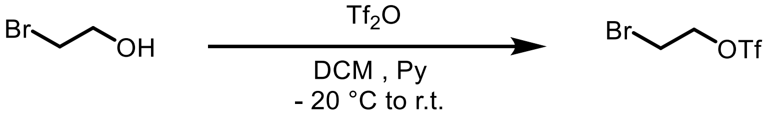

2-bromoethyl trifluoromethanesulfonate was synthesised following a known literature procedure.[5] Trifluoromethanesulfonic anhydride (1.04 equiv.; 83 mmol; 14 mL) was dropwise added to a stirred solution of pyridine (1.08 equiv.; 86 mmol; 7.0 mL) in dry DCM (76 mL) at -20 °C under argon atmosphere and the stirring was continued for 10 minutes. Then 2-bromoethanol (1.00 equiv.; 80 mmol; 5.7 mL) was slowly added to this cold reaction mixture. The cooling bath was removed, and

the mixture was left warming up to room temperature while stirring for 10 minutes (strictly following this time was found to be important). The resulting suspension was filtered, concentrated (using a rotary evaporator maintaining the water bath below 20 °C) and petroleum ether (50 mL) was added. The mixture was again filtered and concentrated under reduced pressure and finally dried under high vacuum to give the product (19.7 g; 96%) as a light brown oil which was used for the next step without further purification.

^{1}H NMR ($CDCl_3$, 400 MHz) δ (ppm): δ 4.74 (t, J = 6.4 Hz, 2H), 3.61 (t, J = 6.4 Hz, 2H); **^{19}F NMR** ($CDCl_3$, 376 MHz) δ (ppm): -74.5 (s); **^{13}C NMR** ($CDCl_3$, 101 MHz) δ (ppm): 74.4, 26.3.

The spectra match the ones reported.[5]

Synthesis of (2-bromoethyl)dineopentylsulfonium triflate

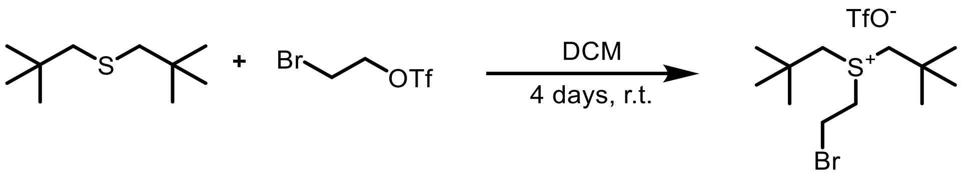

Dineopentyl sulfide (1.0 equiv.; 85.7 mmol; 15.0 g) was dissolved in dry DCM (40 mL) and then 2-bromoethyl trifluoromethanesulfonate (1.1 equiv.; 94.3 mmol; 24.2 g) was added and the reaction was left stirring for 4 days at room temperature and monitored *via* ^{1}H-NMR analysis of a minimal reaction aliquot. The solvent was removed by rotary evaporation and the white solid was washed three times with 70 mL of hexane to give the product (36.3 g; 98%) as a white solid, which was used for the next step without further purifications. **IR** (film) ν_{max}/cm^{-1}:2965, 2876, 1476, 1400, 1372, 1255, 1157, 1030, 907, 755, 705, 637, 572, 518; **M.P.** = 97 °C; **^{1}H NMR** ($CDCl_3$, 500 MHz) δ (ppm): δ 4.24 – 4.16 (m, 2H), 3.80 – 3.73 (m, 2H), 3.68 (d, J = 14.4 Hz, 2H), 3.47 (d, J = 14.3 Hz, 2H), 1.19 (s, 18H); **^{19}F NMR** ($CDCl_3$, 376 MHz) δ (ppm): -78.4 (s); **^{13}C NMR** ($CDCl_3$, 126 MHz) δ (ppm): 58.1, 49.0, 32.9, 29.0, 24.9.**HRMS** (ESI-TOF) mass calculated for $[M]^+$ ($C_{12}H_{26}BrS^+$) expected *m/z* 281.0933; found *m/z* 281.0929.

Synthesis of dineopentyl(vinyl)sulfonium triflate (2b)

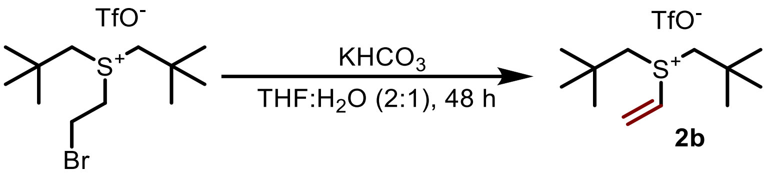

(2-bromoethyl)dineopentylsulfonium triflate (1.0 equiv.; 84.1 mmol; 36.3 g) was dissolved in 170 mL of a 2:1 mixture of THF and water. $KHCO_3$ (1.5 equiv.; 126.1 mmol; 12.6 g) was added and the reaction was left stirring for 2 days at room temperature and monitored *via* ^{1}H-NMR analysis of a minimal reaction aliquot. The reaction mixture was extracted in DCM, the organic phases collected, dried over anhydrous $MgSO_4$ and the solvent was removed through rotary evaporation to give compound **2b** (28.4 g; 96%) as a white solid (see Fig. S1), which does not require further purifications. **IR** (film) ν_{max}/cm^{-1}: 3046, 2966, 2876, 1602, 1482, 1373, 1255, 1224, 1154, 1033, 639; **M.P.** = 139 °C; ^{1}**H NMR** ($CDCl_3$, 500 MHz) δ (ppm): 7.44 – 7.35 (m, 1H), 6.41 – 6.31 (m, 2H), 3.87 (d, *J* = 14.0 Hz, 2H), 3.43 (d, *J* = 14.0 Hz, 2H), 1.16 (s, 18H); ^{19}**F NMR** ($CDCl_3$, 376 MHz) δ (ppm): -78.4 (s); ^{13}**C NMR** ($CDCl_3$, 126 MHz) δ (ppm): 135.5, 128.0, 60.5, 33.1, 29.0. **HRMS** (ESI-TOF) mass calculated for $[M]^+$ ($C_{12}H_{25}S^+$) expected *m/z* 201.1671; found *m/z* 201.1670.

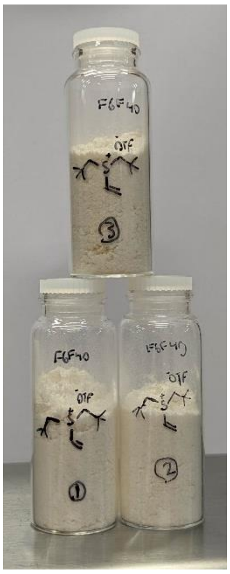

Fig. S1: compound **2b**

2.3. Synthesis of dineopentyl(prop-1-en-1-yl)sulfonium triflate (2g) and dineopentyl(prop-1-en-2-yl)sulfonium triflate (2h)

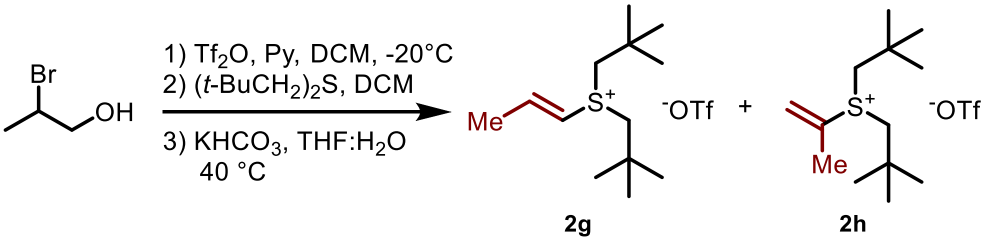

Trifluoromethanesulfonic anhydride (1.02 equiv.; 24.3 mmol; 4.1 mL) was dropwise added to a stirred solution of pyridine (1.03 equiv.; 24.7 mmol; 2.0 mL) in dry DCM (40 mL) at -20 °C under argon atmosphere and the stirring was continued for 10 minutes. Then 2-bromopropan-1-ol (1 equiv.; 23.9 mmol; 3.3 g) was slowly added to this cold reaction mixture. The cooling bath was removed, and the mixture was left warming up to room temperature while stirring for other 10 minutes. The resulting suspension was filtered and concentrated (using a rotary evaporator, maintaining the water bath at 20 °C). Petroleum ether (100 mL) of was added to the oil and stirred. The oil was decanted and the supernatant liquid collected. The procedure was repeated three times. The organic fractions

were combined and evaporated using a rotary evaporator, maintaining the water bath at 20 °C. The resulting crude oil was submitted for the next step without further purification.

The crude was dissolved in dry DCM (12 mL) and then dineopentyl sulfide (12 mmol; 2.1 g) was slowly added to this reaction mixture. The reaction was left stirring at room temperature for 2 days monitoring the conversion *via* ^{1}H-NMR analysis of a minimal reaction aliquot. The solvent was removed by rotary evaporation and petroleum ether (20 mL) was added. The mixture was stirred, the oil decanted and the organic solvent discarded. The procedure was repeated three times and then the crude was submitted to the next step without further purifications.

The crude from the previous step was dissolved in 15 mL of a 2:1 mixture of THF and water. $KHCO_3$ (23 mmol; 2.29 g) was added and the reaction was left stirring at 40 °C monitoring the conversion *via* ^{1}H-NMR analysis of a minimal reaction aliquot (full conversion observed in 16 h). THF was removed under reduced pressure and the reaction mixture was extracted in DCM (25 mL × 3), the organic phases collected, dried over anhydrous $MgSO_4$ and the solvent was removed through rotary evaporation. Chromatography purification (eluent 95:5 to 93:7 DCM:MeOH) to give compounds **2g**, 255.2 mg, and **2h**, 1.31 g, 18% overall yield of three steps. The formation of the two isomers is likely due to the intervention of a bromonium equilibrium at the triflation and sulfonium formation steps.

Compound **2g**: pale yellow solid. **R$_f$** (90:10 DCM:MeOH) 0.4; **IR** (film) ν_{max}/cm^{-1}: 2969, 1480, 1398, 1263, 1224, 1153, 1033, 963, 639, 573, 518; **M.P.** = 116 °C; **^{1}H NMR** ($CDCl_3$, 400 MHz) δ (ppm): 7.01 (d, *J* = 14.9 Hz, 1H), 6.83 – 6.72 (m, 1H), 3.80 (d, *J* = 14.0 Hz, 2H), 3.33 (d, *J* = 14.0 Hz, 2H), 2.06 (dd, *J* = 6.8, 1.6 Hz, 3H), 1.14 (s, 18H); **^{19}F NMR** ($CDCl_3$, 376 MHz) δ (ppm): -78.4 (s); **^{13}C NMR** ($CDCl_3$, 126 MHz) δ (ppm): 150.1, 119.3, 60.5, 33.0, 29.0, 19.2; **HRMS** (ESI-TOF) mass calculated for $[M]^+$ ($C_{13}H_{27}S^+$) expected *m/z* 215.1828; found *m/z* 215.1826.

Compound **2h**: white solid. **R$_f$** (90:10 DCM:MeOH) 0.35; **IR** (film) ν_{max}/cm^{-1}: 2964, 1471, 1397, 1373, 1260, 1225, 1152, 1034, 754, 640, 572, 517; **M.P.** = 125 °C; **^{1}H NMR** ($CDCl_3$, 400 MHz) δ (ppm): 6.23 – 6.20 (m, 1H), 6.09 – 6.01 (m, 1H), 3.70 – 3.55 (m, 4H), 2.41 (br. s, 3H), 1.17 (s, 18H); **^{19}F NMR** ($CDCl_3$, 376 MHz) δ (ppm): -78.3 (s); **^{13}C NMR** ($CDCl_3$, 101 MHz) δ (ppm): 134.9, 133.1, 56.8, 33.1, 28.8, 17.4; **HRMS** (ESI-TOF) mass calculated for $[M]^+$ ($C_{13}H_{27}S^+$) expected *m/z* 215.1828; found *m/z* 215.1833.

2.4. Synthesis of dineopentyl(1-phenylvinyl)sulfonium tetrafluoroborate (2i)

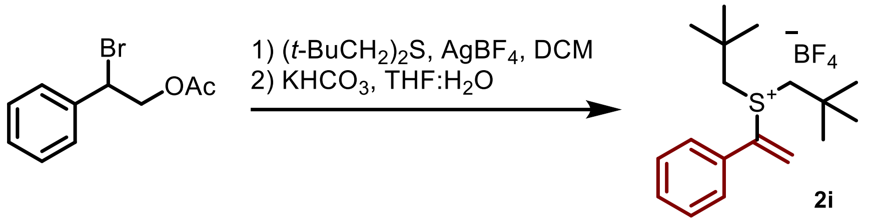

To a solution of 2-bromo-2-phenylethyl acetate (1.0 equiv.; 19.6 mmol; 4.77 g) and dineopentyl sulfide (1.0 equiv.; 19.6 mmol; 3.42 g) in dry DCM (20 mL) was added $AgBF_4$ (1.0 equiv.; 19.6 mmol; 3.82 g), at 0 °C. The mixture was allowed to warm to room temperature and stirred further for 20 h under argon. The mixture was then filtered over celite and concentrated. The resulting crude oil was washed with hexane (20 mL × 2) and once with MTBE (20 mL). The crude material was used for the next step without further purification.

The crude oil from the previous step was dissolved in (40 mL) of a 2:1 mixture of THF and H_2O. $KHCO_3$ (26.0 mmol; 2.60 g) was added and the reaction was stirred at room temperature monitoring the conversion *via* ^{1}H-NMR analysis of a minimal reaction aliquot (full conversion observed in 18 h). The volatiles were removed under reduced pressure, and the aqueous solution was extracted with DCM (40 mL × 3). The combined organic layers were dried over anhydrous $MgSO_4$, filtered over celite, and concentrated under reduce pressure. The crude product was washed with MTBE (40 mL × 2), then recrystallized in *iso*-propanol giving compound **2i** as a colourless solid (3.24 g, 45%). **IR** (film) ν_{max}/cm^{-1}: 2965, 2883, 1494, 1471, 1395, 1374, 1217, 1054, 774, 707, 522; **M.P.** = 178 °C (decomposition); **^{1}H NMR** ($CDCl_3$, 500 MHz) δ (ppm): 7.55 – 7.50 (m, 5H), 6.80 (d, *J* = 4.1 Hz, 1H), 6.62 (d, *J* = 4.1 Hz, 1H), 3.92 (d, *J* = 14.2 Hz, 2H), 3.74 (d, *J* = 14.2 Hz, 2H), 1.13 (s, 18H); **^{19}F NMR** ($CDCl_3$, 376 MHz,) δ -149.9 (s), -150.0 (s); **^{13}C NMR** ($CDCl_3$, 101 MHz) δ (ppm): 138.9, 132.8, 131.5, 130.1, 130.0, 127.1, 61.3, 33.2, 29.0; **HRMS** (ESI-TOF) mass calculated for $[M]^+$ ($C_{18}H_{29}S^+$) expected *m/z* 277.1984; found *m/z* 277.1992.

2.5. Synthesis of (2-hydroxy-7-azaspiro[3.5]nonan-7-yl)(phenyl)methanone (1k)

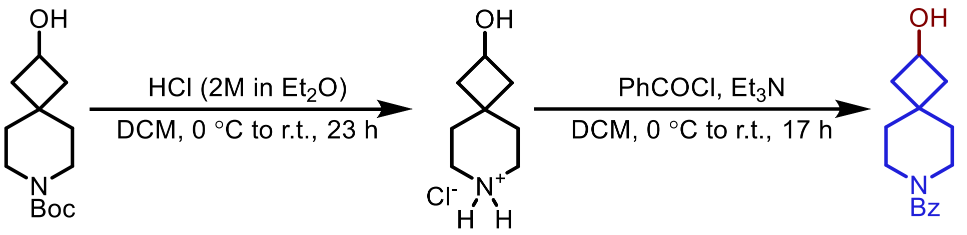

1k

tert-butyl 2-hydroxy-7-azaspiro[3.5]nonane-7-carboxylate (1.0 equiv.; 2.0 mmol; 500.0 mg) was dissolved in dry DCM (0.5 M, 4.1 mL) and cooled down at 0 °C. A solution of HCl (2M in Et_2O) (24.0 equiv.; 49.7 mmol; 24.9 mL) was slowly added to the mixture at 0 °C. The reaction mixture was slowly allowed to warm up at room temperature and stirred for 23 h. The solution was then concentrated under reduced pressure. To this mixture, Et_2O (50 mL) was added and the solid filtered

out. The solid was washed thoroughly with Et_2O (3 × 20 mL) and dried under vacuum to obtain the chloride salt (338.5 mg; 92%) as white solid. **IR** (film) ν_{max}/cm^{-1}: 3181, 2959, 2924, 2808, 2714, 2478, 1590, 1436, 1336, 1234, 1181, 1158, 1080, 973, 947, 899, 676, 546, 477; **^{1}H NMR** (CD_3OD, 500 MHz) δ (ppm): 4.26 (p, *J* = 7.2 Hz, 1H), 3.15 – 3.04 (m, 4H), 2.36 – 2.28 (m, 2H), 1.85 – 1.72 (m, 6H); **^{13}C NMR** (CD_3OD, 126 MHz) δ (ppm): 62.9, 42.8, 42.7, 42.5, 36.9, 34.1, 29.9; **HRMS** (ESI-TOF) mass calculated for $[M]^+$ ($C_8H_{16}NO^+$) expected *m/z* 142.1226; found *m/z* 142.1218.

2-Hydroxy-7-azaspiro[3.5]nonan-7-ium chloride (1.0 equiv.; 0.5 mmol; 89.0 mg) was dissolved in DCM (0.5 M, 1.0 mL) and cooled down at 0 °C. Then Et_3N (2.5 equiv.; 1.25 mmol; 174.2 μL) was added to the mixture followed by dropwise addition of benzoyl chloride (1.1 equiv.; 0.55 mmol; 64.0 μL) at 0 °C. The reaction mixture was allowed to slowly warm up at room temperature and stirred for 17 h. The reaction was quenched by water (15 mL) and extracted with DCM (3 × 20 mL). Combined organic layers were washed with 2M HCl solution (2 × 10 mL) followed by saturated $NaHCO_3$ solution (2 × 10 mL), water (15 mL) and brine (15 mL), dried over anhydrous $MgSO_4$, filtered and the solvent was removed under reduced pressure. The crude product was purified by flash column chromatography (SiO_2; gradient 20:80 to 1:99 hexane:EtOAc) to afford compound **1k** (90.6 mg; 74%) as a white solid. **M. P.** = 122-123 °C; **R_f** (20:80 hexane:EtOAc) 0.2; **IR** (film) ν_{max}/cm^{-1}: 3390, 2921, 2849, 1613, 1576, 1445, 1372, 1275, 1055, 603, 568; **^{1}H NMR** ($CDCl_3$, 400 MHz) δ (ppm): 7.38 (br. s, 5H), 4.34 (br. s, 1H), 3.83 – 3.10 (m, 4H), 2.42 – 2.21 (m, 2H), 1.89 – 1.65 (m, 4H), 1.61 – 1.45 (m, 2H); **^{13}C NMR** ($CDCl_3$, 126 MHz) δ (ppm): 170.5, 136.4, 129.6, 128.6, 127.0, 63.3, 45.2, 42.7, 39.5, 36.5, 30.7; **HRMS** (ESI-TOF) mass calculated for $[M+Na]^+$ ($C_{15}H_{19}NO_2Na^+$) expected *m/z* 268.1308; found *m/z* 268.1307.

2.6. Synthesis of 6-hydroxy-2-azaspiro[3.3]heptan-2-yl)(phenyl)methanone (1j)

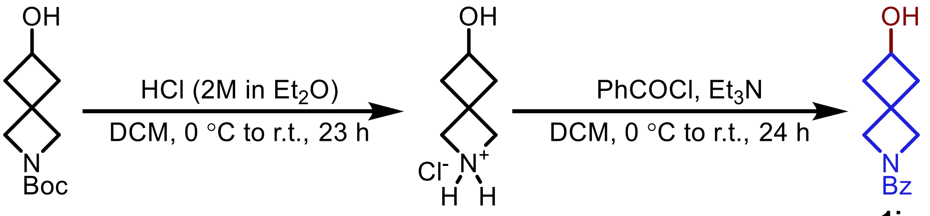

1j

tert-butyl 6-hydroxy-2-azaspiro[3.3]heptane-2-carboxylate (1.0 equiv.; 0.5 mmol; 107 mg) was dissolved in dry DCM (0.5 M, 4.1 mL) and cooled down at 0 °C. A solution of HCl (2M in Et_2O) (24.0 equiv.; 49.7 mmol; 24.9 mL) was slowly added to the mixture at 0 °C. The reaction mixture was slowly allowed to warm up at room temperature and stirred for 23 h. The solution was then concentrated under reduced pressure. To this mixture, Et_2O (50 mL) was added and the solid filtered out. The solid was washed thoroughly with Et_2O (3 × 20 mL) and dried under vacuum to obtain the hydrochloride salt which was used directly in the next step without further purification. The hydrochloride salt was dissolved in DCM (0.5 M, 1.0 mL) and cooled down at 0 °C. Then Et_3N (2.5

equiv.; 1.25 mmol; 174.2 μL) was added to the mixture followed by dropwise addition of benzoyl chloride (1.1 equiv.; 0.55 mmol; 64.0 μL) at 0 °C. The reaction mixture was allowed to slowly warm up at room temperature and stirred for 24 h. The reaction was quenched by water (15 mL) and extracted with DCM (3 × 20 mL). Combined organic layers were washed with 2M HCl solution (2 × 10 mL) followed by saturated $NaHCO_3$ solution (2 × 10 mL), water (15 mL) and brine (15 mL), dried over anhydrous $MgSO_4$, filtered and the solvent was removed under reduced pressure. The crude product was purified by flash column chromatography (SiO_2; gradient 30:70 to 0:100 hexane:EtOAc) to afford compound **1j** as a white solid (23.7 mg, 22%). **R_f** (EtOAc) 0.2; **M.P.** = 97 – 100 °C; **IR** (film) ν_{max}/cm^{-1}: 3391, 2928, 2869, 1728, 1615, 1572, 1452, 1432, 1053, 791, 711; **^{1}H NMR** ($CDCl_3$, 500 MHz) δ (ppm): 7.61 – 7.58 (m, 2H), 7.46 –7.43 (m, 1H), 7.40 – 7.37 (m, 2H), 4.24 – 4.14 (m, 5H), 2.61 – 2.53 (m, 2H), 2.17 – 2.07 (m, 2H); **^{13}C NMR** (126 MHz, $CDCl_3$) rotameric mixture, resonances for minor rotamer are enclosed in parenthesis () δ(ppm): 170.3, 133.3, 131.1, 128.5, 128.0, (65.9), 64.6, 62.2, 61.2, (59.9), (44.1), 43.9, 30.4; **HRMS** (ESI-TOF) mass calculated for $[M+Na]^+$ ($C_{13}H_{15}NO_2Na^+$) expected *m/z* 240.0995l; found *m/z* 240.0994.

2.7. Synthesis of 17-bromoheptadecan-7-ol

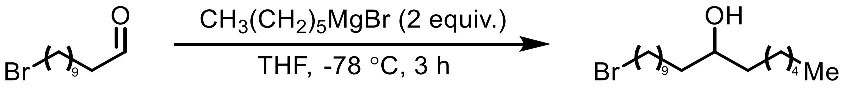

11-bromoundecanal (1 equiv., 13.8 mmol, 3.45 g) was dissolved in dry THF (20 mL) and cooled down to -78 °C. A solution of hexylmagnesium bromide (0.8 M in THF, 27.6 mmol, 34.5 mL) was added dropwise at -78 °C. The reaction mixture was then stirred at -78 °C for 3 h. The mixture was quenched at -78 °C, by addition of a saturated NH_4Cl solution (25 mL), and extracted with EtOAc (3 x 25 mL). The organic layers were combined and dried over $MgSO_4$, filtered, and concentred under reduced pressure. The residue was purified by flash column chromatography (SiO_2; 95:5 to 90:10 hexane:EtOAc) to afford the compound (1.08 g, 24%) as a white solid. **R_f** (90:10 hexane:EtOAc) 0.5; **M.P.** = 48 – 52 °C; **IR** (film) ν_{max}/cm^{-1}: 3355, 2922, 2851, 1694, 1466, 1052, 884, 859, 723; **^{1}H NMR** ($CDCl_3$, 500 MHz) δ (ppm): 3.61 – 3.55 (m, 1H), 3.41 (t, *J* = 7.0 Hz, 2H), 1.88 – 1.82 (m, 2H), 1.47 – 1.38 (m, 8H), 1.34 – 1.25 (m, 18 H), 0.88 (t, *J* = 7.1 Hz, 3H); **^{13}C NMR** ($CDCl_3$, 101 MHz) δ (ppm): 72.2, 37.7, 37.6, 34.2, 33.0, 32.0, 29.8, 29.7, 29.6, 29.6, 29.5, 28.9, 28.3, 25.8, 25.8, 22.8, 14.2; **HRMS** (ESI-TOF) mass calculated for $[M+Na]^+$ ($C_{17}H_{35}BrONa^+$) expected *m/z* 357.1763; found *m/z* 357.1764.

2.8. Synthesis of 12-hydroxyoctadecanenitrile (1r)

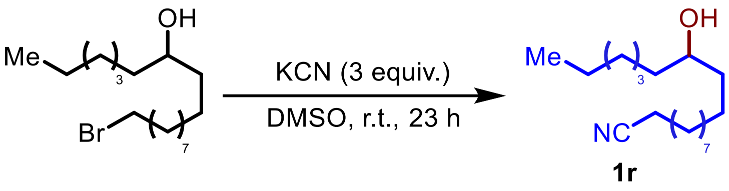

KCN (3 equiv., 3 mmol, 195 mg) was added to a solution of 17-bromoheptadecan-7-ol (1 equiv., 1 mmol, 335 mg) in dry DMSO (10 mL) at room temperature. The mixture was stirred at room temperature for 23 h. Et_2O (15 mL) was then added to the mixture and the organic layers were washed with water (3 x 10 mL)) and once with brine (10 mL). The organic solvent was dried over Na_2SO_4, filtered and concentrated under reduced pressure. The residue was purified by flash column chromatography (SiO_2; 90:10 to 90:20 hexane:EtOAc) to afford compound **1r** (253 mg, 90%) as a white solid. **R_f** (90:10 hexane:EtOAc) 0.2; **M.P.**: 56 – 60 °C; **IR** (film) ν_{max}/cm^{-1}: 3324, 2917, 2849, 2244, 1466, 1421, 1132, 1076, 1032, 997, 916, 862, 723; **^{1}H NMR** ($CDCl_3$, 500 MHz) δ (ppm): 3.61 – 3.56 (m, 1H), 2.33 (t, *J* = 7.2 Hz, 2H), 1.65 (quint, *J* = 7.2 Hz, 2H), 1.47 – 1.38 (m, 8H), 1.34 – 1.25 (m, 18 H), 0.89 (t, *J* = 7.1 Hz, 3H); **^{13}C NMR** ($CDCl_3$, 126 MHz) δ (ppm): 120.0, 72.2, 37.7, 37.6, 32.0, 29.8, 29.7, 29.6, 29.5, 29.4, 28.9, 28.8, 25.8, 25.5, 22.8, 17.3, 14.2; **HRMS** (ESI-TOF) mass calculated for $[M+Na]^+$ ($C_{18}H_{35}NONa^+$) expected *m/z* 304.2611; found *m/z* 304.2610.

2.9. Synthesis of 6-((*tert*-butyldiphenylsilyl)oxy)hexan-2-ol (1q)

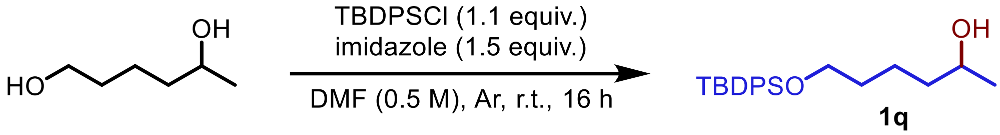

Hexane-1,5-diol (1.0 equiv.; 1.0 mmol; 120 µL) was dissolved in dry DMF (0.5 M, 2.0 mL). Imidazole (1.5 equiv.; 1.5 mmol; 102.4 mg) was added at room temperature, followed by a slow addition of *tert*-butyldimethylsilyl chloride (1.1 equiv.; 1.1 mmol; 286 µL). The reaction mixture was stirred at room temperature for 16 h. Then reaction mixture was diluted with 30 mL ethyl acetate. The organic phase was washed with water (4 × 30 mL) and brine (20 mL), dried over of anhydrous $MgSO_4$, filtered and the solvent was removed under reduced pressure. The crude product was purified by flash column chromatography (SiO_2; gradient 80:20 to 75:25 hexane:EtOAc) to afford compound **1q** (342.6 mg; 96%) as a transparent oil. **R_f** (80:20 hexane:EtOAc) 0.3; **IR** (film) ν_{max}/cm^{-1}: 3351, 3071, 2930, 2857, 1472, 1428, 1109, 1007, 823, 739, 701, 613, 505; **^{1}H NMR** ($CDCl_3$, 400 MHz) δ (ppm): 7.71 – 7.63 (m, 4H), 7.46 – 7.34 (m, 6H), 3.82 – 3.72 (m, 1H), 3.68 (t, *J* = 6.4 Hz, 2H), 1.63 – 1.53 (m, 2H), 1.47 – 1.35 (m, 5H), 1.18 (d, *J* = 6.2 Hz, 3H), 1.05 (s, 9H); **^{13}C NMR** ($CDCl_3$, 126 MHz) δ (ppm): 135.7, 134.2, 129.7, 127.7, 68.2, 63.9, 39.1, 32.6, 27.0, 23.6, 22.1, 19.4; **HRMS** (ESI-TOF) mass calculated for $[M+Na]^+$ ($C_{22}H_{32}O_2SiNa^+$) expected *m/z* 379.2064; found *m/z* 379.2063.

3. Optimization studies

3.1. Investigation of the effect of the sulfonium structure

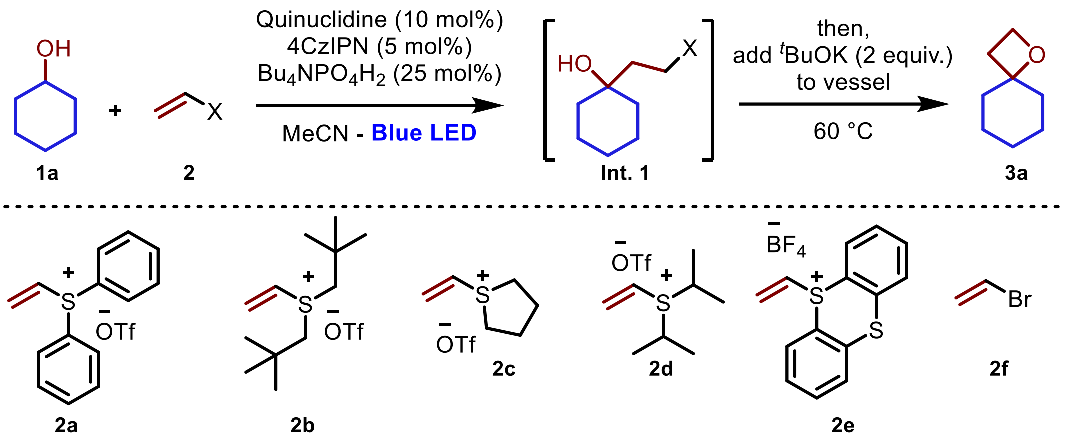

Entry[a]	2	Int. 1 formed (%)[b]	Yield 3a (%)[b]
1[c]	**2a**	<5	<5
2	**2b**	99	99
3	**2c**	<5	<5
4	**2d**	<5	<5
5	**2e**	<5	<5
6	**2f**	<5	<5

[a] Reactions performed in 0.1 mmol scale, using **1a** (1.0 equiv.), **2** (1.5 equiv.), [**1a**]$_0$ = 0.5 M. [b] Unless otherwise stated, ^{1}H-NMR yield using mesitylene or CH_2Br_2 as internal standards. [c] **1a** (2.0 equiv.), **2** (1.0 equiv.), [**2a**]$_0$ = 0.5 M, Ir[dF(CF$_3$)(ppy)$_2$dtbpy]$^+$ (1 mol%)

3.2. Investigation of the effect of standard reaction parameters

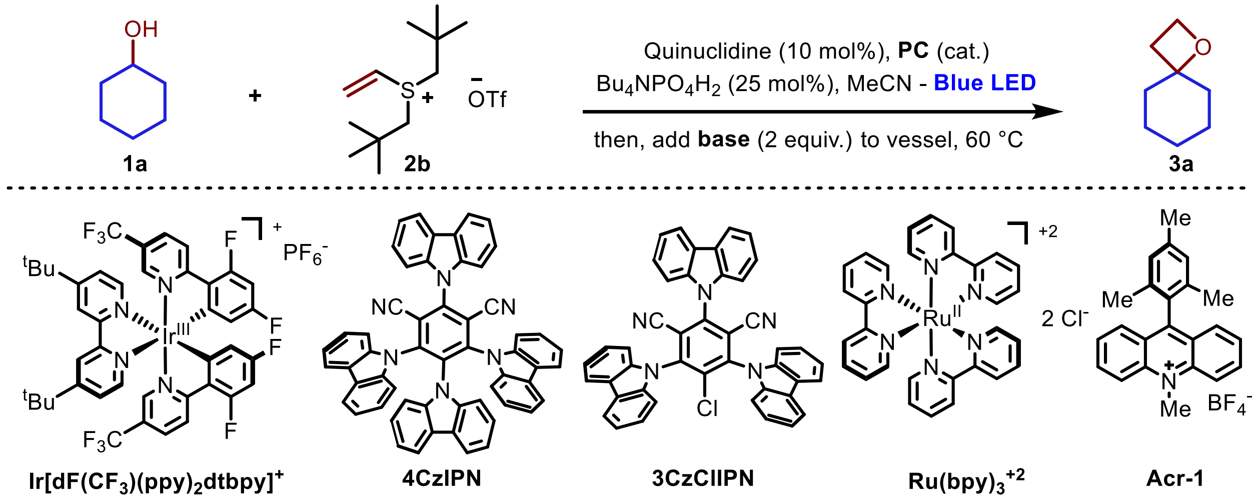

Entry[a]	PC	Stochiometry 1a:2	Base	Yield 3a (%)[b]
1	**Ir[dF(CF$_3$)(ppy)$_2$dtbpy]$^+$** (1 mol%)	2:1	^{t}BuOK	97
2[c]	**Ir[dF(CF$_3$)(ppy)$_2$dtbpy]$^+$** (1 mol%)	2:1	K_3PO_4	72
3	**Ir[dF(CF$_3$)(ppy)$_2$dtbpy]$^+$** (1 mol%)	1:1.5	^{t}BuOK	99
4	**4CzIPN** (5 mol%)	1:1.5	^{t}BuOK	99
5	**3CzClIPN** (5 mol%)	1:1.5	^{t}BuOK	82
6	**Ru(bpy)$_3^{+2}$** (1 mol%)	1:1.5	^{t}BuOK	0
7	**Acr-1** (5 mol%)	1:1.5	^{t}BuOK	0

[a] Reactions performed in 0.1 mmol scale, using **1a** (1.0 equiv.), **2** (1.5 equiv.), [**1a**]$_0$ = 0.5 M.; irradiation time, 24 h, cyclization time 24 h. [b] Unless otherwise stated, ^{1}H-NMR yield using CH_2Br_2 as internal standards. [c] After base addition, cyclization carried out at 80 °C for 41 h.

3.3. Investigation of the $ZnCl_2$ – quinuclidine system

In substrates bearing hydrogen bonding accepting moieties (relatively activated carbonyls and sulfonyl groups, i.e., **3d**), the standard catalytic system led to the products in low yields, with starting material recovery accounting for the remaining mass balance, suggesting an inefficient photochemical process.

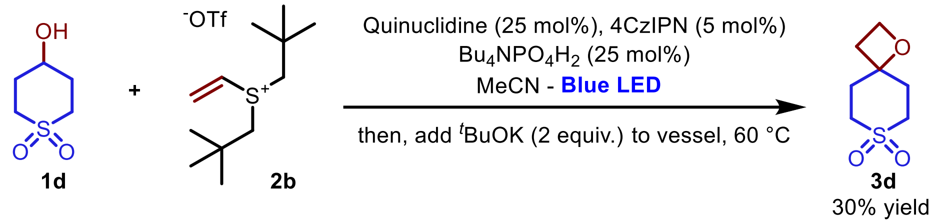

An alternative catalytic system, using stoichiometric $ZnCl_2$ and K_3PO_4 in place of $Bu_4NPO_4H_2$, was found to provide the products in improved yields. Optimization studies for this system are presented below, yield of **Int. 2** was monitored by LC-MS.

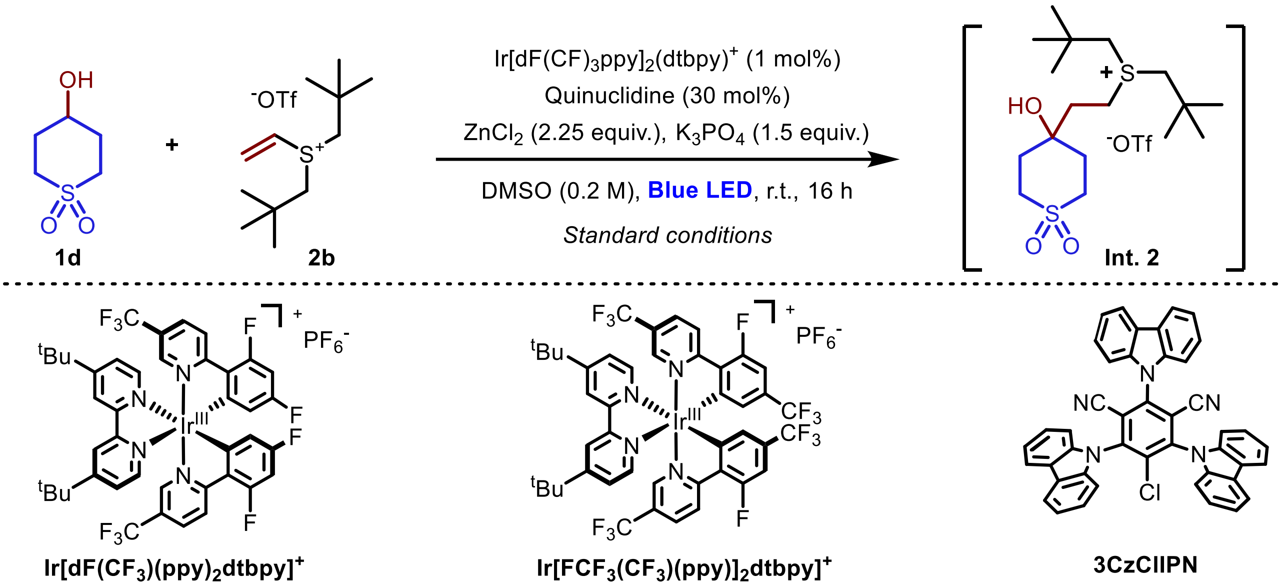

Entry[a]	Variation from standard conditions	Yield of Int. 2 (%)[b]
1	Standard Conditions (SC)	61
2	2 mol% Ir[dF(CF$_3$)ppy]$_2$(dtbpy)$^+$ used as photocatalyst	52
3	1 mol% Ir[FCF$_3$(CF$_3$)ppy]$_2$(dtbpy)$^+$ used as photocatalyst	50
4	5 mol % 3CzClIPN used as photocatalyst	48
5	2.5 equiv. of **2b** used	60
6	5 equiv. of $ZnCl_2$ used	58
7	After 16 h under SC, 1 mol% Ir[dF(CF$_3$)ppy]$_2$(dtbpy)$^+$ and 30 mol% quinuclidine were added, and irradiated for further 24 h	49

[a] Reactions performed in 0.1 mmol. A PF_6^- counterion is intended for the Ir photocatalyst. [b] Yield determined using LCMS with isopentyl dineopentyl sulfonium triflate as an internal standard.

3.4. Optimization studies on primary alcohol substrates

Primary alcohols proved to be challenging substrates, so additional optimization studies were performed. Initial investigations focused on maximizing the yield of the radical process, below an investigation of the effect of the reaction medium on the formation of intermediate **Int. 3** is presented.

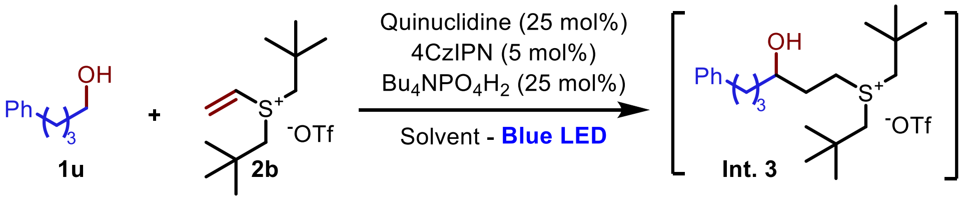

Entry[a]	Solvent	Yield of Int. 3 (%)[b]
1	DCM	70
2	$CHCl_3$	39
3	EtOAc	50
4	Acetone	53
5	MeCN	70
6	DMSO	58

[a] Reactions performed in 0.1 mmol scale, using **1a** (1.0 equiv.), **2** (1.5 equiv.), $[\mathbf{1a}]_0$ = 0.5 M.; irradiation time, 24 h. [b] Unless otherwise stated, ^{1}H-NMR yield using CH_2Br_2 as internal standard.

Then, an investigation of the reaction conditions was performed on the cyclization step. When a different solvent was investigated, the solvent was exchanged *in-situ* in the reaction vessel after irradiation using a standard Schlenk line manifold to apply vacuum.

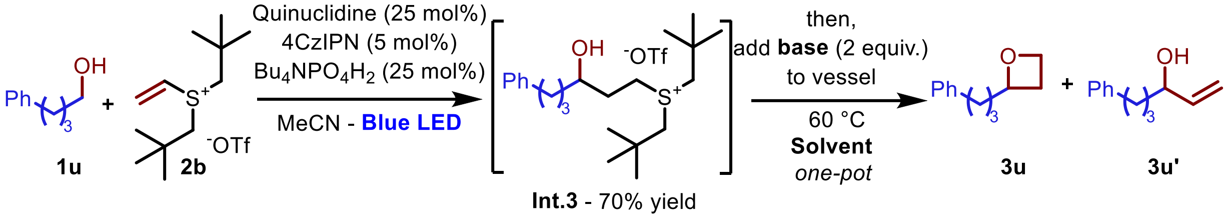

Entry[a]	Solvent	Base	Yield 3u (%)[b]	Yield of 3u' (%)[b]
1[c]	MeCN	K_2CO_3	Traces	0
2[c]	MeCN	K_3PO_4	21	8
3[d]	THF	NaH	36	18
4	MeCN	TMG	0	0
5	MeCN	^{t}BuOK	51	10
6	THF	^{t}BuOK	25	32
7	Dioxane	^{t}BuOK	31	24
8	DCE	^{t}BuOK	30	22
9	PhCl	^{t}BuOK	42	12

[a] Reactions performed in 0.1 mmol scale, using **1a** (1.0 equiv.), **2** (1.5 equiv.), $[\mathbf{1a}]_0$ = 0.5 M.; irradiation time, 24 h, cyclization time 24 h; [b] Unless otherwise stated, ^{1}H-NMR yield using CH_2Br_2 as internal standard. [c] Second step performed at 80 °C for 72 h. [d] Base addition performed at 0 °C and then the reaction was allowed to warm up to room temperature and left stirring for 24 h.

As no improvements were observed with other solvents, the full process was performed in MeCN, which also presented the advantage of not requiring a solvent exchange (base simply added to the vessel after irradiation without additional manipulation needed).

3.5. Investigation of substituted vinyl systems

To investigate the feasibility of using alkyl or aryl substituted vinyl sulfonium systems, the following substituents were introduced in the vinyl system: methyl in β- and α-position to test alkyl substitution, and α-phenyl to test aryl substitution. β-phenyl sulfoniums undergo radical reactivity with opposite regioselectivity and were not tested.[12]

Use of β-methyl substituted vinyl sulfonium **2g**

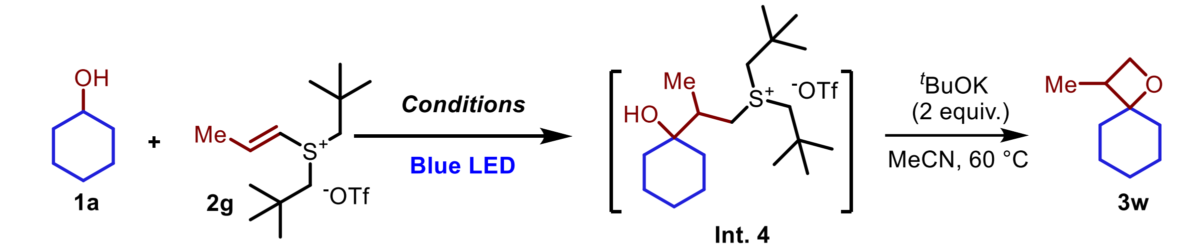

Entry[a]	Conditions	Yield of Int. 4 (%)[b]	Yield of 3w (%)[b]
1	Quinuclidine (25 mol%), 4CzIPN (5 mol%), $Bu_4NPO_4H_2$ (25 mol%), MeCN (0.5 M)	0	-
2	Quinuclidine (60 mol%), $Ir[dF(CF_3)ppy]_2(dtbpy)^+$ (2 mol%), K_3PO_4 (1.5 equiv.) $ZnCl_2$ (2.25 equiv.), DMSO (0.2 M)	40	35
3[c]	Quinuclidine (75 mol%), $Ir[dF(CF_3)ppy]_2(dtbpy)^+$ (3 mol%), K_3PO_4 (1.5 equiv.) $ZnCl_2$ (2.25 equiv.), DMSO (0.2 M)	51	44 (37)

[a] Unless otherwise stated, reactions performed in 0.05 mmol scale, using **1a** (1.0 equiv.), **2g** (1.5 equiv.); $[\mathbf{1a}]_0$ = 0.5 M irradiation time, 24 h. [b] Unless otherwise stated, ^{1}H-NMR yield using mesitylene or phenanthrene as internal standards. [c] Reaction performed on a 0.14 mmol scale. In parenthesis, isolated yield. See section 4.2 for experiment procedure.

Use of α-methyl substituted vinyl sulfonium **2h**

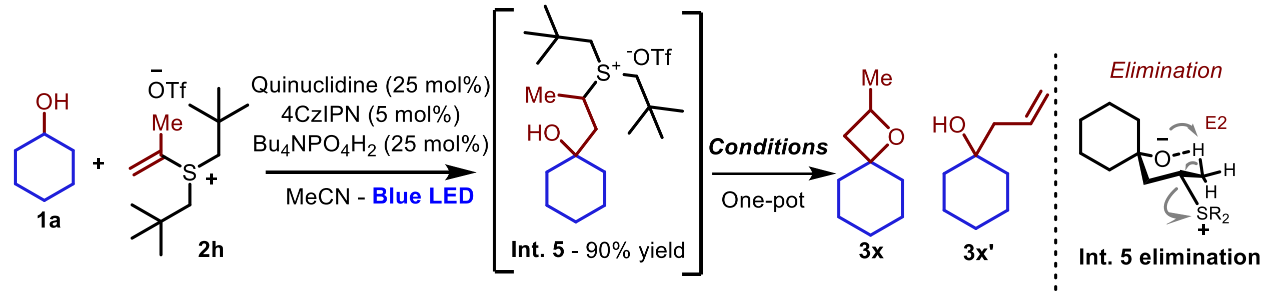

Entry[a]	Conditions	Yield of 3x (%)	Yield of 3x' (%)[b]
1	^{t}BuOK (2 equiv.), MeCN (0.5 M), 60 °C, 24 h	0	44
2	^{t}BuOK (2 equiv.), THF (0.15 M), r.t., 2 h[13]	0	45
3	*n*-BuLi (2 equiv.), THF (0.4 M), r.t., 2 h[14]	0	64
4	MeOH (0.95 equiv.), NaH (2 equiv.), THF (0.1 M), r.t., 2 h[15]	0	56
5	NaH (2 equiv.), DMSO (0.25 M), 0 °C to r.t., 2 h[16]	0	48
6[c]	MeMgBr (2 equiv.), HMPA (0.25 M), 0 °C to r.t., 2 h[17]	25 (21)	66
7	MeMgBr (2 equiv.), DMPU (0.25 M), 0 °C to r.t., 2 h	7	29
8	MeMgBr (2 equiv.), THF (0.25 M), 0 °C to r.t., 2 h	10	5

[a] Unless otherwise stated, reactions performed in 0.05 mmol scale, using **1a** (1.0 equiv.), **2h** (1.5 equiv.), $[\mathbf{1a}]_0$ = 0.5 M; irradiation time, 24 h. In all cases, formation of **Int.5** was observed as being >90% via ^{1}H-NMR using mesitylene as internal standard. When needed, solvent exchange was performed using a standard Schlenk line. Conditions inspired to previous works referenced in each condition. [b] Unless otherwise stated, ^{1}H-NMR yield using phenanthrene as internal standard. [c] Reaction performed on a 0.2 mmol scale. In parenthesis, isolated yield. Section 4.2 for procedure.

Use of α-phenyl substituted vinyl sulfonium **2i**

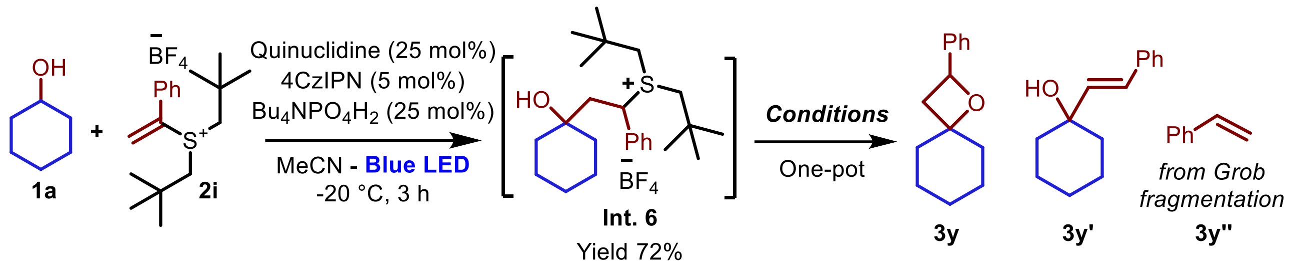

Entry[a]	Conditions	Yield of 3y (%)[b]	Yield of 3y'(%)[b]	Yield of 3y'' (%)[b]
1	^{t}BuOK (2 equiv.), MeCN (0.5 M), 60 °C, 24 h	0	60	0
2	^{t}BuOK (2 equiv.), DCM (0.25 M), r.t., 2 h	0	37	8
3	^{t}BuOK (2 equiv.), DMSO (0.25 M), r.t., 2 h	6	41	traces
4	NaH (2 equiv.), THF (0.17 M), r.t., 2 h	4	42	5
5	MeMgBr (2 equiv.), HMPA (0.25 M), 0 °C to r.t., 2 h[17]	17	0	30
6[c]	MeMgBr (2 equiv.), DMPU (0.25 M), -20 °C, 2 h	22 (24)	0	30

[a] Unless otherwise stated, reactions performed in 0.05 mmol scale, using **1a** (1.0 equiv.), **2i** (1.5 equiv.), $[\mathbf{1a}]_0$ = 0.5 M.; irradiation time, 3 h, -20 °C, see experimental for experiment procedure. In all cases, formation of **Int.6** was observed as being 72% via ^{1}H-NMR using mesitylene as internal standard. [b] Unless otherwise stated, ^{1}H-NMR yield using phenanthrene as internal standard. [c] Reaction performed on a 0.2 mmol scale, in parenthesis, yield of isolated material after chromatographic purification. Section 4.2 for experimental procedure.

4. General procedures & product characterization

General procedure A

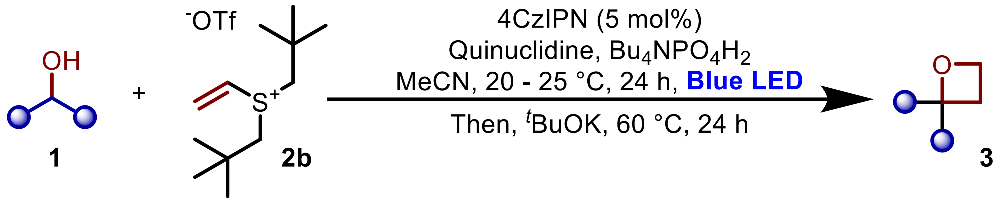

Alcohol **1** (1.0 equiv.; 0.2 mmol), dineopentyl(vinyl)sulfonium triflate **2b** (1.5 equiv.; 0.3 mmol; 105 mg), 4CzIPN (0.05 equiv.; 0.01 mmol; 7.9 mg), quinuclidine (0.25 equiv.; 0.05 mmol; 5.6 mg), and tetra-*n*-butylammonium phosphate (0.25 equiv.; 0.05 mmol; 17.0 mg) were introduced into a Schlenk tube. The atmosphere was exchanged to argon and degassed dry MeCN (0.4 mL, 0.5 M, previously degassed through 10 min argon sparging) was introduced through a syringe. The vessel was sealed and placed in a glass-wall water bath where a fan was blowing air to keep the water temperature between 20 - 25 °C. See Fig. S2 for visual details of the reaction setup. The reaction was irradiated through the glass wall with blue light (Kessil lamp A160WE Tuna Blue Saltwater LED Light 40 W) for 24 h under moderate stirring (500 rpm). The vessel was then removed from the water bath and ^{t}BuOK (2.0 equiv.; 0.4 mmol; 44.9 mg) was introduced into the vessel. The mixture was heated to 60 °C without irradiation for 24 h under vigorous stirring (700 rpm). The crude was subjected to chromatographic purification on silica gel to afford final compounds.

General Procedure B

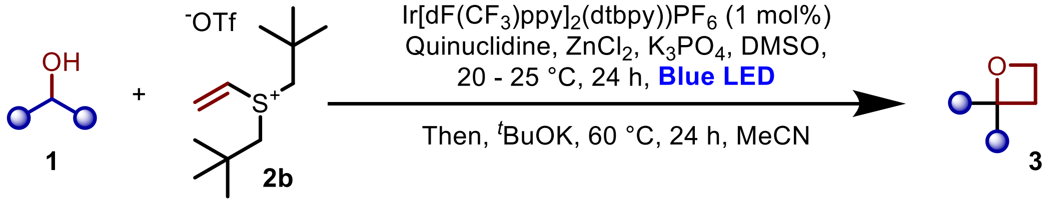

Alcohol **1** (1.0 equiv.; 0.2 mmol), dineopentyl(vinyl)sulfonium triflate **2b** (1.5 equiv.; 0.3 mmol; 105 mg), Ir[dF(CF_3)ppy]$_2$(dtbpy))PF_6 (0.01 equiv.; 0.002 mmol; 2.2 mg), quinuclidine (0.3 equiv.; 0.06 mmol; 6.7 mg) and potassium phosphate (1.5 equiv.; 0.3 mmol; 63.7 mg), were introduced into a Schlenk tube. In an oven-dried vial, $ZnCl_2$ (2.7 mmol; 367.2 mg) was added to 6 mL of dry DMSO. The solution was degassed through 10 min argon sparging and sonicated until complete solubilization of $ZnCl_2$. Then, 1 mL of this solution (containing 2.25 equiv.; 61.2 mg; 0.45 mmol of $ZnCl_2$) was introduced through a syringe into the Schlenk tube. The vessel was sealed and placed in a glass-wall water bath where a fan was blowing air to keep the water temperature between 20 - 25 °C. See Fig. S2 for visual details of the reaction setup. The reaction was irradiated through the glass wall with blue light (Kessil lamp A160WE Tuna Blue Saltwater LED Light 40W) for 24 h under moderate stirring (500 rpm). To remove the $ZnCl_2$, a filtration step with alumina is described as follows.

The vessel was removed from the water bath and basic alumina powder (3.5 g) was added, followed by DCM (10 mL). The suspension was stirred for 1 minute then, using a 5 mL syringe filled with cotton, the aluminium oxide powder was filtered. The syringe with alumina and the Schlenk walls were washed with a 9:1 DCM:MeOH solution (8 mL). The filtrate was collected and the volatiles were removed. The residue was then transferred quantitatively into a Schlenk tube using 2 mL of DCM. The Schenk was placed into a water bath (70°C) and nitrogen was blown to remove DCM. The remaining DMSO was removed by placing the tube under high vacuum ($5 \cdot 10^{-2}$ mbar) at 70 °C. When the distillation was complete, the tube was left under high vacuum at 70 °C for 5 min to ensure complete distillation of DMSO. Finally, the atmosphere in the tube was exchanged to argon and the crude was submitted to the next step.

Dry MeCN (1 mL) was introduced through a syringe. ^{t}BuOK (2 equiv.; 0.4 mmol; 44.9 mg) was added and the mixture was heated to 60 °C for 24 h under vigorous stirring. Volatiles were evaporated under reduced pressure, and the residue was subjected to chromatographic purification on silica gel to afford final compounds.

4.1. Reaction set-up for irradiation of mixtures with blue LEDs

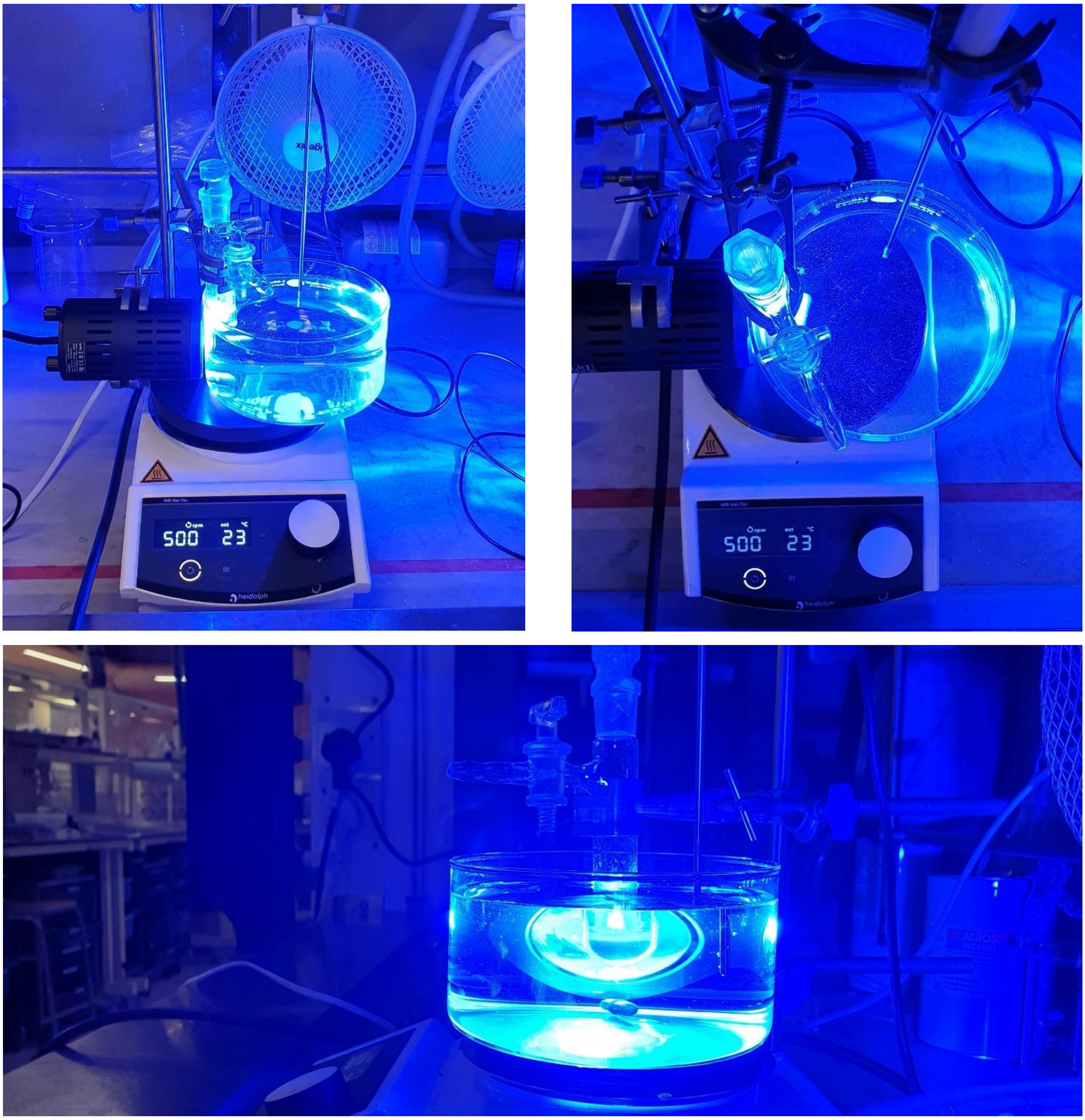

Fig. S2: Visual details of the photochemical reaction set-up: front, top, and lateral views.

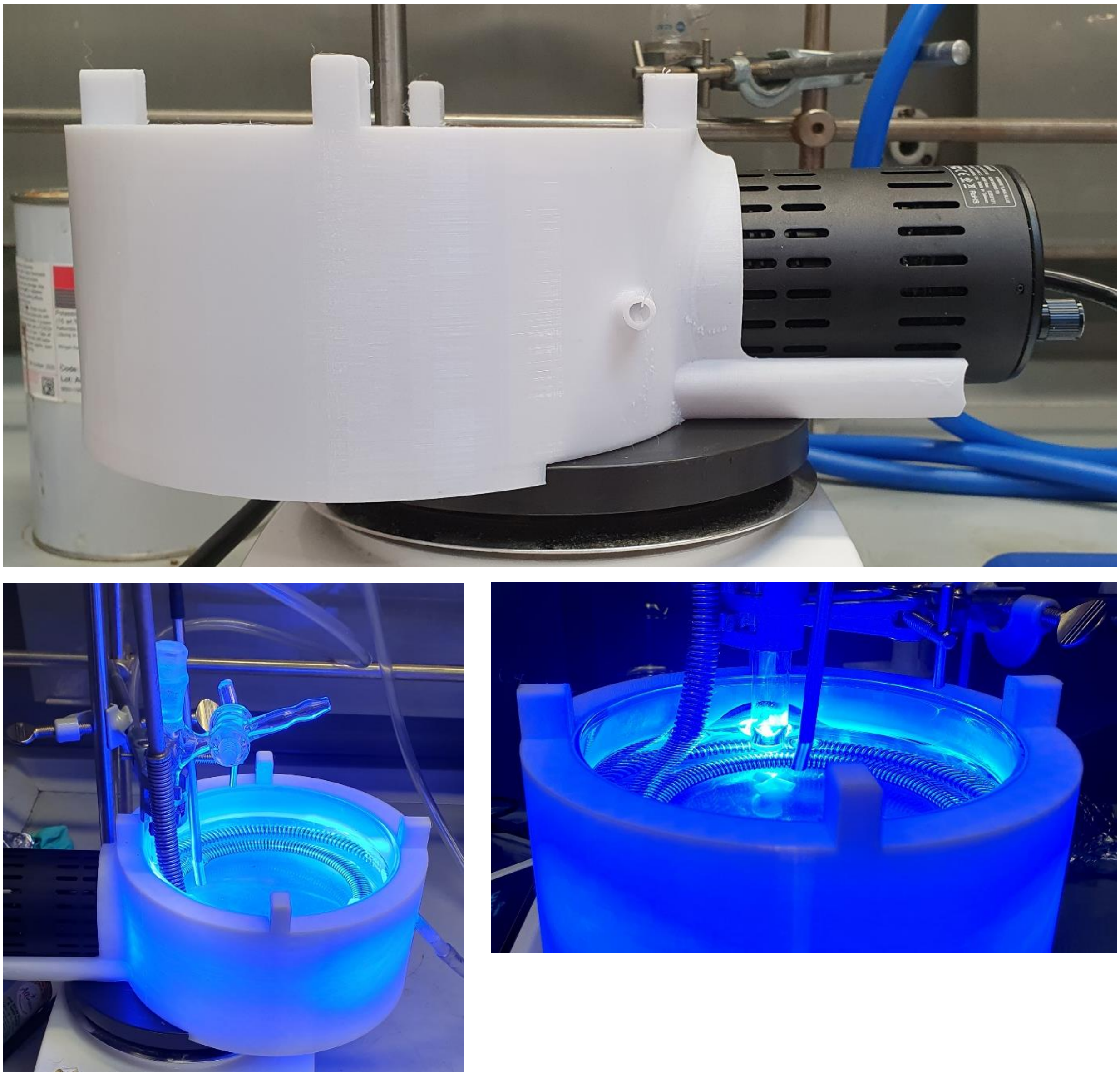

Fig S3: Visual details of the photochemical reaction set-up for irradiation below 0 °C. The 3D printed part encloses a laboratory standard crystallisation basin which is filled with acetone and cooled down to -20 °C by a chiller. Top view shows an attachment for nitrogen gas which ensure no water condensation on the wall facing the Kessil lamp.

4.2. Reaction products and characterization

1-oxaspiro[3.5]nonane (**3a**)

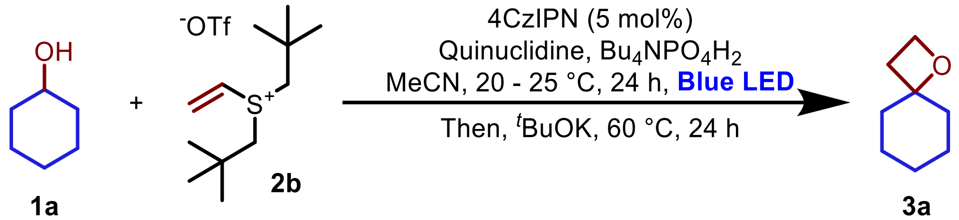

Prepared following general procedure A, using cyclohexanol **1a** (1.0 equiv.; 0.2 mmol; 21.1 µL), dineopentyl(vinyl)sulfonium triflate **2b** (1.5 equiv.; 0.3 mmol; 105 mg), 4CzIPN (0.05 equiv.; 0.01 mmol; 7.9 mg), tetra-*n*-butylammonium dihydrogen phosphate (0.25 equiv.; 0.05 mmol; 17.0 mg), quinuclidine (0.10 equiv.; 0.02 mmol; 2.2 mg) and ^{t}BuOK (2.0 equiv.; 0.4 mmol; 44.9 mg). Due to high volatility of compound **3a**, the NMR yield was measured (99%) using CH_2Br_2 as internal standard. The crude residue was purified by flash column chromatography (SiO_2; gradient 99:1 to 97:3 pentane:THF; eluent removed using a rotary evaporator, maintaining the water bath below 25 °C and vacuum pressure 450 – 80 mbar) to afford compound **3a** (18.7 mg; 74%) as a transparent oil. **R$_f$** (97:3 pentane:THF) 0.4; **IR** (film) ν_{max}/cm^{-1}:2939, 2876, 2855, 1446, 1370, 1337, 1282, 1246, 1222, 1143, 1058, 993, 962, 919, 890, 846, 656, 476, 447, 415; **^{1}H NMR** ($CDCl_3$, 400 MHz) δ (ppm): 4.49 (t, *J* = 7.8 Hz, 2H), 2.31 (t, *J* = 7.8 Hz, 2H), 1.88 – 1.76 (m, 2H), 1.74 – 1.58 (m, 4H), 1.42 – 1.24 (m, 4H); **^{13}C NMR** ($CDCl_3$, 126 MHz) δ (ppm): 86.7, 64.9, 38.8, 32.5, 25.2, 22.6; **HRMS** (ESI-TOF) mass calculated for $[2M+Na]^+$ ($C_{16}H_{28}O_2Na^+$) expected *m/z* 275.1982; found *m/z* 275.1989.

1,7-dioxaspiro[3.5]nonane (**3b**)

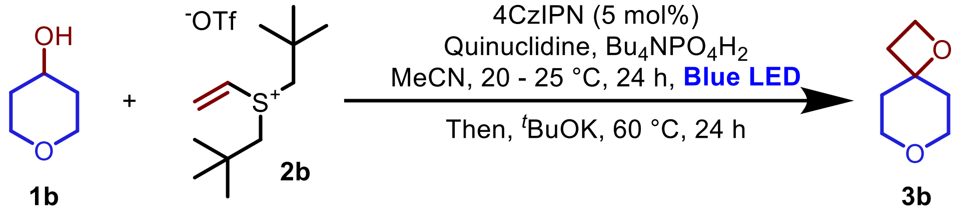

Prepared following General Procedure A, using 4-hydroxytetrahydropyran **1b** (1.0 equiv.; 0.2 mmol; 19.1 µL), dineopentyl(vinyl)sulfonium triflate **2b** (1.5 equiv.; 0.3 mmol; 105 mg), 4CzIPN (0.05 equiv.; 0.01 mmol; 7.9 mg), tetra-*n*-butylammonium dihydrogen phosphate (0.25 equiv.; 0.05 mmol; 17.0 mg), quinuclidine (0.25 equiv.; 0.05 mmol; 5.6 mg) and ^{t}BuOK (2.0 equiv.; 0.4 mmol; 44.9 mg). Due to high volatility of compound **3b**, the NMR yield was measured (73%) using CH_2Br_2 as internal standard. The crude residue was purified by flash column chromatography (SiO_2; 100% DCM to gradient 60:40 to 40:60 pentane:Et_2O; eluent removed using a rotary evaporator, maintaining the water bath below 25 °C and vacuum pressure 450 – 80 mbar) to afford compound **3b** (15.1 mg; 59%) as a pale yellow oil. **R$_f$** (60:40 pentane:Et_2O) 0.25; **IR** (film) ν_{max}/cm^{-1}:2955, 2924, 2858, 1454, 1389, 1363, 1335, 1313, 1260, 1220, 1155, 1121, 1101, 1028, 994, 970, 949, 843, 749, 619; **^{1}H NMR**

($CDCl_3$, 500 MHz) δ (ppm): 4.54 (t, *J* = 7.8 Hz, 2H), 3.77 (ddd, *J* = 11.4, 7.5, 3.8 Hz, 2H), 3.56 (ddd, *J* = 11.2, 6.6, 3.9 Hz, 2H), 2.41 (t, *J* = 7.8 Hz, 2H), 1.96 – 1.83 (m, 4H); **^{13}C NMR** ($CDCl_3$, 126 MHz) δ (ppm): 83.3, 65.0, 64.2, 39.0, 32.6; **HRMS** (ESI-TOF) mass calculated for $[M+Na]^+$ ($C_7H_{12}O_2Na^+$) expected *m/z* 151.0730; found *m/z* 151.0735.

1,8,11-trioxadispiro[3.2.4^7.2^4]tridecane (**3c**)

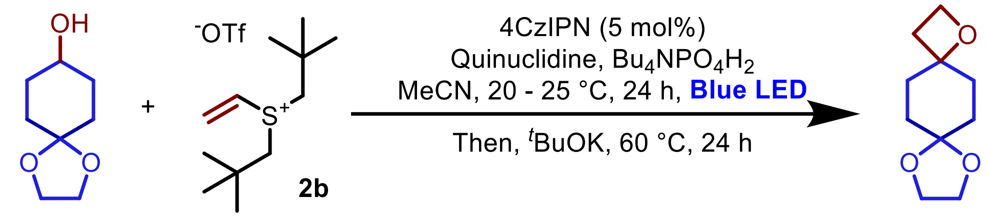

Prepared following general procedure A, using ethyl 1,4-dioxaspiro[4.5]decan-8-ol **1c** (1.0 equiv.; 0.2 mmol; 31.6 mg), dineopentyl(vinyl)sulfonium triflate **2b** (1.5 equiv.; 0.3 mmol; 105 mg), 4CzIPN (0.05 equiv.; 0.01 mmol; 7.9 mg), tetra-*n*-butylammonium dihydrogen phosphate (0.25 equiv.; 0.05 mmol; 17.0 mg), quinuclidine (0.25 equiv.; 0.05 mmol; 5.6 mg) and ^{t}BuOK (2.0 equiv.; 0.4 mmol; 44.9 mg) at 60 °C for 24 h. The crude residue was purified by flash column chromatography (SiO_2; gradient 60:40 to 50:50 pentane:Et_2O; eluent removed using a rotary evaporator, maintaining the water bath below 25 °C and vacuum pressure 450 – 80 mbar) to afford compound **3c** (34.6 mg; 94%) transparent oil. **R_f** (1:1 pentane:Et_2O) 0.5; **IR** (film) ν_{max}/cm^{-1}: 2935, 2877, 1442, 1375, 1329, 1249, 1153, 1092, 1034, 1000, 954, 921, 865, 770, 667, 540, 475, 432; **^{1}H NMR** ($CDCl_3$, 500 MHz) δ (ppm): 4.49 (t, *J* = 7.8 Hz, 2H), 3.98 – 3.87 (m, *J* = 3.5 Hz, 4H), 2.37 (t, *J* = 7.8 Hz, 2H), 2.06 – 1.97 (m, 2H), 1.90 – 1.82 (m, 2H), 1.81 – 1.73 (m, 2H), 1.61 – 1.53 (m, 2H); **^{13}C NMR** ($CDCl_3$, 126 MHz) δ (ppm): 108.1, 85.13, 64.9, 64.4, 64.4, 35.6, 31.9, 30.6; **HRMS** (ESI-TOF) mass calculated for $[M+Na]^+$ ($C_{10}H_{16}O_3Na^+$) expected *m/z* 207.0992; found *m/z* 207.0989.

1-oxa-7-thiaspiro[3.5]nonane 7,7-dioxide (**3d**)

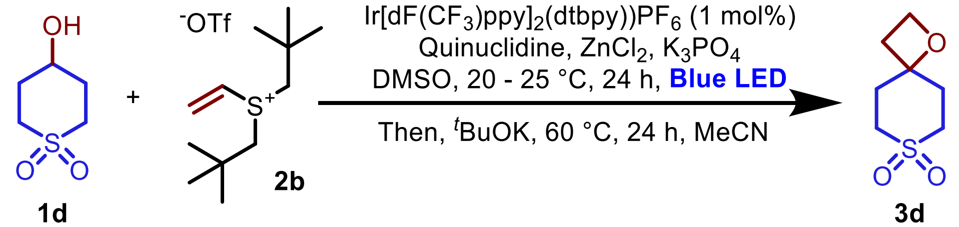

Prepared following general procedure B, using tetrahydro-2*H*-thiopyran-4-ol 1,1-dioxide **1d** (1.0 equiv.; 0.2 mmol; 31.6 mg), dineopentyl(vinyl)sulfonium triflate **2b** (1.5 equiv.; 0.3 mmol; 105 mg), Ir[dF(CF_3)ppy]$_2$(dtbpy))PF_6 (0.01 equiv.; 0.002 mmol; 2.2 mg), quinuclidine (0.3 equiv.; 0.06 mmol; 6.7 mg), potassium phosphate (1.5 equiv.; 0.3 mmol; 63.7 mg) and zinc chloride (2.25 equiv.; 0.45 mmol; 61.3 mg). After 24 h, ^{t}BuOK (2.0 equiv.; 0.4 mmol; 44.9 mg) was added and the mixture heated at 60 °C for 24 h. The crude residue was purified by flash column chromatography (SiO_2;

90:10 to 80:20 DCM:EtOAc) to afford compound **3d** (20.9 mg; 59%) as a yellow solid. **R$_f$** (80:20 DCM:EtOAc) 0.3; **M.P.** = 99 – 100 °C; **IR** (film) ν_{max}/cm^{-1}: 2924, 1425, 1313, 1286, 1228, 1187, 1130, 1090, 1059, 980, 952, 880, 943, 703; **^{1}H NMR** (500 MHz, $CDCl_3$) δ (ppm): 4.56 (t, *J* =7.8 Hz, 2H), 3.31 – 3.25 (m, 2H), 2.91 – 2.86 (m, 2H), 2.54 – 2.45 (m, 4H), 2.34 – 2.28 (m, 2H); **^{13}C NMR** (126 MHz, $CDCl_3$) δ (ppm): 81.3 65.1, 46.8, 35.7, 31.8; **HRMS** (ESI-TOF) mass calculated for $[M+Na]^+$ ($C_7H_{12}SO_3Na^+$) expected *m/z* 199.0399; found *m/z* 199.0398.

phenyl(1-oxa-7-azaspiro[3.5]nonan-7-yl)methanone (**3e**)

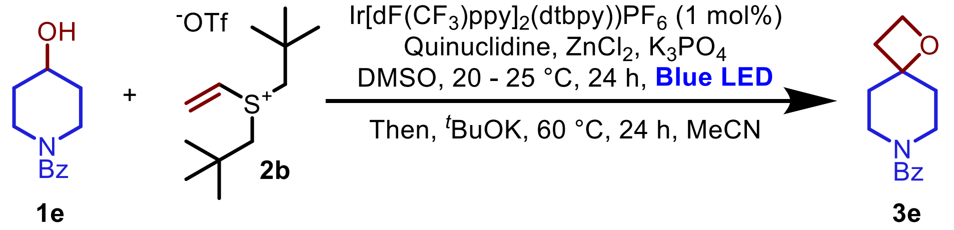

Prepared following general procedure B, using (4-hydroxypiperidin-1-yl)(phenyl)methanone **1e** (1.0 equiv.; 0.2 mmol; 41.0 mg), dineopentyl(vinyl)sulfonium triflate **2b** (1.5 equiv.; 0.3 mmol; 105 mg), Ir[dF(CF_3)ppy]$_2$(dtbpy))PF_6 (0.01 equiv.; 0.002 mmol; 2.2 mg), quinuclidine (0.3 equiv.; 0.06 mmol; 6.7 mg), potassium phosphate (1.5 equiv.; 0.3 mmol; 63.7 mg) and zinc chloride (2.25 equiv.; 0.45 mmol; 61.3 mg). After 24 h, ^{t}BuOK (2.0 equiv.; 0.4 mmol; 44.9 mg) was added and the mixture heated at 60 °C for 24 h. The crude residue was purified by flash column chromatography (SiO_2; 80:20 to 30:70 DCM:Et_2O) to afford compound **3e** (34.4 mg; 74%) transparent oil. **R$_f$** (1:1 pentane:Et_2O) 0.6; **IR** (film) ν_{max}/cm^{-1}: 3436, 2961, 2926, 2852, 1703, 1682, 1455, 1394, 1367, 1455, 1394, 1367, 1256, 1174, 1047, 988, 922, 862, 775; **^{1}H NMR** ($CDCl_3$, 500 MHz) δ (ppm): 7.42 – 7.36 (m, 5H), 4.59 – 4.50 (m, 2H), 3.95 (bs, 1H), 3.63 – 3.28 (m, 3H), 2.50 – 2.32 (m, 2H), 2.15 – 1.62 (m, 4H); **^{13}C NMR** ($CDCl_3$, 126 MHz) rotameric mixture, resonances for minor rotamer are enclosed in parenthesis () δ (ppm): 170.5, 136.2, 129.7, 128.6, 126.9, 83.8, 65.0, 43.8, 38.4, (37.5), 32.1; **HRMS** (ESI-TOF) mass calculated for $[M+H]^+$ ($C_{14}H_{18}NO_2^+$) expected *m/z* 232.1332; found *m/z* 232.1327.

ethyl 1-oxaspiro[3.5]nonane-7-carboxylate (**3f**)

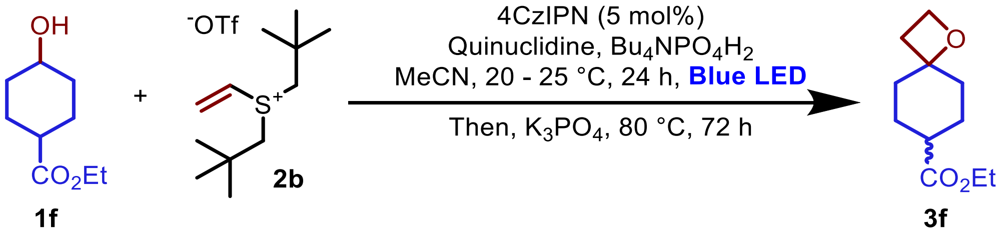

Prepared following general procedure A, using ethyl 4-hydroxycyclohexane-1-carboxylate **1f** (1.0 equiv.; 0.2 mmol; 34.4 mg), dineopentyl(vinyl)sulfonium triflate **2b** (1.5 equiv.; 0.3 mmol; 105 mg), 4CzIPN (0.05 equiv.; 0.01 mmol; 7.9 mg), tetra-*n*-butylammonium dihydrogen phosphate (0.25

equiv.; 0.05 mmol; 17.0 mg), quinuclidine (0.1 equiv.; 0.05 mmol; 2.2 mg). Then K_3PO_4 (2.0 equiv.; 0.4 mmol; 84.8 mg) was added and the reaction left under stirring at 80 °C for 72 h. The crude residue was purified by flash column chromatography (SiO_2; 97.5:2.5 DCM:Et_2O then gradient 6:4 to 1:1 pentane:Et_2O) to afford compound **3f** (19.5 mg; 49%; d.r. = 3.8:1) as yellow oil. **R$_f$** (1:1 pentane:Et_2O) 0.6; **IR** (film) ν_{max}/cm^{-1}: 2935, 2877, 1720, 1445, 1377, 1350, 1306, 1185, 1136, 1069, 1040 ,992, 971, 919, 861, 656, 520; **^{1}H NMR** ($CDCl_3$, 500 MHz) δ (ppm): 4.49 (t, J = 7.8 Hz, 2H), 4.11 (qd, J = 7.1, 1.7 Hz, 2H), 2.36 (t, J = 7.8 Hz, 2H), 2.28 – 2.20 (m, 1H), 2.16 – 2.08 (m, 2H), 2.00 – 1.91 (m, 2H), 1.60 – 1.44 (m, 4H), 1.24 (t, J = 7.3 Hz, 3H); **^{13}C NMR** ($CDCl_3$, 126 MHz) δ (ppm): 175.5, 86.0, 65.0, 60.4, 41.6, 37.3, 31.9, 25.3, 14.4; **HRMS** (ESI-TOF) mass calculated for $[M+Na]^+$ ($C_{11}H_{18}O_3Na^+$) expected m/z 221.1148; found m/z 221.1153.

1-oxaspiro[3.11]pentadecane (3g)

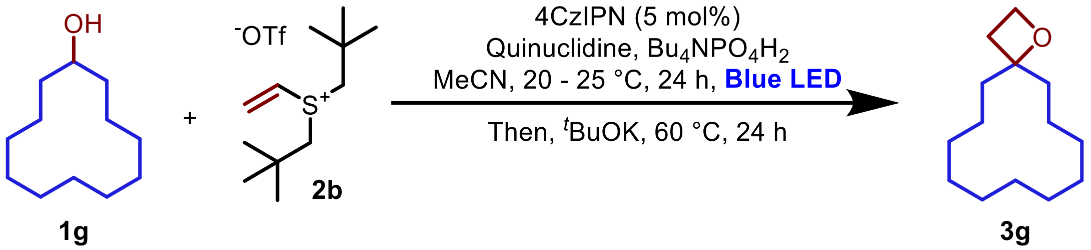

Prepared following general procedure A, using cyclododecanol **1g** (1.0 equiv.; 0.2 mmol; 36.9 mg), dineopentyl(vinyl)sulfonium triflate **2b** (1.5 equiv.; 0.3 mmol; 105 mg), 4CzIPN (0.05 equiv.; 0.01 mmol; 7.9 mg), tetra-*n*-butylammonium dihydrogen phosphate (0.25 equiv.; 0.05 mmol; 17.0 mg), quinuclidine (0.25 equiv.; 0.05 mmol; 5.6 mg) and ^{t}BuOK (2.0 equiv.; 0.4 mmol; 44.9 mg). The crude residue was purified by flash column chromatography (SiO_2; gradient 95:5 to 90:10 pentane:Et_2O) to afford compound **3g** (31.7 mg; 75%) as a white solid. **R$_f$** (95:5 pentane:Et_2O) 0.25; **M. P.** = 35-36 °C; **IR** (film) ν_{max}/cm^{-1}: 2936, 2865, 2850, 1470, 1445, 1347, 1285, 1244, 1055, 981, 960, 863, 724, 474; **^{1}H NMR** ($CDCl_3$, 400 MHz) δ (ppm): 4.49 (t, J = 7.8 Hz, 2H), 2.31 (t, J = 7.8 Hz, 2H), 1.94 – 1.82 (m, 2H), 1.79 – 1.66 (m, 2H), 1.44 – 1.22 (m, 18H); **^{13}C NMR** ($CDCl_3$, 101 MHz) δ (ppm): 89.4, 65.0, 34.9, 32.6, 26.4, 26.0, 22.7, 22.4, 18.8; **HRMS** (ESI-TOF) mass calculated for $[M+H]^+$ ($C_{14}H_{27}O^+$) expected m/z 211.2056; found m/z 211.2056.

1-oxaspiro[3.3]heptane (3h)

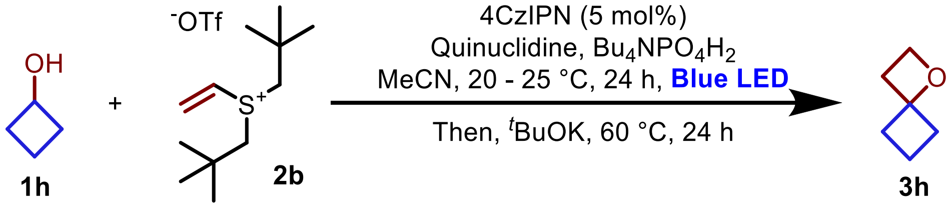

Prepared following general procedure A, using cyclobutanol **1h** (1.0 equiv.; 0.2 mmol; 15.7 µL), dineopentyl(vinyl)sulfonium triflate **2b** (1.5 equiv.; 0.3 mmol; 105 mg), 4CzIPN (0.05 equiv.; 0.01

mmol; 7.9 mg), tetra-*n*-butylammonium dihydrogen phosphate (0.25 equiv.; 0.05 mmol; 17.0 mg), quinuclidine (0.25 equiv.; 0.05 mmol; 5.6 mg) and ^{t}BuOK (2.0 equiv.; 0.4 mmol; 44.9 mg). Due to high volatility of compound **3h**, the NMR yield was measured (76%) using CH_2Br_2 as internal standard. The crude residue was purified by flash column chromatography (SiO_2; gradient 95:5 to 90:10 pentane:Et_2O; eluent removed using a rotary evaporator, maintaining the water bath below 25 °C and vacuum pressure 450 – 80 mbar) to afford compound **3h** (9.2 mg; 47%) as a transparent oil. **R$_f$** (95:5 pentane:Et_2O) 0.3; **IR** (film) ν_{max}/cm^{-1}:2926, 2854, 1451, 1327, 1119, 748, 725; **^{1}H NMR** ($CDCl_3$, 500 MHz) δ (ppm): 4.49 (t, *J* = 7.5 Hz, 2H), 2.68 (t, *J* = 7.5 Hz, 2H), 2.28 – 2.19 (m, 4H), 1.68 – 1.59 (m, 1H), 1.49 – 1.39 (m, 1H); **^{13}C NMR** ($CDCl_3$, 126 MHz) δ (ppm): 87.8, 65.6, 38.4, 34.3, 11.0; **HRMS** (ESI-TOF) mass calculated for $[2M+Na]^+$ ($C_{12}H_{20}O_2Na^+$) expected *m/z* 219.1356; found *m/z* 219.1358.

<u>*tert*-butyl 1-oxa-8-azadispiro[3.1.36.14]decane-8-carboxylate (3i)</u>

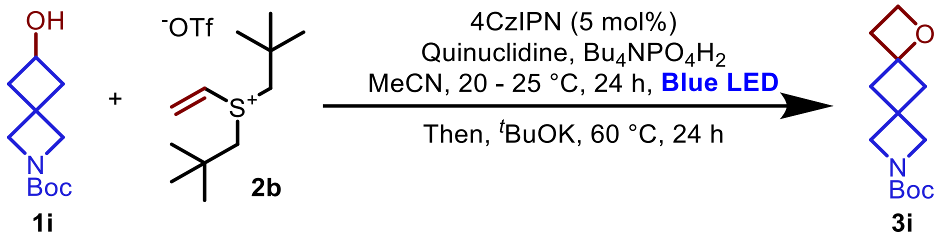

Prepared following general procedure A, using tert-butyl 6-hydroxy-2-azaspiro[3.3]heptane-2-carboxylate **1i** (1.0 equiv.; 0.2 mmol;), dineopentyl(vinyl)sulfonium triflate **2b** (1.5 equiv.; 0.3 mmol; 105 mg), 4CzIPN (0.05 equiv.; 0.01 mmol; 7.9 mg), tetra-*n*-butylammonium dihydrogen phosphate (0.25 equiv.; 0.05 mmol; 17.0 mg), quinuclidine (0.25 equiv.; 0.05 mmol; 5.6 mg) and ^{t}BuOK (2.0 equiv.; 0.4 mmol; 44.9 mg). The crude residue was purified by flash column chromatography (SiO_2; gradient 85:15 to 70:30 DCM:EtOAc) to afford compound **3i** (17.3 mg, 37%) as a yellow oil. **R$_f$** (70:30 DCM:EtOAc) 0.4; **IR** (film) ν_{max}/cm^{-1}: 3415, 2962, 2925, 2854, 1703, 1455, 1394, 1367, 1256, 1171, 1047, 988, 922, 861; **^{1}H NMR** ($CDCl_3$, 500 MHz) δ (ppm): 4.50 (t, *J* = 7.5 Hz, 2H), 3.86 (d, *J* = 4.3 Hz, 4H), 2.64 (t, *J* = 7.5 Hz, 2H), 2.50 – 2.46 (m, 2H), 2.44 – 2.40 (m, 2H), 1.42 (s, 9H); **^{13}C NMR** ($CDCl_3$, 126 MHz) δ (ppm): 156.3, 83.5, 79.5, 65.9, 59.9, 48.7, 33.9, 28.9, 28.5; **HRMS** (ESI-TOF) mass calculated for $[M+Na]^+$ ($C_{13}H_{21}NO_3Na^+$) expected *m/z* 262.1414; found *m/z* 262.1414.

(1-oxa-8-azadispiro[3.1.36.14]decan-8-yl)(phenyl)methanone (**3j**)

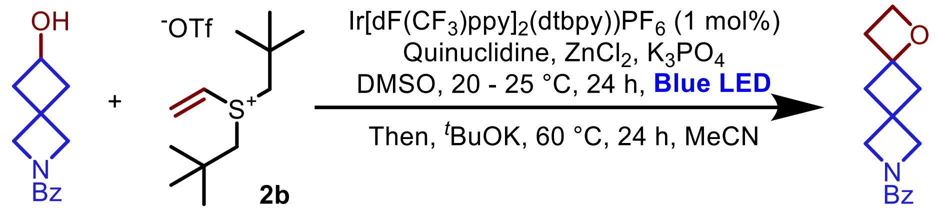

1j **3j**

Prepared following general procedure B, using (6-hydroxy-2-azaspiro[3.3]heptan-2-yl)(phenyl)methanone **1j** (1.0 equiv.; 0.1 mmol; 21.7 mg), dineopentyl(vinyl)sulfonium triflate **2b** (1.5 equiv.; 0.15 mmol; 52.6 mg),), Ir[dF(CF_3)ppy]$_2$(dtbpy))PF_6 (0.01 equiv.; 0.001 mmol; 1.1 mg), quinuclidine (0.3 equiv.; 0.03 mmol; 3.3 mg), potassium phosphate (1.5 equiv.; 0.15 mmol; 31.8 mg) and zinc chloride (2.25 equiv.; 0.23 mmol; 30.6 mg) and ^{t}BuOK (2.0 equiv.; 0.2 mmol; 22.4 mg). The crude residue was purified by flash column chromatography (SiO_2; gradient 50:50 to 0:100 DCM:EtOAc) to afford compound **3j** (11.4 mg; 47%) as a pale yellow oil. **R$_f$** (EtOAc) 0.25; **IR** (film) ν_{max}/cm^{-1}: 3472, 2924, 2873, 1630, 1575, 1450, 1416, 1314, 1287, 979, 952, 795, 713; **^{1}H NMR** ($CDCl_3$, 500 MHz) δ (ppm): 7.65 – 7.57 (m, 2H), 7.48 – 7.35 (m, 3H), 4.55 – 4.45 (m, 2H), 4.29 – 4.09 (m, 4H), 2.73 – 2.59 (m, 2H), 2.59 – 2.39 (m, 4H); **^{13}C NMR** (126 MHz, $CDCl_3$) rotameric mixture, resonances for minor rotamer are enclosed in parenthesis () δ(ppm): 170.3, (133.4) 133.3, 128.5, 128.0, 124.96, 83.5, (83.2), 66.1, (65.9), (65.7) 64.4, 60.9, (59.7), (48.9), 48.8, (33.8), 33.7, 29.7; **HRMS** (ESI-TOF) mass calculated for $[M+Na]^+$ ($C_{12}H_{17}NO_2Na^+$) expected *m/z* 266.1151; found *m/z* 266.1152.

(1-oxa-9-azadispiro[3.1.5^6.1^4]dodecan-9-yl)(phenyl)methanone (**3k**)

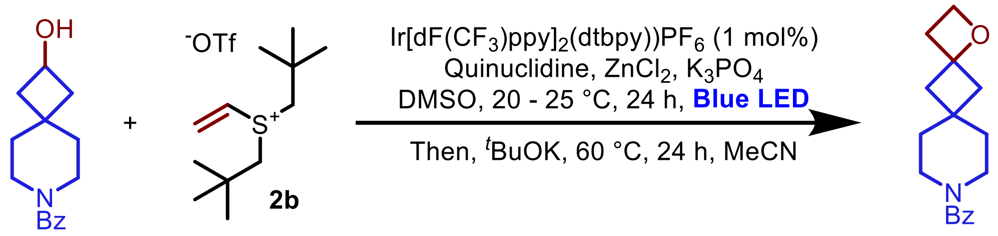

1k **3k**

Prepared following general procedure B, using (2-hydroxy-7-azaspiro[3.5]nonan-7-yl)(phenyl)methanone **1k** (1.0 equiv.; 0.2 mmol; 49.0 mg), dineopentyl(vinyl)sulfonium triflate **2b** (1.5 equiv.; 0.3 mmol; 105 mg), Ir[dF(CF_3)ppy]$_2$(dtbpy))PF_6 (0.01 equiv.; 0.002 mmol; 2.2 mg), quinuclidine (0.3 equiv.; 0.06 mmol; 6.7 mg), potassium phosphate (1.5 equiv.; 0.3 mmol; 63.7 mg) and zinc chloride (2.25 equiv.; 0.45 mmol; 61.3 mg) and ^{t}BuOK (2.0 equiv.; 0.4 mmol; 44.9 mg). The crude residue was purified by flash column chromatography (SiO_2; gradient 50:50 to 30:70 hexane:EtOAc) to afford compound **3k** (36.8 mg; 68%) as a pale yellow oil. **R$_f$** (50:50 hexane:EtOAc) 0.25; **IR** (film) ν_{max}/cm^{-1}: 2914, 1600, 1433, 1275, 1248, 1061, 981, 952, 788, 709; **^{1}H NMR** ($CDCl_3$, 400 MHz) δ (ppm): 7.41 – 7.32 (m, 5H), 4.52 (t, J = 7.5 Hz, 2H), 3.65 (br. s, 2H), 3.27 (br. s, 2H),

2.67 (br. s, 2H), 2.34 – 2.04 (m, 4H), 1.70 – 1.34 (m, 4H); **^{13}C NMR** ($CDCl_3$, 126 MHz) rotameric mixture, resonances for minor rotamer are enclosed in parenthesis () δ (ppm): 170.5, 136.4, 129.6, 128.6, 126.9, 84.8, 66.2, 47.9 , (45.2), 39.7, (39.2), (38.2), (37.4), 36.5, 35.8, 30.1; **^{1}H NMR** (DMSO-*d*$_6$, 400 MHz, 80 °C) δ (ppm): 7.46 – 7.38 (m, 3H), 7.38 – 7.30 (m, 2H), 4.38 (t, *J* = 7.5 Hz, 2H), 3.47 – 3.24 (m, 4H), 2.63 (t, *J* = 7.5 Hz, 2H), 2.28 – 2.19 (m, 2H), 2.06 – 1.97 (m, 2H), 1.53 – 1.40 (m, 4H); **^{13}C NMR** (DMSO-*d*$_6$, 101 MHz, 80 °C) δ (ppm): 168.6, 136.2, 128.7, 127.8, 126.1, 83.7, 64.5, 46.9, 37.8, 35.9, 34.6, 29.4; **HRMS** (ESI-TOF) mass calculated for $[M+Na]^+$ ($C_{17}H_{21}NO_2Na^+$) expected *m/z* 294.1465; found *m/z* 294.1468.

spiro[bicyclo[2.2.1]heptane-2,2'-oxetane] (**3l**)

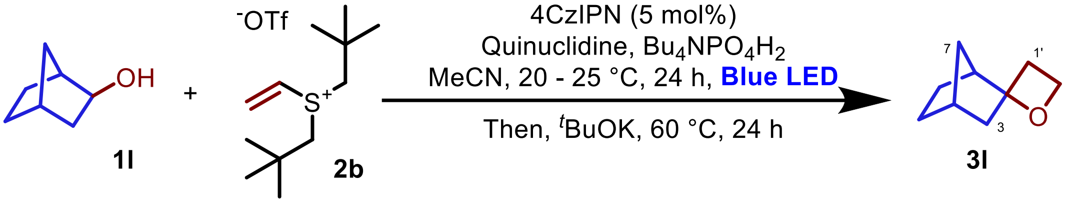

Prepared following general procedure A, using *exo*-Norborneol **1l** (1.0 equiv.; 0.2 mmol; 22.4 mg), dineopentyl(vinyl)sulfonium triflate **2b** (1.5 equiv.; 0.3 mmol; 105 mg), 4CzIPN (0.05 equiv.; 0.01 mmol; 7.9 mg), tetra-*n*-butylammonium dihydrogen phosphate (0.25 equiv.; 0.05 mmol; 17.0 mg), quinuclidine (0.25 equiv.; 0.05 mmol; 5.6 mg) and ^{t}BuOK (2.0 equiv.; 0.4 mmol; 44.9 mg). Due to high volatility of compound **3b**, the NMR yield was measured (92%) using CH_2Br_2 as internal standard. The crude residue was purified by flash column chromatography (SiO_2; 97.5:2.5 to 9:1 pentane:Et_2O; eluent removed using a rotary evaporator, maintaining the water bath below 25 °C and vacuum pressure 450 – 80 mbar) to afford compound **3l** (19.7 mg; 71%; >20:1 d.r.) transparent oil. The stereochemistry of the final compound was unambiguously inferred by NOESY analysis by detecting strong NOE interaction between $H_{1'}$ and both H_7 and $H_{3(equat.)}$. **R_f** (1:1 pentane:Et_2O) 0.6; **IR** (film) ν_{max}/cm^{-1}: 2949, 2872, 1704, 1449, 1333, 1311, 1236, 1180, 1135, 1100, 1061, 986, 970, 917, 883, 447; **^{1}H NMR** ($CDCl_3$, 500 MHz) δ (ppm): 4.42 – 4.36 (m, 2H), 2.56 – 2.42 (m, 2H), 2.41 (d, *J* = 4.4 Hz, 1H), 2.19 – 2.13 (m, 1H), 1.84 – 1.71 (m, 2H), 1.52 – 1.43 (m 2H), 1.36 (tt, *J* = 12.4, 4.1 Hz, 1H), 1.29 – 1.14 (m, 3H); **^{13}C NMR** ($CDCl_3$, 126 MHz) δ (ppm): 92.6, 64.4, 47.6, 46.9, 37.1, 37.0, 36.0, 28.9, 20.0 **HRMS** (ESI-TOF) mass calculated for $[M+H]^+$ ($C_9H_{15}O^+$) expected *m/z* 139.1117; found *m/z* 139.1113.

spiro[adamantane-2,2'-oxetane] (3m)

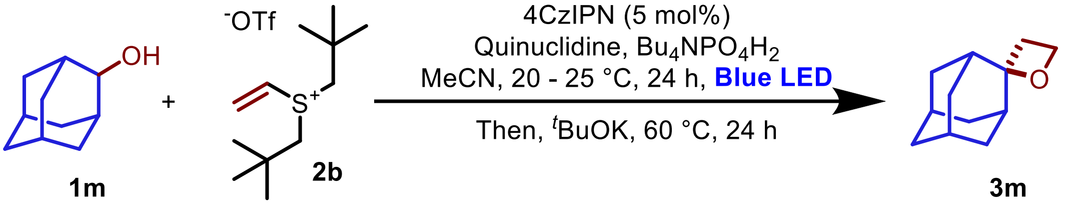

Prepared following general procedure A, using 2-adamantanol **1m** (1.0 equiv.; 0.2 mmol; 30.4 mg), dineopentyl(vinyl)sulfonium triflate **2b** (1.5 equiv.; 0.3 mmol; 105 mg), 4CzIPN (0.05 equiv.; 0.01 mmol; 7.9 mg), tetra-*n*-butylammonium dihydrogen phosphate (0.25 equiv.; 0.05 mmol; 17.0 mg), quinuclidine (0.25 equiv.; 0.05 mmol; 5.6 mg) and *ᵗ*BuOK (2.0 equiv.; 0.4 mmol; 44.9 mg). After the heating step, a minor amount of an unknown olefin was formed. To effectively removed it, the crude mixture was filtered through celite, eluted with DCM, and concentrated under reduced pressure. The mixture was dissolved in dry DCM (0.1 M, 2.0 mL), cooled down at 0 °C and *m*CPBA (70%) (4.0 equiv.; 0.8 mmol; 197 mg) was slowly added. The reaction mixture was then warmed up to room temperature – allowing the ice bath to melt – and left stirring (700 ppm) at room temperature until complete consumption of olefin monitored *via* ^{1}H-NMR analysis of a small reaction aliquot *(disappearance of multiplet at 5.70 – 5.61 ppm)*. Then, the mixture was diluted with DCM (20 mL), washed with saturated $NaHCO_3$ solution (10 mL), followed by water (15 mL) and brine (15 mL), dried over anhydrous $MgSO_4$. Finally, the solution was filtered and solvent removed under reduced pressure. The crude residue was purified by flash column chromatography (SiO_2; gradient 95:5 to 85:15 pentane:Et_2O) to afford compound **3m** (17.7 mg; 50%) as a white solid. **R_f** (90:10 pentane:Et_2O) 0.3; **M. P.** = 61-62 °C; **IR** (film) ν_{max}/cm^{-1}:2902, 2852, 1467, 1449, 1351, 1230, 1134, 1102, 1083, 1003, 981, 919, 825, 662, 488; **^{1}H NMR** ($CDCl_3$, 500 MHz) δ (ppm): 4.43 (t, *J* = 7.8 Hz, 2H), 2.37 (t, *J* = 7.8 Hz, 2H), 2.19 – 2.13 (m, 2H), 2.03 – 1.95 (m, 2H), 1.78 – 1.61 (m, 8H), 1.57 – 1.52 (m, 2H); **^{13}C NMR** ($CDCl_3$, 101 MHz) δ (ppm): 90.5, 64.1, 38.7, 36.9, 33.6, 32.6, 31.9, 26.8, 26.7; **HRMS** (ESI-TOF) mass calculated for $[M+NH_4]^+$ ($C_{12}H_{22}NO^+$) expected *m/z* 196.1696; found *m/z* 196.1688.

tert-butyl (1*R*,3*s*,5*S*)-8-azaspiro[bicyclo[3.2.1]octane-3,2'-oxetane]-8-carboxylate (3n)

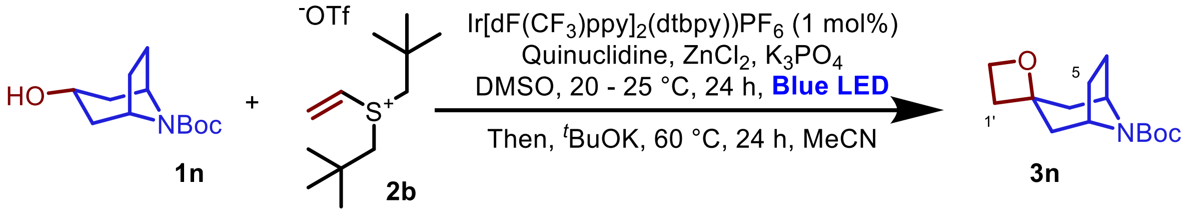

Prepared following general procedure B, using *tert*-butyl 3-endo-3-hydroxy-8-azabicyclo[3.2.1]octane-8-carboxylate1,1-dioxide **1n** (1.0 equiv.; 0.2 mmol; 45.5 mg), dineopentyl(vinyl)sulfonium triflate **2b** (1.5 equiv.; 0.3 mmol; 105 mg), $Ir[dF(CF_3)ppy]_2(dtbpy))PF_6$ (0.01 equiv.; 0.002 mmol; 2.2 mg), quinuclidine (0.3 equiv.; 0.06 mmol; 6.7 mg), potassium

phosphate (1.5 equiv.; 0.3 mmol; 63.7 mg) zinc chloride (2.25 equiv.; 0.45 mmol; 61.3 mg) and ^{t}BuOK (2.0 equiv.; 0.4 mmol; 44.9 mg). The crude residue was purified by flash column chromatography (SiO_2; 95:5 to 75:25 hexane:THF) to afford compound **3n** (39.6 mg; 78%; >20:1 d.r.) as a yellow oil. The stereochemistry of the final compound was elucidated by NOESY analysis at 80°C by total absence of NOE interaction between $H_{1'}$ and H_5. **R$_f$** (80:20 hexane:THF) 0.4; **IR** (film) ν_{max}/cm^{-1}: 2975, 2877, 1689, 1477, 1390, 1364, 1329, 1307, 1256, 1228, 1170, 1119, 1097, 1081, 1011, 986, 960, 879, 838, 796, 770, 572, 541, 497; **^{1}H NMR** (500 MHz, $CDCl_3$) δ (ppm): 4.45 (t, *J* = 7.4 Hz, 2H), 4.2 8 – 4.05 (m, 2H), 2.35 – 2.25 (m, 4H), 2.07 – 2.00 (m, 2H), 1.94 – 1.75 (m, 4H), 1.45 (s, 9H); **^{13}C NMR** (126 MHz, $CDCl_3$) rotameric mixture, resonances for minor rotamer are enclosed in parenthesis () δ(ppm): 153.5, 85.3, 79.3, 65.1, 53.5, (52.7), 44.0, (43.2), 34.6, 28.6, 27.7, (27.1); **HRMS** (ESI-TOF) mass calculated for $[M+Na]^+$ ($C_{14}H_{23}NO_3Na^+$) expected *m/z* 276.1570; found *m/z* 276.1568; **^{1}H NMR** (400 MHz, DMSO-d_6, 80°C) δ (ppm): 4.36 (t, *J* = 7.8 Hz, 2H), 4.11 – 4.01 (m, 2H), 2.29 (t, *J* = 7.8 Hz, 2H), 2.26 – 2.18 (m, 2H), 2.02 – 1.94 (m, 2H), 1.81 – 1.71 (m, 4H), 1.40 (s, 9H); **^{13}C NMR** (101 MHz, DMSO-d_6, 80°C) δ (ppm): 152.2, 83.7, 77.9, 63.7, 52.3, 42.5, 33.5, 27.8, 26.5.

2-heptyl-2-hexyloxetane (3o)

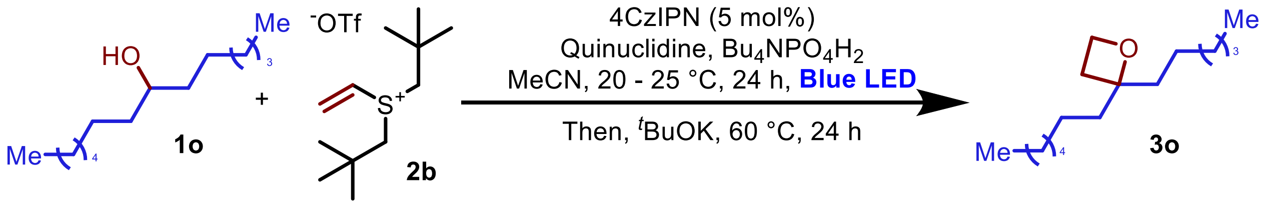

Prepared following general procedure A, using 7-tetradecanol **1o** (1.0 equiv.; 0.2 mmol; 42.9 mg), dineopentyl(vinyl)sulfonium triflate **2b** (1.5 equiv.; 0.3 mmol; 105 mg), 4CzIPN (0.05 equiv.; 0.01 mmol; 7.9 mg), tetra-*n*-butylammonium dihydrogen phosphate (0.25 equiv.; 0.05 mmol; 17.0 mg), quinuclidine (0.25 equiv.; 0.05 mmol; 5.6 mg) and ^{t}BuOK (2.0 equiv.; 0.4 mmol; 44.9 mg). The crude residue was purified by flash column chromatography (SiO_2; gradient 96:4 to 95:5 pentane:Et_2O) to afford compound **3o** (32.8 mg; 68%) as a transparent oil. **R$_f$** (95:5 pentane:Et_2O) 0.3; **IR** (film) ν_{max}/cm^{-1}: 2955, 2926, 2872, 2856, 1465, 1378, 1229, 1139, 964, 843, 725; **^{1}H NMR** ($CDCl_3$, 500 MHz) δ (ppm): 4.43 (t, *J* = 7.9 Hz, 2H), 2.33 (t, *J* = 7.9 Hz, 2H), 1.72 – 1.56 (m, 4H), 1.37 – 1.23 (m, 18H), 0.93 – 0.84 (m, 6H); **^{13}C NMR** ($CDCl_3$, 126 MHz) δ (ppm): 89.0, 64.8, 39.9, 32.1, 32.0, 30.7, 30.2, 29.9, 29.5, 23.3, 23.2, 22.8, 22.8, 14.2; **HRMS** (ESI-TOF) mass calculated for $[M+H]^+$ ($C_{16}H_{33}O^+$) expected *m/z* 241.2531; found *m/z* 241.2529.

2-methyl-2-phenethyloxetane (3p)

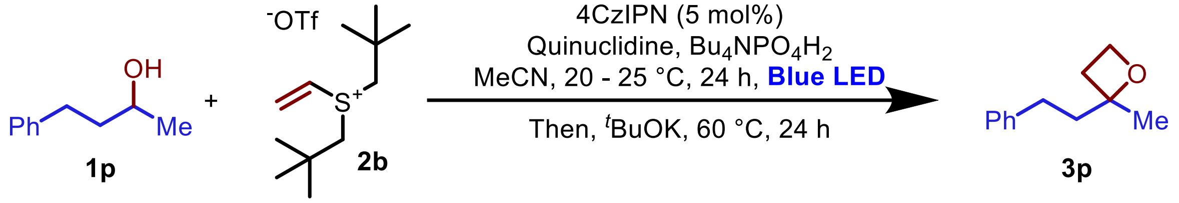

Prepared following general procedure A, using 4-phenyl-2-butanol **1p** (1.0 equiv.; 0.2 mmol; 31.0 μL), dineopentyl(vinyl)sulfonium triflate **2b** (1.5 equiv.; 0.3 mmol; 105 mg), 4CzIPN (0.05 equiv.; 0.01 mmol; 7.9 mg), tetra-*n*-butylammonium dihydrogen phosphate (0.25 equiv.; 0.05 mmol; 17.0 mg), quinuclidine (0.25 equiv.; 0.05 mmol; 5.6 mg) and ^{t}BuOK (2.0 equiv.; 0.4 mmol; 44.9 mg). The crude residue was purified by flash column chromatography (SiO_2; gradient 50:50 pentane:DCM to 95:5-85:15 pentane:Et_2O) to afford compound **3p** (14.6 mg; 41%) as a transparent oil. **R$_f$** (95:5 pentane:Et_2O) 0.35; **IR** (film) ν_{max}/cm^{-1}:3026, 2964, 2927, 1603, 1497, 1453, 1373, 1258, 1140, 1097, 1031, 962, 857, 750, 699, 568, 509; **^{1}H NMR** ($CDCl_3$, 500 MHz) δ (ppm): 7.32 – 7.27 (m, 2H), 7.25 – 7.17 (m, 3H), 4.57 – 4.50 (m, 1H), 4.50 – 4.44 (m, 1H), 2.77 – 2.67 (m, 2H), 2.50 (ddd, *J* = 10.9, 9.0, 6.8 Hz, 1H), 2.37 (ddd, *J* = 10.9, 8.8, 6.6 Hz, 1H), 2.03 – 1.95 (m, 2H), 1.48 (s, 3H); **^{13}C NMR** ($CDCl_3$, 126 MHz) δ (ppm): 142.4, 128.6, 128.5, 125.9, 86.5, 64.4, 44.1, 32.5, 30.1, 27.5; **HRMS** (ESI-TOF) mass calculated for $[M+H]^+$ ($C_{12}H_{16}ONa^+$) expected *m/z* 199.1099; found *m/z* 199.1095.

tert-butyl(4-(2-methyloxetan-2-yl)butoxy)diphenylsilane (3q)

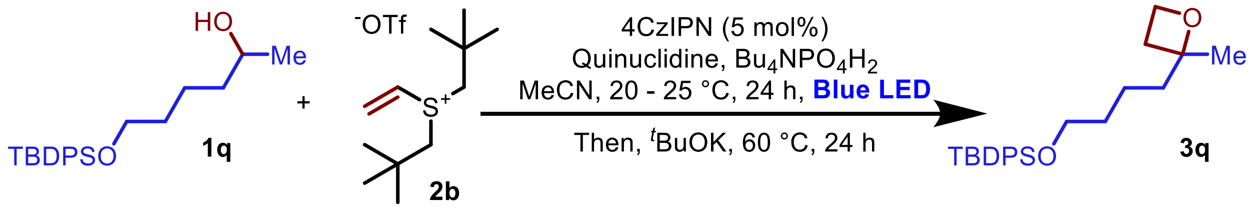

Prepared following general procedure A, using 6-((*tert*-butyldiphenylsilyl)oxy)hexan-2-ol **1q** (1.0 equiv.; 0.2 mmol; 71.3 mg), dineopentyl(vinyl)sulfonium triflate **2b** (1.5 equiv.; 0.3 mmol; 105 mg), 4CzIPN (0.05 equiv.; 0.01 mmol; 7.9 mg), tetra-*n*-butylammonium dihydrogen phosphate (0.25 equiv.; 0.05 mmol; 17.0 mg), quinuclidine (0.25 equiv.; 0.05 mmol; 5.6 mg) and ^{t}BuOK (2.0 equiv.; 0.4 mmol; 44.9 mg). The crude residue was purified by flash column chromatography (SiO_2; gradient 95:5 to 85:15 pentane:Et_2O) to afford compound **3q** (45.6 mg; 60%) as a transparent oil. **R$_f$** (90:10 pentane:Et_2O) 0.3; **IR** (film) ν_{max}/cm^{-1}:3071, 3049, 2931, 2858, 1589, 1472, 1428, 1389, 1373, 1257, 1188, 1110, 994, 964, 823, 740, 702, 614, 505; **^{1}H NMR** ($CDCl_3$, 500 MHz) δ (ppm): 7.70 – 7.65 (m, 4H), 7.45 – 7.35 (m, 6H), 4.49 (dt, *J* = 8.9, 6.4 Hz, 1H), 4.41 (dt, *J* = 8.9, 6.4 Hz, 1H), 3.69 (t, *J* = 6.4 Hz, 2H), 2.47 – 2.39 (m, 1H), 2.34 – 2.26 (m, 1H), 1.68 – 1.58 (m, 4H), 1.48 – 1.41 (m, 2H), 1.39 (s, 3H), 1.06 (s, 9H); **^{13}C NMR** ($CDCl_3$, 126 MHz) δ (ppm): 135.7, 134.2, 129.7, 127.7, 86.8, 64.3, 63.9, 42.0, 33.0, 32.4, 27.3, 27.0, 20.1, 19.4; **HRMS** (ESI-TOF) mass calculated for $[M+Na]^+$ ($C_{24}H_{34}O_2SiNa^+$) expected *m/z* 405.2220; found *m/z* 405.2216.

11-(2-hexyloxetan-2-yl)undecanenitrile (**3r**)

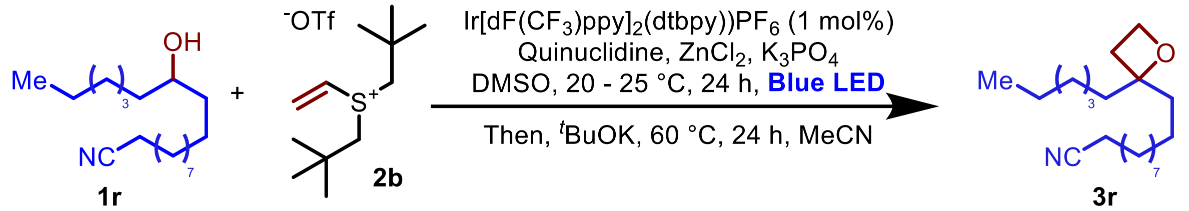

Prepared following general procedure B, using 12-hydroxyoctadecanenitrile **1r** (1.0 equiv.; 0.2 mmol; 56.3 mg), dineopentyl(vinyl)sulfonium triflate **2b** (1.5 equiv.; 0.3 mmol; 105 mg), Ir[dF(CF_3)ppy]$_2$(dtbpy))PF_6 (0.01 equiv.; 0.002 mmol; 2.2 mg), quinuclidine (0.3 equiv.; 0.06 mmol; 6.7 mg), potassium phosphate (1.5 equiv.; 0.3 mmol; 63.7 mg), zinc chloride (2.25 equiv.; 0.45 mmol; 61.3 mg) and ^{t}BuOK (2.0 equiv.; 0.4 mmol; 44.9 mg). The crude residue was purified by flash column chromatography (SiO_2; gradient 95:5 to 90:10 hexane:THF) to afford compound **3r** (35.0 mg; 57%) as a pale yellow oil. **R$_f$** (90:10 hexane:THF) 0.4; **IR** (film) ν_{max}/cm^{-1}: 2926, 2855, 2247, 1693, 1459, 1430, 1403, 1379, 1333, 1257, 1180, 1051, 962, 909, 882, 848, 723, 684; **^{1}H NMR** (500 MHz, $CDCl_3$) δ (ppm): 4.43 (t, *J* = 7.8 Hz, 2H), 2.35 – 2.32 (m, 4H), 1.70 – 1.61 (m, 6H), 1.47 – 1.41 (m, 2H), 1.36 – 1.26 (m, 20H), 0.89 (t, *J* = 7.0 Hz, 3H); **^{13}C NMR** ($CDCl_3$, 126 MHz) δ (ppm): δ 120.0, 89.0, 64.8, 39.9, 39.8, 32.0, 30.7, 30.2, 29.9, 29.7, 29.6, 29.4, 28.9, 28.8, 25.5, 23.3, 22.8, 17.3, 14.2; **HRMS** (ESI-TOF) mass calculated for $[M+Na]^+$ ($C_{20}H_{37}NONa^+$) expected *m/z* 330.2767 found *m/z* 330.2768

4-(oxetan-2-yl)tetrahydro-2*H*-pyran (**3s**)

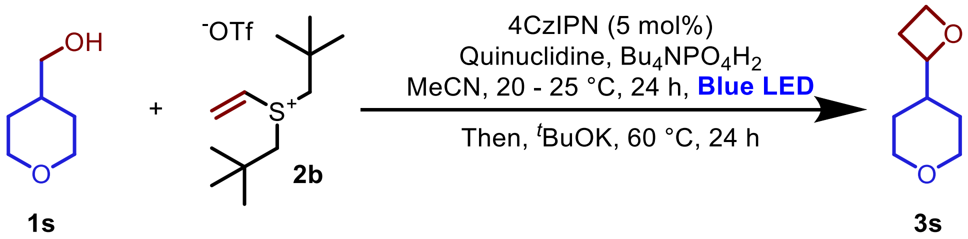

Prepared following general procedure A, using tetrahydropyran-4-methanol **1s** (1.0 equiv.; 0.2 mmol; 23.0 μL), dineopentyl(vinyl)sulfonium triflate **2b** (1.5 equiv.; 0.3 mmol; 105 mg), 4CzIPN (0.05 equiv.; 0.01 mmol; 7.9 mg), tetra-*n*-butylammonium dihydrogen phosphate (0.25 equiv.; 0.05 mmol; 17.0 mg), quinuclidine (0.25 equiv.; 0.05 mmol; 5.6 mg) and ^{t}BuOK (2.0 equiv.; 0.4 mmol; 44.9 mg). Due to high volatility of compound **3s**, the NMR yield was measured (68%) using CH_2Br_2 as internal standard. The crude residue was purified by flash column chromatography (SiO_2; gradient 60:40 to 40:60 pentane:Et_2O; eluent removed using a rotary evaporator, maintaining the water bath below 25 °C and vacuum pressure 450 – 80 mbar) to afford compound **3s** (16.0 mg; 56%) as a pale yellow oil. **R$_f$** (50:50 pentane:Et_2O) 0.35; **IR** (film) ν_{max}/cm^{-1}: 2934, 2845, 1446, 1387, 1235, 1132, 1080, 1017, 979, 751; **^{1}H NMR** ($CDCl_3$, 400 MHz) δ (ppm): 4.73 – 4.62 (m, 1H), 4.60 – 4.41 (m, 2H), 4.07 – 3.95 (m, 2H), 3.47 – 3.32 (m, 2H), 2.66 – 2.54 (m, 1H), 2.46 – 2.33 (m, 1H), 1.97 – 1.83 (m, 1H), 1.79 – 1.70 (m, 1H), 1.54 – 1.45 (m, 1H), 1.35 – 1.21 (m, 2H); **^{13}C NMR** ($CDCl_3$, 126 MHz) δ (ppm):

85.8, 68.5, 67.7, 67.6, 41.9, 27.7, 26.2, 25.3; **HRMS** (ESI-TOF) mass calculated for $[M+H]^+$ ($C_8H_{14}NaO_2^+$) expected *m/z* 165.0886; found *m/z* 165.0880.

2-undecyloxetane (**3t**)

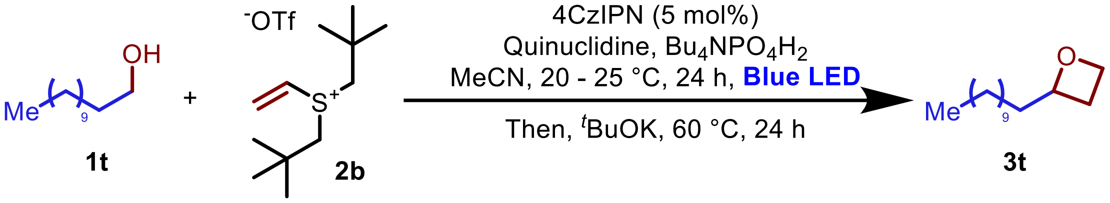

Prepared following general procedure A, using 1-dodecanol **1t** (1.0 equiv.; 0.2 mmol; 44.7 µL), dineopentyl(vinyl)sulfonium triflate **2b** (1.5 equiv.; 0.3 mmol; 105 mg), 4CzIPN (0.05 equiv.; 0.01 mmol; 7.9 mg), tetra-*n*-butylammonium dihydrogen phosphate (0.25 equiv.; 0.05 mmol; 17.0 mg), quinuclidine (0.25 equiv.; 0.05 mmol; 5.6 mg) and *ᵗ*BuOK (2.0 equiv.; 0.4 mmol; 44.9 mg). The crude residue was purified by flash column chromatography (SiO_2; gradient 97:3 to 95:5 pentane:Et_2O) to afford compound **3t** (17.9 mg; 42%) as a pale yellow oil. **R_f** (95:5 pentane:Et_2O) 0.4; **IR** (film) ν_{max}/cm^{-1}: 2922, 2874, 2853, 1465, 1376, 1225, 981, 722; **^{1}H NMR** ($CDCl_3$, 500 MHz) δ (ppm): 4.81 (p, *J* = 6.9 Hz, 1H), 4.65 (td, *J* = 8.0, 5.9 Hz, 1H), 4.49 (dt, *J* = 9.1, 5.8 Hz, 1H), 2.68 – 2.59 (m, 1H), 2.36 – 2.27 (m, 1H), 1.84 – 1.75 (m, 1H), 1.69 – 1.61 (m, 1H), 1.33 – 1.21 (m, 18H), 0.88 (t, *J* = 6.9 Hz, 3H); **^{13}C NMR** ($CDCl_3$, 126 MHz) δ (ppm): 83.0, 68.2, 38.2, 32.1, 29.8, 29.8, 29.7, 29.6, 29.5, 27.9, 24.2, 22.8, 14.3; **HRMS** (ESI-TOF) mass calculated for $[M+NH_4]^+$ ($C_{14}H_{32}NO^+$) expected *m/z* 230.2484; found *m/z* 230.2477.

2-(3-phenylpropyl)oxetane (**3u**)

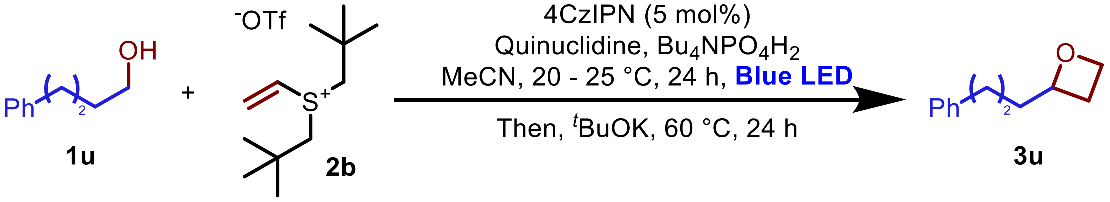

Prepared following general procedure A, using 4-phenyl-1-butanol **1u** (1.0 equiv.; 0.2 mmol; 30.5 µL), dineopentyl(vinyl)sulfonium triflate **2b** (1.5 equiv.; 0.3 mmol; 105 mg), 4CzIPN (0.05 equiv.; 0.01 mmol; 7.9 mg), tetra-*n*-butylammonium dihydrogen phosphate (0.25 equiv.; 0.05 mmol; 17.0 mg), quinuclidine (0.25 equiv.; 0.05 mmol; 5.6 mg) and *ᵗ*BuOK (2.0 equiv.; 0.4 mmol; 44.9 mg). The crude residue was purified by flash column chromatography (SiO_2; gradient 100% DCM to 85:15 to 80:20 pentane:Et_2O) to afford compound **3u** (15.9 mg; 45%) as a transparent oil. **R_f** (85:15 pentane:Et_2O) 0.4; **IR** (film) ν_{max}/cm^{-1}: 3061, 3026, 2932, 2877, 1603, 1496, 1463, 1379, 1225, 1079, 1030, 976, 919, 773, 748, 699; **^{1}H NMR** ($CDCl_3$, 500 MHz) δ (ppm): 7.30 – 7.26 (m, 2H), 7.21 – 7.16 (m, 3H), 4.87 – 4.80 (m, 1H), 4.66 (td, *J* = 8.0, 5.9 Hz, 1H), 4.49 (dt, *J* = 9.1, 5.8 Hz, 1H), 2.70 – 2.60 (m, 3H), 2.36 – 2.28 (m, 1H), 1.89 – 1.80 (m, 1H), 1.74 – 1.66 (m, 2H), 1.66 – 1.59 (m,

1H); **^{13}C NMR** ($CDCl_3$, 126 MHz) δ (ppm): 142.4, 128.6, 128.4, 125.9, 82.7, 68.2, 37.7, 35.8, 27.7, 26.0; **HRMS** (ESI-TOF) mass calculated for $[M+Na]^+$ ($C_{12}H_{16}ONa^+$) expected *m/z* 199.1093; found *m/z* 199.1095.

tert-butyl((5-(oxetan-2-yl)pentyl)oxy)diphenylsilane (**3v**)

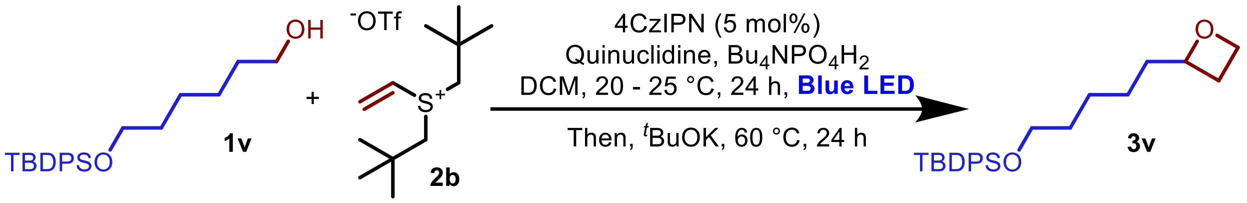

Prepared following general procedure A, using 6-((*tert*-butyldiphenylsilyl)oxy)hexan-1-ol **1v** (1.0 equiv.; 0.2 mmol; 71.3 mg), dineopentyl(vinyl)sulfonium triflate **2b** (1.5 equiv.; 0.3 mmol; 105 mg), 4CzIPN (0.05 equiv.; 0.01 mmol; 7.9 mg), tetra-*n*-butylammonium dihydrogen phosphate (0.25 equiv.; 0.05 mmol; 17.0 mg), quinuclidine (0.25 equiv.; 0.05 mmol; 5.6 mg) in DCM. After 24 h, DCM was removed by blowing with nitrogen, and the solvent was switched to anhydrous MeCN (0.4 mL), then ^{t}BuOK (2.0 equiv.; 0.4 mmol; 44.9 mg) was added and the mixture heated at 60 °C for 24 h. The crude residue was purified by flash column chromatography (SiO_2; gradient 95:5-90:10 pentane:Et_2O) to afford compound **3v** (32.3 mg, 43%) as a yellow oil. **R_f** (90:10 pentane:Et_2O) 0.3; **IR** (film) ν_{max}/cm^{-1}: 3071, 2931, 2858, 1472, 1428, 1389, 1111, 980, 823, 740, 720, 614, 504, 490, 435; **^{1}H NMR** (500 MHz, $CDCl_3$) δ (ppm): 7.68 – 7.64 (m, 4H), 7.43 – 7.36 (m, 6H), 4.82 – 4.77 (m, 1H), 4.67 – 4.63 (m, 1H), 4.53 – 4.49 (dt, *J* = 9.2 Hz, 5.7 Hz, 1H), 3.66 (t, *J* = 6.5 Hz, 2H), 2.67 – 2.60 (m, 1H), 2.34 – 2.37 (m, 1H), 1.82 – 1.75 (m, 1H), 1.67 – 1.62 (m, 1H), 1.60 – 1.54 (m, 2H), 1.41 – 1.35 (m, 2H), 1.34 – 1.23 (m, 2H), 1.05 (s, 9H); **^{13}C NMR** (126 MHz, $CDCl_3$) δ (ppm): 135.7, 134.3, 129.6, 127.7, 82.9, 68.2, 64.0, 38.1, 32.7, 27.8, 27.0, 25.8, 24.0, 19.4; **HRMS** (ESI-TOF) mass calculated for $[M+Na]^+$ ($C_{24}H_{34}O_2SiNa^+$) expected *m/z* 405.2220; found *m/z* 405.2226.

3-methyl-1-oxaspiro[3.5]nonane (**3w**)

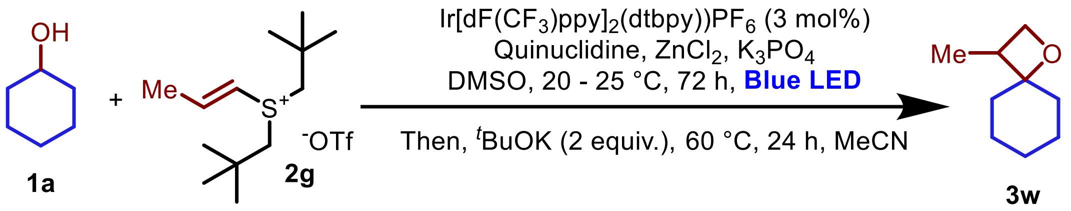

Prepared following a modified version of general procedure B, using cyclohexanol **1a** (1.0 equiv.; 0.14 mmol; 14.8 µL), dineopentyl(prop-1-en-1-yl)sulfonium triflate **2g** (1.5 equiv.; 0.21 mmol; 76.5 mg), Ir[dF(CF_3)ppy]$_2$(dtbpy))PF_6 (0.01 equiv.; 0.0014 mmol; 1.6 mg), quinuclidine (0.3 equiv.; 0.042 mmol; 4.7 mg), potassium phosphate (1.5 equiv.; 0.21 mmol; 44.6 mg), zinc chloride (2.25 equiv.; 0.315 mmol; 42.9 mg) in degassed dry DMSO (700 µL; [**1a**]$_0$ = 0.2 M). The reaction was irradiated

for 14 h under moderate stirring. After which a second batch of Ir[dF(CF_3)ppy]$_2$(dtbpy))PF_6 (0.01 equiv.; 0.0014 mmol; 1.6 mg) and quinuclidine (0.3 equiv.; 0.042 mmol; 4.7 mg) was added, keeping the reaction mixture under argon. The reaction mixture was irradiated again for 24 h, after which a third batch of Ir[dF(CF_3)ppy]$_2$(dtbpy))PF_6 (0.01 equiv.; 0.0014 mmol; 1.6 mg), and quinuclidine (0.15 equiv.; 0.023 mmol; 2.3 mg) was added and the reaction mixture was irradiated again for 16 h. Finally, the $ZnCl_2$ was removed following the general procedure B adjusting the quantity of alumina and solvents accordingly to this scale. Dry MeCN (700 µL) was added followed by ^{t}BuOK (2.0 equiv.; 0.28 mmol; 31.4 mg) and the reaction mixture was heated at 60 °C for 24 h. Due to high volatility of compound **3w**, the ^{1}H NMR yield was measured (44%) on a minimal reaction aliquot using CH_2Br_2 as internal standard. H_2O (200 µL) was added to the reaction mixture and it was extracted with pentane (2 mL × 5). The organic layers were combined, and the solvent removed under rotary evaporation (300 mbar; 5 min with a water bath below 20°C). The crude residue was purified by flash column chromatography (SiO_2; gradient 95:5 to 90:10 pentane:Et_2O) to afford compound **3w** (10.3 mg; 37%) as a pale yellow oil. **R_f** (95:5 pentane:Et_2O) 0.2; **IR** (film) ν_{max}/cm^{-1}: 2934, 2867, 1454, 1387, 1259, 1479, 1025, 804, 616, 567; **^{1}H NMR** ($CDCl_3$, 400 MHz) δ (ppm): 4.57 (dd, *J* = 8.1, 5.9 Hz, 1H), 4.04 – 3.98 (m, 1H), 2.67 – 2.54 (m, 1H), 1.84 – 1.72 (m, 2H), 1.84 – 1.56 (m, 4H), 1.48 – 1.25 (m, 4H), 1.14 (d, *J* = 7.1 Hz, 3H); **^{13}C NMR** ($CDCl_3$, 101 MHz) δ 87.9, 72.5, 39.9, 38.3, 32.7, 25.5, 22.5, 22.3, 13.8. The spectra match with the one reported.[18]

2-methyl-1-oxaspiro[3.5]nonane (**3x**)

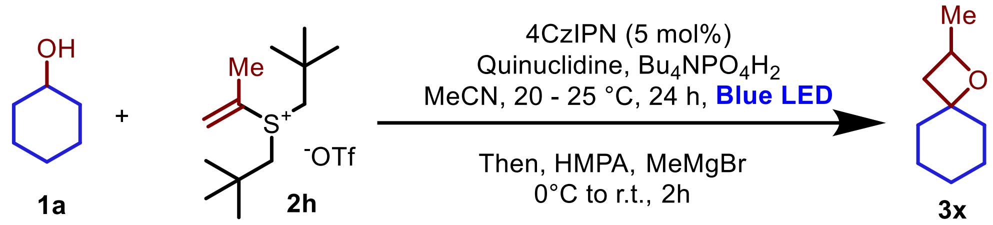

Prepared following general procedure A, using cyclohexanol **1a** (1.0 equiv.; 0.2 mmol; 21.6 µL), dineopentyl(prop-1-en-2-yl)sulfonium triflate **2h** (1.5 equiv.; 0.3 mmol; 109 mg), 4CzIPN (0.05 equiv.; 0.01 mmol; 7.9 mg), tetra-*n*-butylammonium dihydrogen phosphate (0.25 equiv.; 0.05 mmol; 17.0 mg), quinuclidine (0.25 equiv.; 0.05 mmol; 5.6 mg). After 24 h, MeCN was removed under reduced pressure using a Schlenk manifold, and the solvent was switched to dry HMPA (800 µL; 0.25M [**1a**]$_0$). Phenanthrene (1 equiv.; 0.2 mmol; 35.7 mg; as an internal standard) was added and the reaction was cooled down to 0 °C. Then a solution of MeMgBr (3 M in Et_2O; 2 equiv.; 133 µL) was slowly added in 1 min. while the reaction was vigorously stirring. After the addition was completed, the ice bath was removed and the reaction was left warming up to room temperature and kept under vigorously stirring for 2 h. Due to high volatility of compound **3x**, the ^{1}H NMR yield was measured (25%) on a minimal reaction aliquot using phenanthrene as internal standard. The reaction was quenched with a saturated water solution of NH_4Cl (100 µL). The crude was then diluted with H_2O

(2 mL) and extracted with pentane (2 mL × 4). The organic layers were combined, dried over anhydrous $MgSO_4$ and the solvent removed under rotary evaporation (300 mbar; 5 min with a water bath below 20 °C). The crude residue was purified by flash column chromatography (SiO_2; gradient 95:5 to 90:10 pentane:Et_2O) to afford compound **3x** (6.0 mg, 21%) as a pale yellow oil. **R$_f$** (95:5 pentane:Et_2O) 0.2; **IR** (film) ν_{max}/cm^{-1}:2919, 2178, 1261, 1219, 1204, 772, 483, 459; **^{1}H NMR** (500 MHz, $CDCl_3$) δ (ppm): 4.77 – 4.68 (m, 1H), 2.38 (dd, *J* = 10.7, 7.5 Hz, 1H), 1.91 (dd, *J* = 10.7, 6.9 Hz, 1H), 1.86 – 1.78 (m, 1H), 1.78 – 1.57 (m, 6H), 1.38 (d, *J* = 6.1 Hz, 3H), 1.35 – 1.25 (m, 3H); **^{13}C NMR** (126 MHz, $CDCl_3$) δ (ppm): 81.6, 71.5, 40.9, 39.4, 38.4, 25.3, 25.1, 23.0, 22.5; **HRMS** (EI-TOF) mass calculated for $[M]^{•+}$ $(C_9H_{16}O)^{•+}$ expected *m/z* 140.1196; found *m/z* 140.1188.

2-phenyl-1-oxaspiro[3.5]nonane (3y)

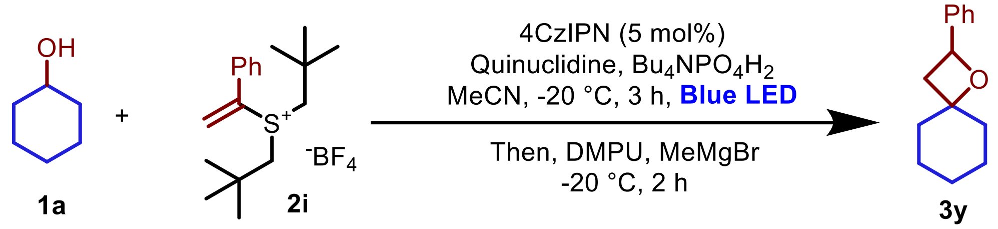

Prepared following a modification of general procedure A, using cyclohexanol **1a** (1.0 equiv.; 0.2 mmol; 21.6 µL), dineopentyl(1-phenylvinyl)sulfonium tetrafluoroborate **2i** (1.5 equiv.; 0.3 mmol; 109 mg), 4CzIPN (0.05 equiv.; 0.01 mmol; 7.9 mg), tetra-*n*-butylammonium dihydrogen phosphate (0.25 equiv.; 0.05 mmol; 17.0 mg), quinuclidine (0.25 equiv.; 0.05 mmol; 5.6 mg). The reaction was placed in a glass basin with a 3D printed support to allow irradiation from the side with a Kessil lamp (a nitrogen flow prevents water condensation on the wall facing the lamp, see section 4.1 for visual details). The reaction was left cooling down for 1 min under stirring (500 rpm) and then the light was turned on. The reaction was left stirring under irradiation for 3 h. The reaction was warmed up at room temperature and the solvent removed using a standard Schlenk manifold. Dry DMPU (800 µL, 0.25M $[\mathbf{1a}]_0$) was added followed by phenanthrene (1 equiv.; 0.2 mmol; 35.7 mg) as internal standard. The reaction was cooled down to -20 °C and then a solution of MeMgBr (3 M in Et_2O; 2.0 equiv.; 133 µL) was slowly added in 1 min while the reaction was vigorously stirring at -20 °C. The reaction was left stirring at -20 °C for 2 h. ^{1}H-NMR analysis of a minimal reaction aliquot confirmed reaction completion. The reaction was quenched with a saturated water solution of NH_4Cl (100 µL). The crude was then diluted with H_2O (4 mL) and extracted with pentane (5 mL × 4). The organic layers were combined, washed once with brine (5 mL), dried over anhydrous $MgSO_4$ and the solvent removed under rotary evaporation. The crude residue was purified by flash column chromatography (SiO_2; gradient 97:3 to 95:5 pentane:Et_2O) to afford compound **3y** (9.8 mg, 24%) as a transparent oil. **R$_f$** (95:5 pentane:Et_2O) 0.3; **IR** (film) ν_{max}/cm^{-1}: 2930, 2855, 1447, 1334, 1260, 1216, 1090, 1017, 798, 698, ; **^{1}H NMR** (500 MHz, $CDCl_3$) δ (ppm): 7.44 – 7.40 (m, 2H), 7.38 – 7.33 (m, 2H), 7.29 – 7.24 (m, 1H), 5.62 (t, *J* = 7.7 Hz, 1H), 2.70 (dd, *J* = 10.8, 8.1 Hz, 1H), 2.27 (dd, *J* = 10.8, 7.3 Hz, 1H),

1.97 (d, J = 10.3 Hz, 1H), 1.88 – 1.64 (m, 5H), 1.45 – 1.28 (m, 4H); **^{13}C NMR** (126 MHz, $CDCl_3$) δ (ppm): 144.4, 128.5, 127.5, 125.4, 82.5, 75.7, 40.8, 39.9, 38.3, 25.3, 23.1, 22.4; **HRMS** (ESI-TOF) mass calculated for $[M+Na]^+$ ($C_{14}H_{18}ONa^+$) expected m/z 225.2150; found m/z 225.1256.

1-((3*S*,8*S*,9*S*,10*R*,13*S*,14*S*,17*S*)-10,13-dimethyl-1,2,4,7,8,9,10,11,12,13,14,15,16,17-tetradecahydrospiro[cyclopenta[a]phenanthrene-3,2'-oxetan]-17-yl)ethan-1-one (**3z**)

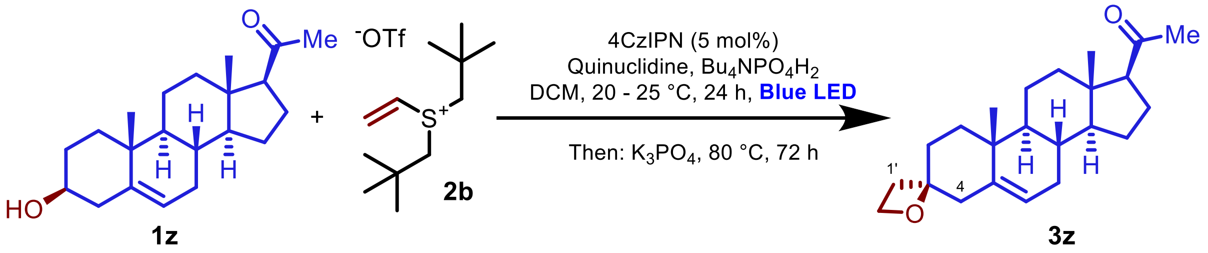

Prepared following general procedure A, using pregnenolone **1z** (1.0 equiv.; 0.2 mmol; 63.3 mg), dineopentyl(vinyl)sulfonium triflate **2b** (1.5 equiv.; 0.3 mmol; 105 mg), 4CzIPN (0.05 equiv.; 0.01 mmol; 7.9 mg), tetra-*n*-butylammonium dihydrogen phosphate (0.25 equiv.; 0.05 mmol; 17.0 mg), quinuclidine (0.25 equiv.; 0.05 mmol; 5.6 mg) and DCM (0.4 mL, 0.5 M). After photochemical step, the crude was transferred in a 5 mL Young tube. K_3PO_4 (2.0 equiv.; 0.4 mmol; 84.8 mg) was added and the mixture heated at 80 °C for 72 h. The crude residue was purified by flash column chromatography (SiO_2; gradient 90:10 to 80:20 hexane:EtOAc) to afford compound **3z** (30.6 mg; 44%; >20:1 d.r.) as a white solid. The stereochemistry of the final compound was elucidated by NOESY analysis by detecting strong NOE interaction between $H_{1'}$ and $H_{4(equat.)}$ and the absence of NOE interaction between $H_{1'}$ and $H_{4(axial)}$. **R_f** (80:20 hexane:EtOAc) 0.4; **$[\alpha]_D^{25}$** = +23 (c = 1.00 $CHCl_3$); **M. P.** = 174-175 °C; **IR** (film) ν_{max}/cm^{-1}: 2935, 2878, 2845, 1698, 1437, 1384, 1355, 1225, 1136, 1064, 975, 958, 758, 593; **^{1}H NMR** ($CDCl_3$, 500 MHz) δ (ppm): 5.41 – 5.36 (m, 1H), 4.53 – 4.44 (m, 2H), 2.52 (t, J = 9.0 Hz, 1H), 2.50 – 2.41 (m, 2H), 2.31 – 2.21 (m, 2H), 2.20 – 2.14 (m, 1H), 2.12 (s, 3H), 2.08 – 1.96 (m, 3H), 1.83 – 1.75 (m, 1H), 1.75 – 1.59 (m, 4H), 1.57 – 1.53 (m, 1H), 1.53 – 1.39 (m, 3H), 1.28 – 1.19 (m, 1H), 1.17 – 1.10 (m, 1H), 1.06 – 1.00 (m, 1H), 0.99 (s, 3H), 0.98 – 0.91 (m, 1H), 0.63 (s, 3H); **^{13}C NMR** ($CDCl_3$, 126 MHz) δ (ppm): 209.7, 139.8, 122.4, 87.5, 64.9, 63.9, 57.1, 50.1, 45.8, 44.1, 39.0, 36.7, 35.5, 34.9, 32.0, 31.9, 31.7, 31.3, 24.6, 23.0, 21.3, 19.1, 13.4; **HRMS** (ESI-TOF) mass calculated for $[M+H]^+$ ($C_{23}H_{35}O_2^+$) expected m/z 343.2637; found m/z 343.2630.

(3a*R*,5*R*,5a*S*,8a*S*,8b*R*)-2,2,7,7-tetramethyl-5-((*R*)-oxetan-2-yl)tetrahydro-5*H*-bis([1,3]dioxolo)[4,5-*b*:4',5'-d]pyran (**3aa**)

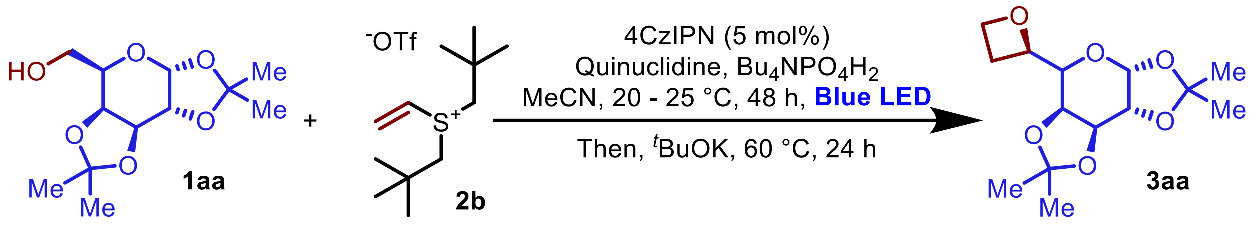

Prepared following general procedure A, using 1,2:3,4-Di-*O*-isopropylidene-α-D-galactopyranose **1aa** (1.0 equiv.; 0.2 mmol; 52.1 mg), dineopentyl(vinyl)sulfonium triflate **2b** (1.5 equiv.; 0.3 mmol; 105 mg), 4CzIPN (0.05 equiv.; 0.01 mmol; 7.9 mg), tetra-*n*-butylammonium dihydrogen phosphate (0.25 equiv.; 0.05 mmol; 17.0 mg), quinuclidine (0.25 equiv.; 0.05 mmol; 5.6 mg) and ^{t}BuOK (2.0 equiv.; 0.4 mmol; 44.9 mg). The crude residue was purified by flash column chromatography (SiO_2; gradient 80:20 to 70:30 hexane:EtOAc) allowed separation of the minor diastereoisomer to afford compound **3aa** (24.9 mg; 43%; >20:1 d.r.) as a pale yellow oil. The d.r. detected by ^{1}H NMR in the crude reaction mixture was 6:1. **R$_f$** (80:20 hexane:EtOAc) 0.3; **$[\alpha]_D^{25}$** = –38° (c = 1.00 $CHCl_3$); **IR** (film) ν_{max}/cm^{-1}: 2987, 2936, 1456, 1382, 1255, 1212, 1169, 1067, 1000, 916, 896, 773, 510; **^{1}H NMR** ($CDCl_3$, 500 MHz) δ (ppm): 5.59 (d, *J* = 5.0 Hz, 1H), 4.95 (q, *J* = 6.9 Hz, 1H), 4.68 (td, *J* = 8.0, 5.8 Hz, 1H), 4.64 – 4.57 (m, 2H), 4.36 – 4.28 (m, 2H), 3.96 (dd, *J* = 6.2, 1.9 Hz, 1H), 2.74 – 2.64 (m, 2H), 1.58 (s, 3H), 1.43 (s, 3H), 1.35 (s, 3H), 1.32 (s, 3H); **^{13}C NMR** ($CDCl_3$, 126 MHz) δ (ppm): 109.4, 108.8, 96.5, 80.2, 70.8, 70.7, 70.3, 70.2, 70.1, 26.3, 26.0, 25.1, 24.9, 24.4; **HRMS** (ESI-TOF) mass calculated for $[M+Na]^+$ ($C_{14}H_{22}O_6Na^+$) expected *m/z* 309.1309; found *m/z* 309.1310.

(8*R*,9*S*,10*R*,13*S*,14*S*,17*R*)-10,13-dimethyl-1,6,7,8,9,10,11,12,13,14,15,16-dodecahydrospiro[cyclopenta[*a*]phenanthrene-17,2'-oxetan]-3(2*H*)-one (**3ab**)

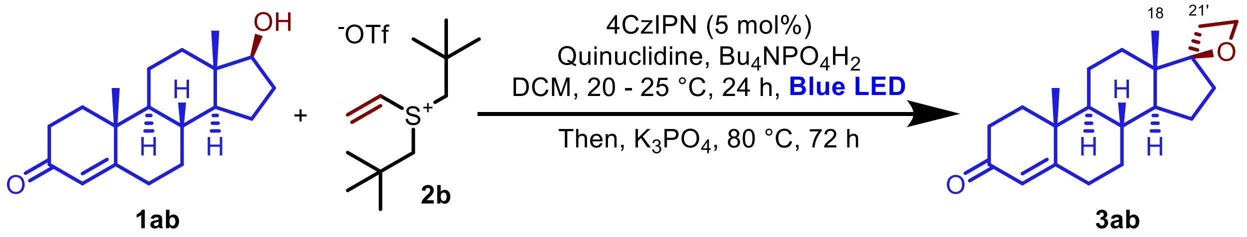

Prepared following general procedure A, using testosterone **1ab** (1.0 equiv.; 0.2 mmol; 57.7 mg), dineopentyl(vinyl)sulfonium triflate **2b** (1.5 equiv.; 0.3 mmol; 105 mg), 4CzIPN (0.05 equiv.; 0.01 mmol; 7.9 mg), tetrabutylammonium dihydrogen phosphate (0.25 equiv.; 0.05 mmol; 17.0 mg), quinuclidine (0.25 equiv.; 0.05 mmol; 5.6 mg) and DCM (0.4 mL, 0.5 M). After photochemical step, the crude was transferred in a 5 mL Young tube. Then K_3PO_4 (2.0 equiv.; 0.4 mmol; 84.8 mg) was added and heated at 80 °C for 72 h. The crude residue was purified by flash column chromatography (SiO_2; gradient 75:25 to 60:40 hexane:EtOAc) to afford compound **3ab** (25.9 mg; 41%; >20:1 d.r.)

as a white solid. The stereochemistry of the final compound was elucidated by NOESY analysis by detecting total absence of NOE interaction between $H_{21'}$ and H_{18}. **Rf** (70:30 hexane:EtOAc) 0.3; **[α]D**[25] = +69 (c = 1.00 $CHCl_3$); **M. P.** = 170-171 °C; **IR** (film) ν_{max}/cm^{-1}: 2944, 2875, 1674, 1616, 1451, 1270, 1229, 1185, 1066, 1044, 978, 868; **^{1}H NMR** ($CDCl_3$, 500 MHz) δ (ppm): 5.72 (s, 1H), 4.46 – 4.40 (m, 1H), 4.34 – 4.27 (m, 1H), 2.76 (ddd, *J* = 11.0, 8.5, 6.2 Hz, 1H), 2.45 – 2.31 (m, 3H), 2.31 – 2.23 (m, 2H), 2.10 – 2.01 (m, 3H), 1.90 – 1.80 (m, 2H), 1.76 – 1.69 (m, 1H) 1.66 – 1.57 (m, 2H), 1.57 – 1.45 (m, 3H), 1.34 – 1.22 (m, 1H), 1.20 (s, 3H), 1.03 – 0.96 (m, 1H), 0.96 – 0.85 (m, 2H), 0.81 (s, 3H); **^{13}C NMR** ($CDCl_3$, 126 MHz) δ (ppm): 199.7, 171.3, 124.0, 96.4, 65.0, 53.8, 48.0, 44.6, 38.8, 37.8, 36.3, 35.9, 34.1, 32.9, 32.1, 31.6, 31.0, 23.2, 20.8, 17.6, 12.0; **HRMS** (ESI-TOF) mass calculated for $[M+Na]^+$ ($C_{21}H_{30}NaO_2^+$) expected *m/z* 337.2138; found *m/z* 337.2127.

4.3. Elucidation of the stereochemistry of 3aa

Compound **3aa** or derivatives were unsuitable for crystallographic analysis. As the stereochemistry of C_3 within **3aa** is forged during the radical addition of vinyl sulfonium (and not in the oxetane cyclization), the stereochemistry was elucidated as presented in Scheme S2. Intermediate sulfonium **S1,** obtained from **1aa** using identical photochemical conditions used for the synthesis of **3aa** (see also experimental details in the following section), was isolated and not subjected to cyclisation. **S1** was then converted to **S2** *via* sulfonium amination.[19] In a separate chemical synthesis, the same compound (**S2**) was obtained via reductive amination[20] of known aldehyde **S3**[21] with defined stereochemistry. Compound **S2** obtained through the two synthetic routes in Scheme S2 showed identical spectral data. From this experiment, the stereochemistry in C_3 was unambiguously inferred.

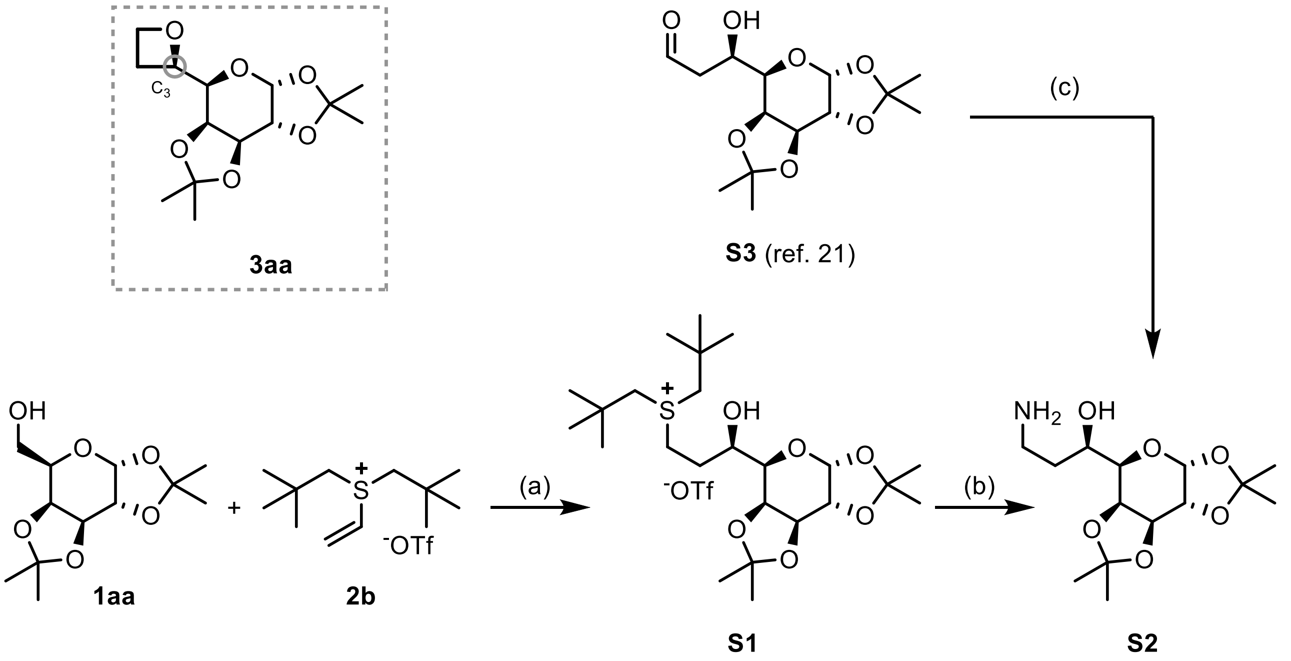

Scheme S2: Elucidation of stereochemistry of **3aa**. (a) 4CzIPN (5 mol%), Quinuclidine, $Bu_4NPO_4H_2$, MeCN, 24 h, r.t. (b) NH_3 (7M in MeOH), 130 °C, 3 h (c) $NaCNBH_3$ (3 equiv.), sat. NH_4OAc in EtOH/30% NH_3 (aq.) (5:2), 80 °C, EtOH, 18h.

Synthesis of S2 from aldehyde S3

Compound **S2** was obtained via reductive amination of **S3** as following.[20]

To a solution of Aldehyde **S3** (1 equiv.; 0.042 mmol; 12.6 mg,) in a saturated solution of NH_4OAc in EtOH (850 µL) were added $NaCNBH_3$ (3 equiv.; 0.13; 7.9 mg) and 30% aq. NH_3 (340 µL). The mixture was stirred at 80 °C for 18 h, cooled to room temperature, and concentrated under reduced pressure. The crude mixture was taken up in DCM (2 mL) and washed with a saturated $NaHCO_3$ solution (1 mL). The aqueous layer was extracted further with DCM (3 x 2 mL) the combined organic layers were dried over Na_2SO_4. The organic layers were combined and dried over $MgSO_4$, filtered, and concentred under reduced pressure. The residue was purified by flash column chromatography (SiO_2; 90:10 DCM:MeOH + 2 % ammonia in water (35%)) to afford the compound (1.57 mg, 13 %) as a colourless oil.

Synthesis of S2 from sulfonium intermediate S1

1,2:3,4-Di-*O*-isopropylidene-α-D-galactopyranose **1aa** (1.0 equiv.; 0.2 mmol; 52.06 mg), dineopentyl(vinyl)sulfonium triflate **2b** (1.5 equiv.; 0.3 mmol; 105 mg), 4CzIPN (0.05 equiv.; 0.01 mmol; 7.9 mg), quinuclidine (0.25 equiv.; 0.05 mmol; 5.6 mg), and tetra-*n*-butylammonium phosphate (0.25 equiv.; 0.05 mmol; 17.0 mg) were introduced into a Schlenk tube. The atmosphere was exchanged to argon and degassed dry MeCN (0.4 mL, 0.5 M, previously degassed through 10 min argon sparging) was introduced through a syringe. The vessel was sealed and placed in a glass-wall water bath where a fan was blowing air to keep the water temperature between 20 - 25 °C. See Fig. S2 for visual details of the reaction setup. The reaction was irradiated through the glass wall with blue light (Kessil lamp A160WE Tuna Blue Saltwater LED Light 40 W) for 24 h under moderate stirring (500 rpm). The crude residue was purified by flash column chromatography (SiO_2; gradient 98:2 to 96:4 DCM:MeOH) to afford compound **S1** (26.4 mg, 22%) as a yellow sticky oil. **R_f** (96:4 DCM:MeOH) 0.3; **IR** (film) ν_{max}/cm^{-1}: 2963, 1478, 1373, 1254, 1223, 1161, 1068, 1030, 1002, 901, 774, 638, 573, 517; **^{1}H NMR** (500 MHz, $CDCl_3$) δ (ppm): 5.49 (d, *J* = 5.0 Hz, 1H), 4.62 (dd, *J* = 7.8, 2.4 Hz, 1H), 4.47 (dd, *J* = 7.9, 2.0 Hz, 1H), 4.31 (dd, *J* = 5.1, 2.4 Hz, 1H). 3.98 – 3.91 (m, 1H), 3.76 – 3.63 (m, 3H), 3.63 – 3.58 (m, 2H), 3.55 (d, *J* = 14.2 Hz, 1H), 3.37 (d, *J* = 14.1 Hz, 1H), 3.32 (d, *J* = 14.1 Hz, 1H), 2.32 – 3.63 (m, 1H), 2.05 – 1.98 (m, 1H), 1.53 (s, 3H), 1.43 (s, 3H), 1.35 (s, 3H), 1.31 (s, 3H) 1.18 – 1.14 (m, 18H); **^{19}F NMR** ($CDCl_3$, 376 MHz) δ (ppm): -78.3 (s); **^{13}C NMR** ($CDCl_3$, 126 MHz) δ (ppm): 109.4, 108.9, 96.5, 70.7, 70.7, 70.4, 69.8, 68.0, 57.8, 57.7, 41.8, 32.8, 32.6, 29.2, 29.0, 28.9, 26.2, 26.1, 25.0, 24.4; **HRMS** (ESI-TOF) mass calculated for $[M]^+$ ($C_{24}H_{45}O_6S^+$) expected *m/z* 461.2931 found *m/z* 461.2933.

Sulfonium adduct **S1** (1.0 equiv.; 0.025 mmol; 15.7 mg) was added to a microwave vial, ammonia (7M in methanol; 0.85 mL; 0.03M) was added and the reaction was heated to 130 °C for 3 h in a

microwave reactor. A solution of NaOH (2 M; 1 mL) was added and the mixture extracted three times with DCM (3 x 5 mL). The organic phases were collected and dried with anhydrous Na_2SO_4 and the solvent removed by rotary evaporator. The residue was purified by flash column chromatography (SiO_2; 90:10 DCM:MeOH + 2 % ammonia in water (35%)) to afford compound **S2** (3.1 mg, 39 %) as a colourless oil. **R_f** (90:10 DCM:MeOH + 2 % ammonia in water (35%)) 0.2; **$[\alpha]_D^{25}$** = – 8.0 (c = 0.50 $CHCl_3$); **IR** (film) ν_{max}/cm^{-1}: 3375, 2985, 2933, 1457, 1308, 1256, 1211, 1169, 1066, 1000, 899; **^{1}H NMR** (500 MHz, $CDCl_3$) δ (ppm): δ 5.52 (d, J = 5.0 Hz, 1H), 4.61 (dd, J = 8.0, 2.3 Hz, 1H), 4.53 (dd, J = 8.1, 1.8 Hz, 1H), 4.30 (dd, J = 5.0, 2.3 Hz, 1H), 4.01 (td, J = 9.0, 2.4 Hz, 1H), 3.58 (dd, J = 8.8, 1.8 Hz, 1H), 3.24 – 3.17 (m, 1H), 2.97 – 2.90 (m, 1H). 2.01 –1.96 (m, 1H) 1.54 – 1.49 (m, 4H), 1.47 (s, 3H), 1.38 (s, 3H), 1.33 (s, 3H); **^{13}C NMR** ($CDCl_3$, 126 MHz) δ (ppm): 109.2, 108.6, 96.6, 71.6, 70.9, 70.7, 70.4, 70.3, 40.7, 33.9, 26.2, 26.1, 25.1, 24.4; **HRMS** (ESI-TOF) mass calculated for $[M+H]^+$ ($C_{14}H_{26}NO_6^+$) expected m/z 304.1755 found m/z 304.1751.

5. Scope limitations

Activated alcohols (allyl, benzylic and propargylic alcohols) and substrates bearing terminal olefins did not undergo the desired reactivity, leading instead to extensive alcohol starting material recovery. Steric hindrance also has an impact in this reaction, as demonstrated by the unsuitability of isoborneol for this reactivity (in contrast, the less hindered norborneol leads to good results, entry **3l**). A selection of unsuccessful substrates is reported below.

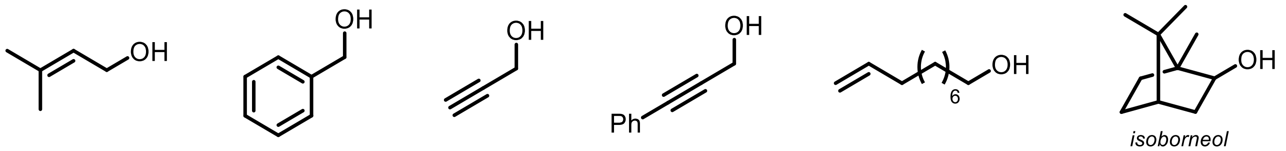

6. Mechanistic investigations

6.1. Experiment carried out in the presence of TEMPO as radical inhibitor

To elucidate the intervention of a radical mechanism, the reaction was carried out in the presence of 2,2,6,6-tetramethyl-1-piperidinyloxy (TEMPO) free radical. The reaction was stopped after irradiation, and the absence of the desired sulfonium intermediate was unambiguously inferred via analysis of the crude reaction mixture via HRMS and ^{1}H-NMR analysis.

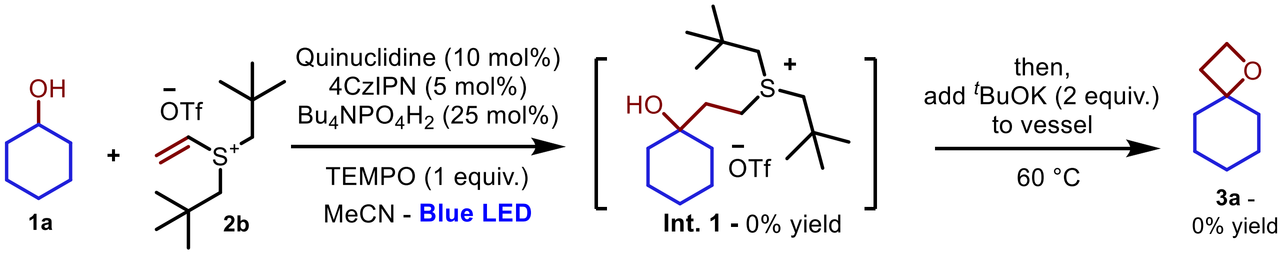

Cyclohexanol **1a** (1.0 equiv.; 0.1 mmol), dineopentyl(vinyl)sulfonium triflate **2b** (1.5 equiv.; 0.15 mmol; 52.6 mg), 4CzIPN (0.05 equiv.; 0.005 mmol; 3.9 mg), quinuclidine (0.10 equiv.; 0.01 mmol; 1.1 mg), and tetra-*n*-butylammonium phosphate (0.25 equiv.; 0.025 mmol; 8.5 mg) and TEMPO (1.0 equiv.; 0.1 mmol; 15.6 mg) were introduced into a Schlenk tube. The atmosphere was exchanged to argon and degassed dry MeCN (0.2 mL, 0.5 M, previously degassed through 10 min argon sparging) was introduced through a syringe. The vessel was sealed and placed in a glass-wall water bath where a fan was blowing air to keep the water temperature between 20 - 25 °C. See Fig. S2 for visual details of the reaction setup. The reaction was irradiated through the glass wall with blue light (Kessil lamp A160WE Tuna Blue Saltwater LED Light 40 W) for 24 h under moderate stirring (500 rpm). A 25 μL of this solution was then collected under an Ar atmosphere and diluted with $CDCl_3$ (500 μL) and analysed via ^{1}H-NMR and HRMS. No traces of the desired intermediate were observed, and 82% starting material recovery was observed via ^{1}H-NMR analysis using mesitylene (1.0 equiv.; 0.1 mmol; 14.0 μL) as internal standard.

The vessel was then removed from the water bath and ^{t}BuOK (2.0 equiv.; 0.2 mmol; 22.4 mg) was introduced into the vessel. The mixture was heated to 60 °C without irradiation for 24 h under vigorous stirring (700 rpm). After cooling down to room temperature, dibromomethane (1.0 equiv.; 0.1 mmol; 7.0 μL) was then added to the reaction mixture and 25 μL of this solution were collected and analyzed via ^{1}H-NMR. As expected, no traces of oxetane were detected.

6.2. Fluorescence quenching studies

The emission spectra were recorded using an Edinburgh Instruments FLS980, equipped with a 450 W continuous xenon lamp (Xe1). 2 mL of a $9.51 \cdot 10^{-6}$ M solution of 4CzIPN in acetonitrile (spectrophotometric grade) were placed in a Hellma® fluorescence cuvette, 10x10 mm light path Supersil® quartz, equipped with PTFE lid. The solution was degassed through argon sparging for two minutes, and analysed immediately. The excitation wavelength was fixed at 420 nm, while the emission light was acquired from 490 nm to 650 nm. For quenching data the emission wavelength was fixed to 550 nm.

Different solutions, with different concentration of quencher were prepared and analysed following the procedure detailed above, the results are presented in Fig. S4 and Fig. S5. The concentration of 4CzIPN photocatalyst was maintained constant, varying only the quencher concentration.

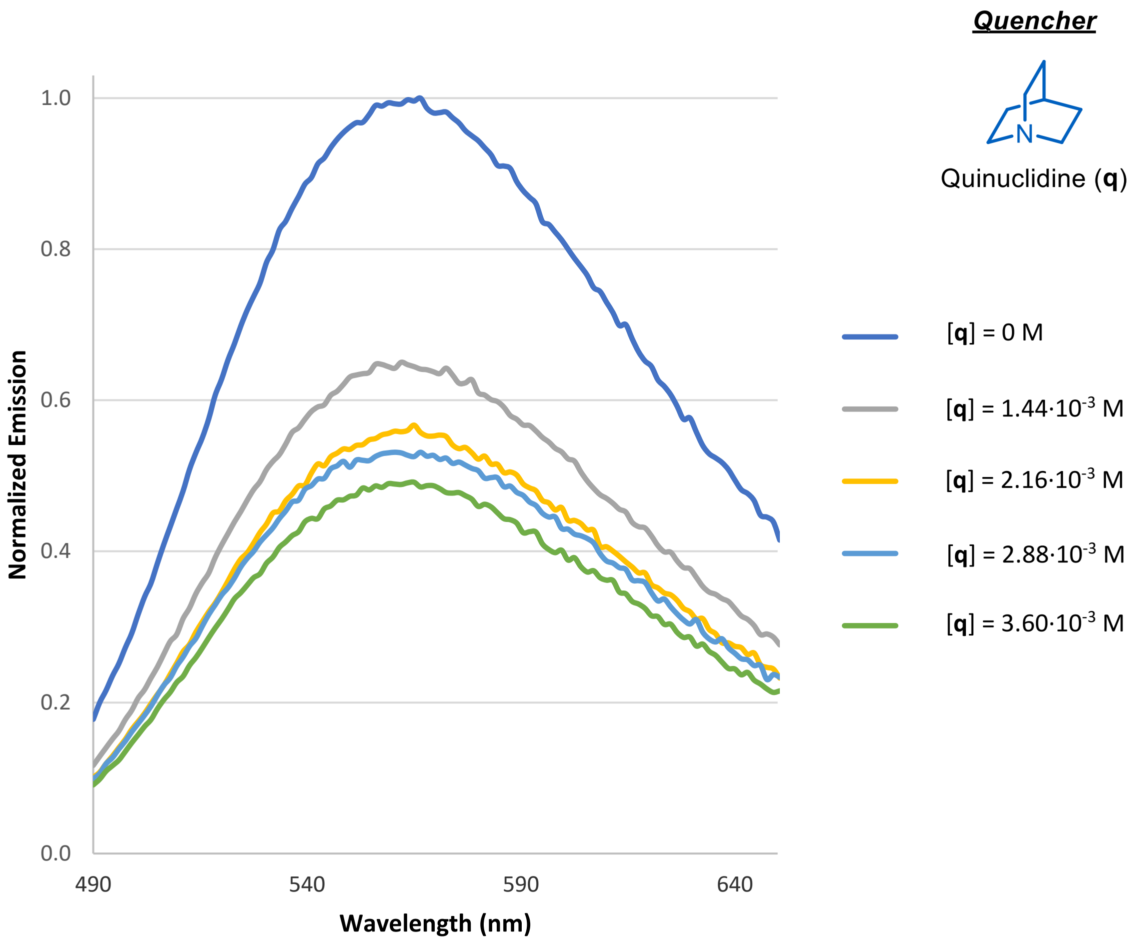

Fig. S4: Emission spectrum of solutions of 4CzIPN ($9.51 \cdot 10^{-6}$ M in acetonitrile) at different concentrations of quinuclidine (**q**).

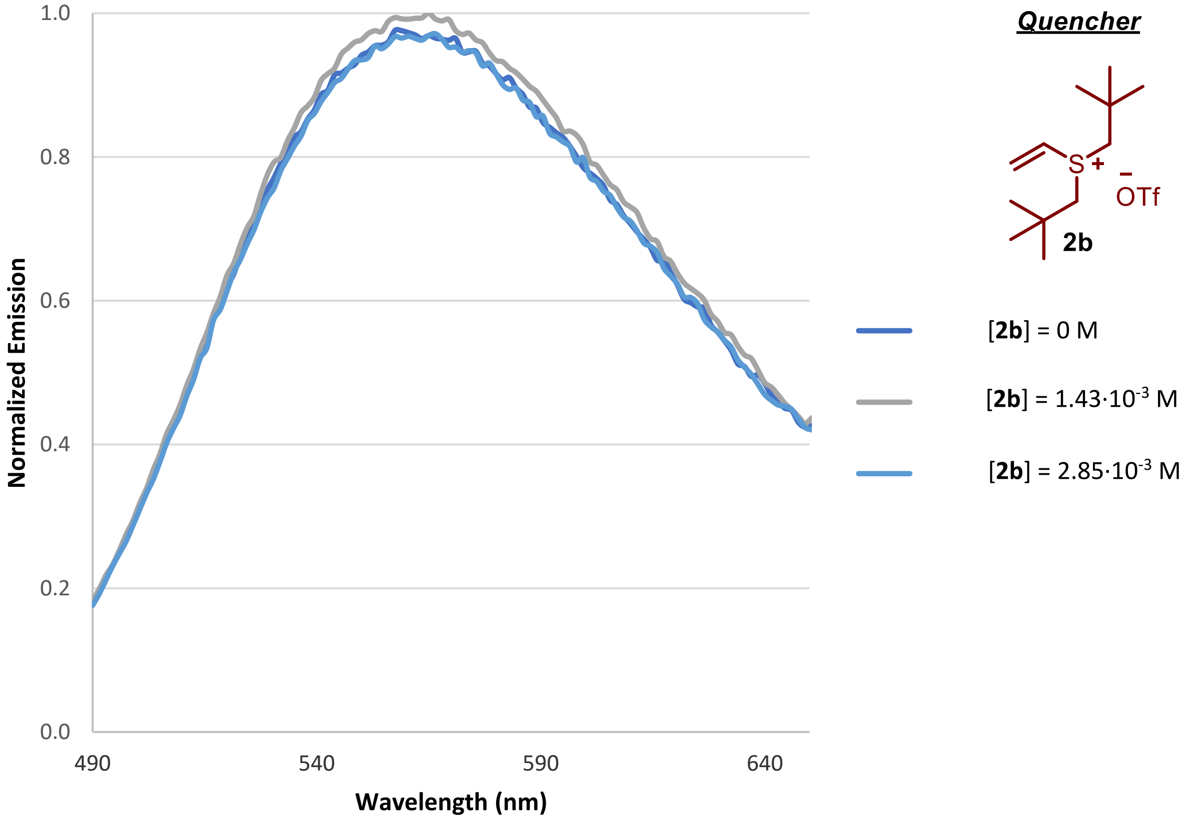

Fig. S5: Emission spectrum of solutions of 4CzIPN ($9.51 \cdot 10^{-6}$ M in acetonitrile) at different concentrations of vinyl sulfonium **2b**.

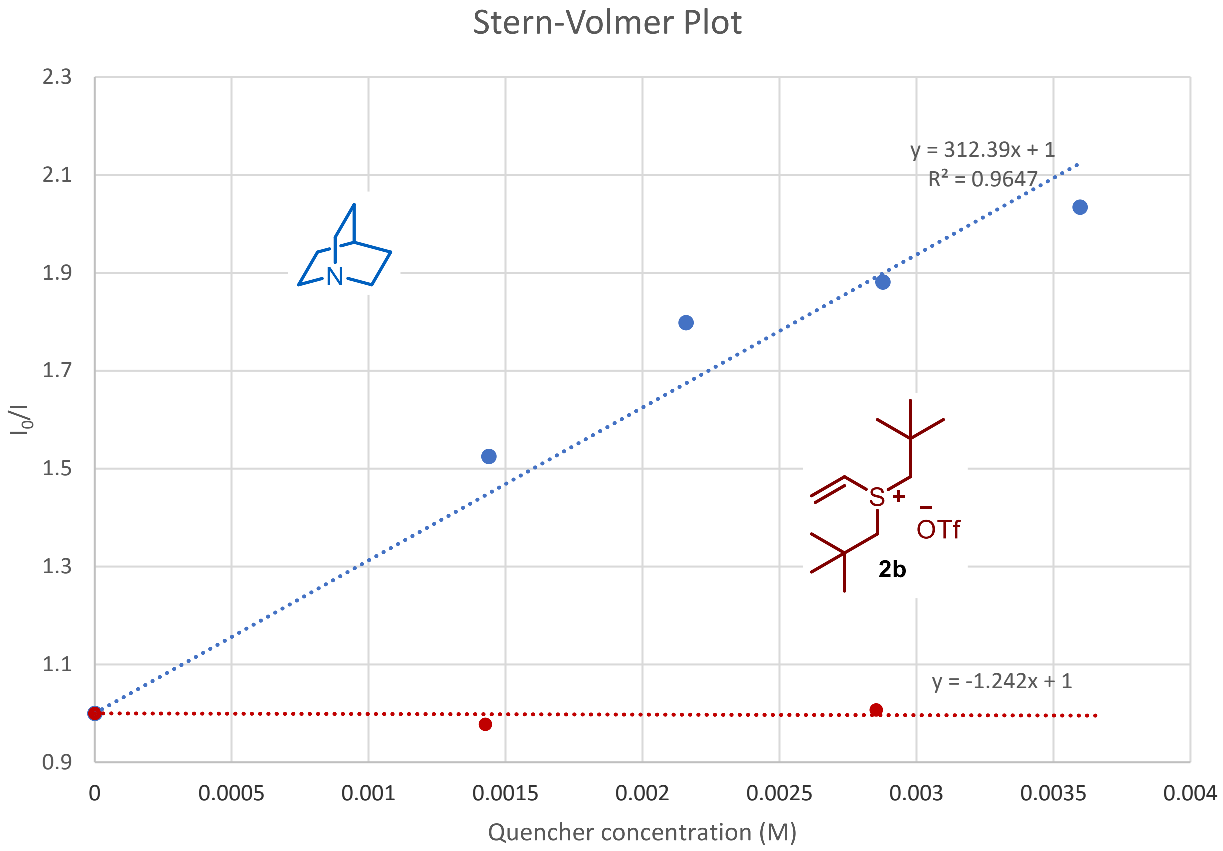

Fig. S6: Stern-Volmer plot for the quenching studies above. Emission wavelength fixed at 550 nm.

As presented in figs. S4 - S6, quinuclidine is an effective quencher for 4CzIPN (K_{SV} = 312.4 M^{-1}). In contrast, sulfonium **2b** is not a quencher for 4CzIPN. This suggests the occurrence of a photochemical process between the photoexcited 4CzIPN and quinuclidine, and no interaction with **2b**.

6.3. Electrochemical studies

Cyclic voltammetry analyses were performed using a CH Instruments Model 600E potentiostat. Electrochemical grade tetrabutylammonium hexafluorophosphate (193.7 mg, 0.5 mmol) was added to a 5 mL, 5 mM solution of analyte (**2b**) in dry MeCN and the solution was vigorously bubbled with argon for 5 minutes prior to the measurement. The measure was carried out under an argon atmosphere. The anodic/cathodic peak potentials (E_p^a and E_p^c) were measured using a 3 mm glassy carbon working electrode, a platinum wire counter electrode, and a 10 mM Ag/$AgNO_3$ reference electrode at 100 mV/s scan rate using ferrocene as internal standard. The measurements are reported in graphs vs SCE, considering the conversion SCE = +420 mV Fc/Fc^+.[22] E_p were obtained from the graphs as the potential corresponding to the maximum current observed as presented in Fig. S7.

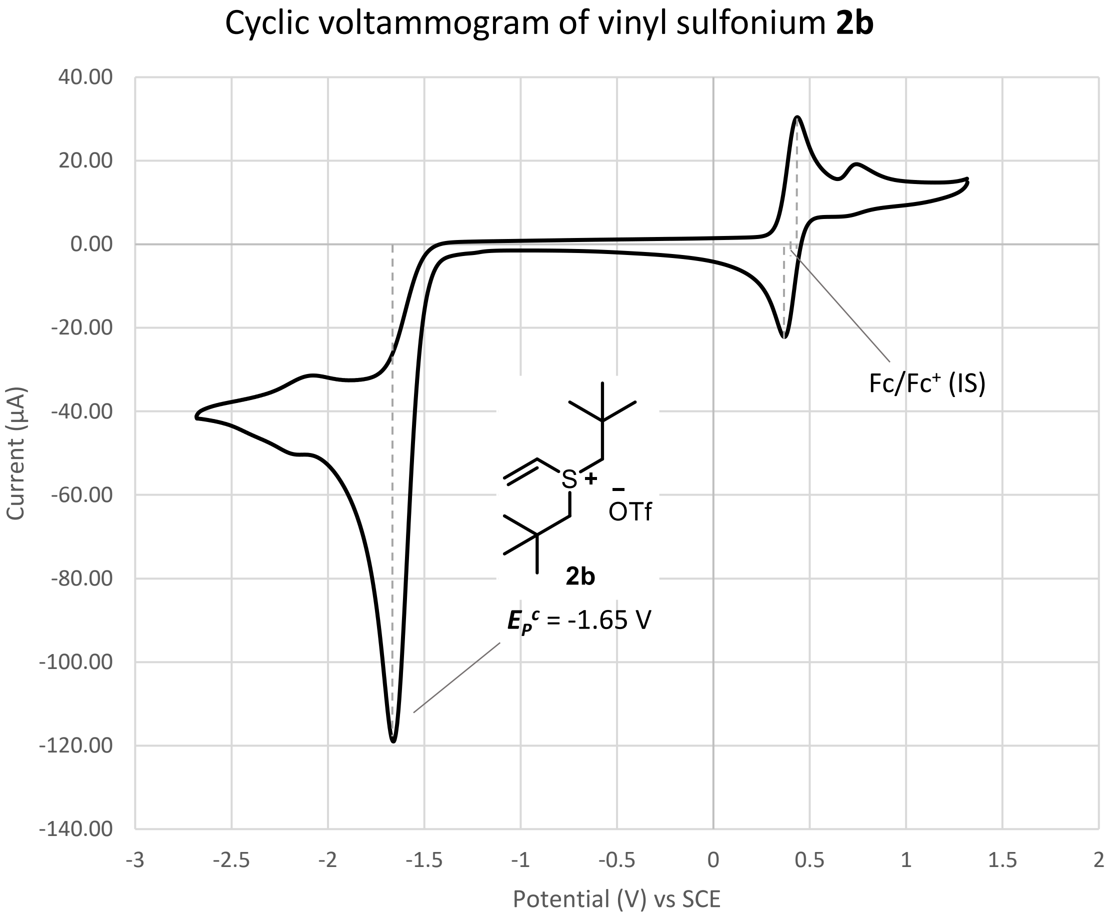

Fig. S7: Cyclic voltammograms of a 5 mM solution of **2b** in the presence of ferrocene as internal standard.

The cyclic voltammetry analysis of vinyl sulfonium **2b** shows a completely irreversible reduction wave with $E_p{}^c$ = −1.65 V (vs SCE). The electrochemical proprieties of the compound suggest that electron-transfer with the photoexcited 4CzIPN is thermodynamically unfavourable [$E_{1/2}$(4CzIPN*/4CzIPN$^{\bullet+}$) = −1.18 V vs SCE).[23] This is in agreement with the inability of sulfonium **2b** to quench the excited state of 4CzIPN, elucidated during our fluorescence quenching studies (Figs. S5 and S6). Please note that no even the anionic ground-state 4CzIPN$^{\bullet-}$ radical anion (generated if a reductive quenching regime is operating) would be able to reduce **2b** [$E_{1/2}$(4CzIPN/4CzIPN$^{\bullet-}$) = −1.24 V vs SCE],[23] therefore **2b** should be insensitive to redox chemistry under the reaction conditions reported in this study.

In contrast, the oxidation of quinuclidine **q** [$E_p{}^a$ = +1.1 V vs SCE][24] by the photoexcited 4CzIPN [$E_{1/2}$(4CzIPN*/4CzIPN$^{\bullet-}$) = +1.43 V vs SCE][23] is thermodynamically feasible. This is in agreement with the ability of quinuclidine to quench the excited state of 4CzIPN, elucidated during our fluorescence quenching studies (Figs. S4 and S6). Taken together, the mechanistic studies above suggest the scenario reported in the main text, Scheme 3.

7. References and Notes

[1] Huang, H.-M.; Bellotti, P.; Pflüger, P. M.; Schwarz, J. L.; Heidrich, B.; Glorius, F. *J. Am. Chem. Soc.* **2020**, *142*, 10173–10183.

[2] Gopinath, P.; Chandrasekaran, S. *Eur. J. Org. Chem.* **2018**, 6541–6547.

[3] Soulard, V.; Villa, G.; Vollmar, D. P.; Renaud, P. *J. Am. Chem. Soc.* **2018**, *140*, 155–158.

[4] Paraja, M.; Matile, S. *Angew. Chem. Int. Ed.* **2020**, *59*, 6273–6277.

[5] Yar, M.; McGarrigle, E. M.; Aggarwal, V. K. *Angew. Chem. Int. Ed.* **2008**, *20*, 3784 –3786.

[6] Wang, Y.; Zhang, W.; Colandrea, V. J.; Jimenez, L. S. *Tetrahedron* **1999**, *55*, 10659– 10672.

[7] Juliá, F.; Yan, J.; Paulus, F.; Ritter, T. *J. Am. Chem. Soc.* **2021**, *143*, 12992–12998.

[8] Huang, H.; Yu, C.; Zhang, Y.; Zhang, Y.; Mariano, P. S.; Wang, W. *J. Am. Chem. Soc.* **2017**, *139*, 9799–9802.

[9] Garreau, M.; Vaillant, F. Le; Waser, J. *Angew. Chem. Int. Ed.* **2019**, *58*, 8182–8186.

[10] Landini, D.; Rolla, F. *Synthesis* **1974**, 565–566.

[11] Freeman, F.; Angeletakis, C. N.; Maricich, T. *J. Org. Mag. Reson.* **1981**, *17*, 53–58.

[12] Zhang, Y.-L.; Yang, L.; Wu, J.; Zhu, C.; Wang, P. *Org. Lett.* **2020**, *22*, 7768–7772.

[13] Conditions inspired from: Dussault, P. H.; Trullinger, T. K.; Noor-e-Ain, F. *Org. Lett.* **2002**, *4*, 4591–4593.

[14] Conditions inspired from: Picard D; Bats, J -P; Moulines, J, P. L. *Synthesis.* **1981**, 550–551.

[15] Conditions inspired from: Vitale, P.; Perna, F. M.; Agrimi, G.; Scilimati, A.; Salomone, A.; Cardellicchio, C.; Capriati, V. *Org. Biomol. Chem.* **2016**, *14*, 11438–11445.

[16] Conditions inspired from: Guo, B.; Schwarzwalder, G.; Njardarson, J. T. *Angew. Chem. Int. Ed.* **2012**, *51*, 5675–5678.

[17] Conditions inspired from: Servrin, M.; Krief, A. *Tetrahedron Lett.* **1980**, *21*, 585–586.

[18] Christlieb, M.; Davies, J. E.; Eames, J.; Hooley, R.; Warren, S. *J. Chem. Soc. Perkin Trans. 1* **2001**, 2983–2996.

[19] Paul, S.; Filippini, D.; Silvi, M. *J. Am. Chem. Soc.* **2023**, *145*, 2773–2778.

[20] Dangerfield, E. M.; Plunkett, C. H.; Win-Mason, A. L.; Stocker, B. L.; Timmer, M. S. M. *J. Org. Chem.* **2010**, *75*, 5470–5477.

[21] Sharma, G.; Cherukupalli, G.; Rao, M. Ramana; Kunwar, A. *Synthesis* **2007**, 2100–2106.

[22] Roth, H. G.; Romero, N. A.; Nicewicz, D. A. *Synlett* **2016**, *27*, 714–723.

[23] Speckmeier, E.; Fischer, T. G.; Zeitler, K. *J. Am. Chem. Soc.* **2018**, *140*, 15353–15365.

[24] Jeffrey, J. L.; Terrett, J. A.; MacMillan, D. W. *Science* **2015**, *349*, 1532–1536.

8. NMR Spectra

Dineopentyl sulfide

^{1}H-NMR ($CDCl_3$, 500 MHz)

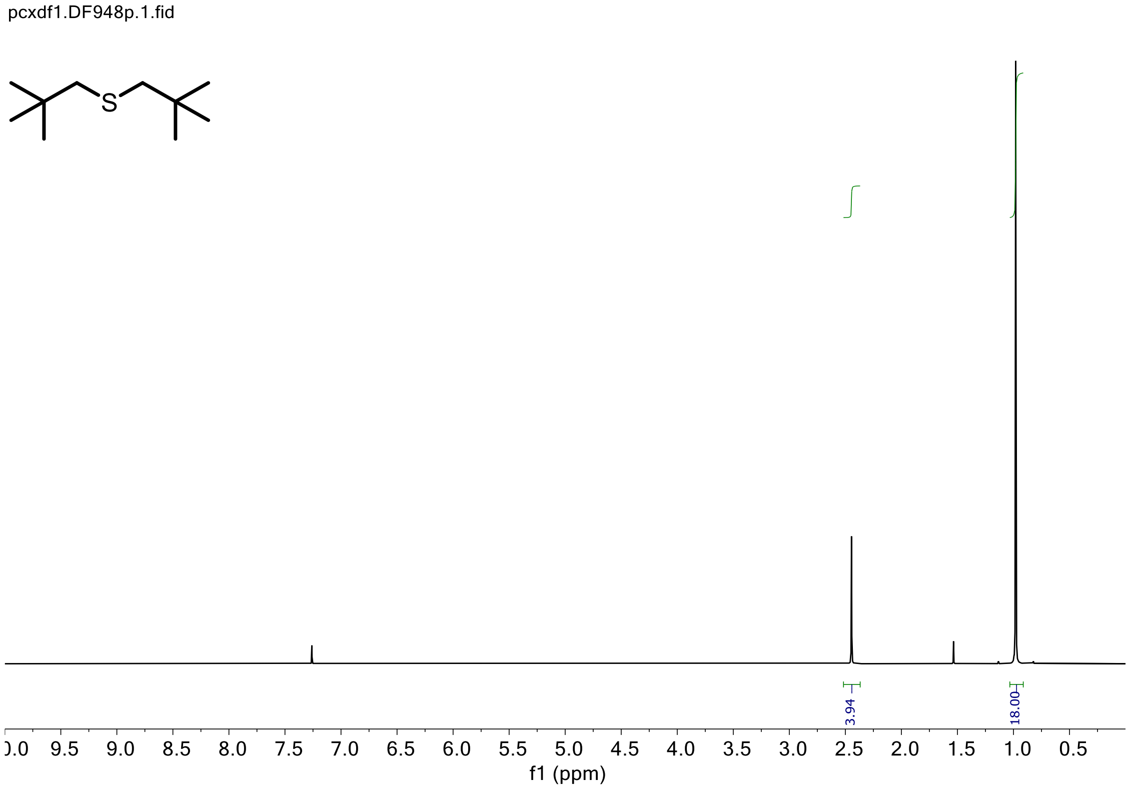

^{13}C-NMR ($CDCl_3$, 126 MHz)

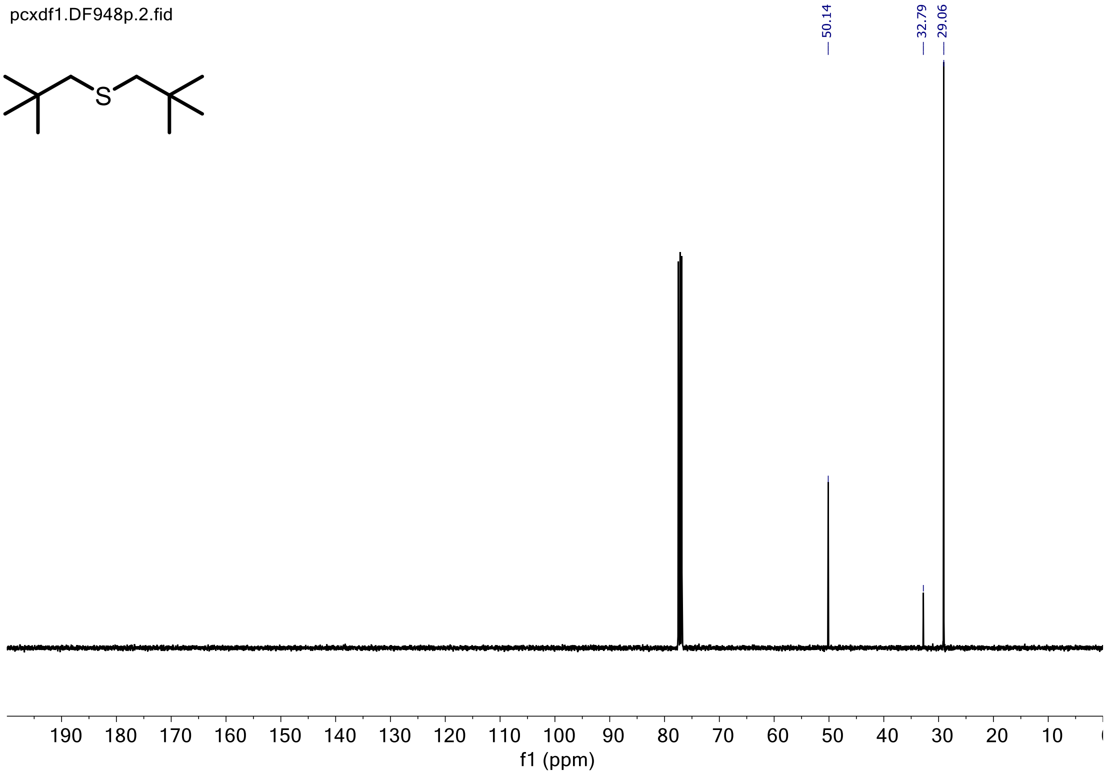

(2-bromoethyl)dineopentylsulfonium triflate

^{1}H-NMR ($CDCl_3$, 500 MHz)

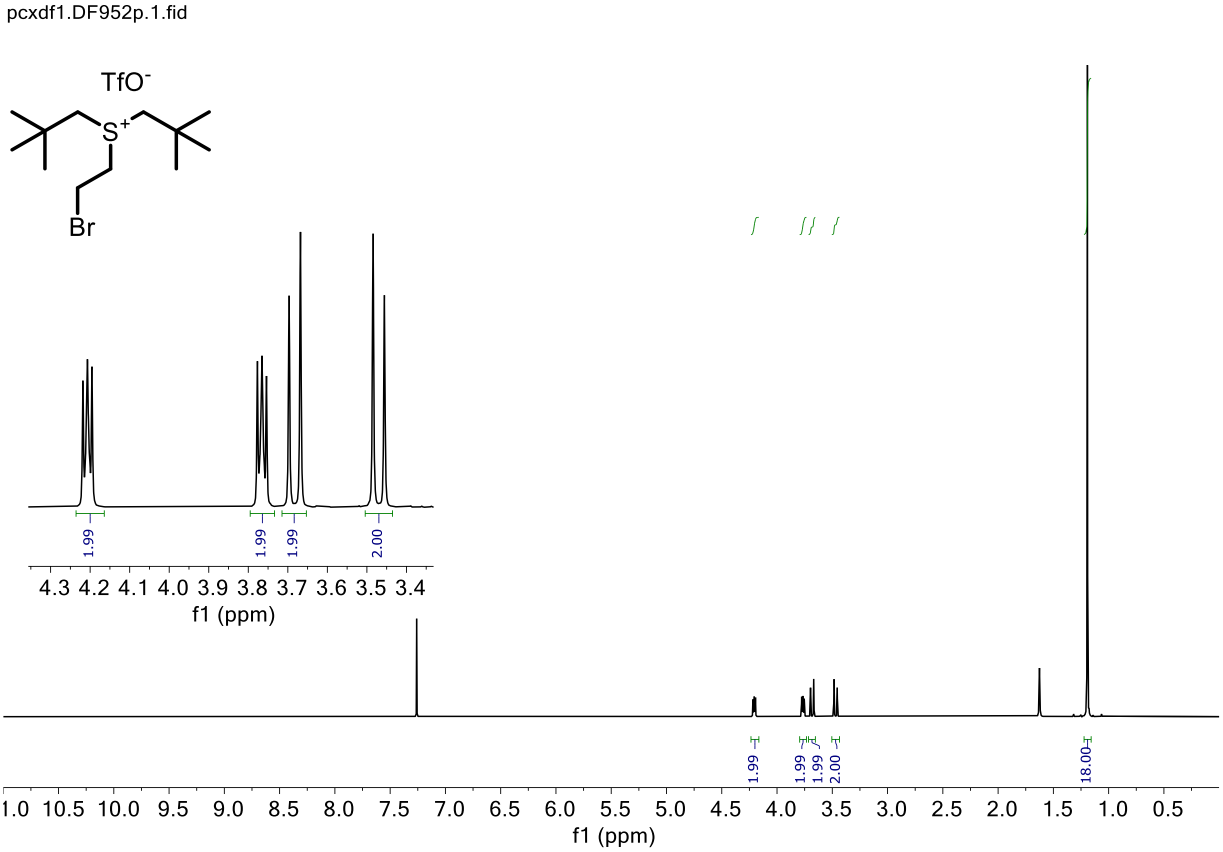

^{13}C-NMR ($CDCl_3$, 126 MHz)

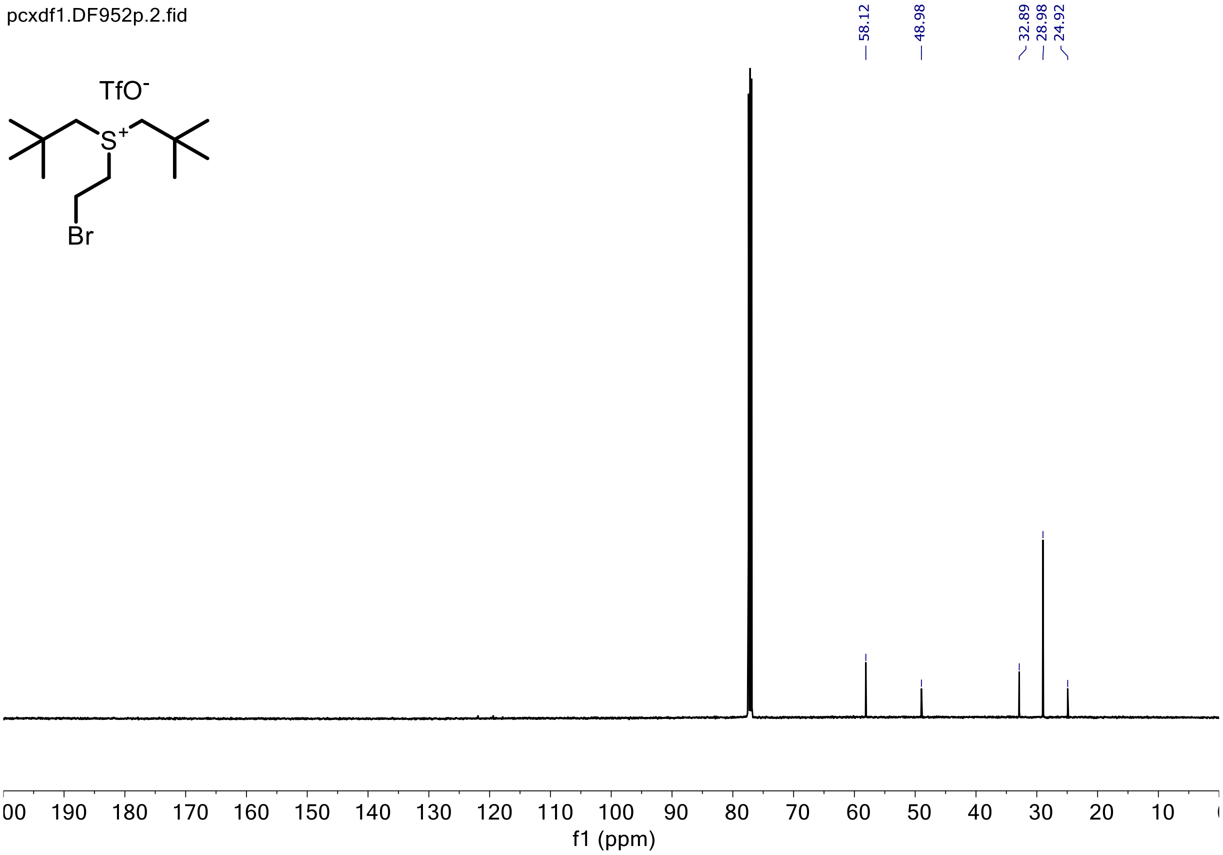

^{19}F-NMR ($CDCl_3$, 376 MHz)

pcxdf1.DF952p_19F.1.fid

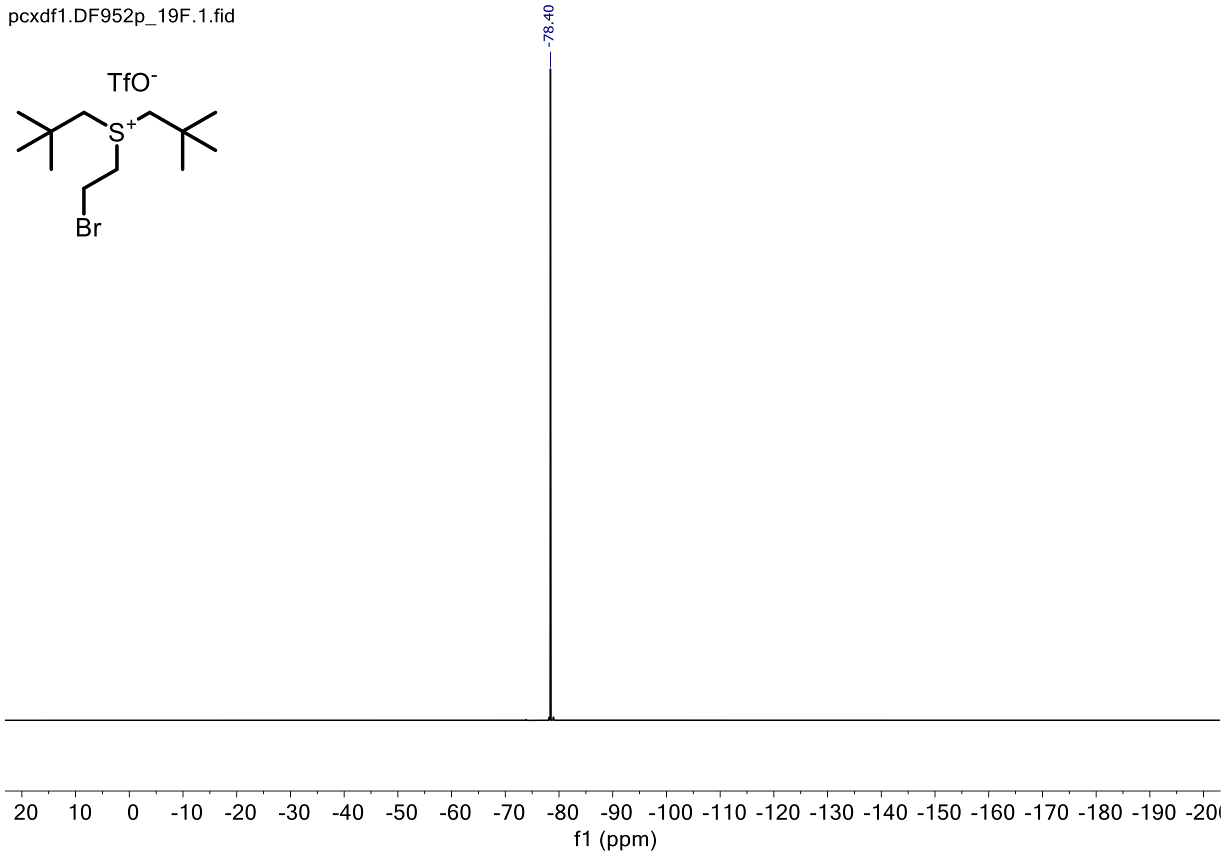

Dineopentyl(vinyl)sulfonium triflate (2b)

^{1}H-NMR ($CDCl_3$, 500 MHz)

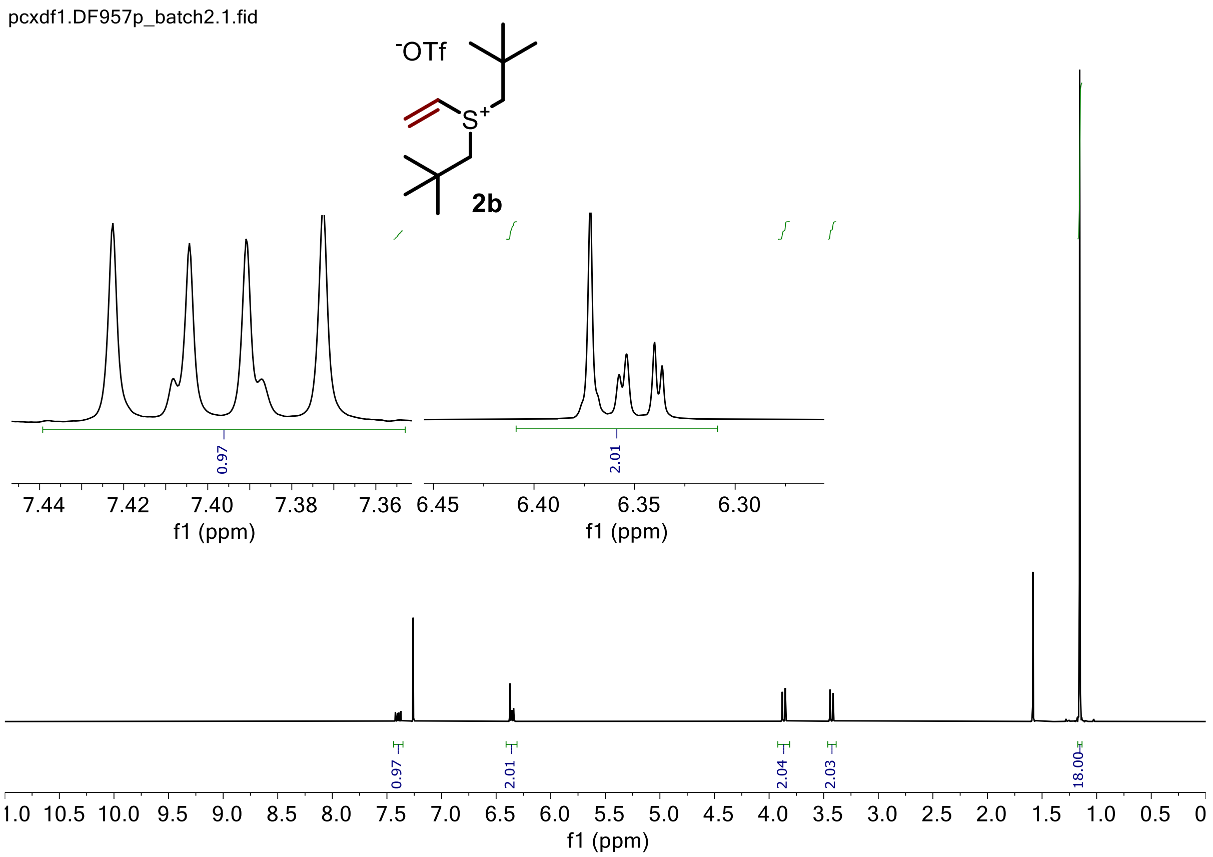

^{13}C-NMR ($CDCl_3$, 126 MHz)

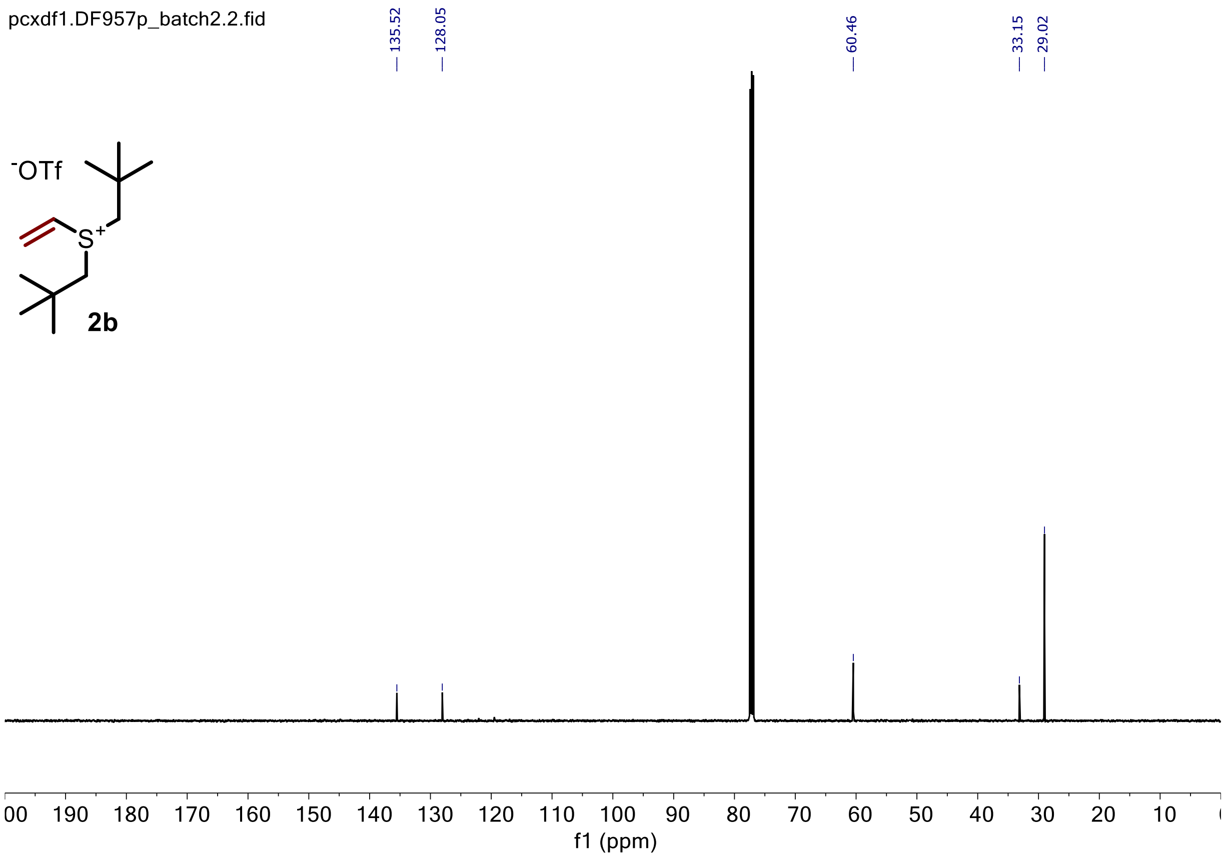

^{19}F-NMR ($CDCl_3$, 376 MHz)

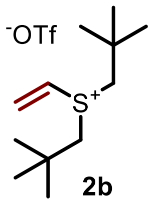

2b

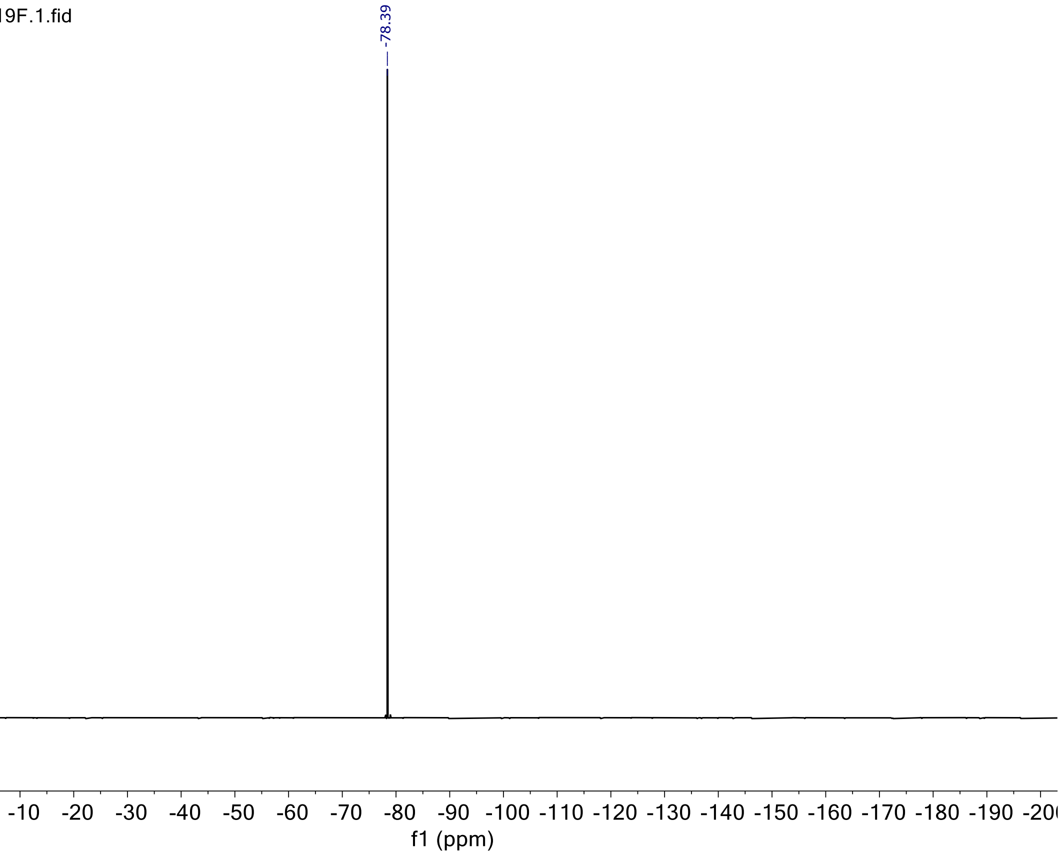

Dineopentyl(prop-1-en-1-yl)sulfonium triflate (2g)

^{1}H-NMR ($CDCl_3$, 400 MHz)

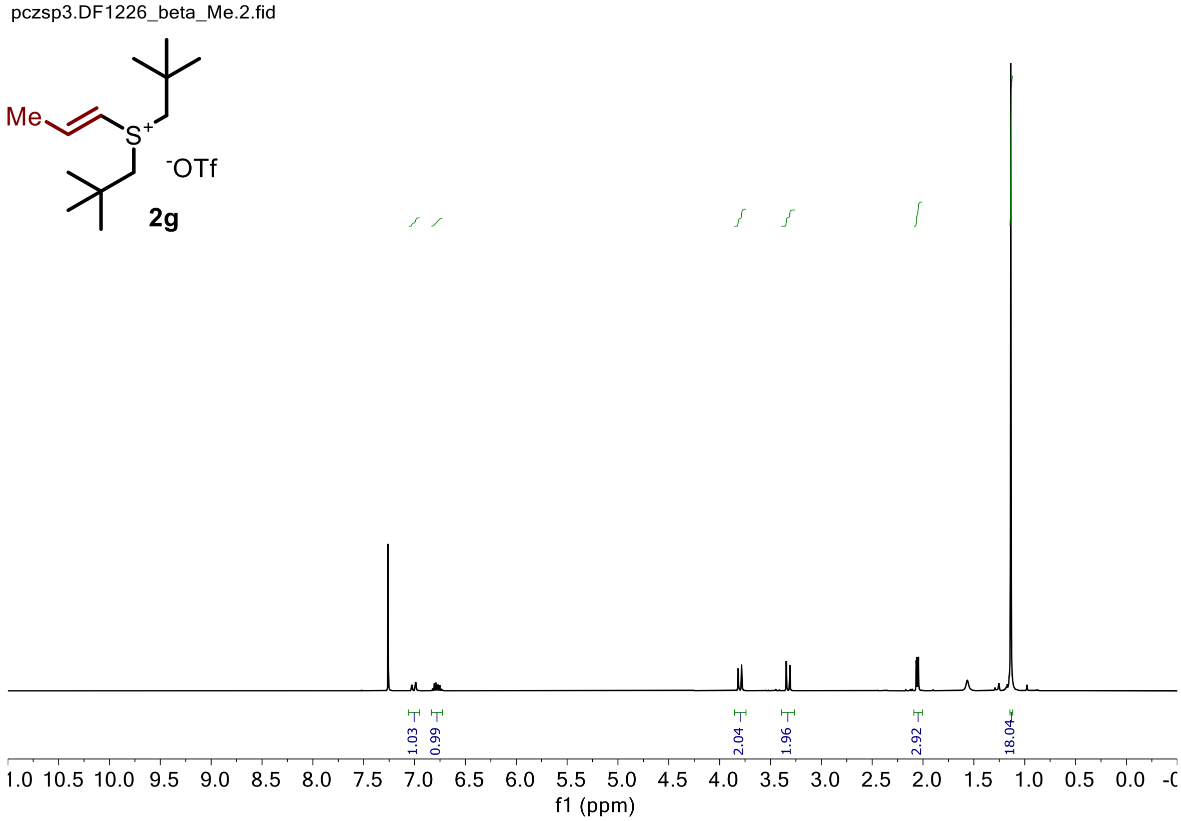

^{13}C-NMR ($CDCl_3$, 126 MHz)

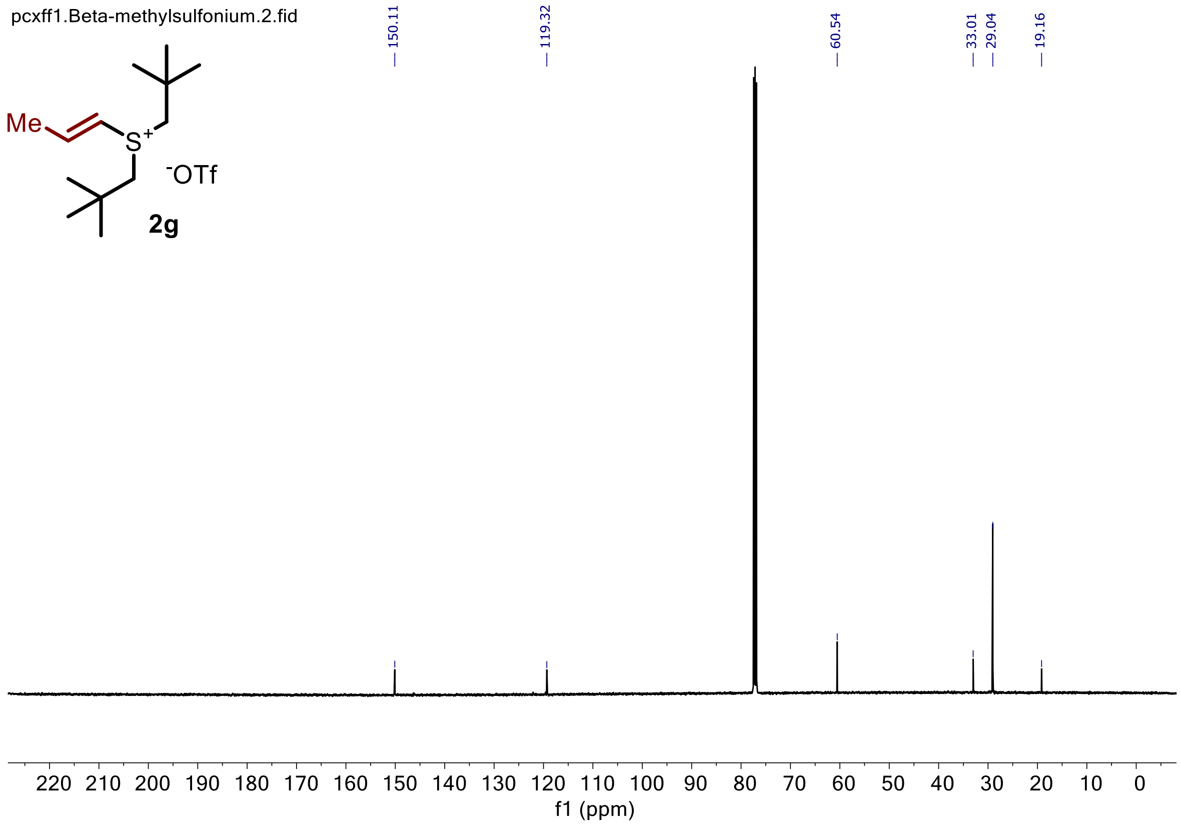

^{19}F-NMR (CDCl$_3$, 376 MHz)

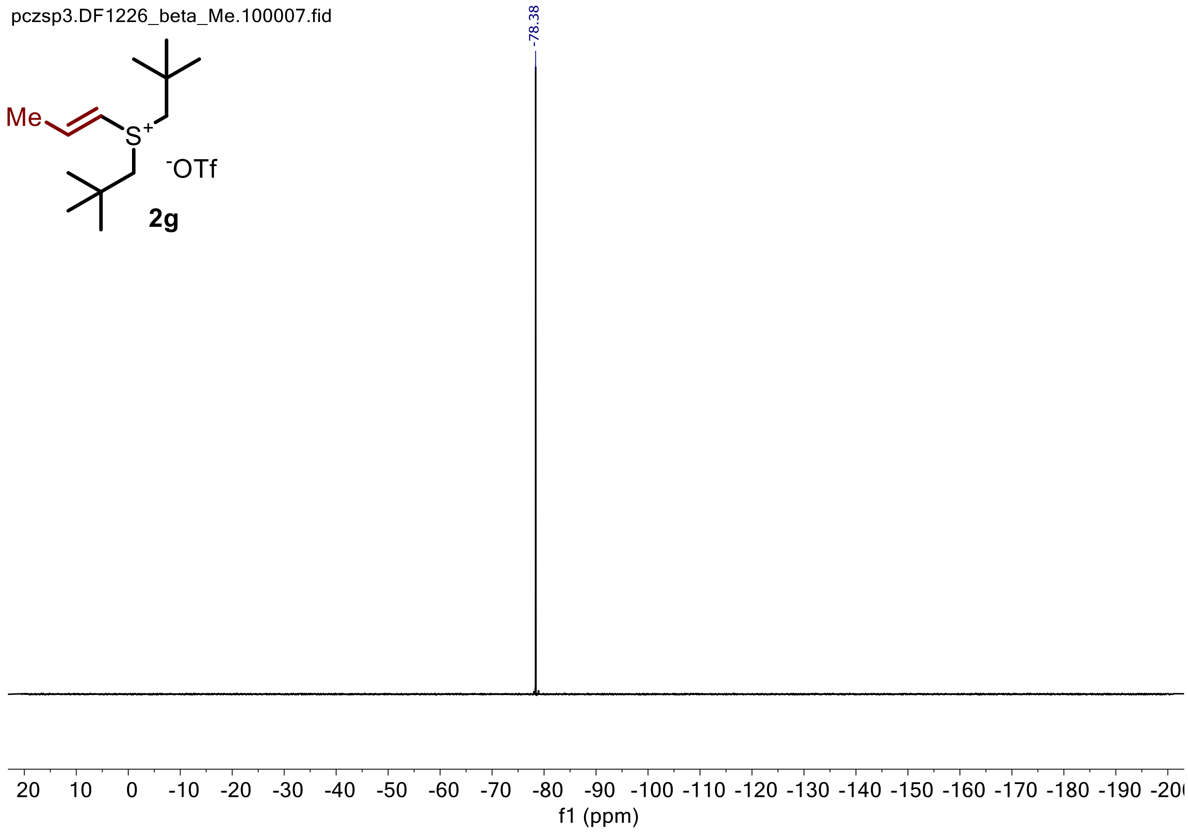

Dineopentyl(prop-1-en-2-yl)sulfonium triflate (2h)

^{1}H-NMR ($CDCl_3$, 400 MHz)

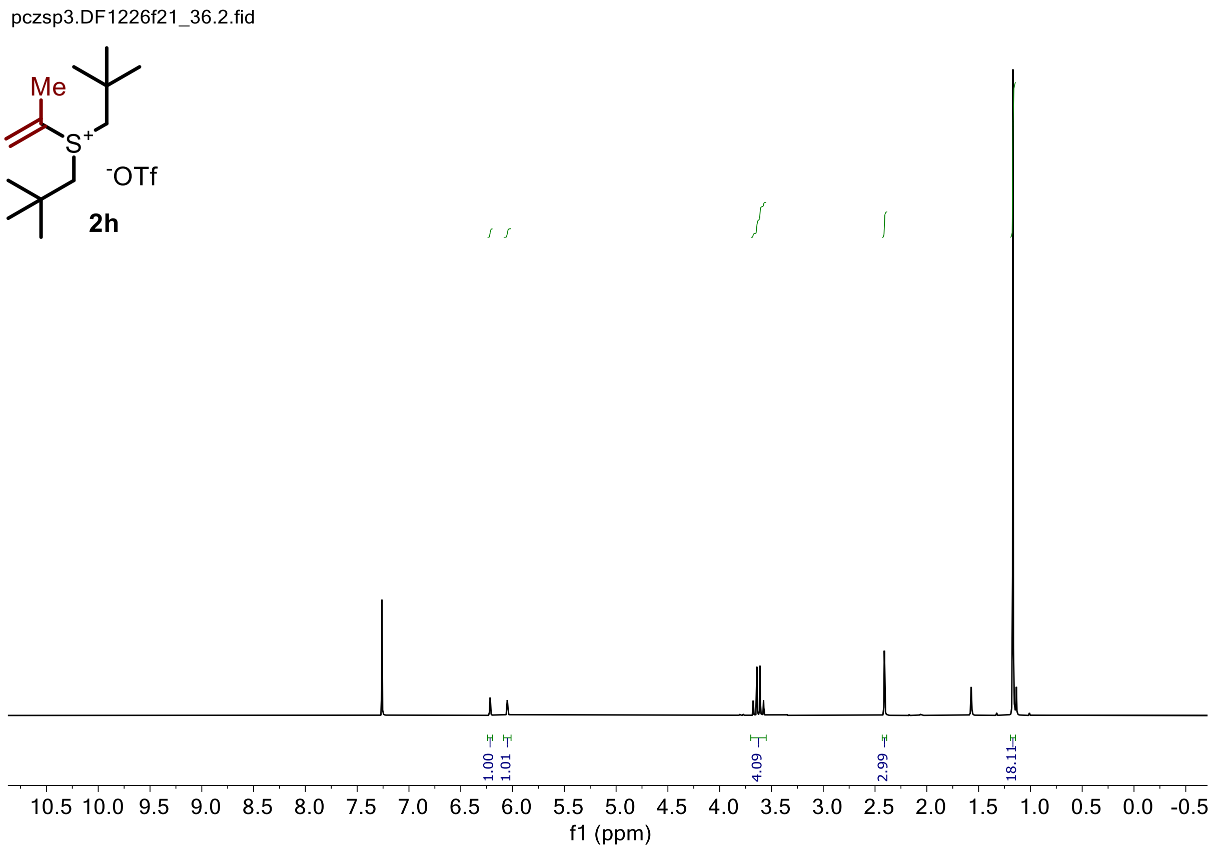

^{13}C-NMR ($CDCl_3$, 101 MHz)

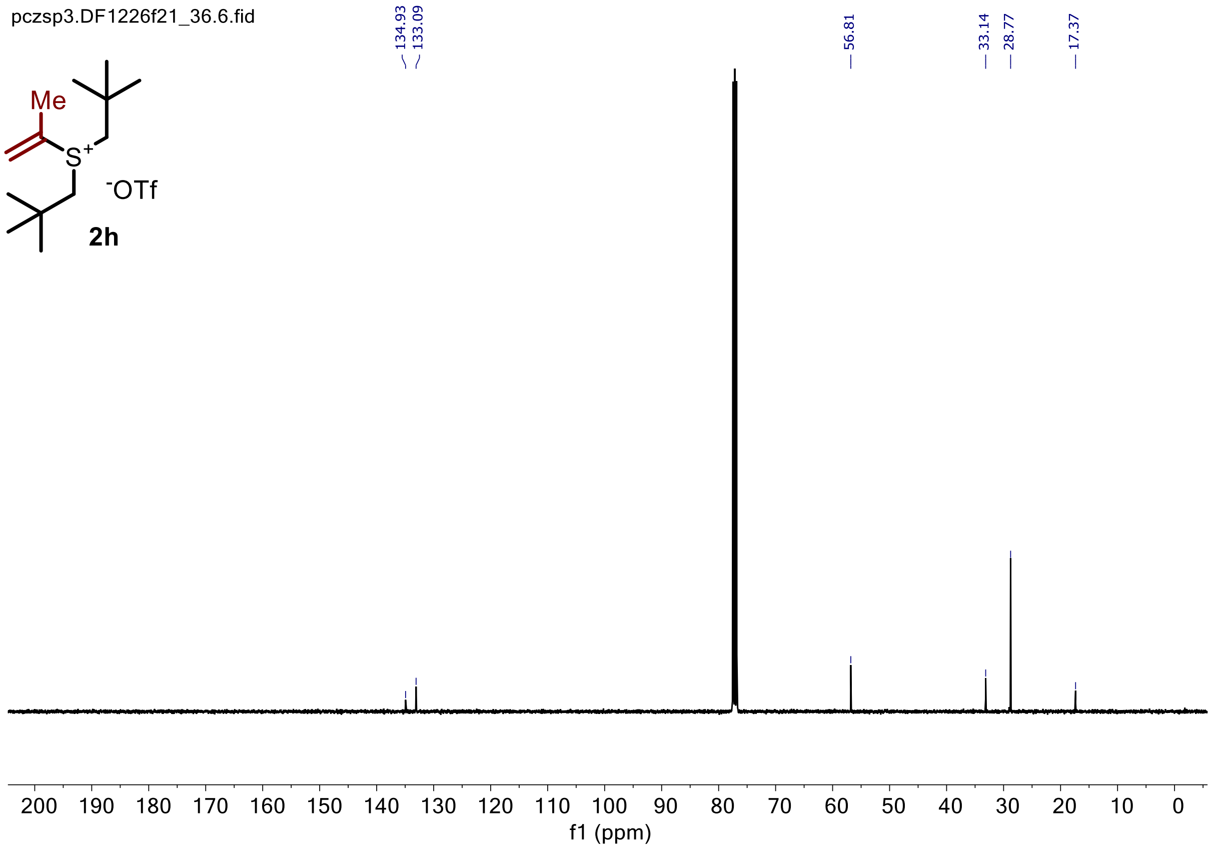

^{19}F-NMR ($CDCl_3$, 376 MHz)

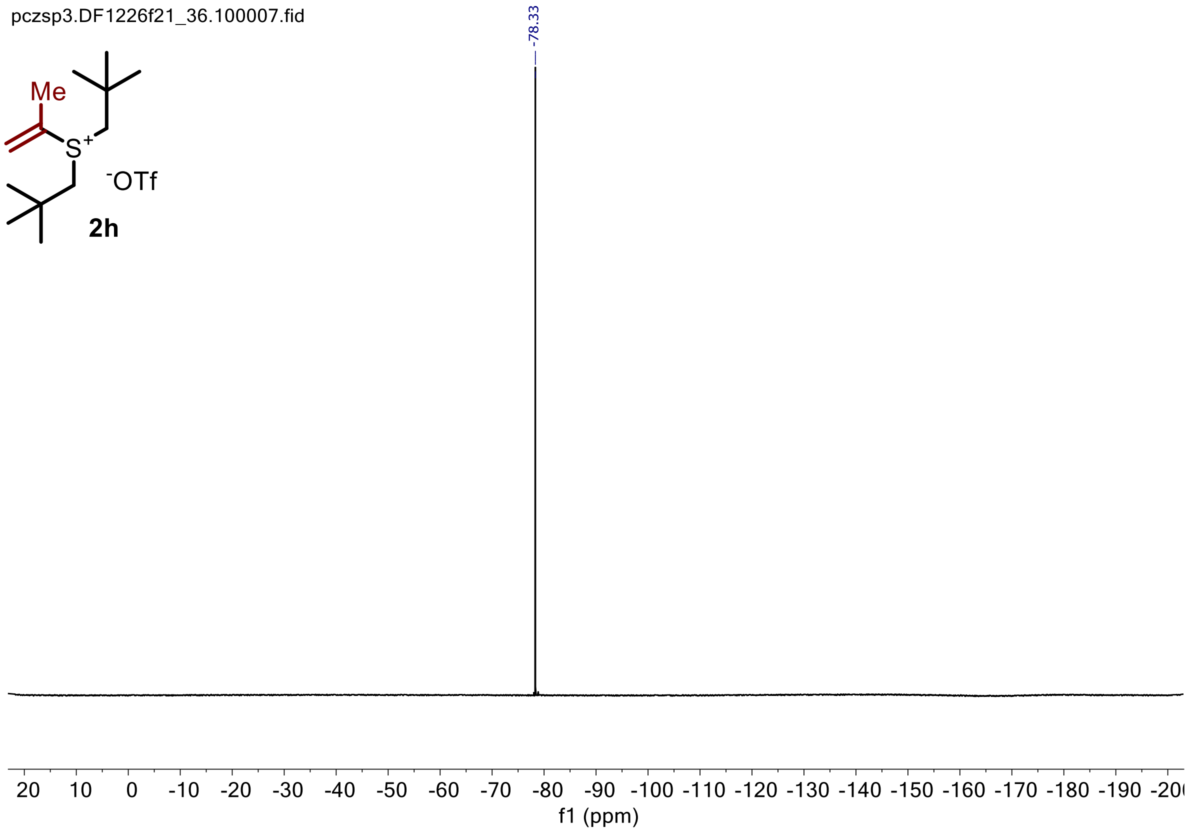

2h

Dineopentyl(1-phenylvinyl)sulfonium tetrafluoroborate (2i)

^{1}H-NMR ($CDCl_3$, 500 MHz)

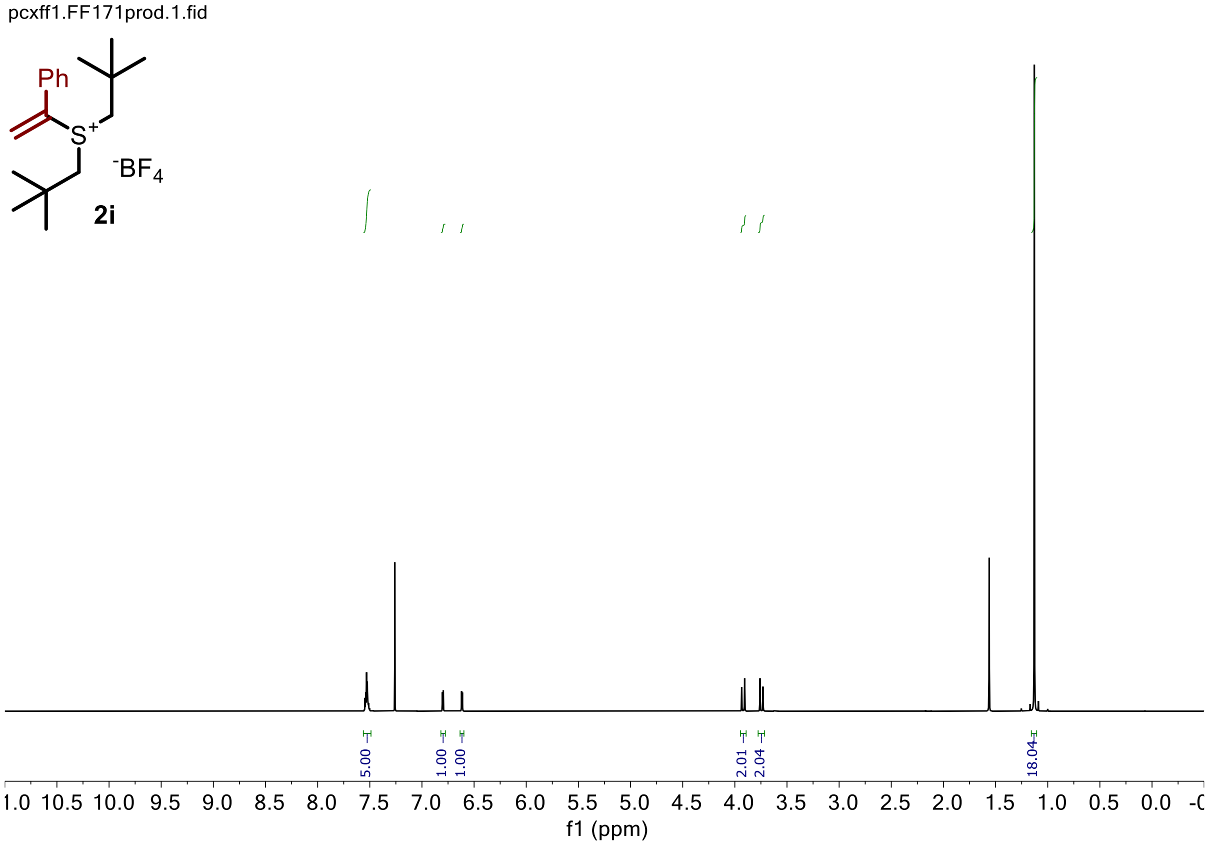

^{13}C-NMR ($CDCl_3$, 101 MHz)

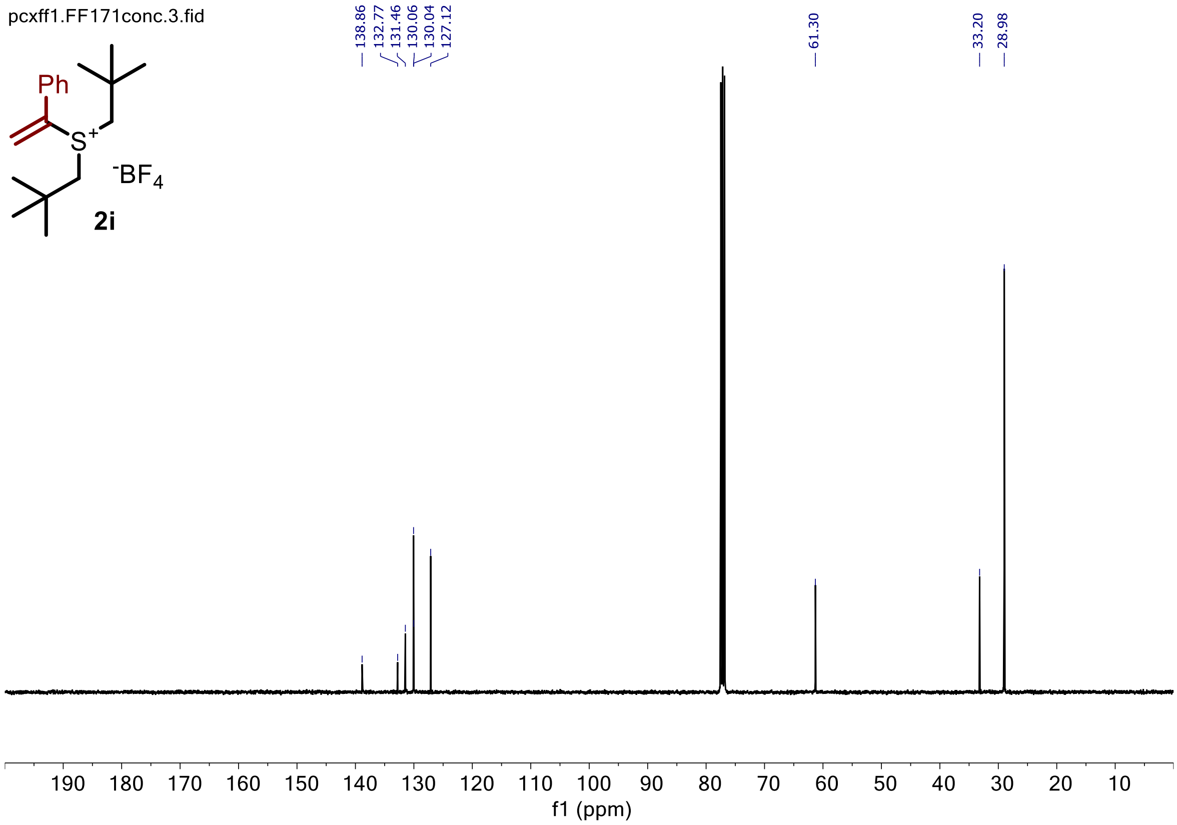

^{19}F-NMR ($CDCl_3$, 376 MHz)

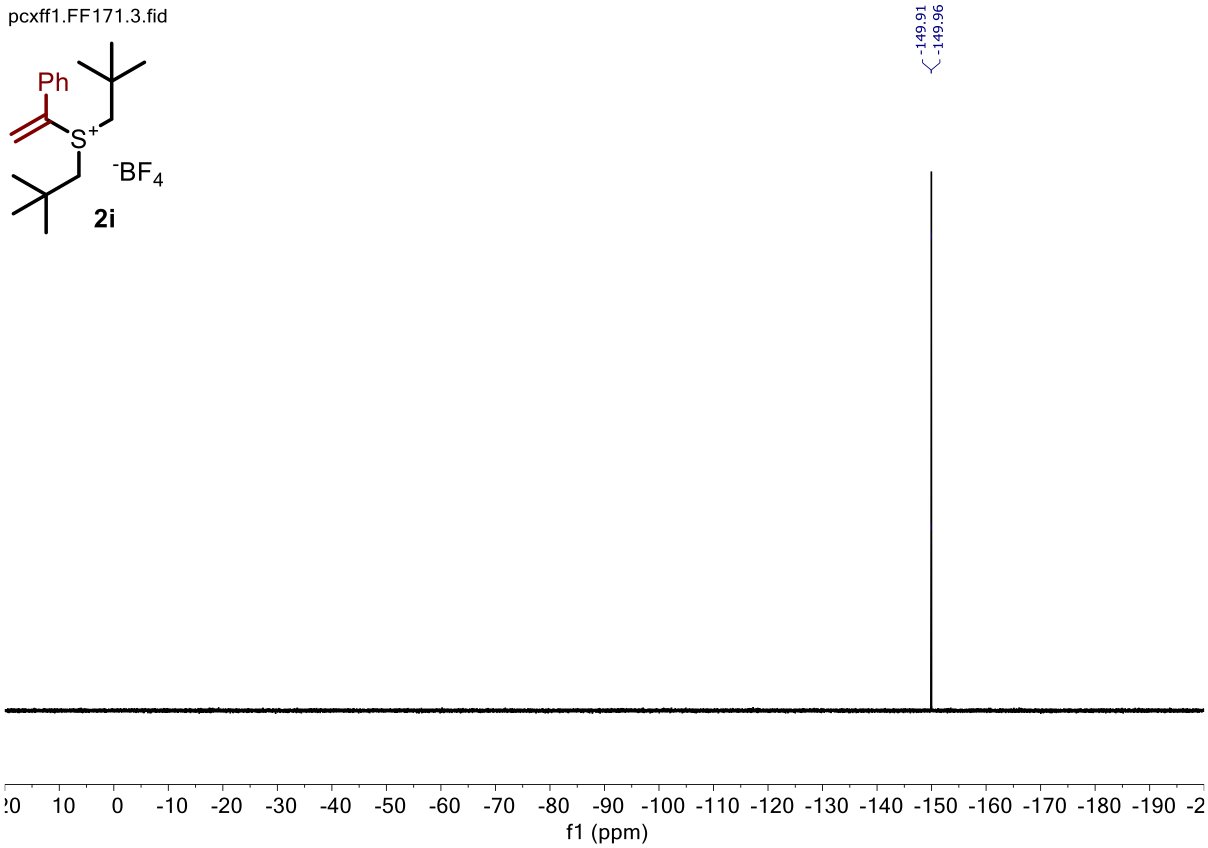

2-hydroxy-7-azaspiro[3.5]nonan-7-ium chloride

^{1}H-NMR (CD_3OD, 500 MHz)

pczsp3.SP732.1.fid

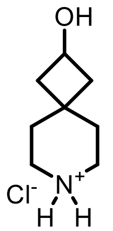

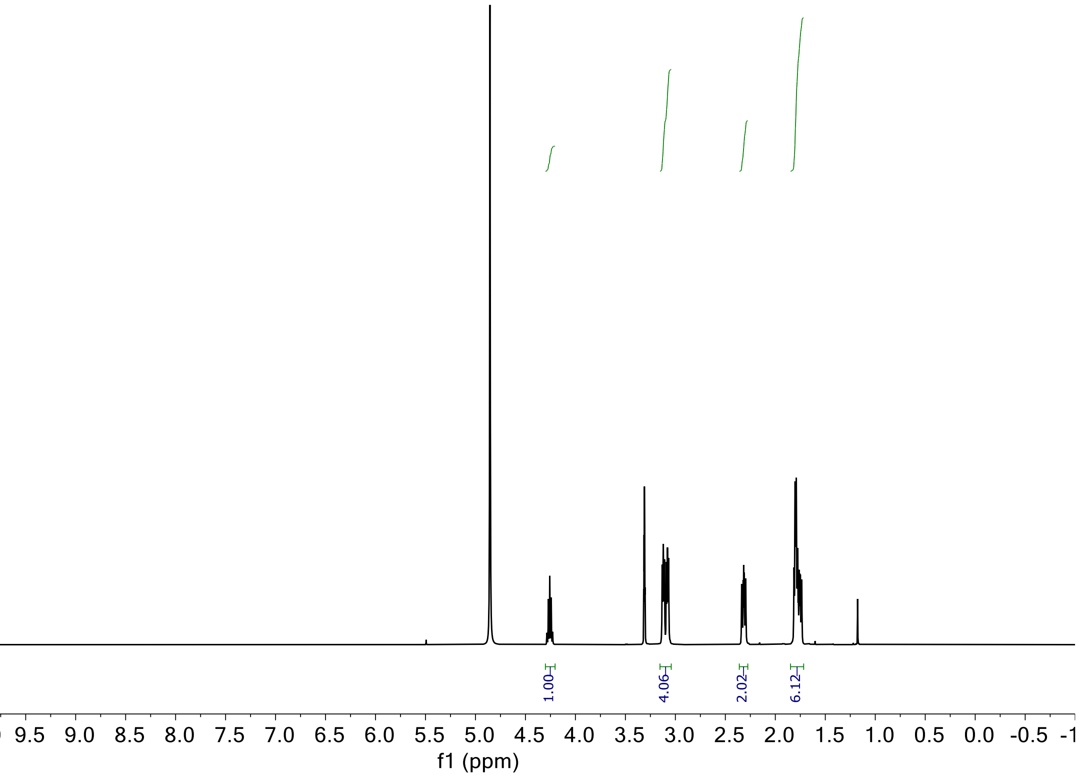

^{13}C-NMR (CD_3OD, 126 MHz)

pczsp3.SP732.6.fid

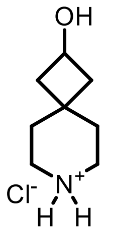

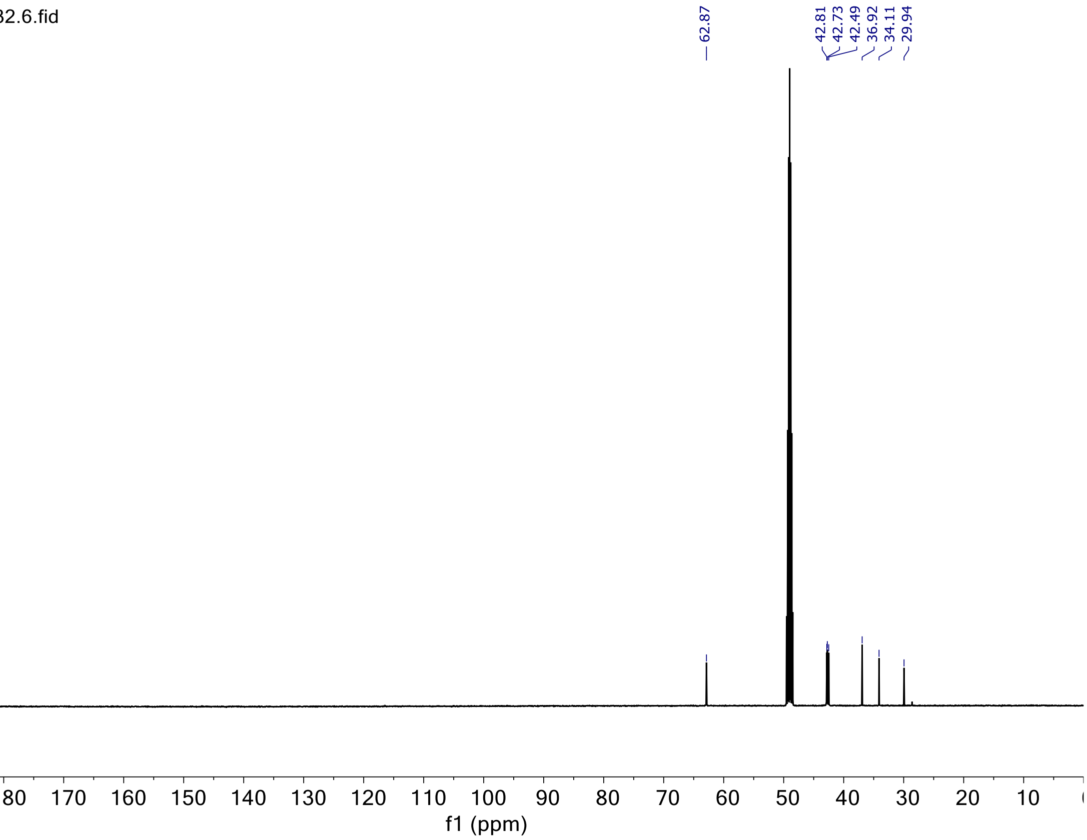

(2-hydroxy-7-azaspiro[3.5]nonan-7-yl)(phenyl)methanone (1k)

^{1}H-NMR ($CDCl_3$, 400 MHz)

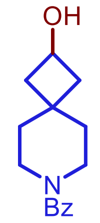

1k

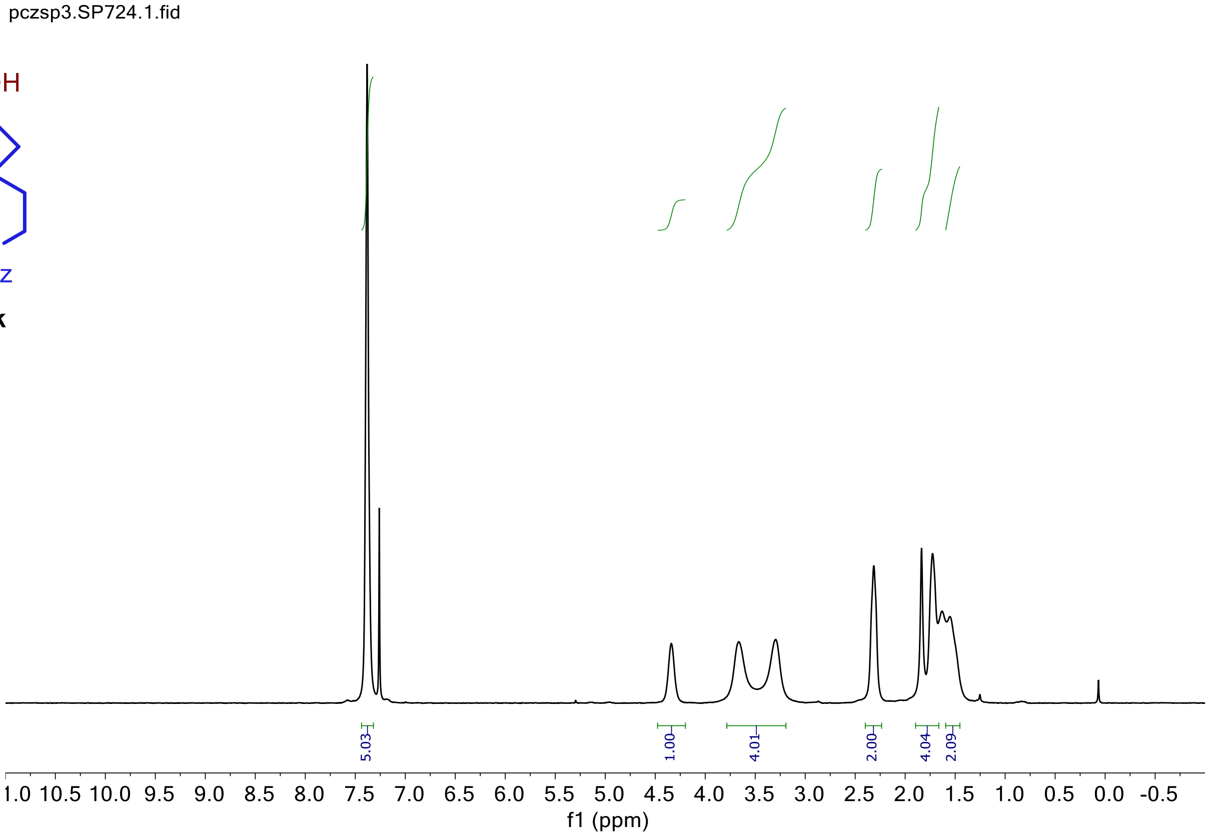

^{13}C-NMR ($CDCl_3$, 126 MHz)

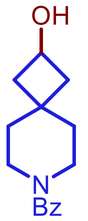

1k

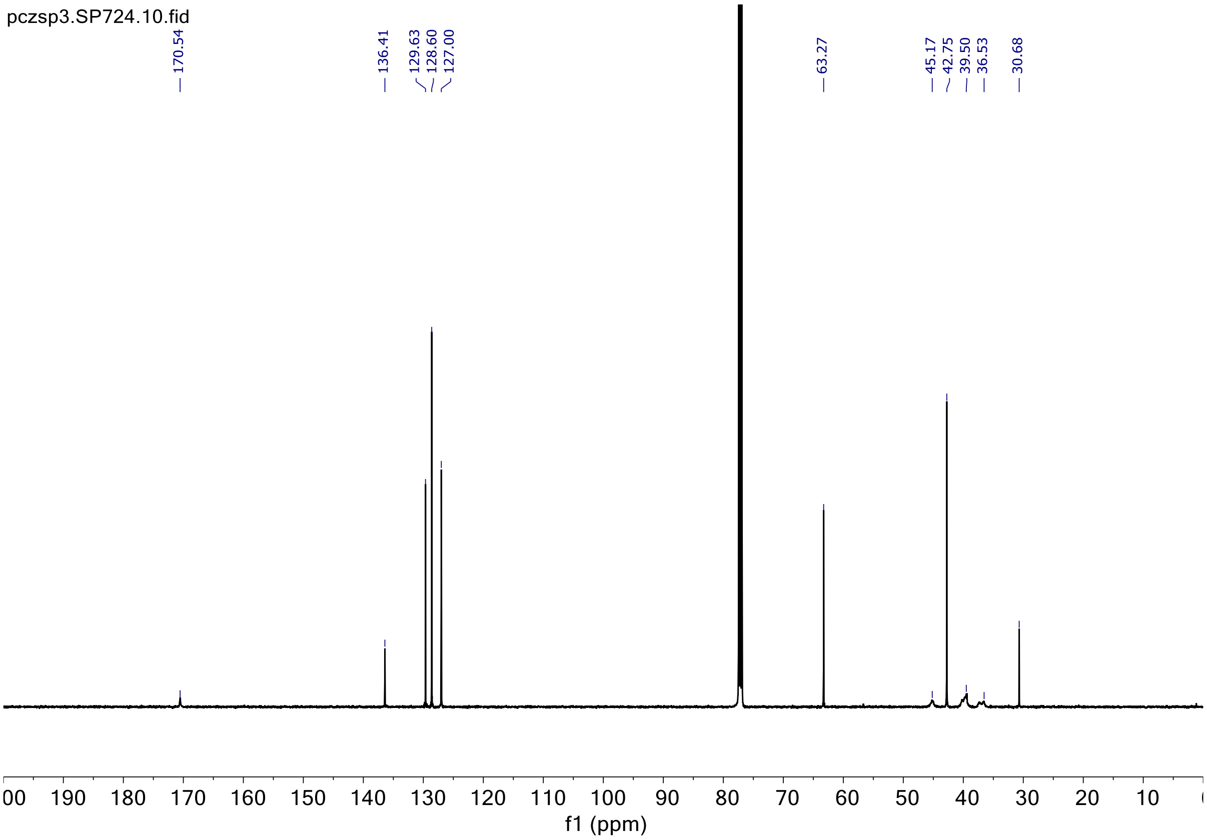

6-hydroxy-2-azaspiro[3.3]heptan-2-yl)(phenyl)methanone (1j)

^{1}H-NMR ($CDCl_3$, 500 MHz)

pcxff1.FGF96col.1.fid

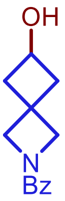

1j

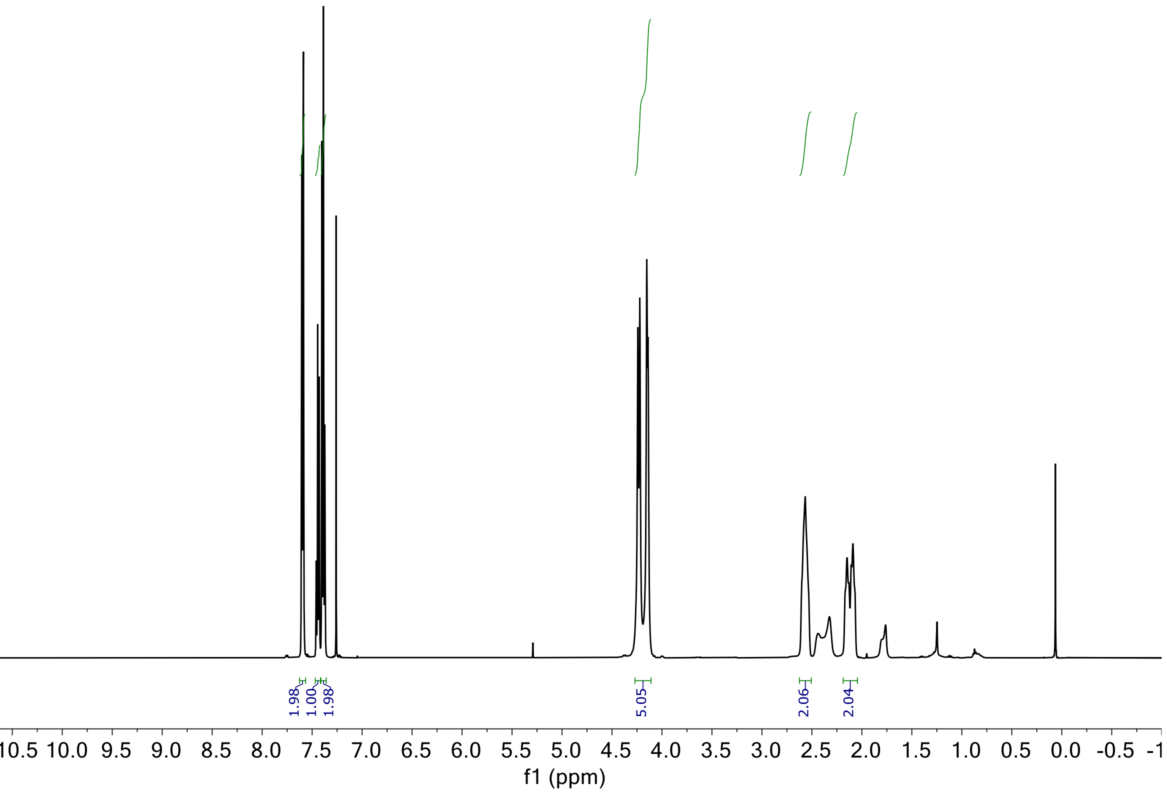

^{13}C-NMR ($CDCl_3$, 126 MHz)

pcxff1.FGF96col.2.fid

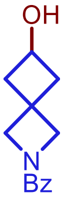

1j

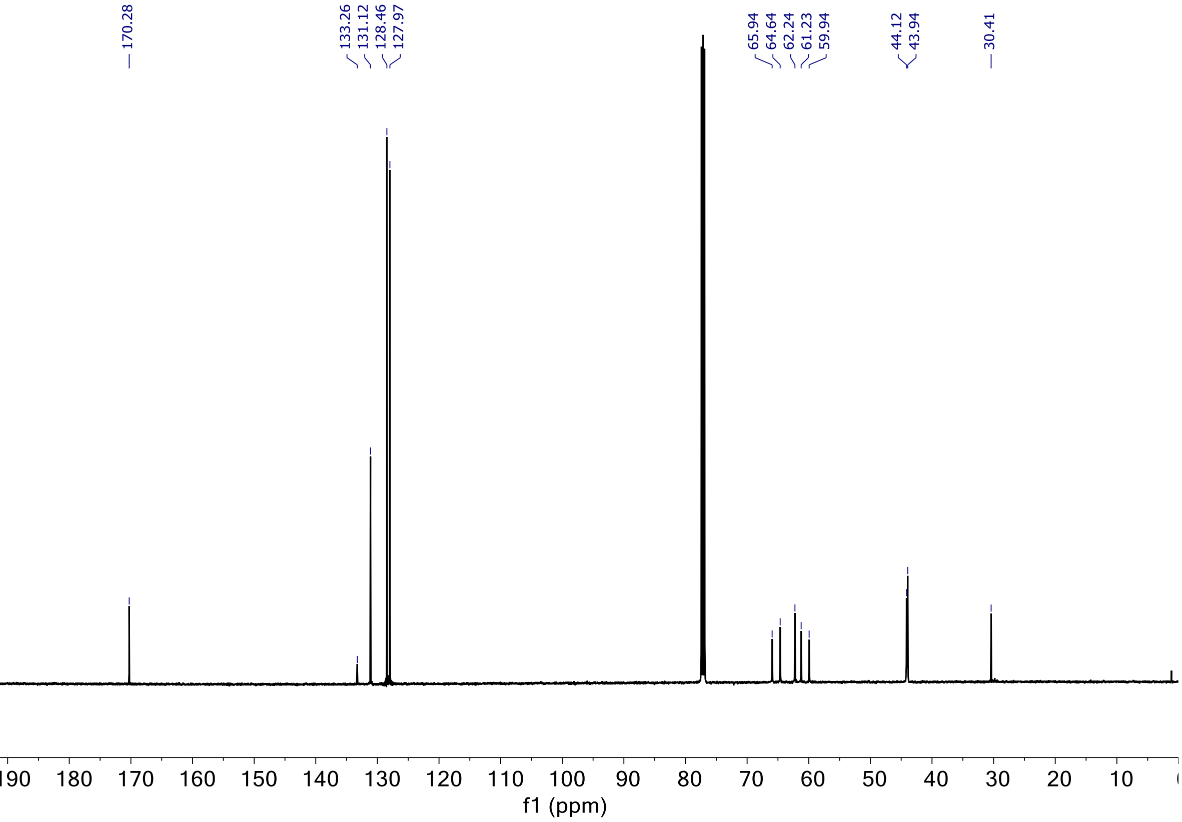

17-bromoheptadecan-7-ol

^{1}H-NMR (CDCl$_3$, 500 MHz)

pcxff1.FGF25-frac26-56.1.fid

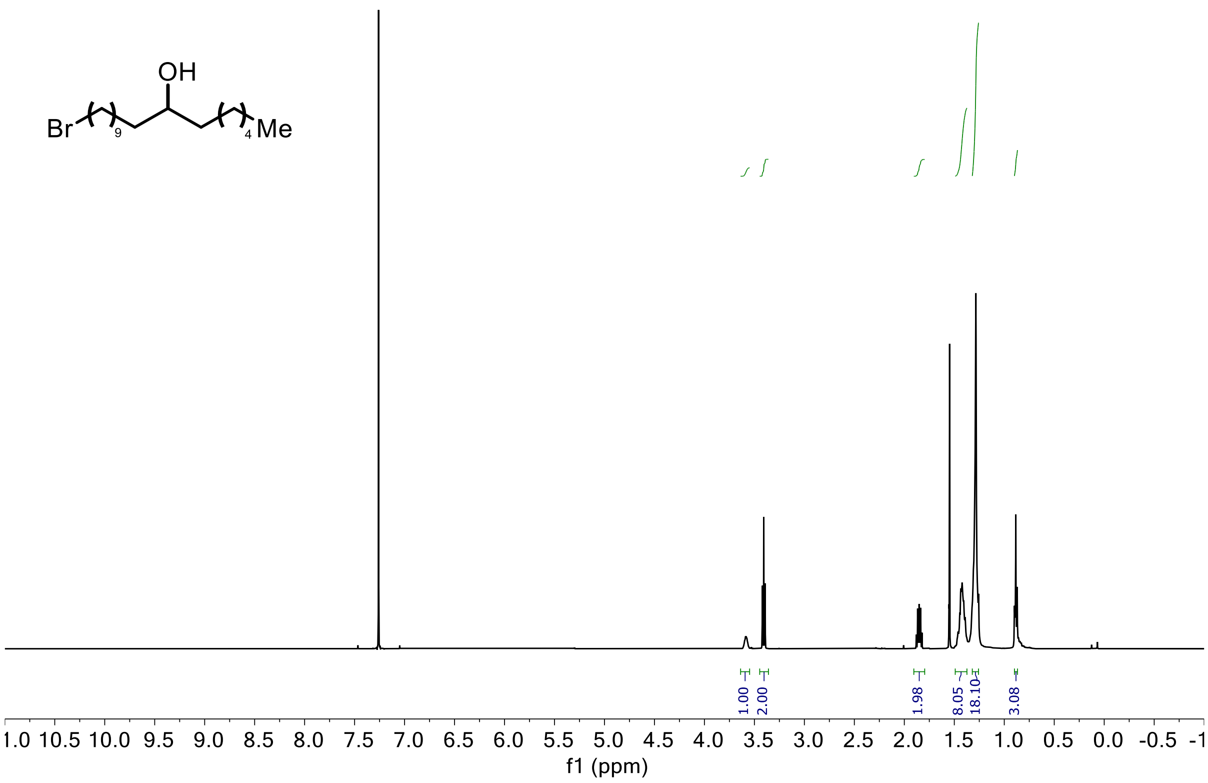

^{13}C-NMR (CDCl$_3$, 101 MHz)

pcxff1.FGF25.2.fid

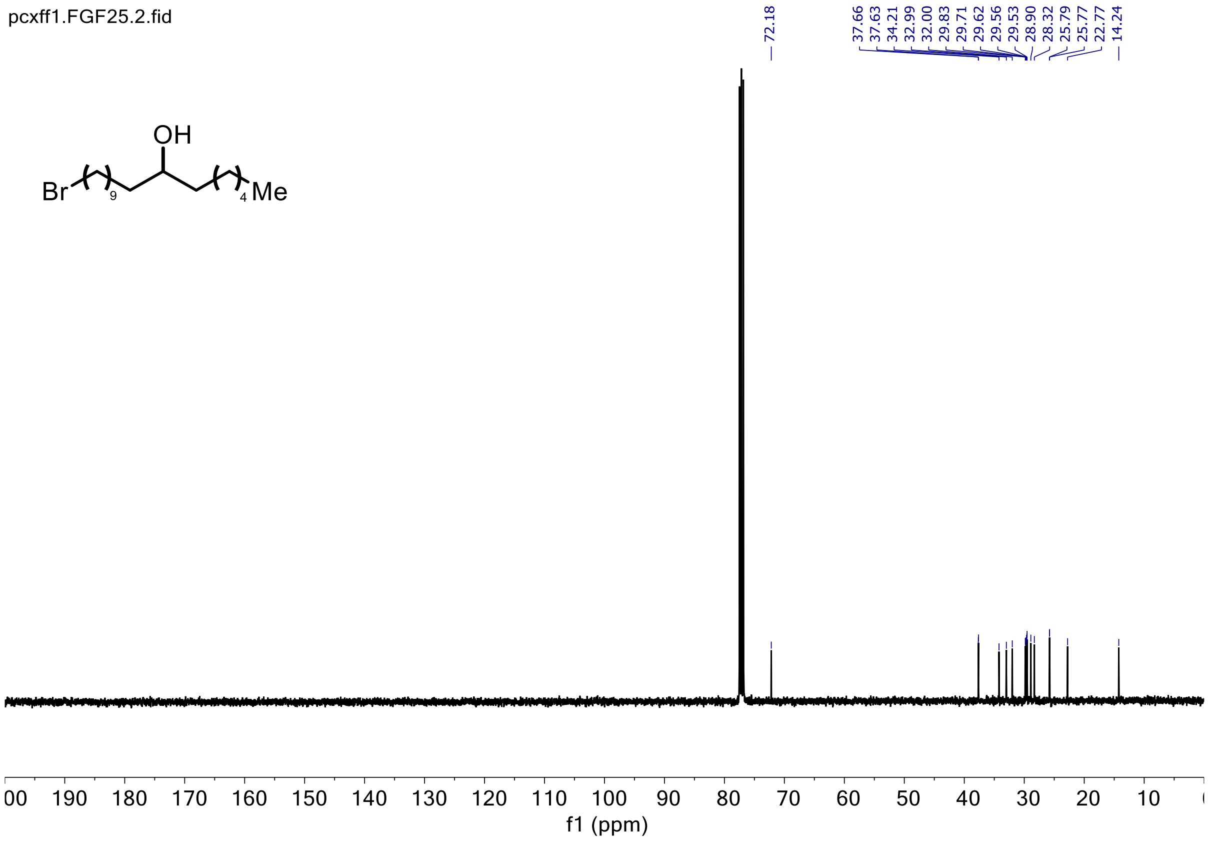

12-hydroxyoctadecanenitrile (1r)

^{1}H-NMR ($CDCl_3$, 500 MHz)

pcxff1.FGF98column.1.fid

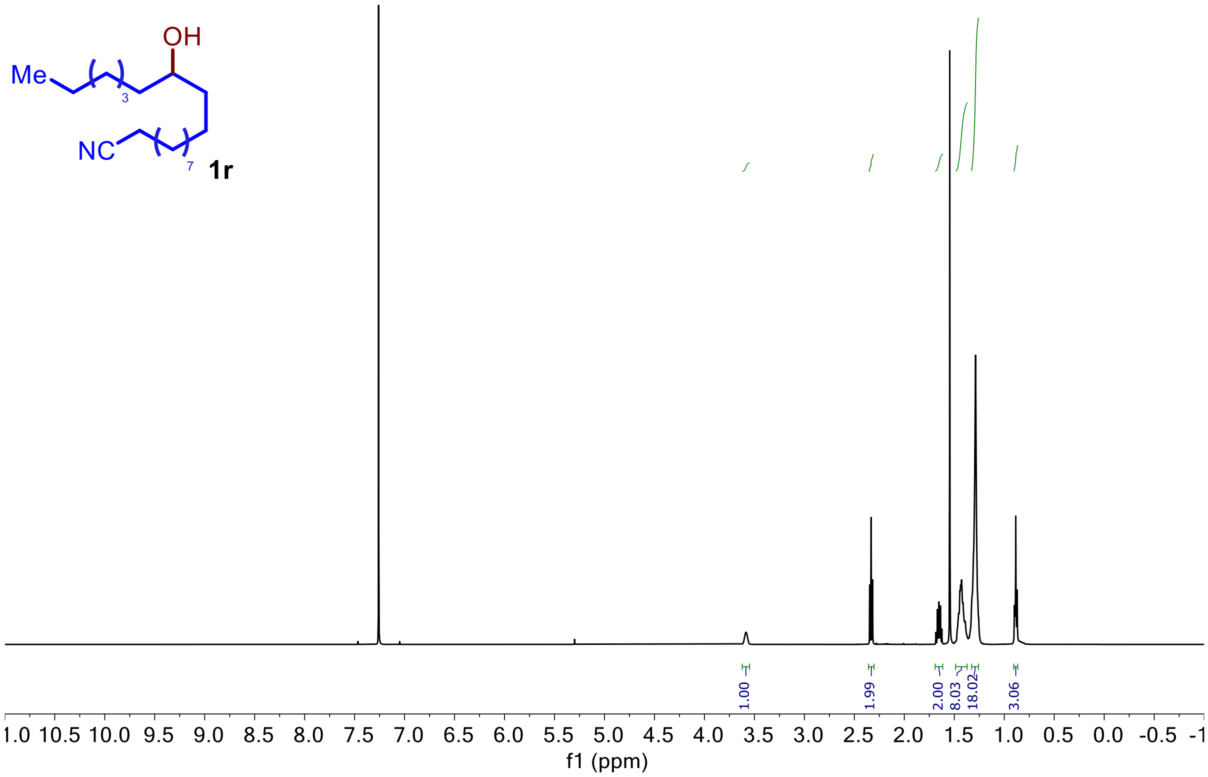

^{13}C-NMR ($CDCl_3$, 126 MHz)

pcxff1.FGF98column.2.fid

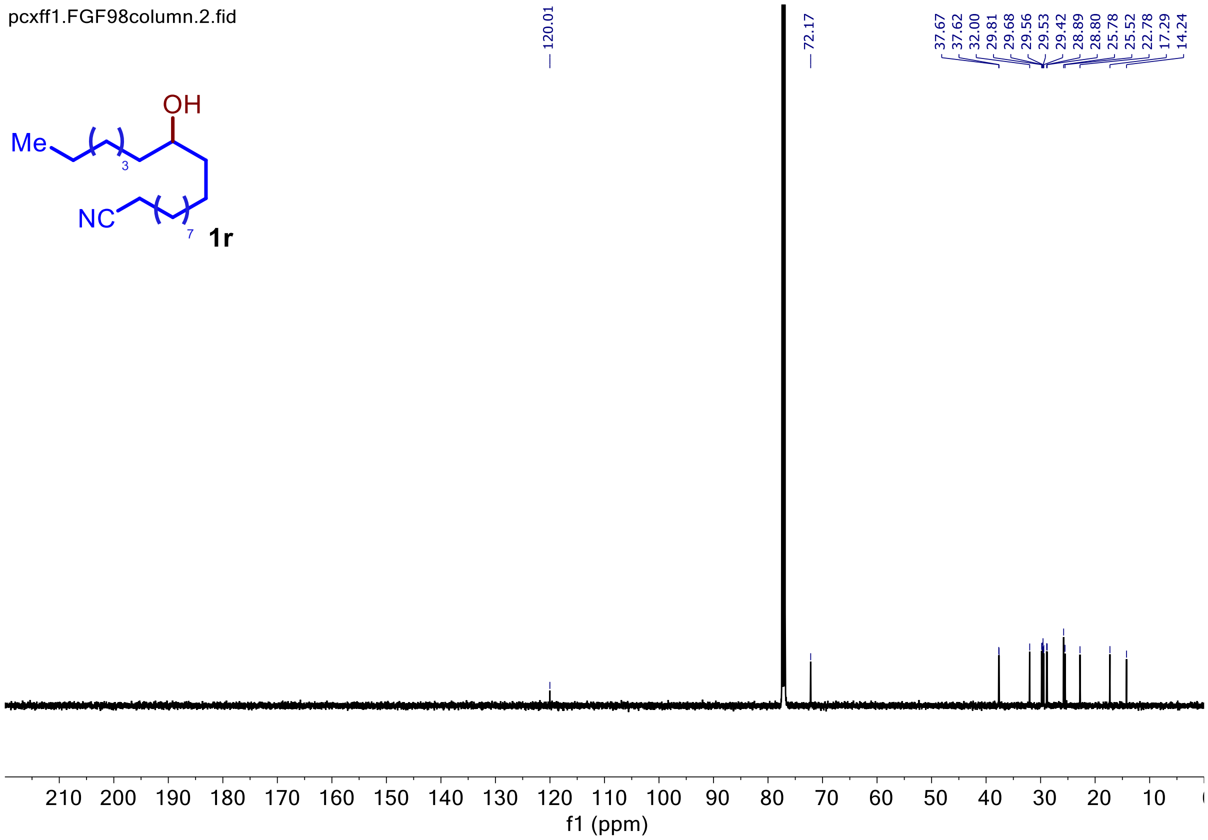

6-((*tert*-butyldiphenylsilyl)oxy)hexan-2-ol (1q)

^{1}H-NMR ($CDCl_3$, 400 MHz)

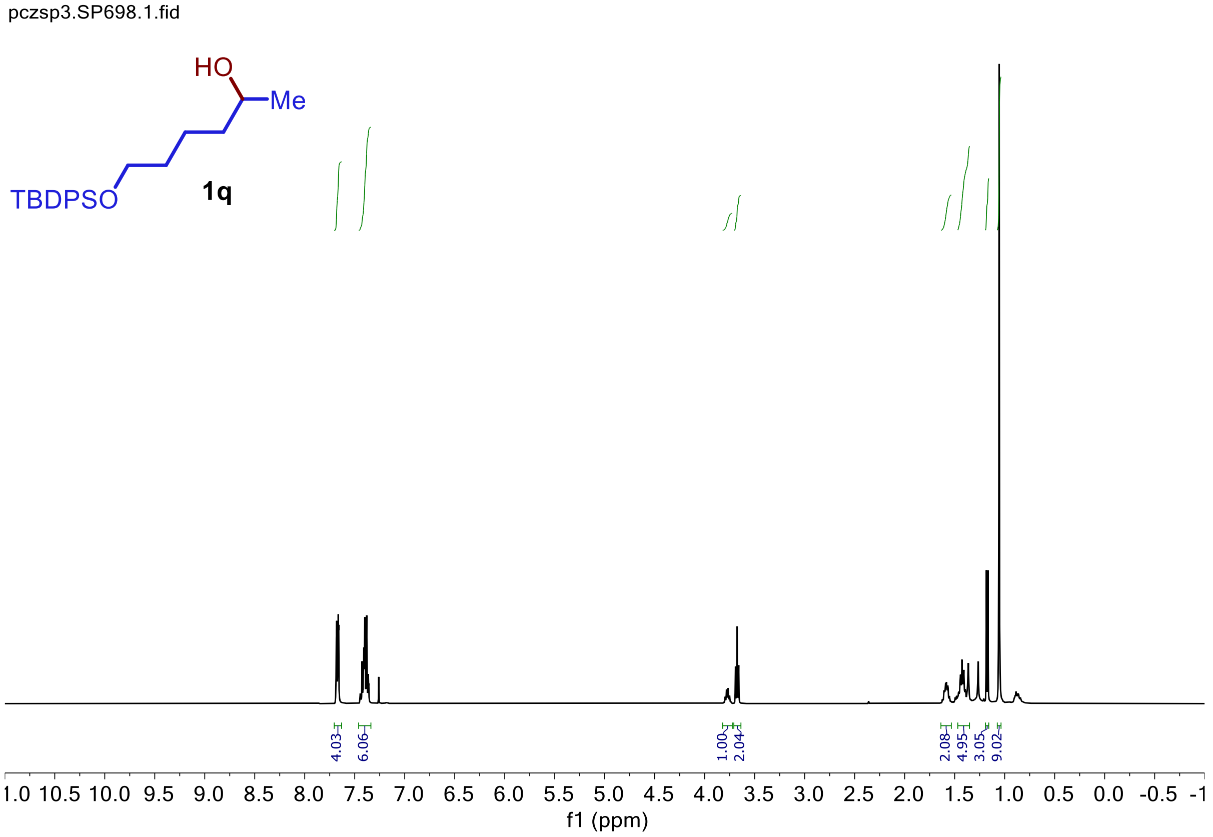

^{13}C-NMR ($CDCl_3$, 126 MHz)

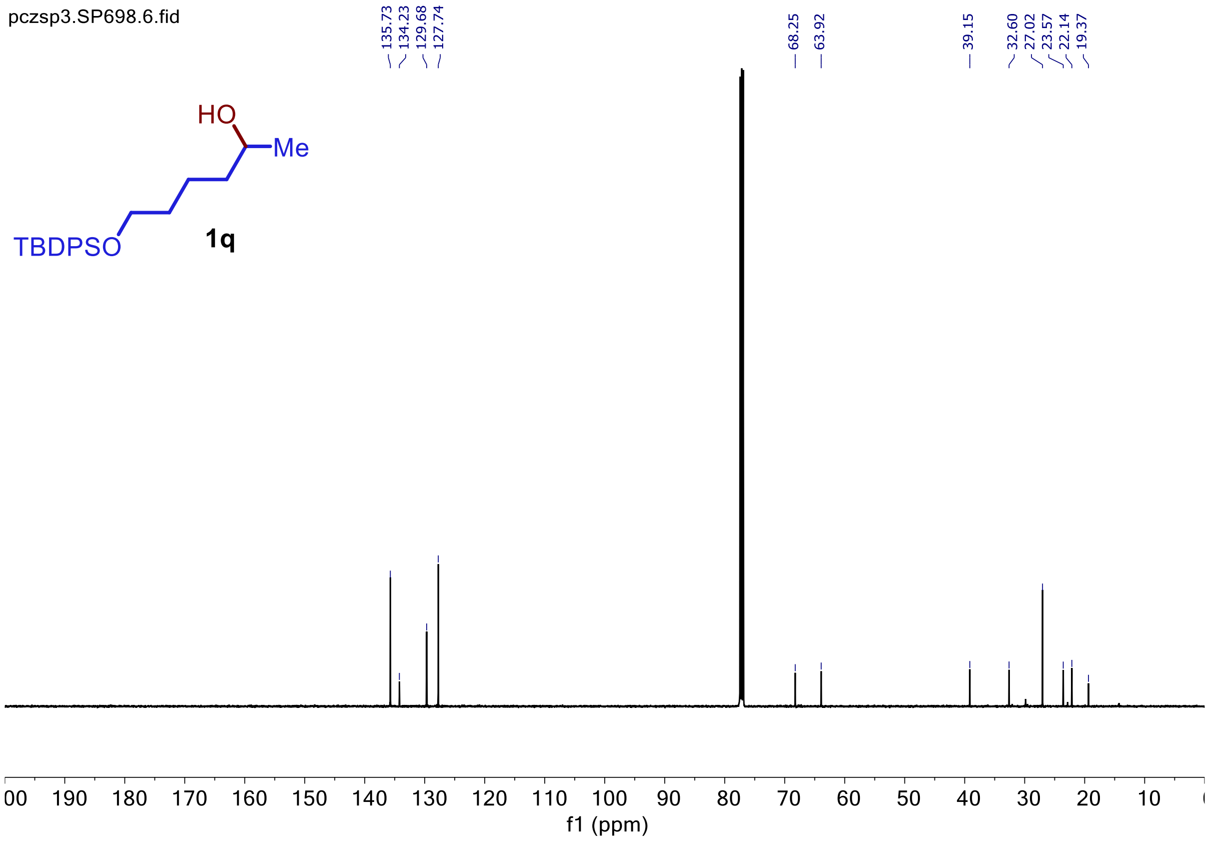

1-oxaspiro[3.5]nonane (3a)

^{1}H-NMR ($CDCl_3$, 400 MHz)

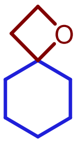

3a

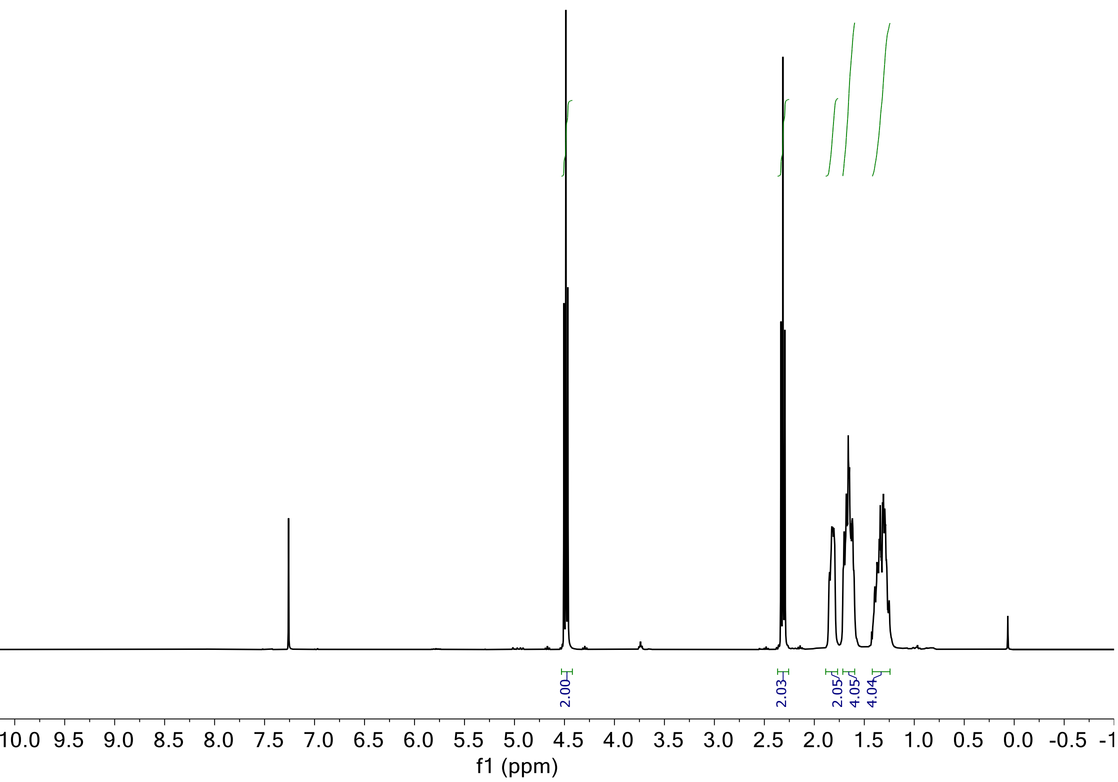

^{13}C-NMR ($CDCl_3$, 126 MHz)

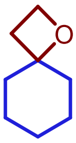

3a

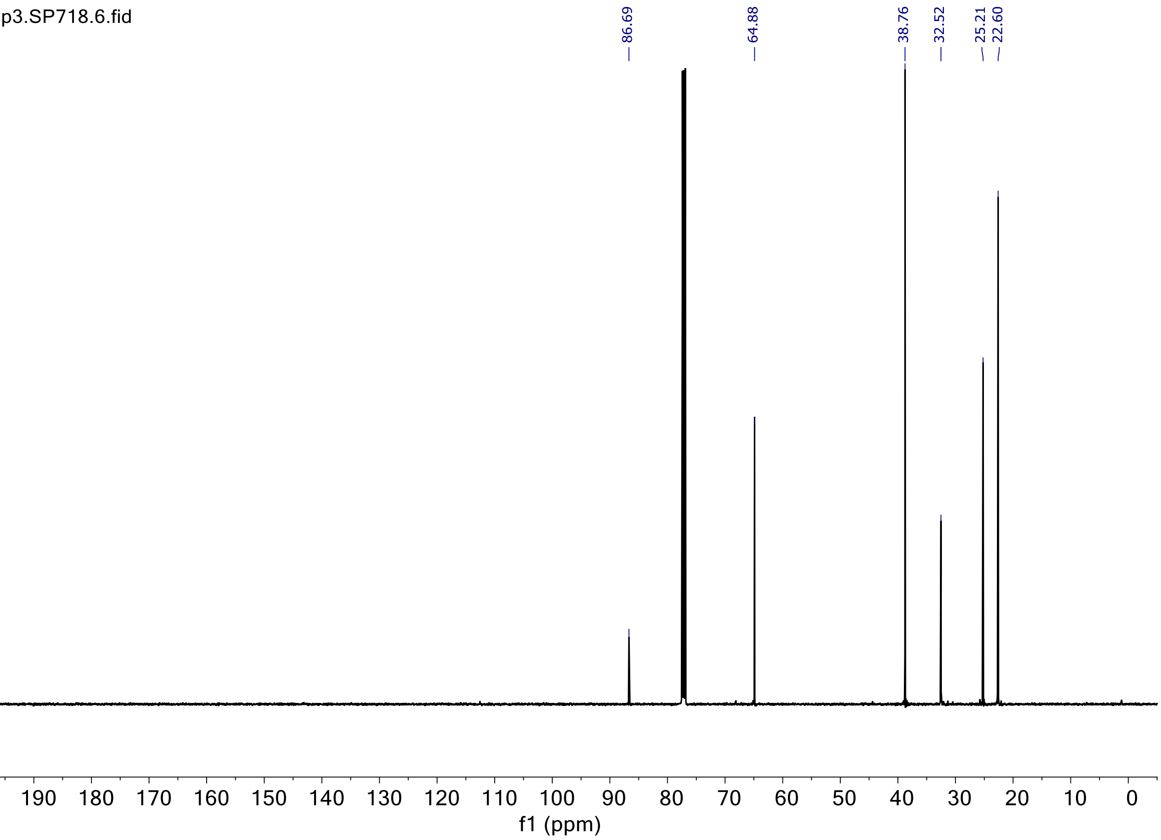

1,7-dioxaspiro[3.5]nonane (3b)

^{1}H-NMR ($CDCl_3$, 500 MHz)

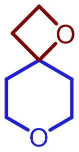

3b

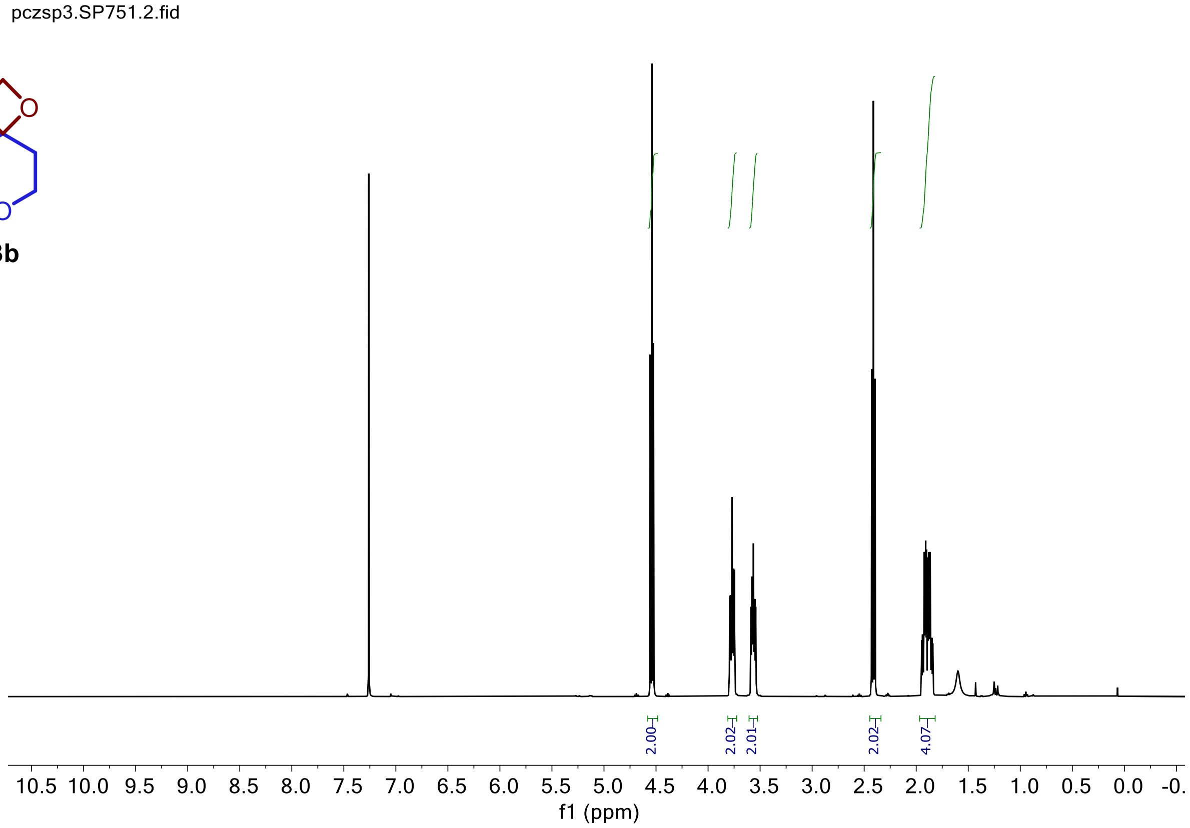

^{13}C-NMR ($CDCl_3$, 126 MHz)

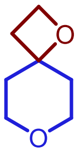

3b

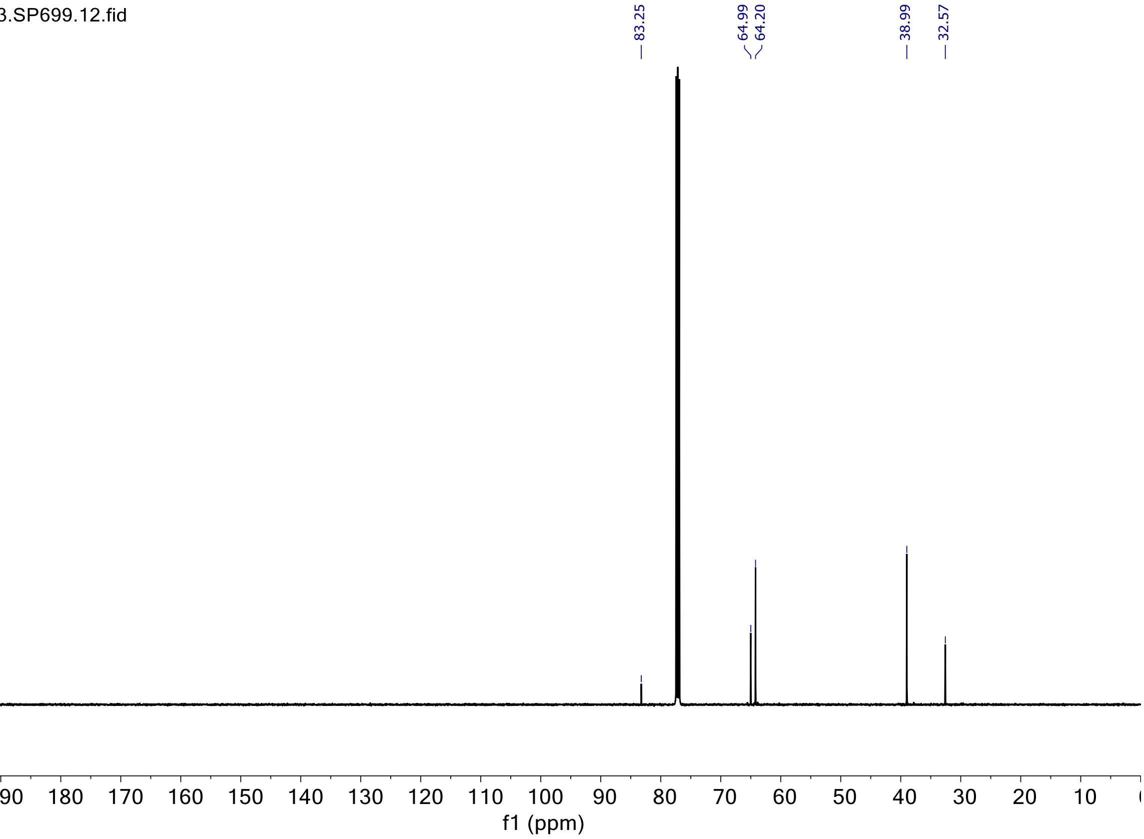

1,8,11-trioxadispiro[3.2.47.24]tridecane (3c)

^{1}H-NMR (CDCl$_3$, 500 MHz)

pcxdf1.DF1141p.1.fid

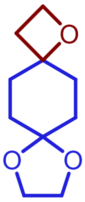

3c

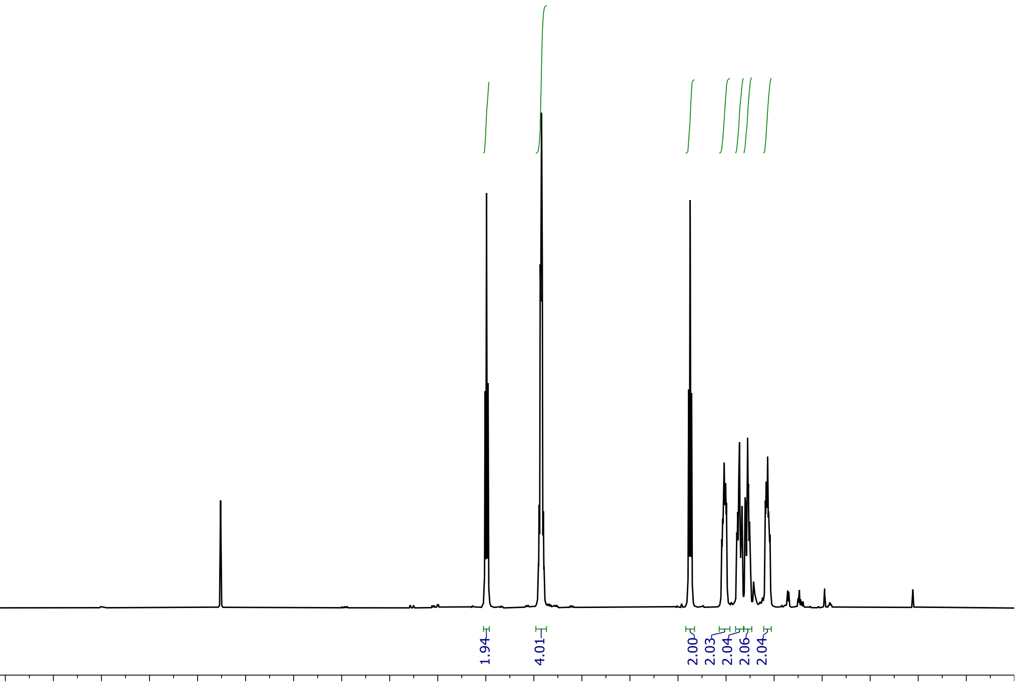

^{13}C-NMR (CDCl$_3$, 126 MHz)

pcxdf1.DF1141p.2.fid

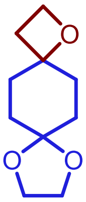

3c

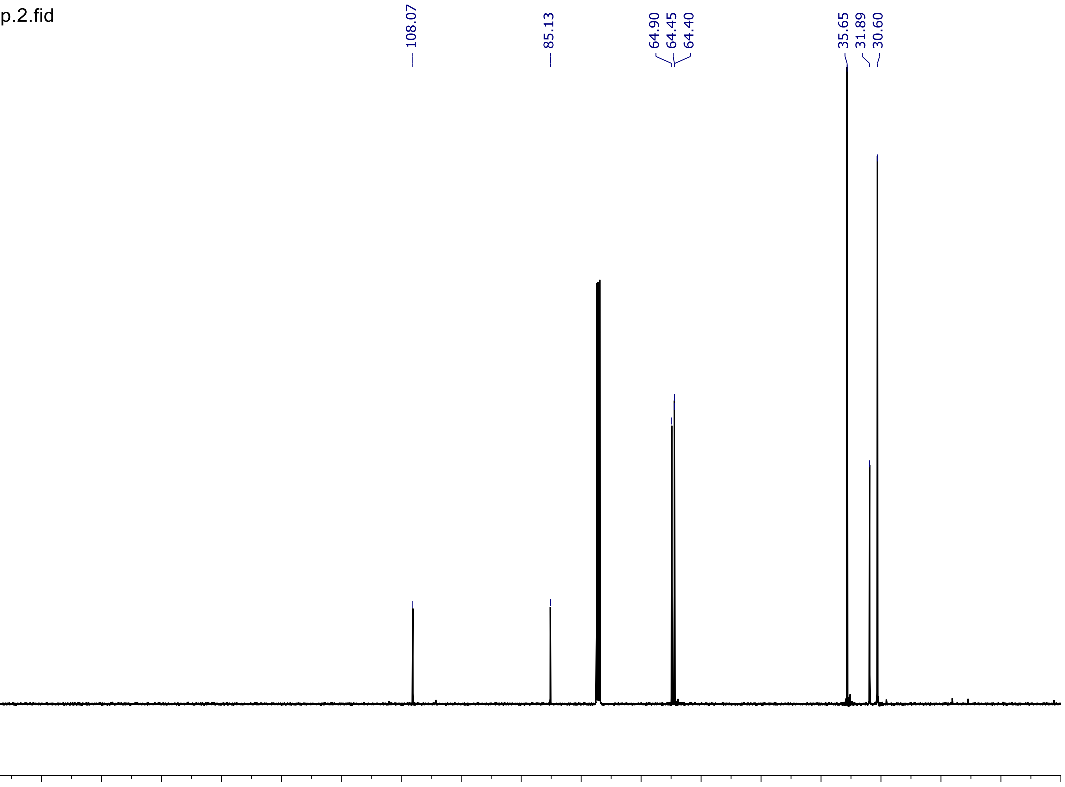

1-oxa-7-thiaspiro[3.5]nonane 7,7-dioxide (3d)

^{1}H-NMR ($CDCl_3$, 500 MHz)

pcxff1.FGF78_column.1.fid

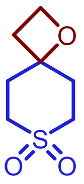

3d

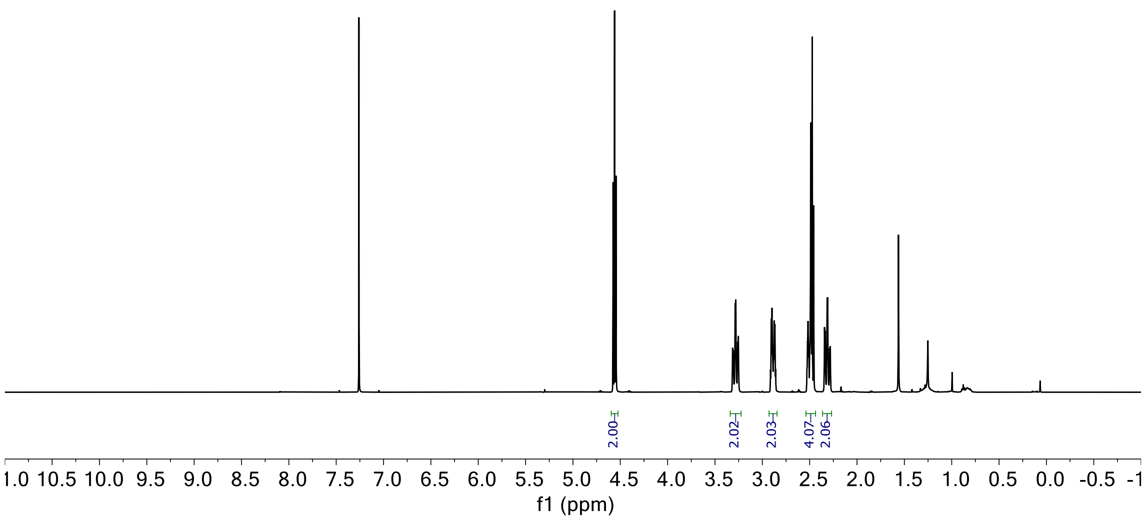

^{13}C-NMR ($CDCl_3$, 126 MHz)

pcxff1.FGF78_column.2.fid

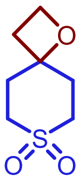

3d

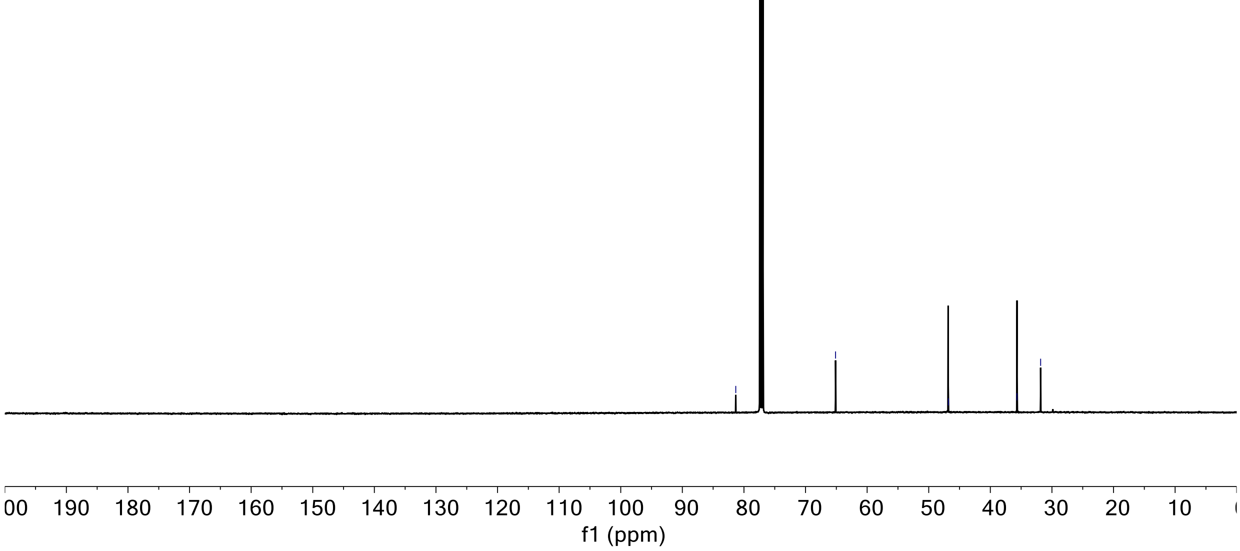

phenyl(1-oxa-7-azaspiro[3.5]nonan-7-yl)methanone (3e)

^{1}H-NMR ($CDCl_3$, 500 MHz)

pcxdf1.DF1095p_fr4860.1.fid

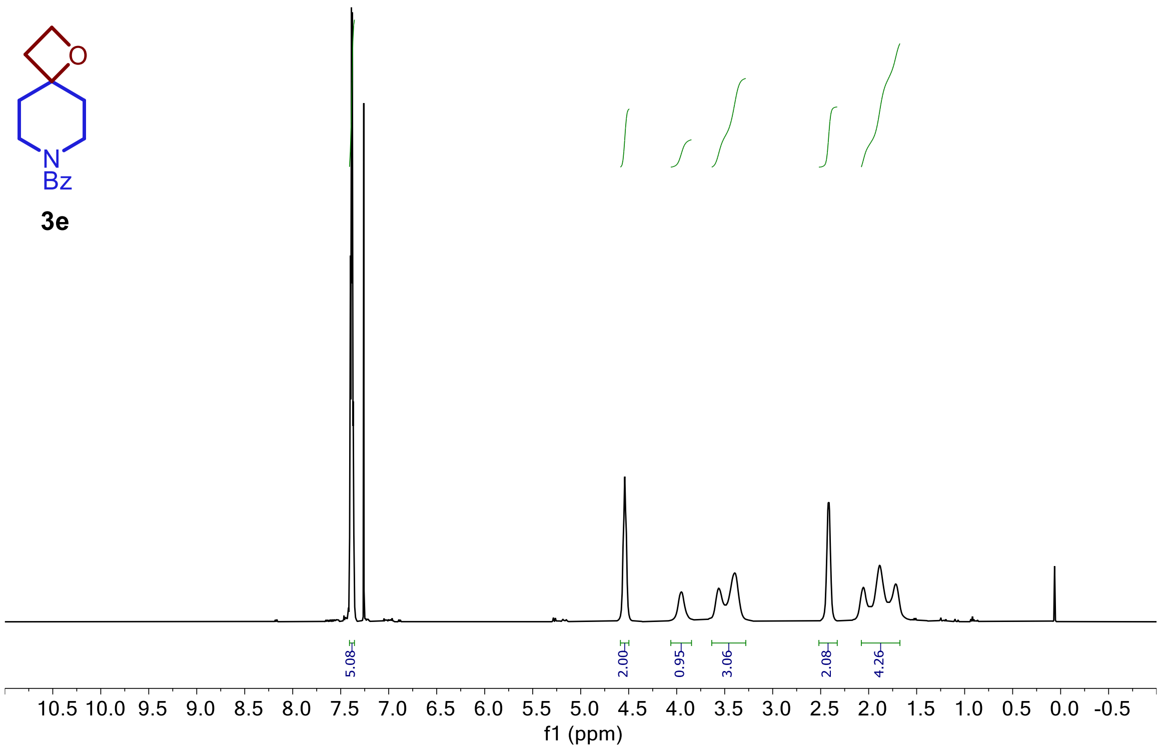

^{13}C-NMR ($CDCl_3$, 126 MHz)

pcxdf1.DF1095p_fr4860.2.fid

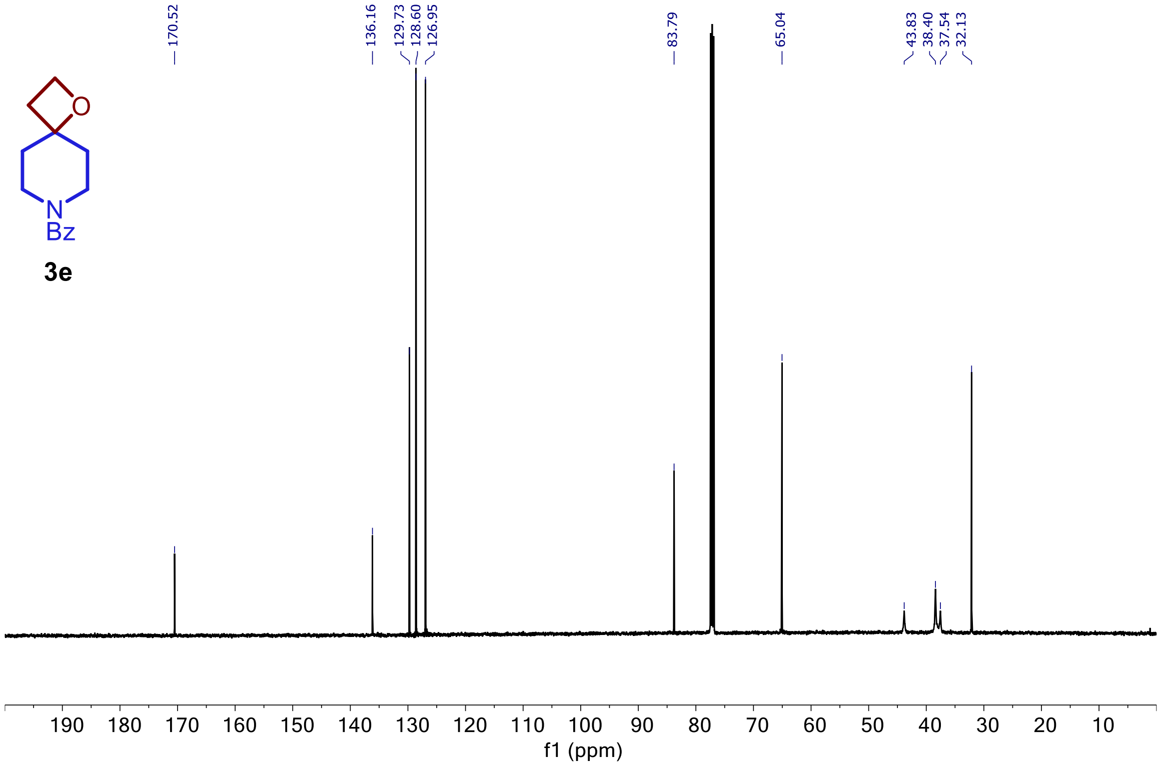

ethyl 1-oxaspiro[3.5]nonane-7-carboxylate (3f)

^{1}H-NMR ($CDCl_3$, 500 MHz)

pcxdf1.DF1113p2.1.fid

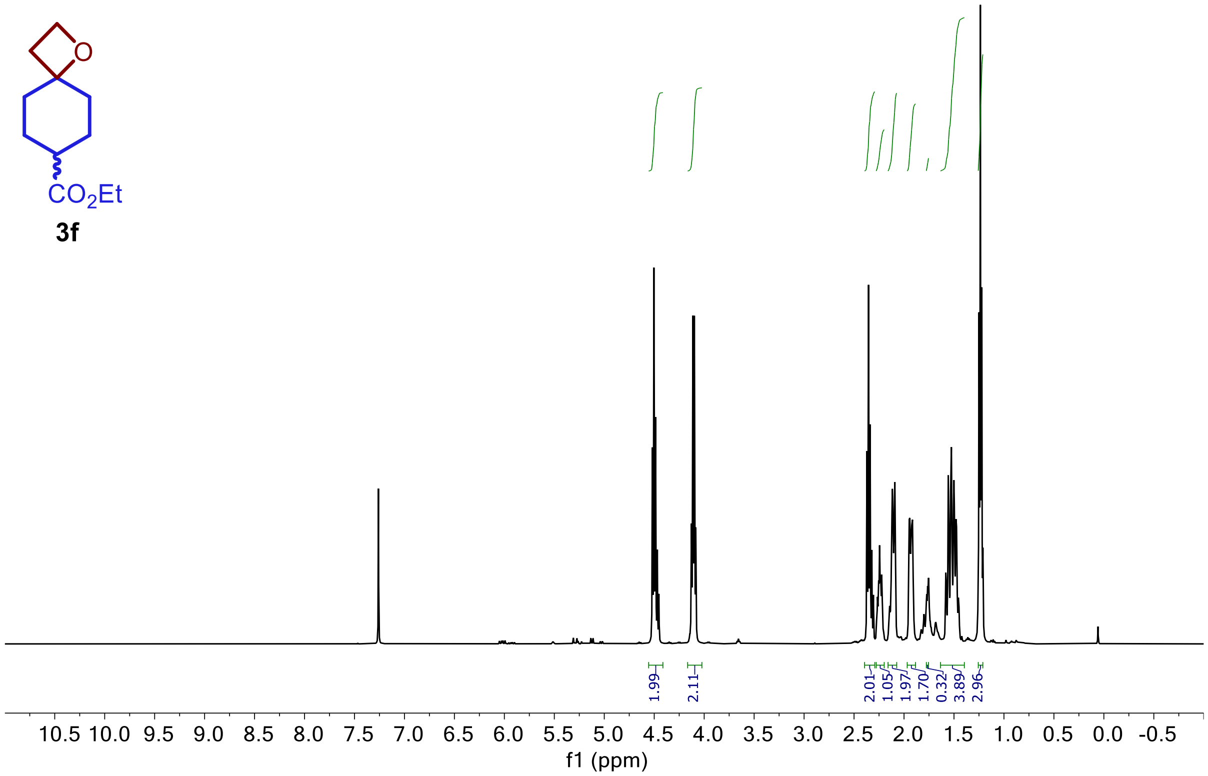

^{13}C-NMR ($CDCl_3$, 126 MHz)

pcxdf1.DF1113p.2.fid

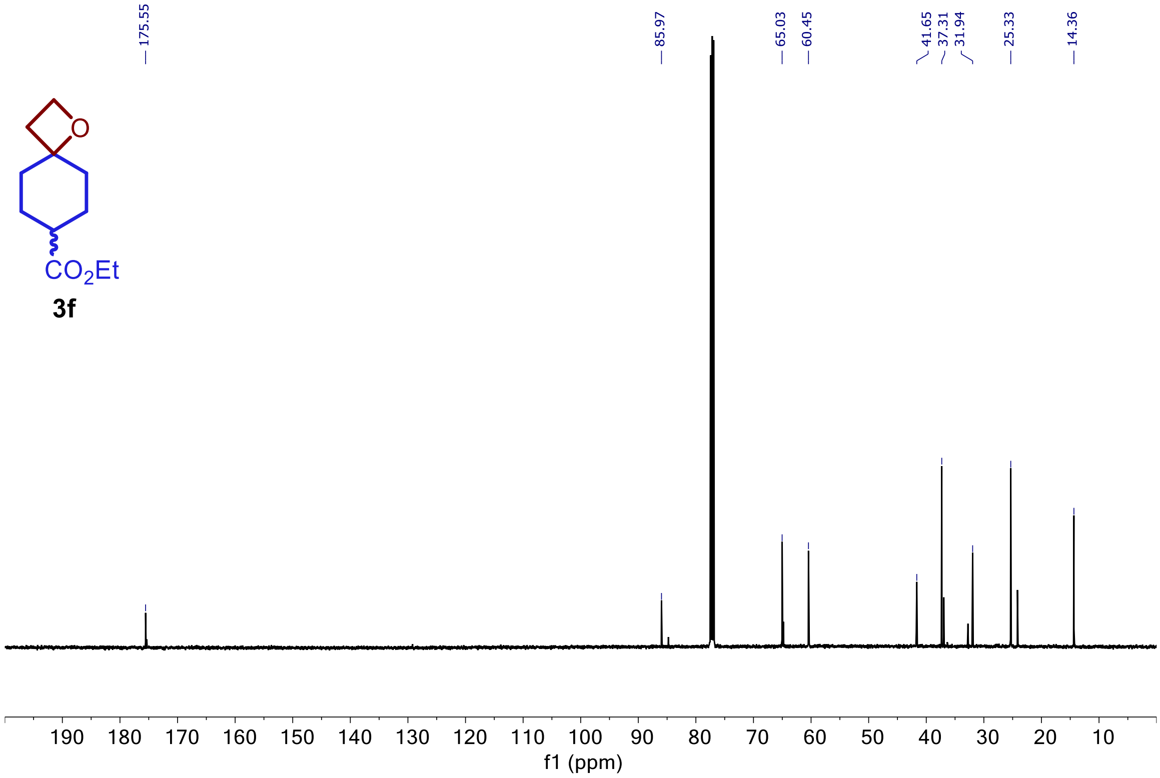

1-oxaspiro[3.11]pentadecane (3g)

^{1}H-NMR (CDCl$_3$, 400 MHz)

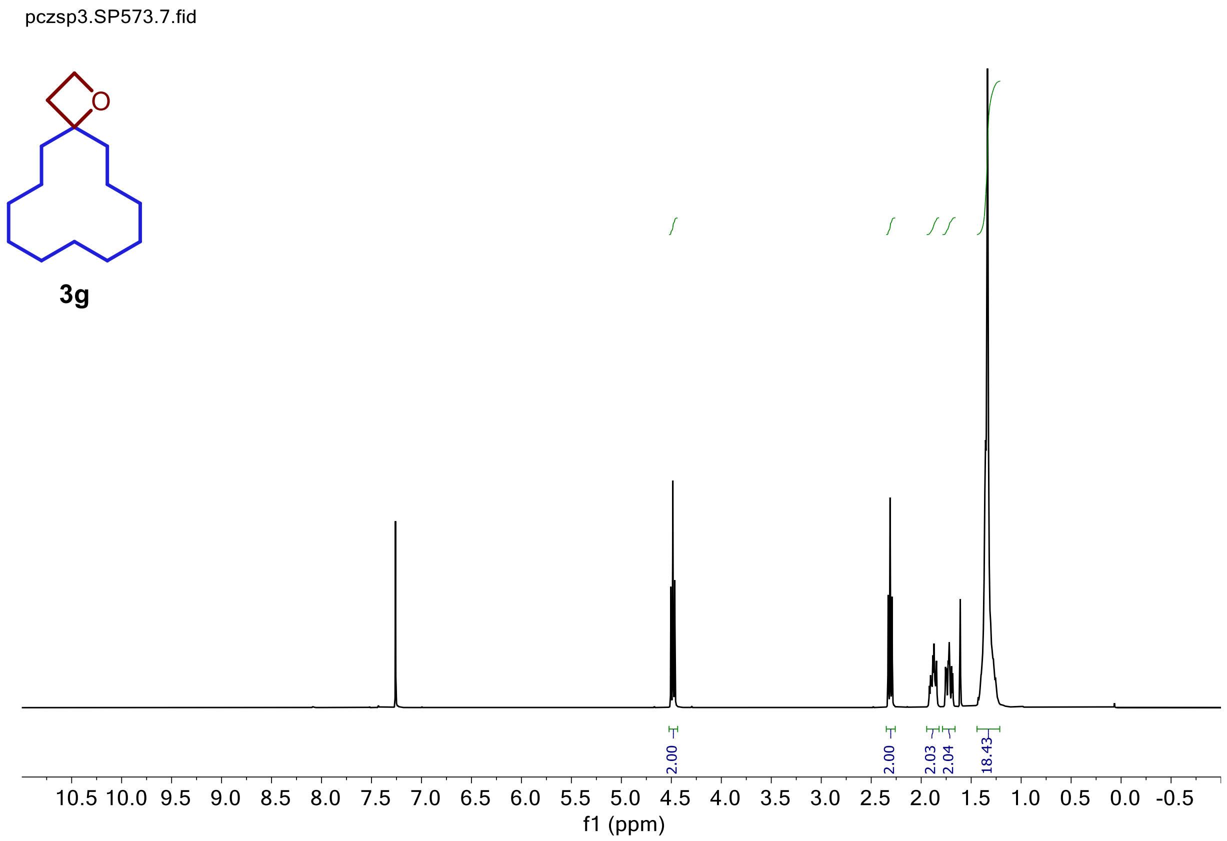

^{13}C-NMR (CDCl$_3$, 101 MHz)

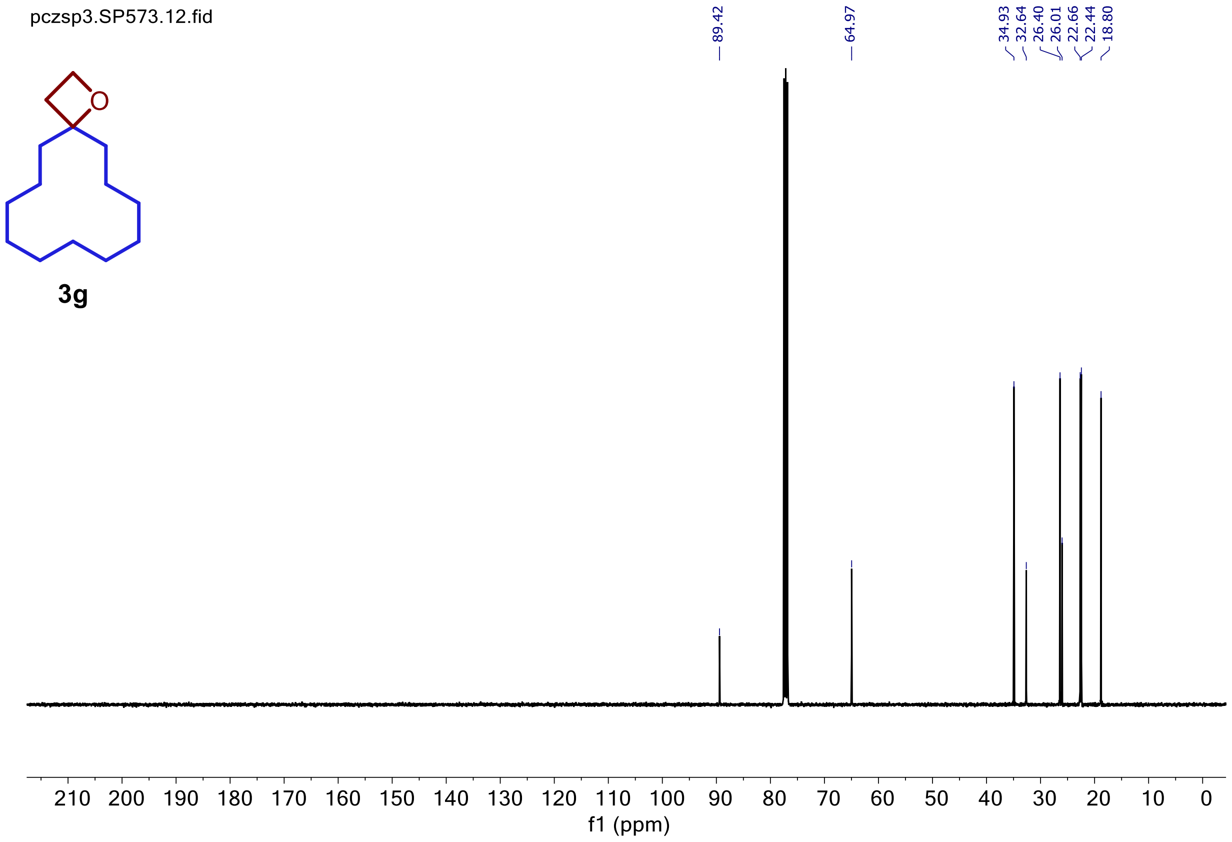

1-oxaspiro[3.3]heptane (3h)

^{1}H-NMR ($CDCl_3$, 500 MHz)

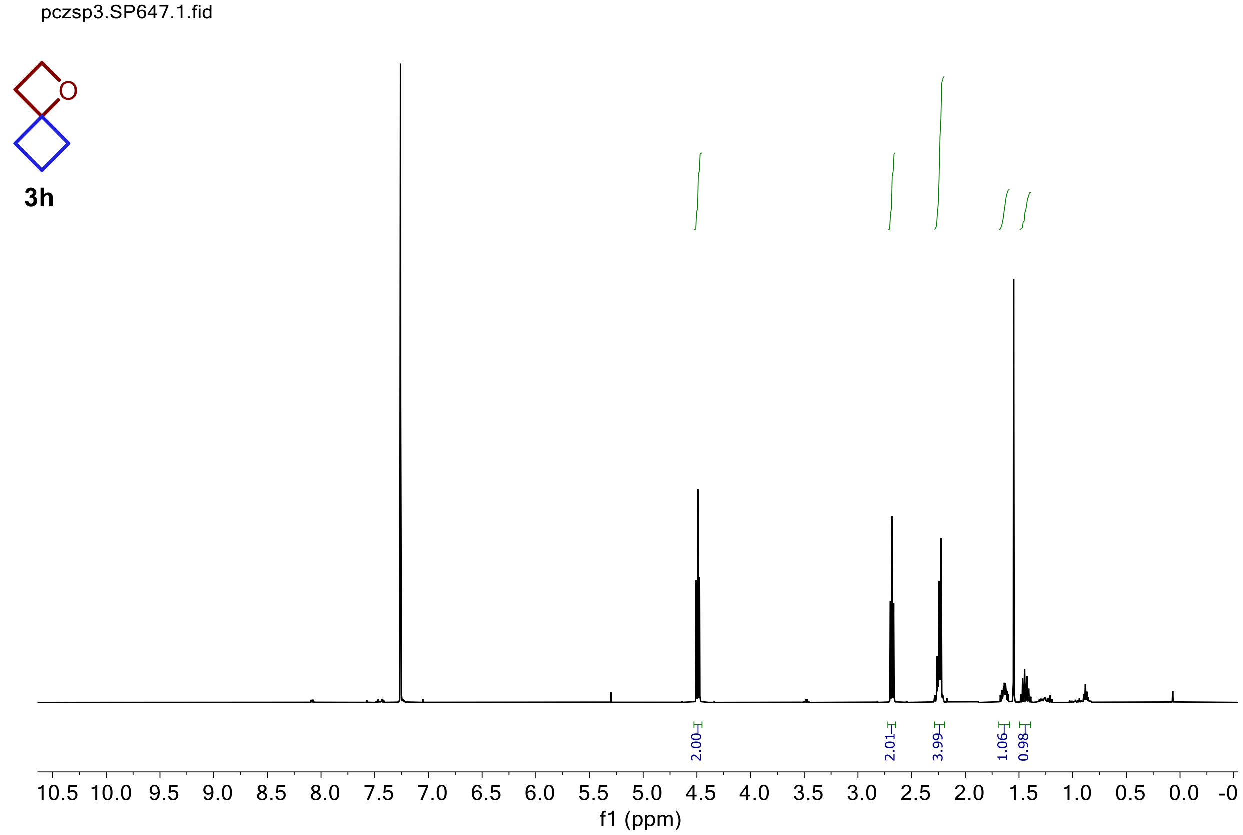

^{13}C-NMR ($CDCl_3$, 126 MHz)

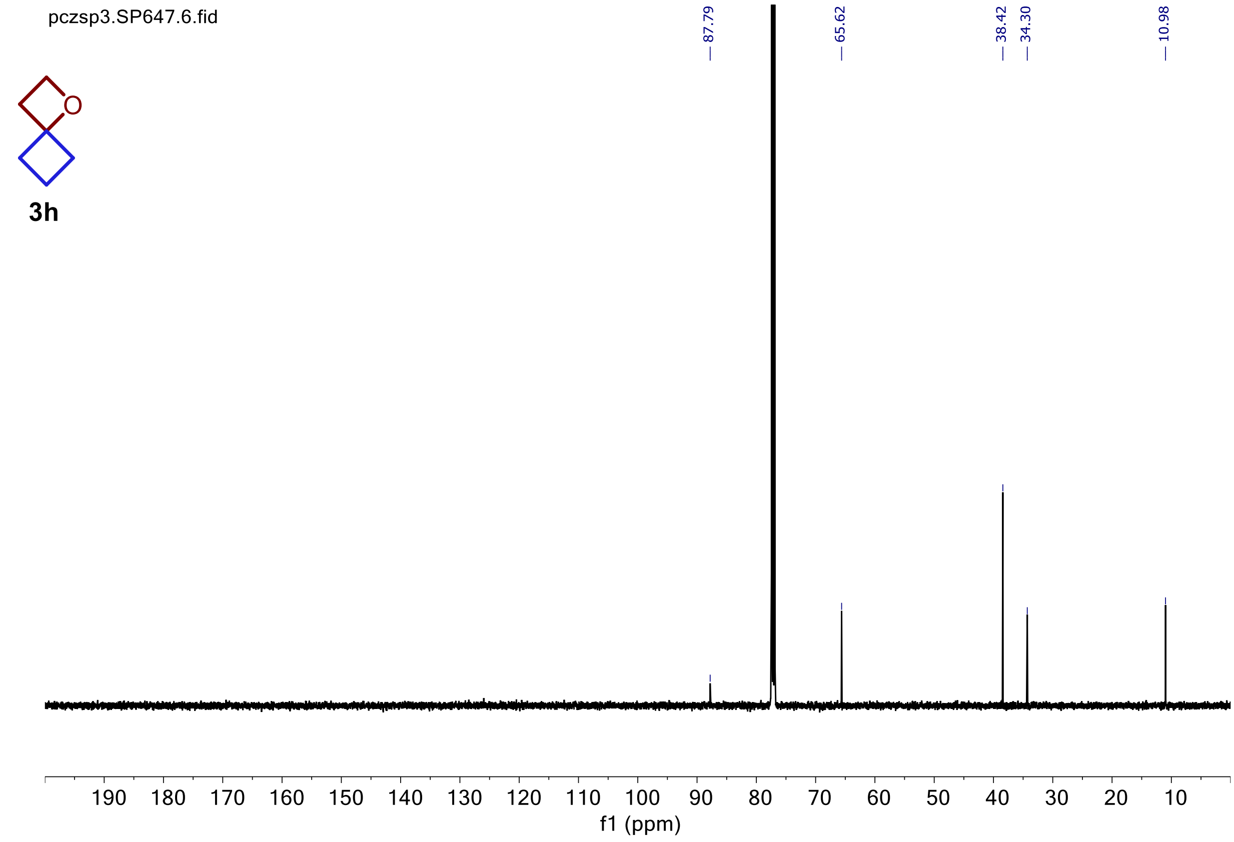

tert-butyl 1-oxa-8-azadispiro[3.1.36.14]decane-8-carboxylate (3i)

^{1}H-NMR ($CDCl_3$, 500 MHz)

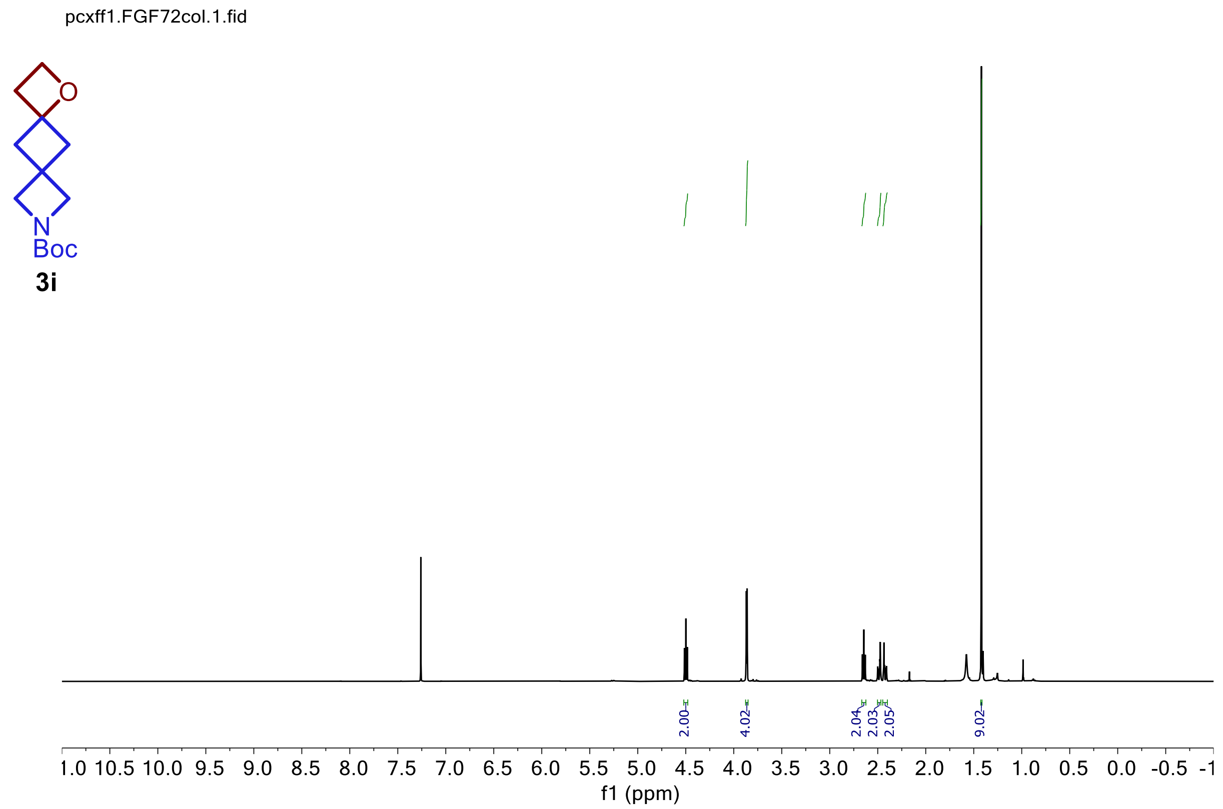

^{13}C-NMR ($CDCl_3$, 126 MHz)

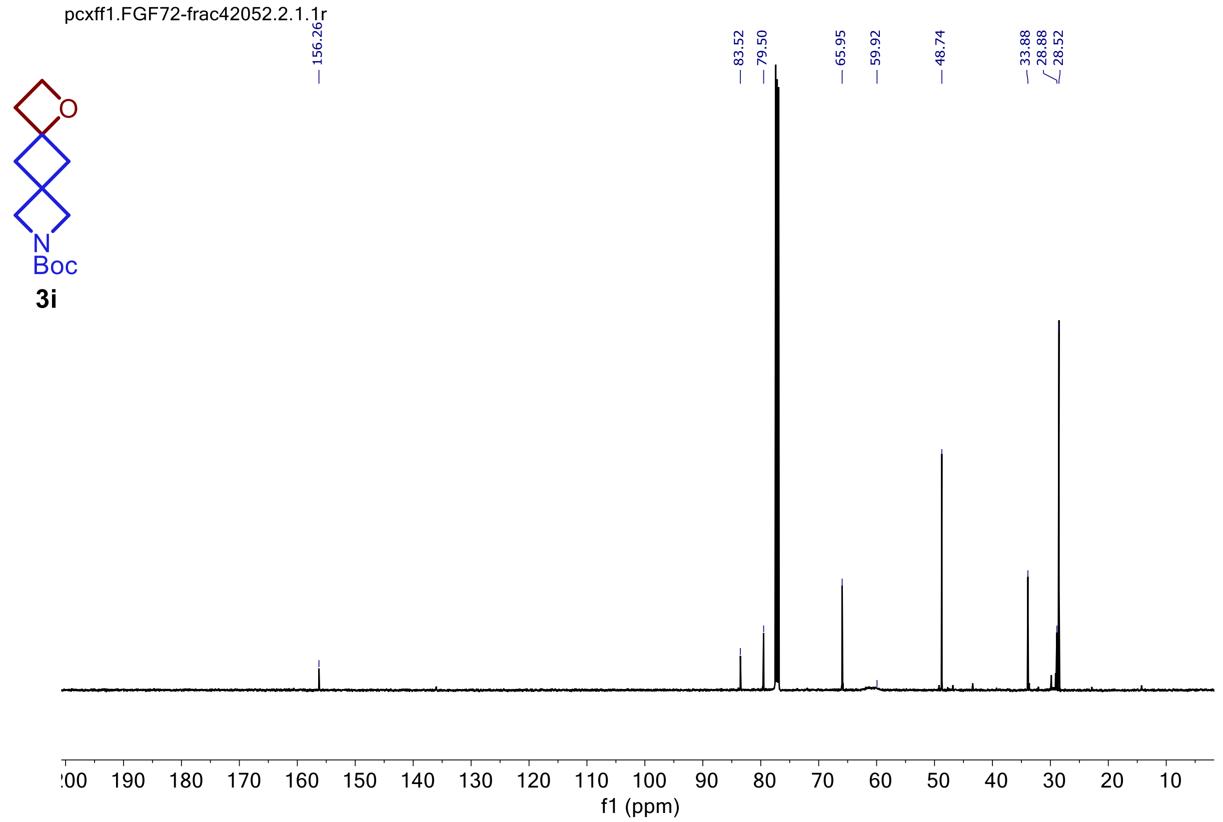

(1-oxa-8-azadispiro[3.1.36.14]decan-8-yl)(phenyl)methanone (3j)

^{1}H-NMR ($CDCl_3$, 500 MHz)

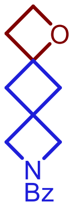

3j

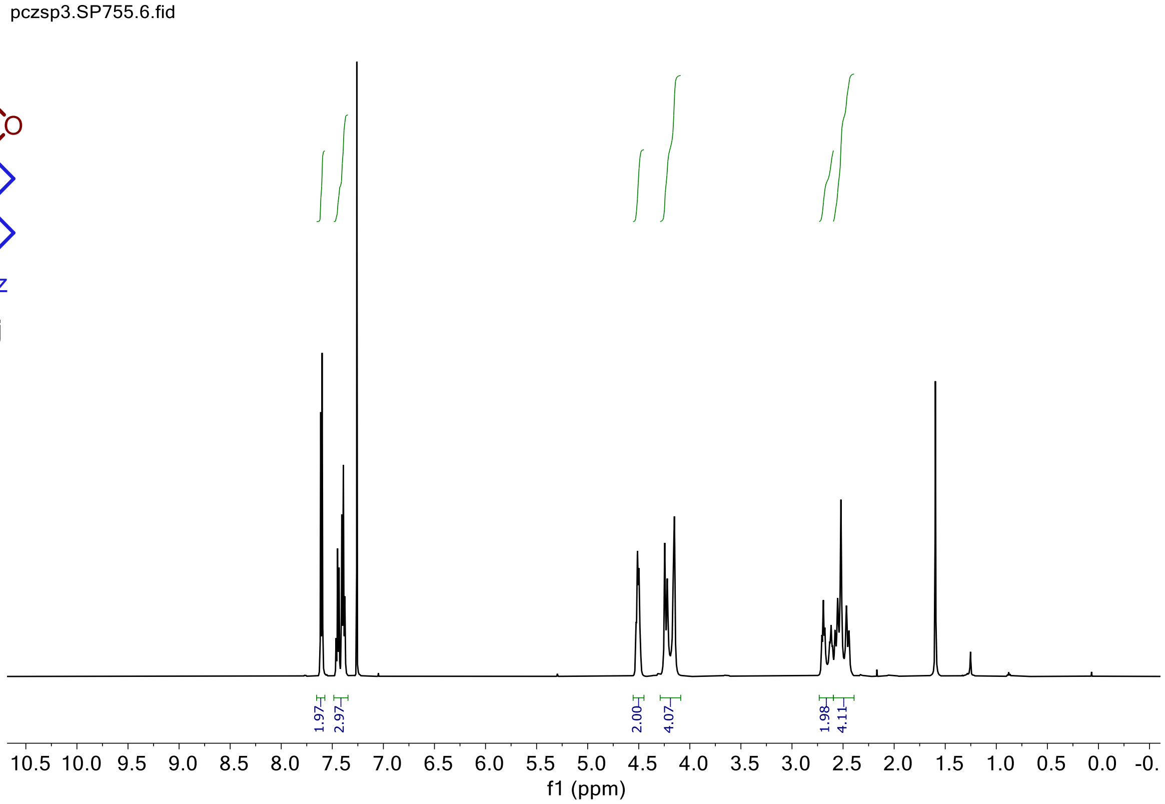

^{13}C-NMR ($CDCl_3$, 126 MHz)

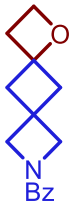

3j

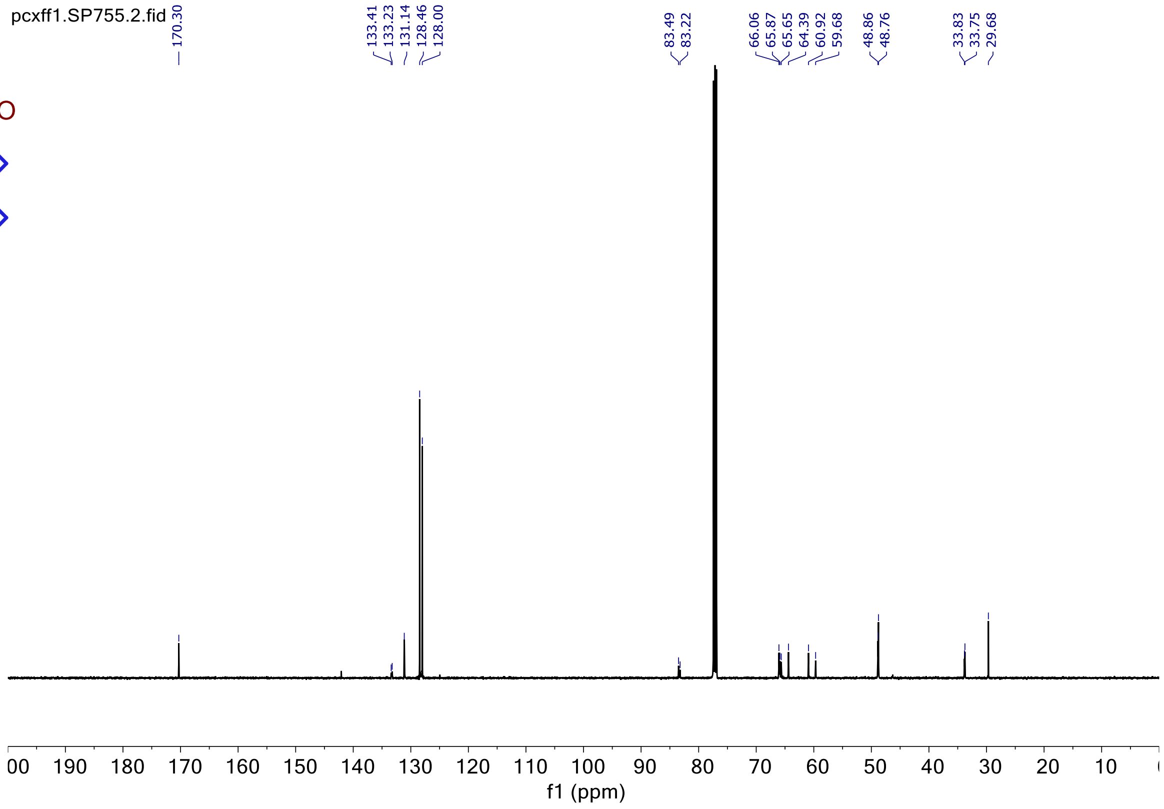

(1-oxa-9-azadispiro[3.1.5^6.1^4]dodecan-9-yl)(phenyl)methanone (3k)

^{1}H-NMR ($CDCl_3$, 400 MHz)

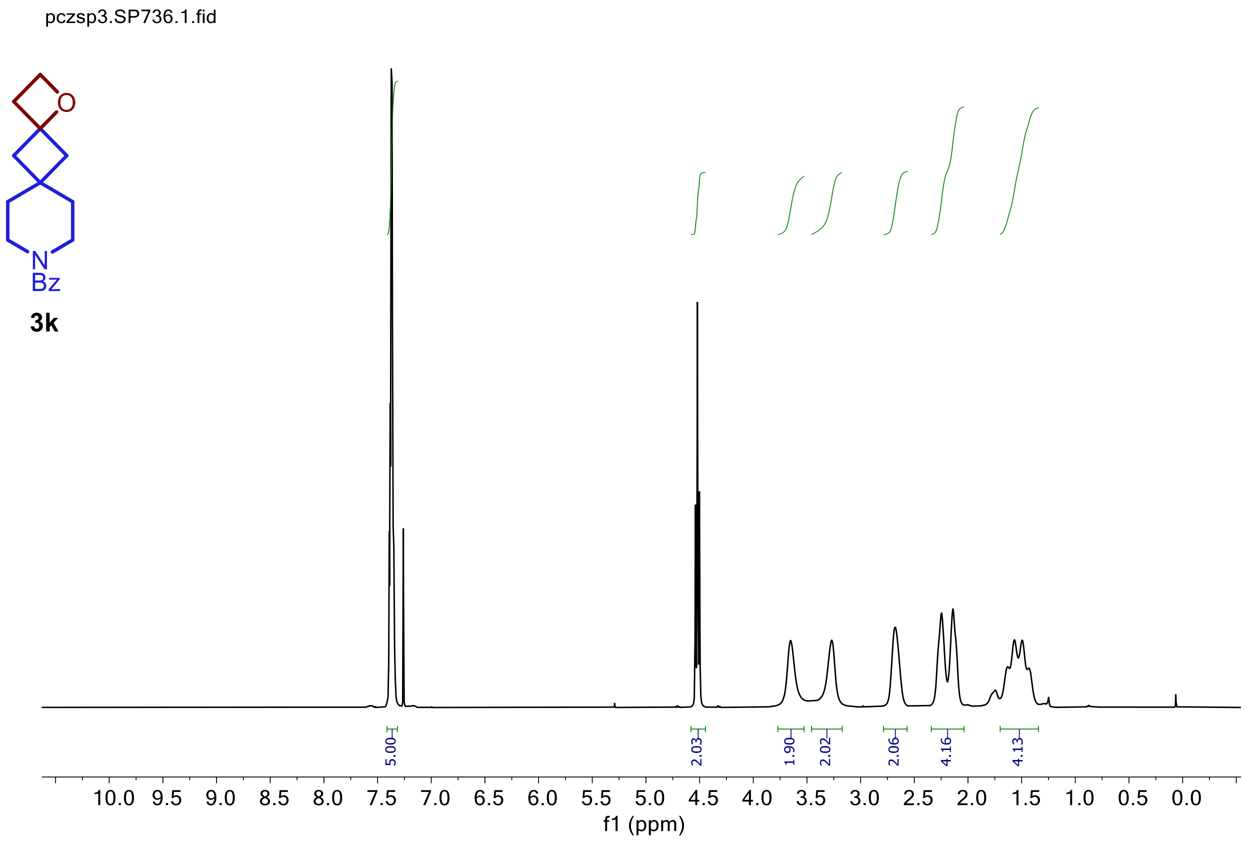

^{13}C-NMR ($CDCl_3$, 126 MHz)

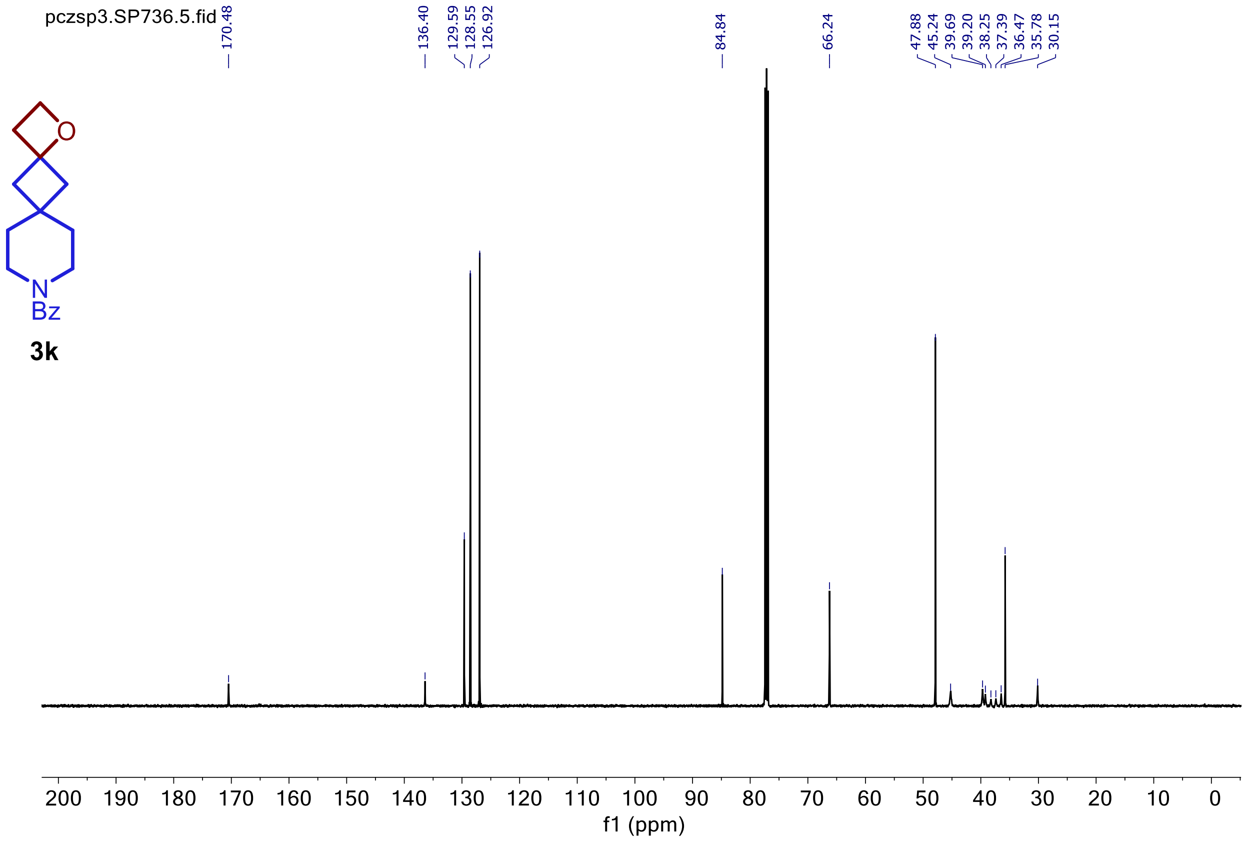

^{1}H-NMR (DMSO-d_6, 400 MHz, 80 °C)

pczsp3.SP736_VT.2.fid

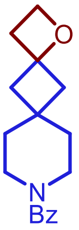

3k

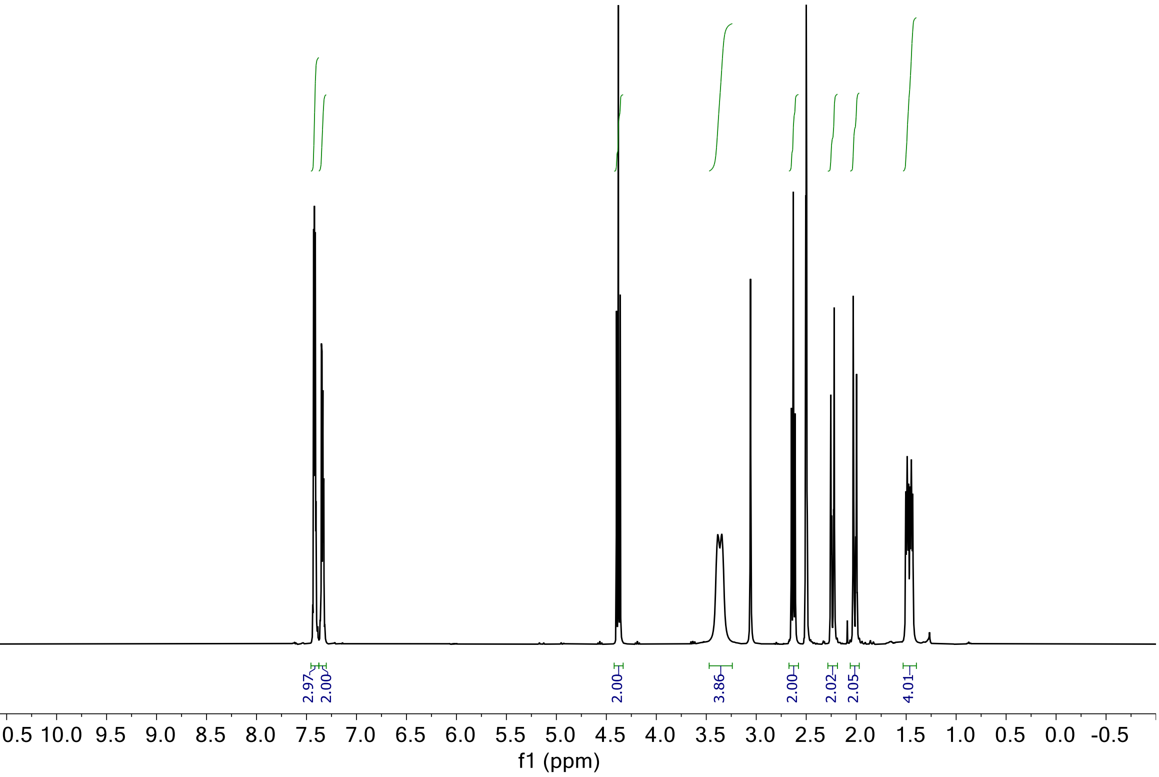

^{13}C-NMR (DMSO-d_6, 101 MHz, 80 °C)

pczsp3.SP736_VT.3.fid

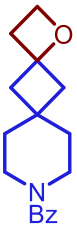

3k

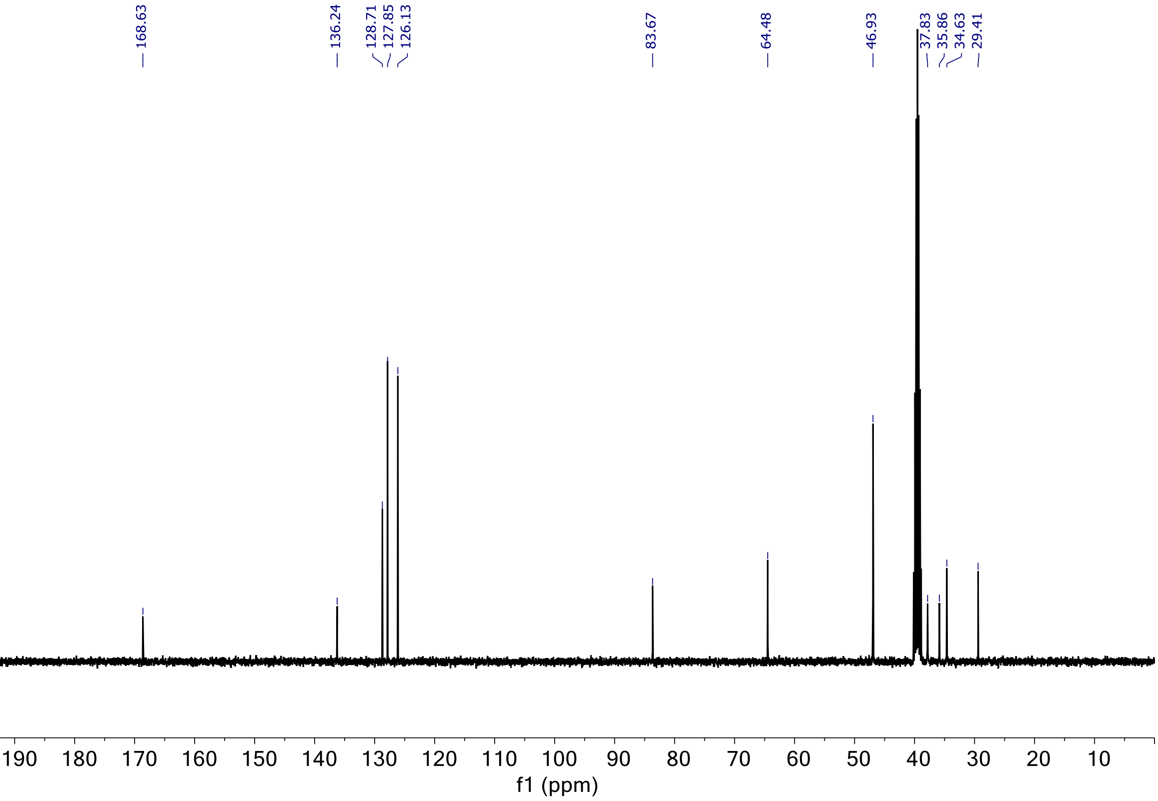

spiro[bicyclo[2.2.1]heptane-2,2'-oxetane] (3l)

^{1}H-NMR ($CDCl_3$, 500 MHz)

pcxdf1.DF1135p.1.fid

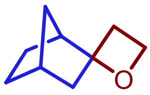

3l

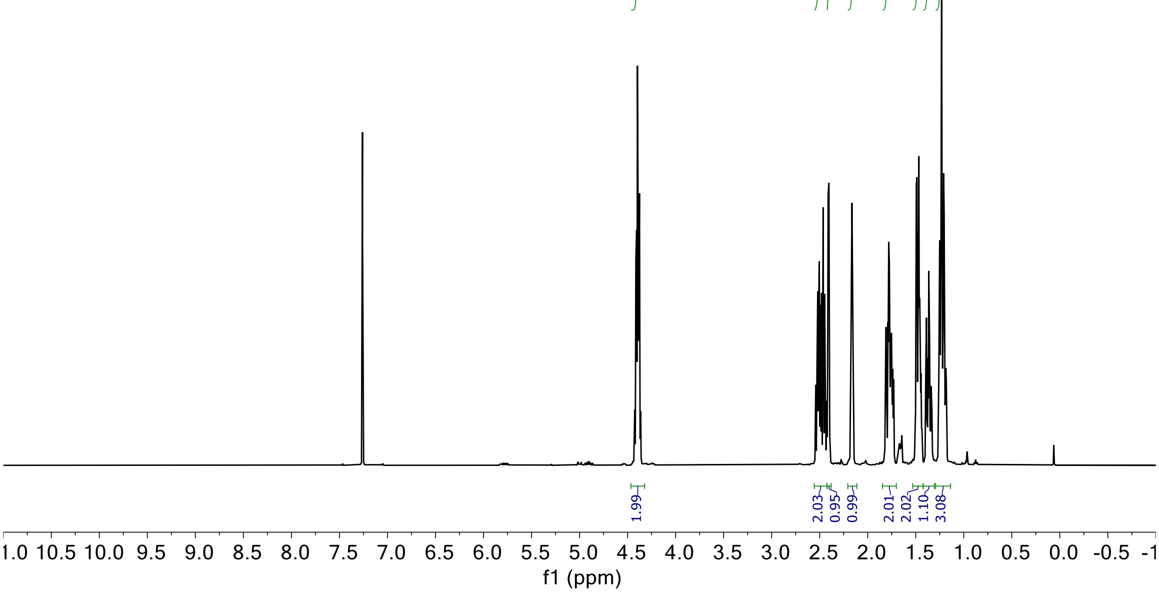

^{13}C-NMR ($CDCl_3$, 126 MHz)

pcxlc1.DF1135p.2.fid

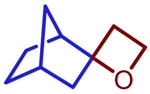

3l

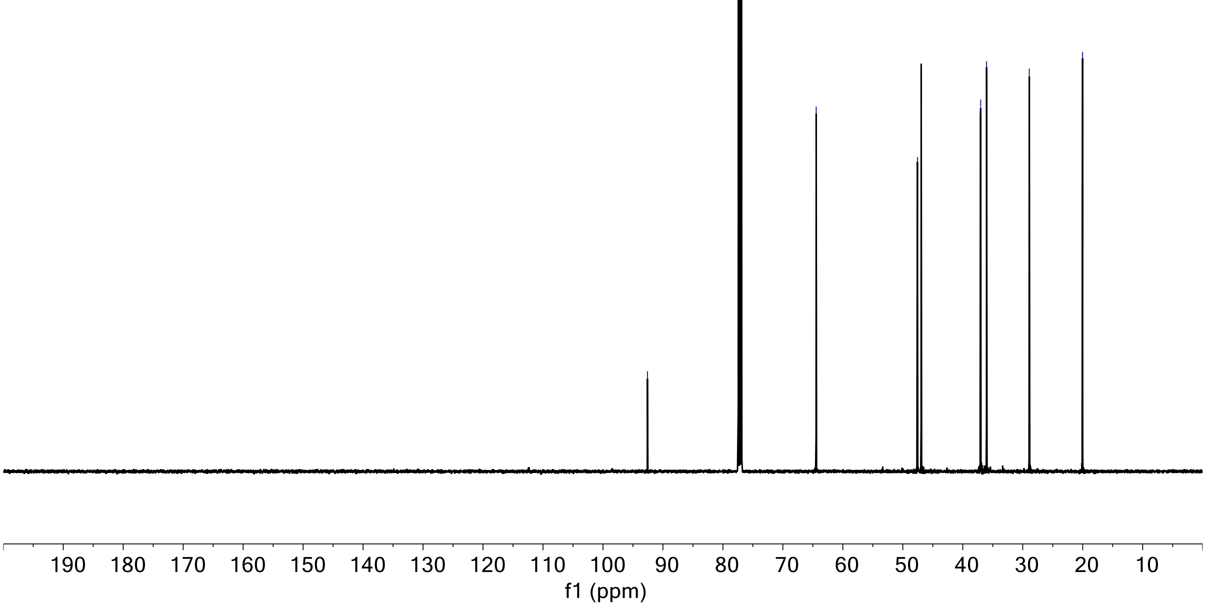

Spiro[adamantane-2,2'-oxetane] (3m)

^{1}H-NMR ($CDCl_3$, 500 MHz)

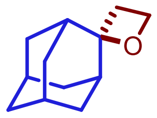

3m

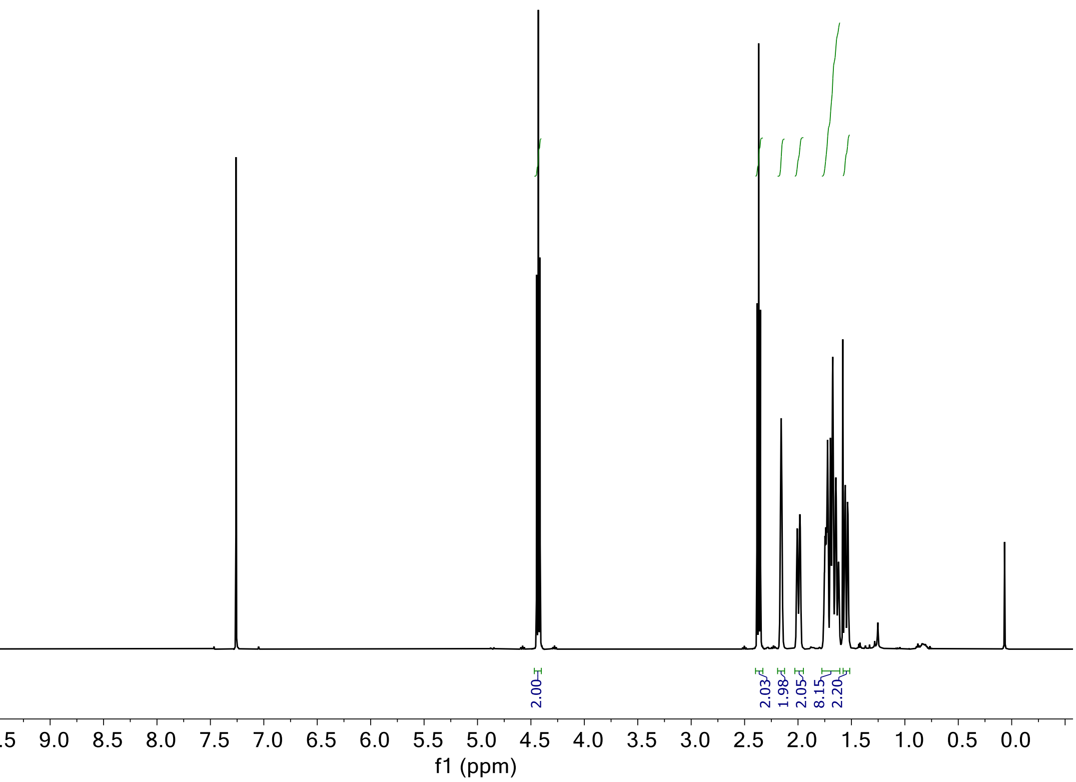

^{13}C-NMR ($CDCl_3$, 101 MHz)

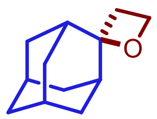

3m

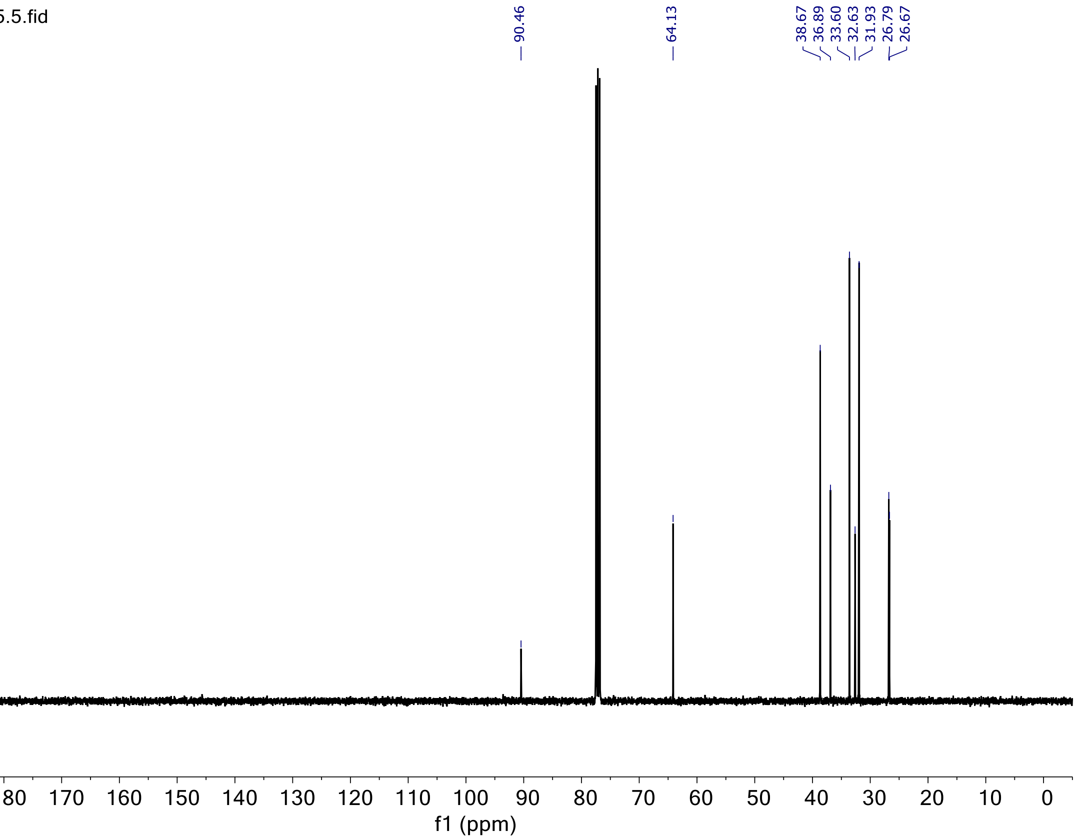

tert-butyl (1*R*,3s,5*S*)-8-azaspiro[bicyclo[3.2.1]octane-3,2'-oxetane]-8-carboxylate (3n)

^{1}H-NMR (CDCl3, 500 MHz)

pczsp3.DF1189A.1.fid

3n

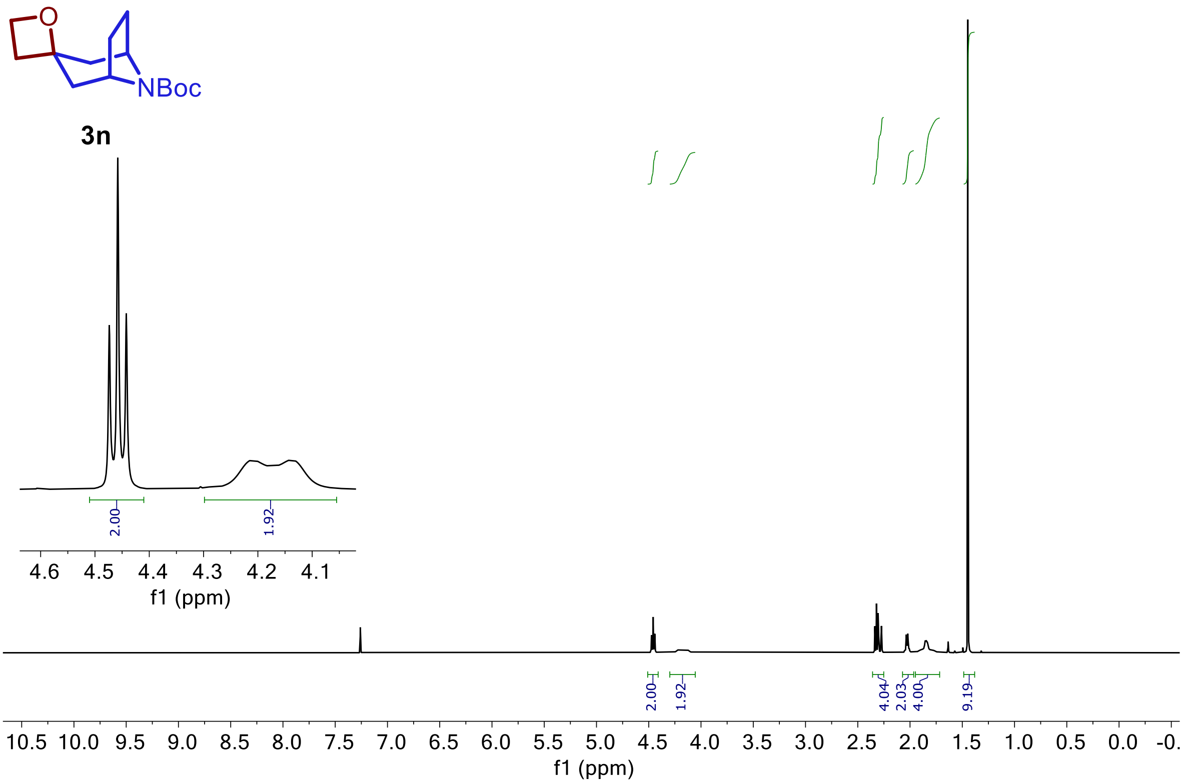

^{13}C-NMR (CDCl$_3$, 126 MHz)

pcxdf1.DF1189p_fr1529.2.fid

3n

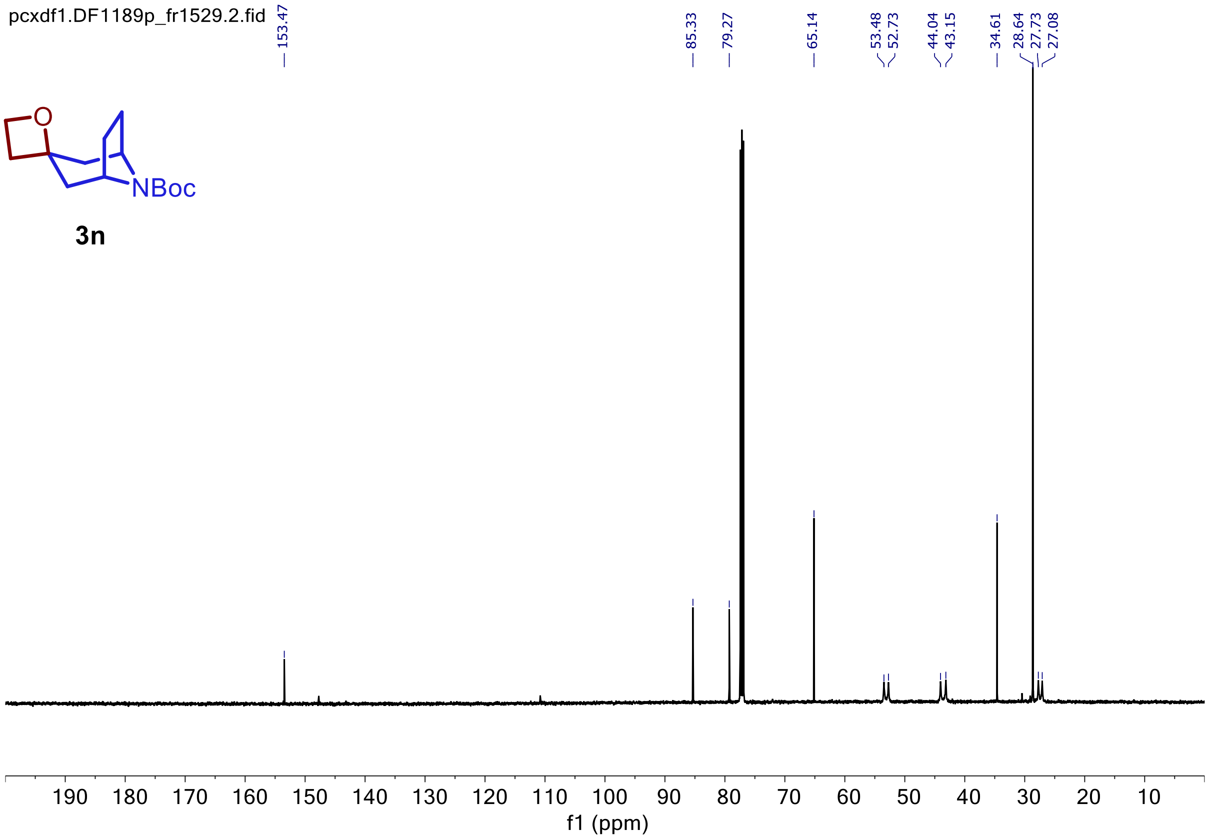

^{1}H-NMR (DMSO-d_6, 500 MHz, 80°C)

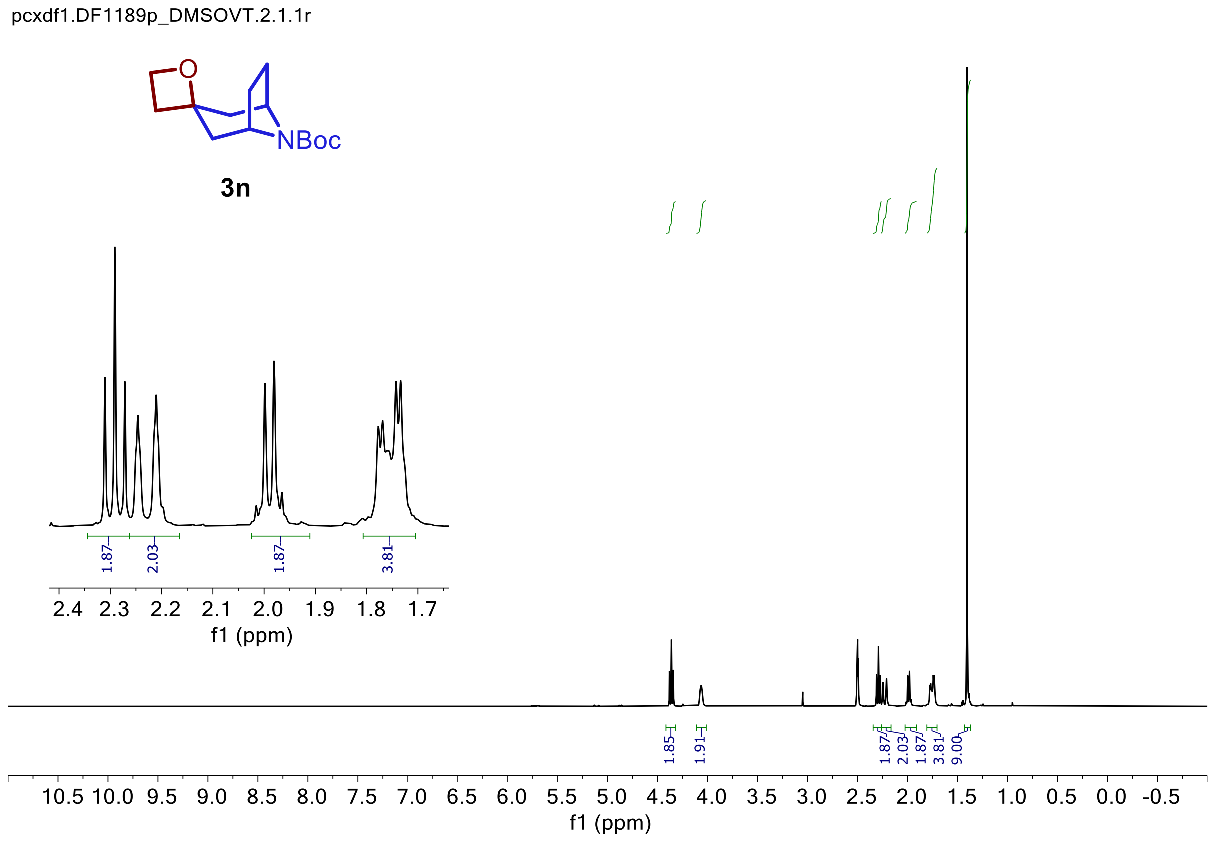

^{13}C-NMR (DMSO-d_6, 126 MHz, 80°C)

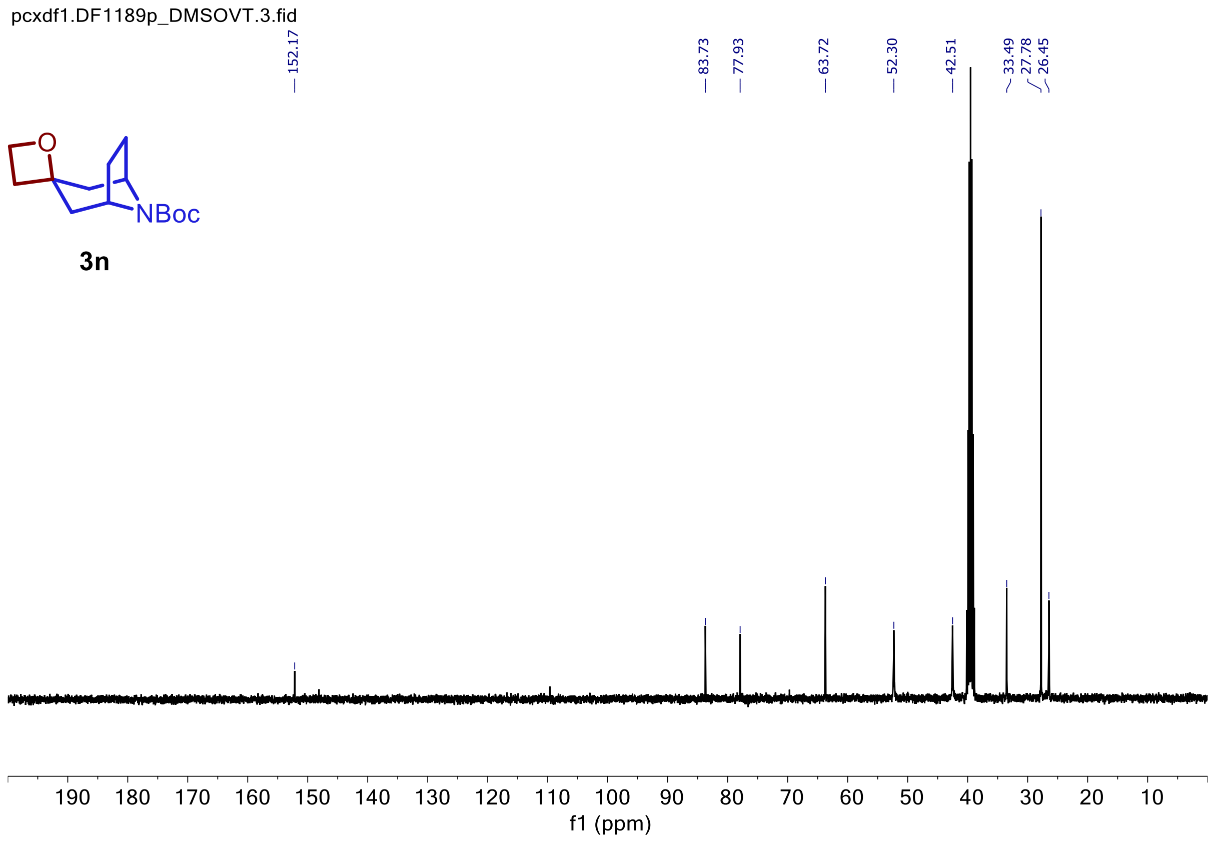

7-tetradecanol (3o)

^{1}H-NMR ($CDCl_3$, 500 MHz)

pczsp3.SP593.7.fid

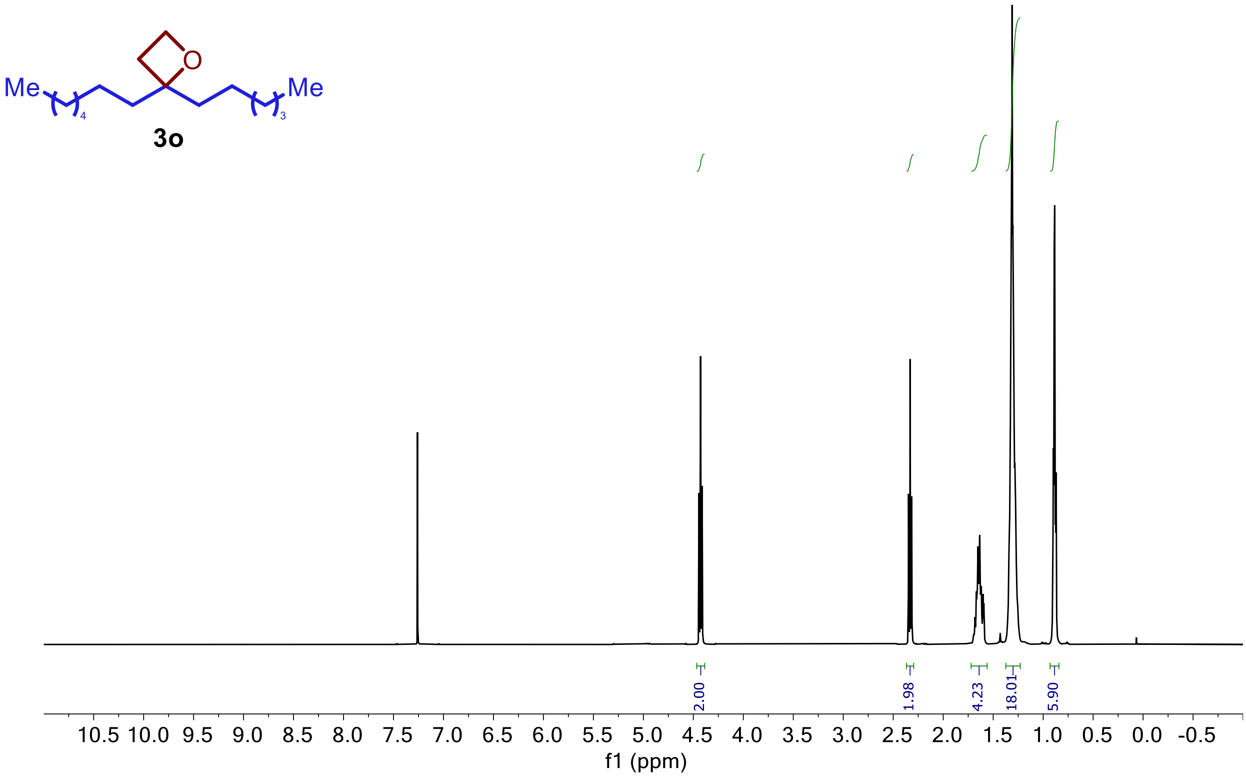

^{13}C-NMR ($CDCl_3$, 126 MHz)

pczsp3.SP593.8.fid

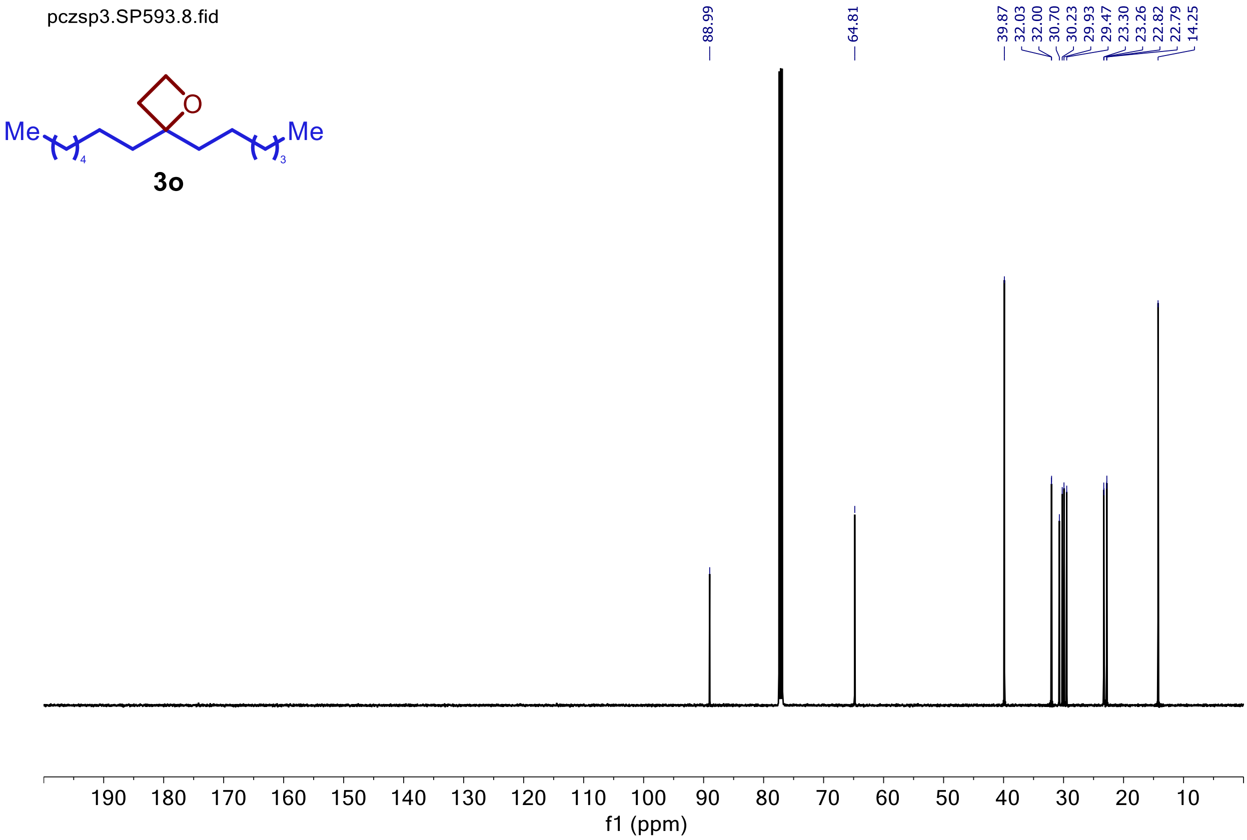

2-methyl-2-phenethyloxetane (3p)

^{1}H-NMR ($CDCl_3$, 500 MHz)

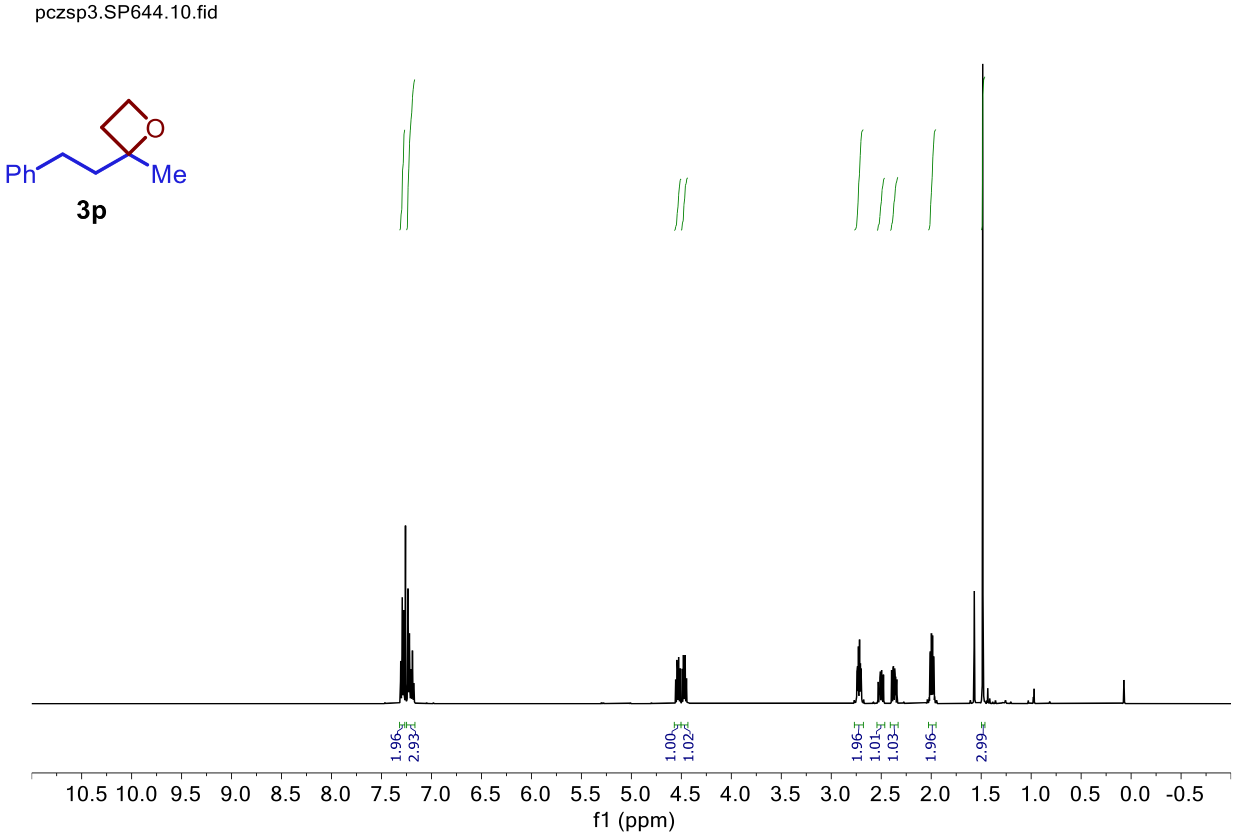

^{13}C-NMR ($CDCl_3$, 126 MHz)

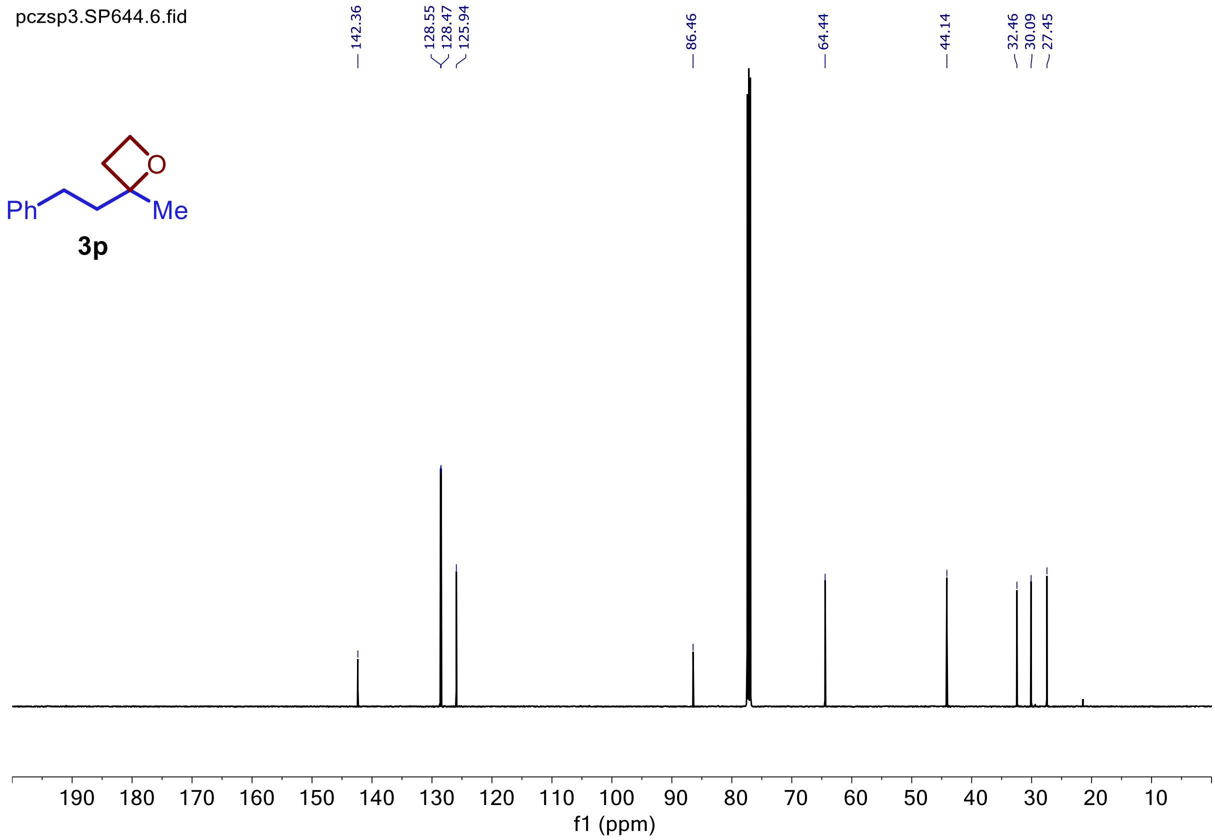

tert-butyl(4-(2-methyloxetan-2-yl)butoxy)diphenylsilane (3q)

^{1}H-NMR ($CDCl_3$, 500 MHz)

pczsp3.SP712.1.fid

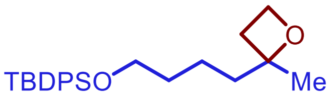

3q

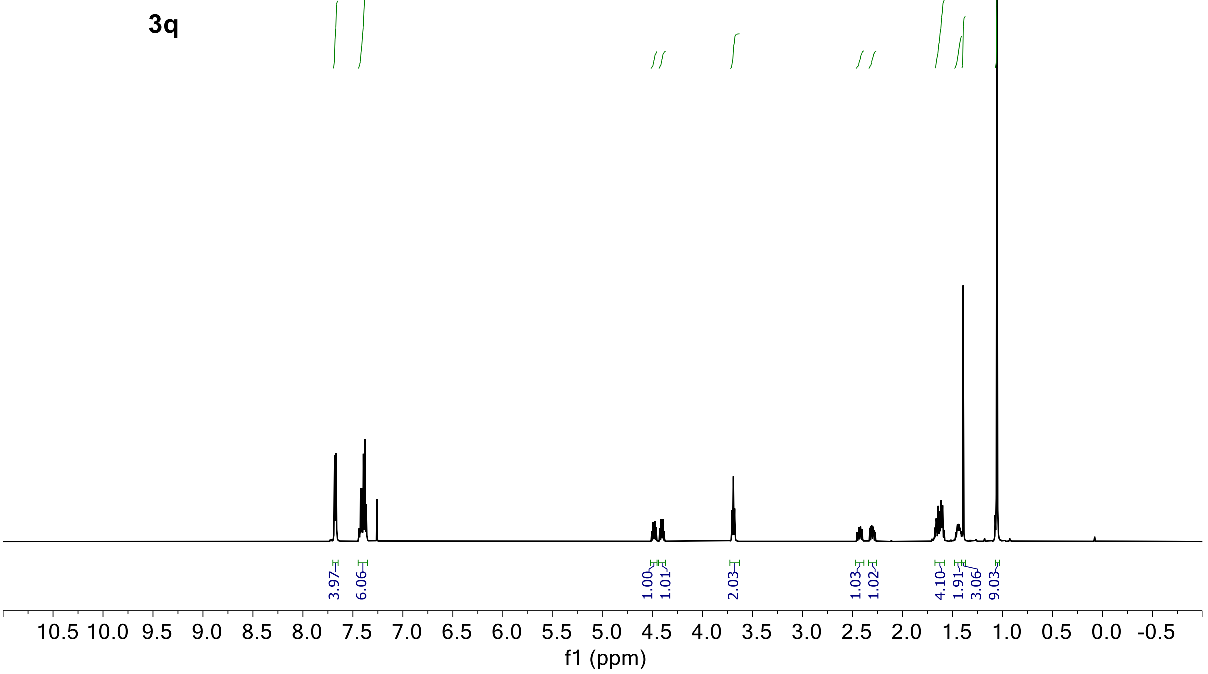

^{13}C-NMR ($CDCl_3$, 126 MHz)

pczsp3.SP712.6.fid

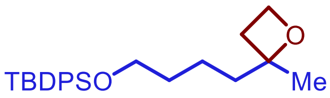

3q

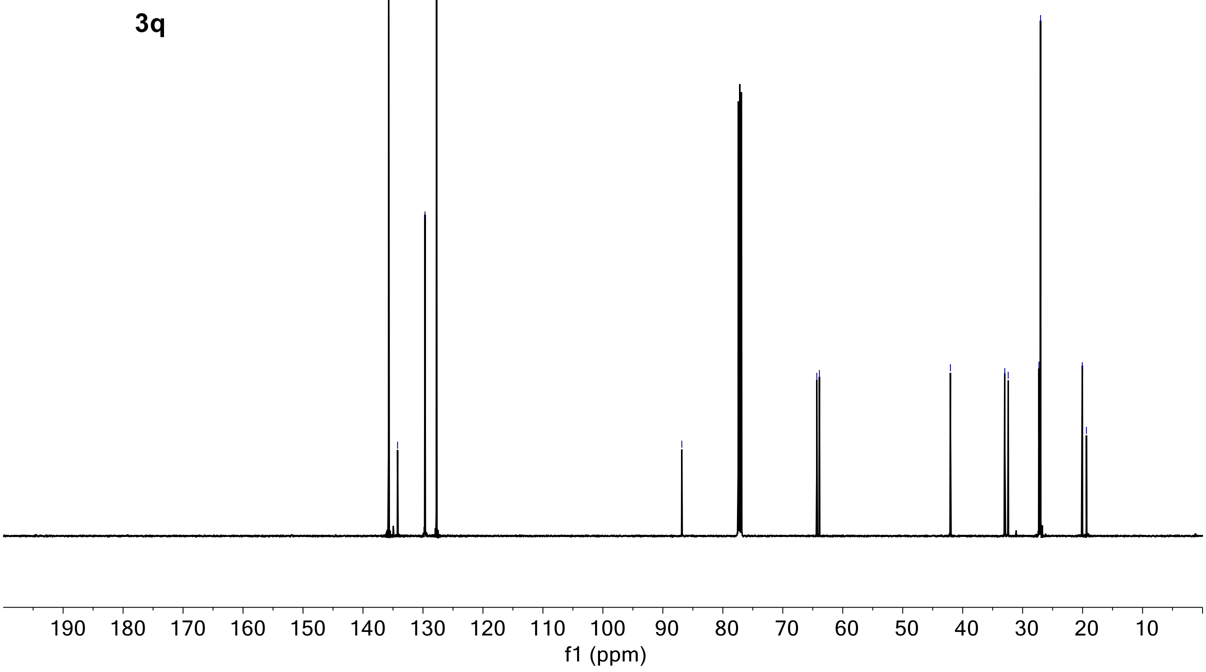

11-(2-hexyloxetan-2-yl)undecanenitrile (3r)

^{1}H-NMR (CDCl$_3$, 500 MHz)

pcxff1.FGf104frac36-52.1.fid

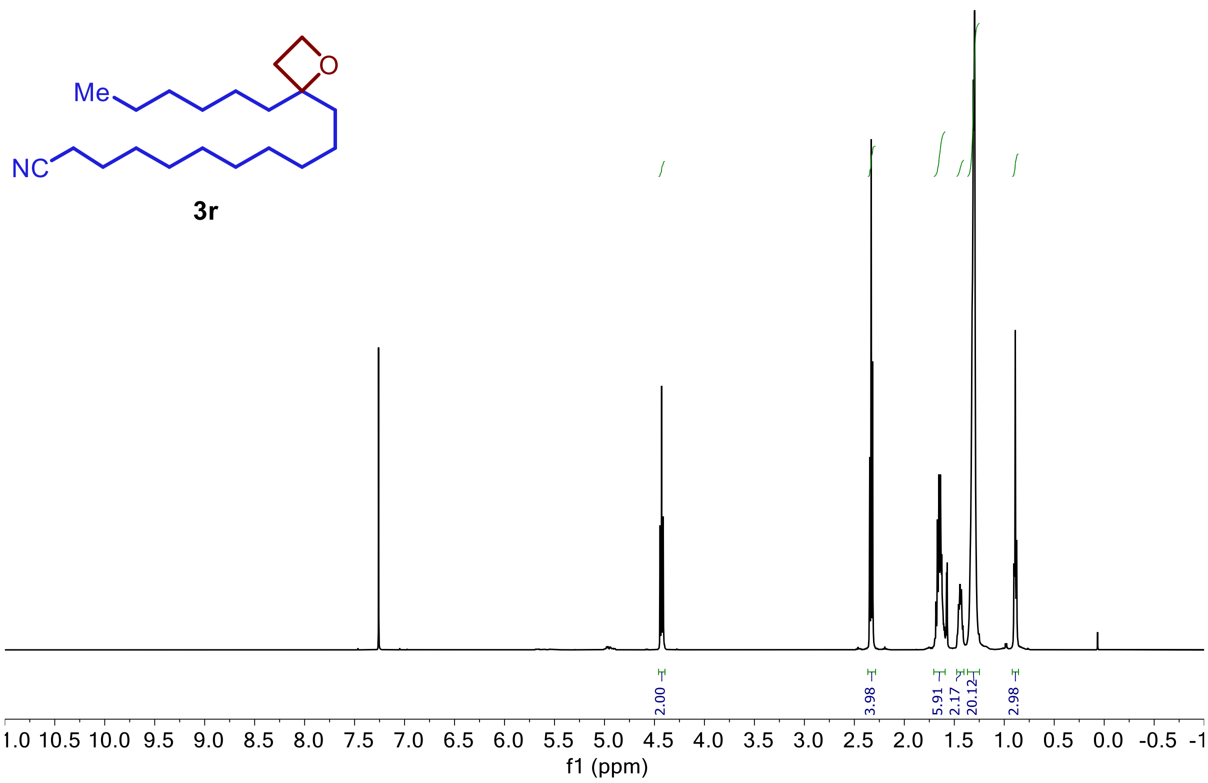

^{13}C-NMR (CDCl$_3$, 126 MHz)

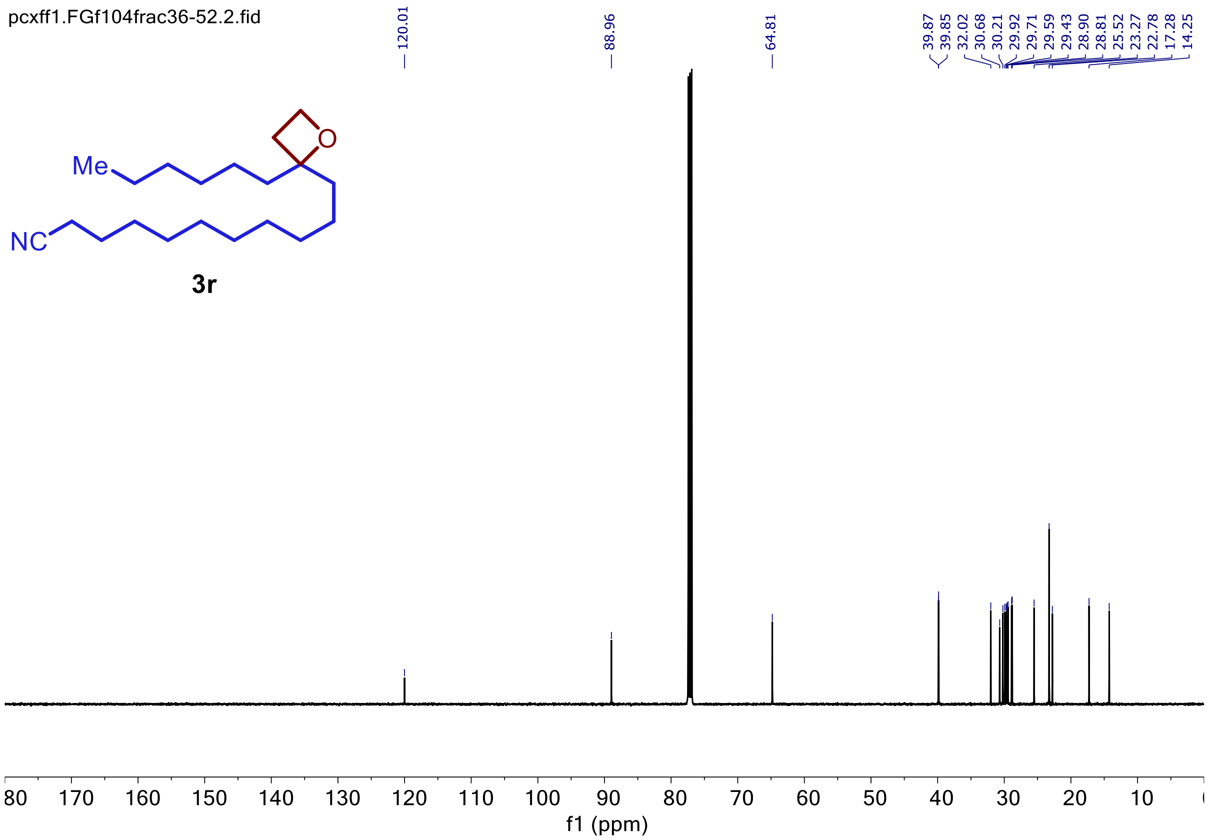

4-(oxetan-2-yl)tetrahydro-2*H*-pyran (3s)

^{1}H-NMR ($CDCl_3$, 400 MHz)

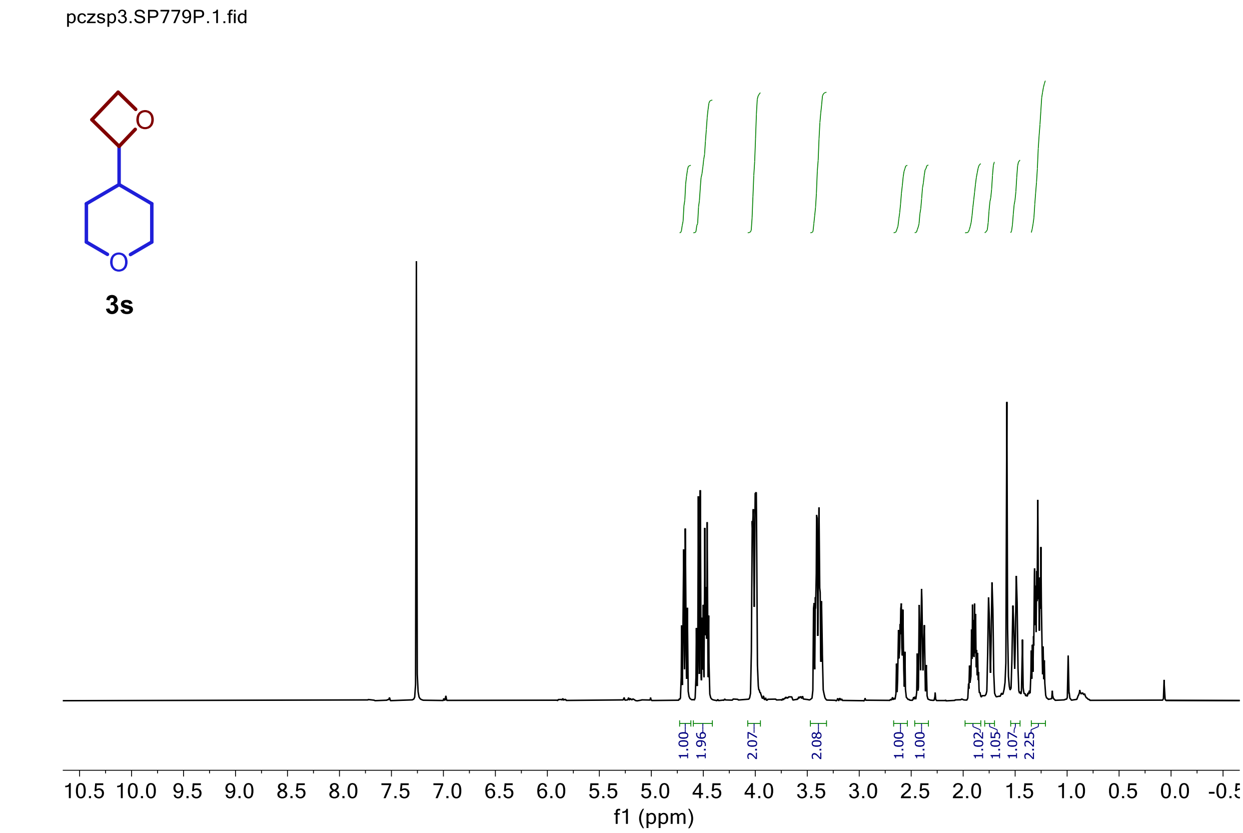

^{13}C-NMR ($CDCl_3$, 126 MHz)

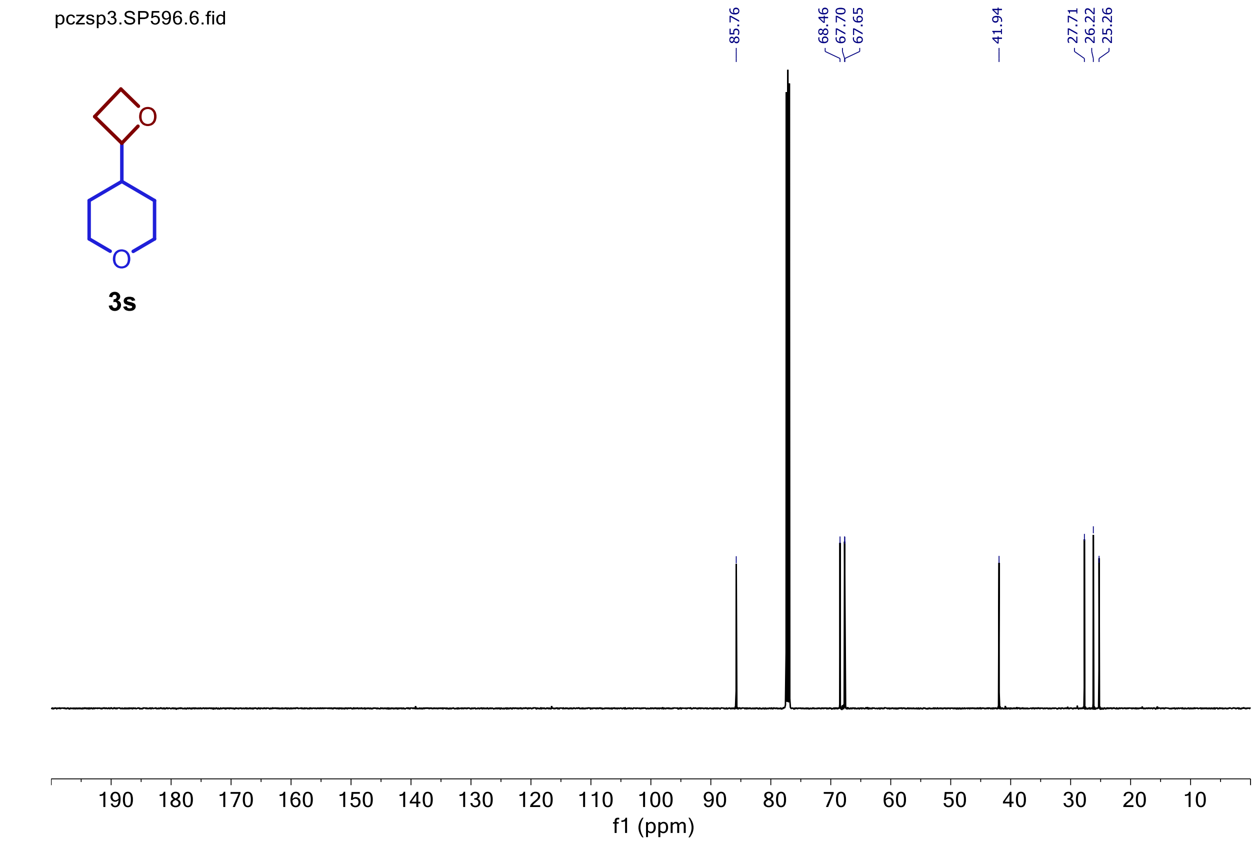

2-undecyloxetane (3t)

^{1}H-NMR ($CDCl_3$, 500 MHz)

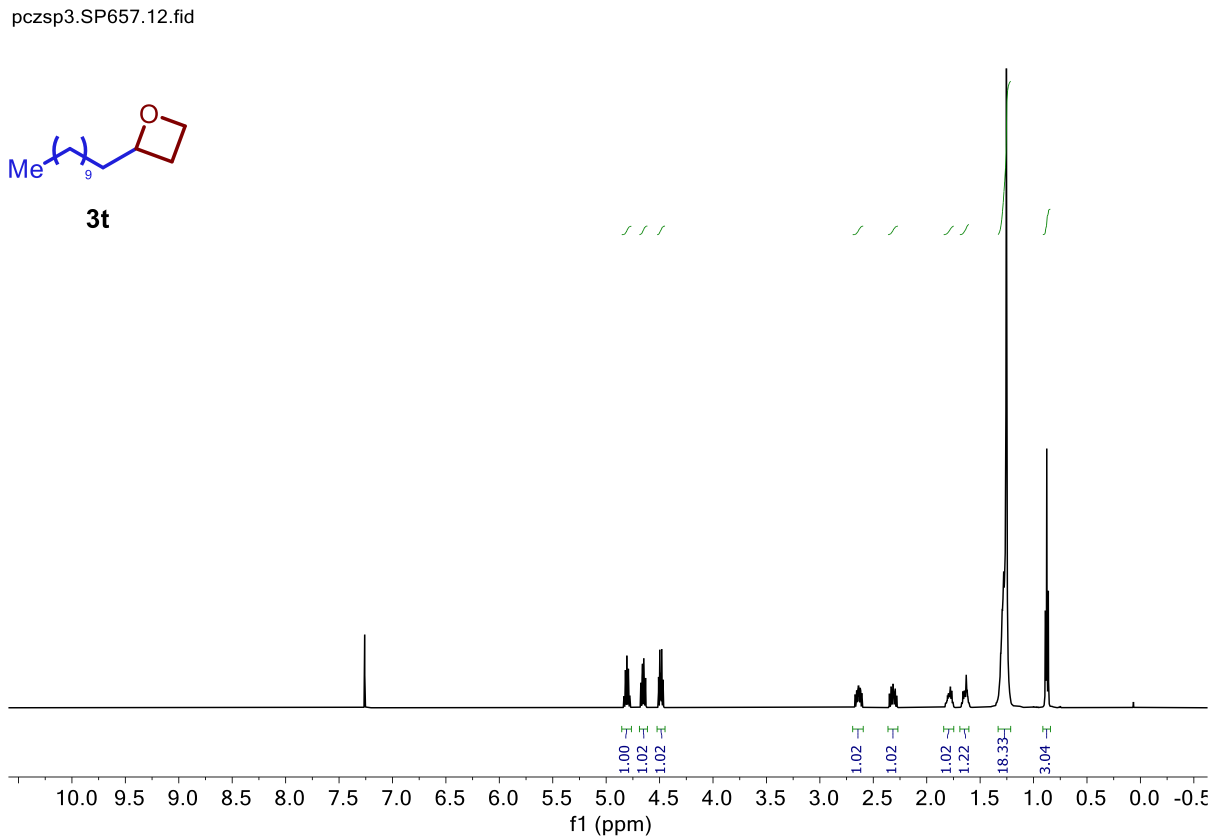

^{13}C-NMR ($CDCl_3$, 126 MHz)

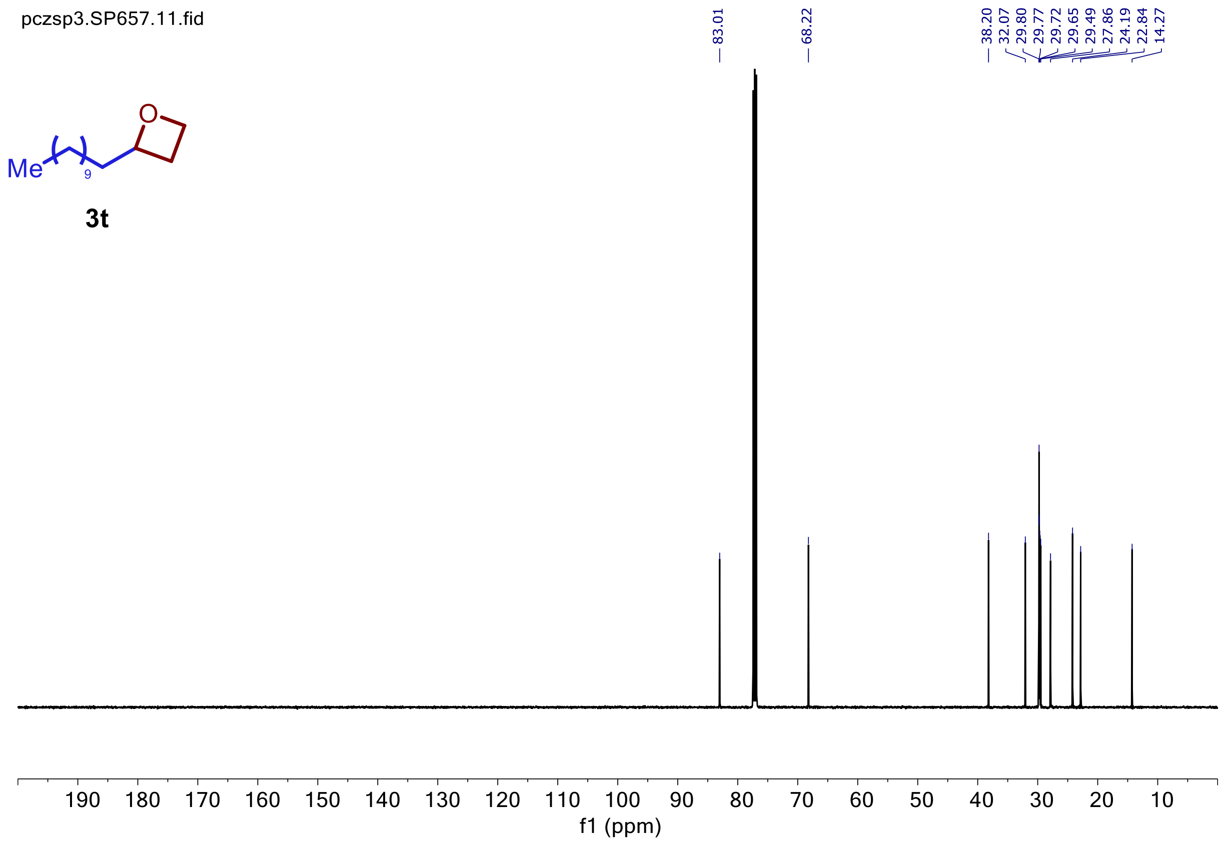

2-(3-phenylpropyl)oxetane (3u)

^{1}H-NMR ($CDCl_3$, 500 MHz)

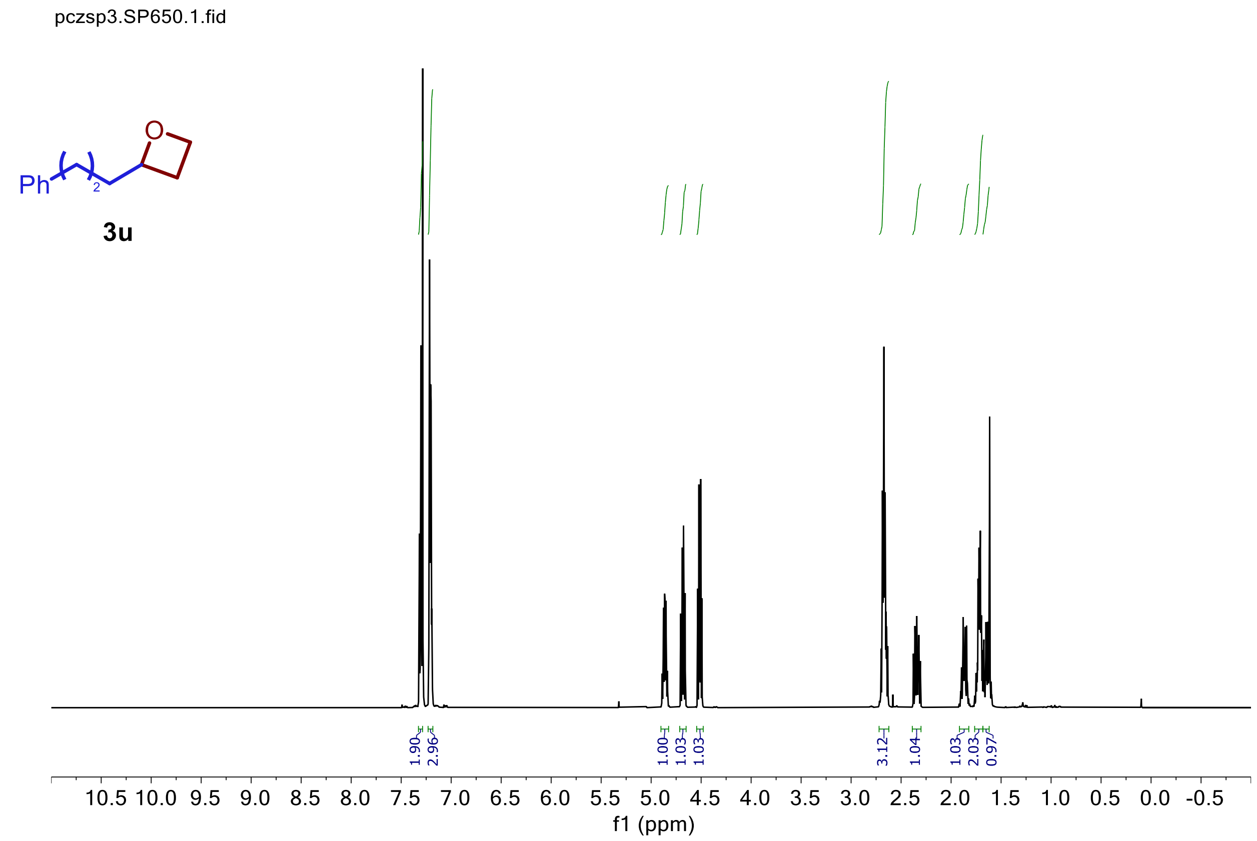

^{13}C-NMR ($CDCl_3$, 126 MHz)

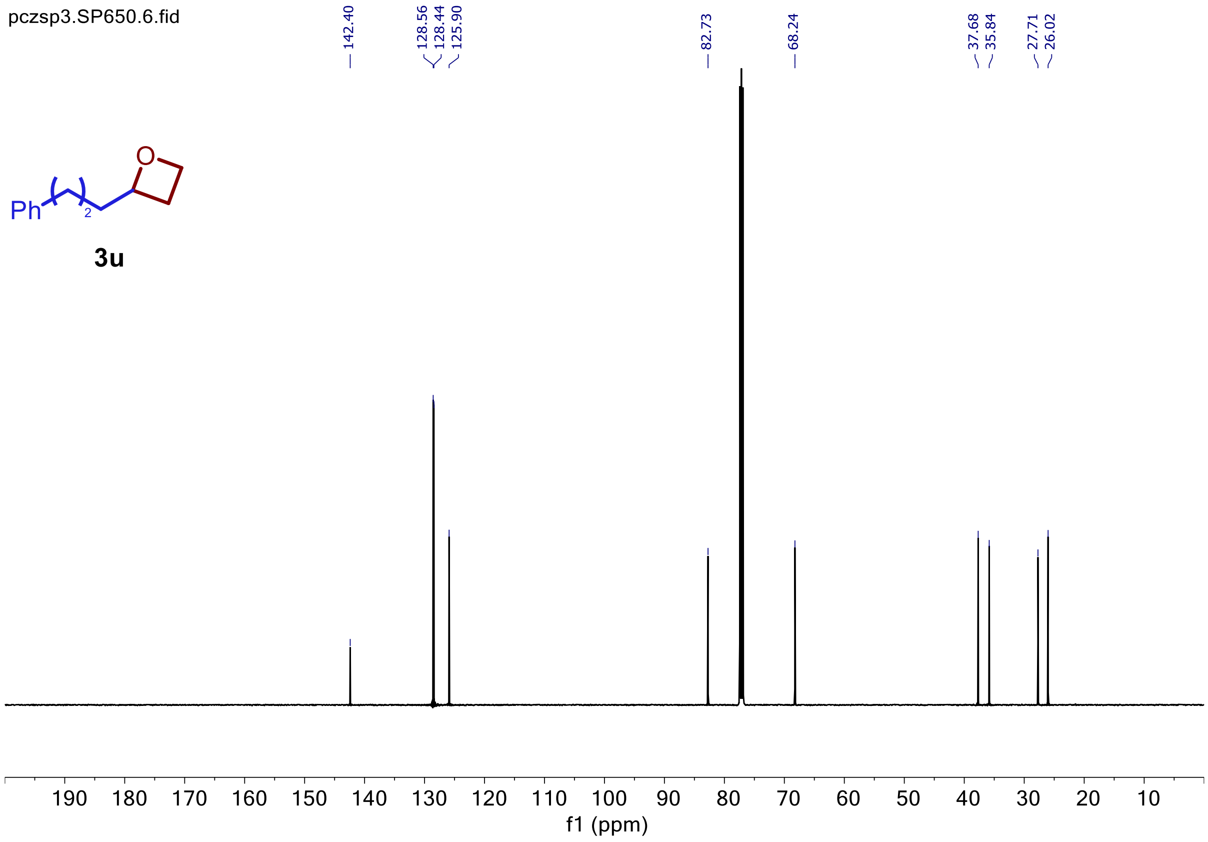

tert-butyl((5-(oxetan-2-yl)pentyl)oxy)diphenylsilane (3v)

^{1}H-NMR ($CDCl_3$, 500 MHz)

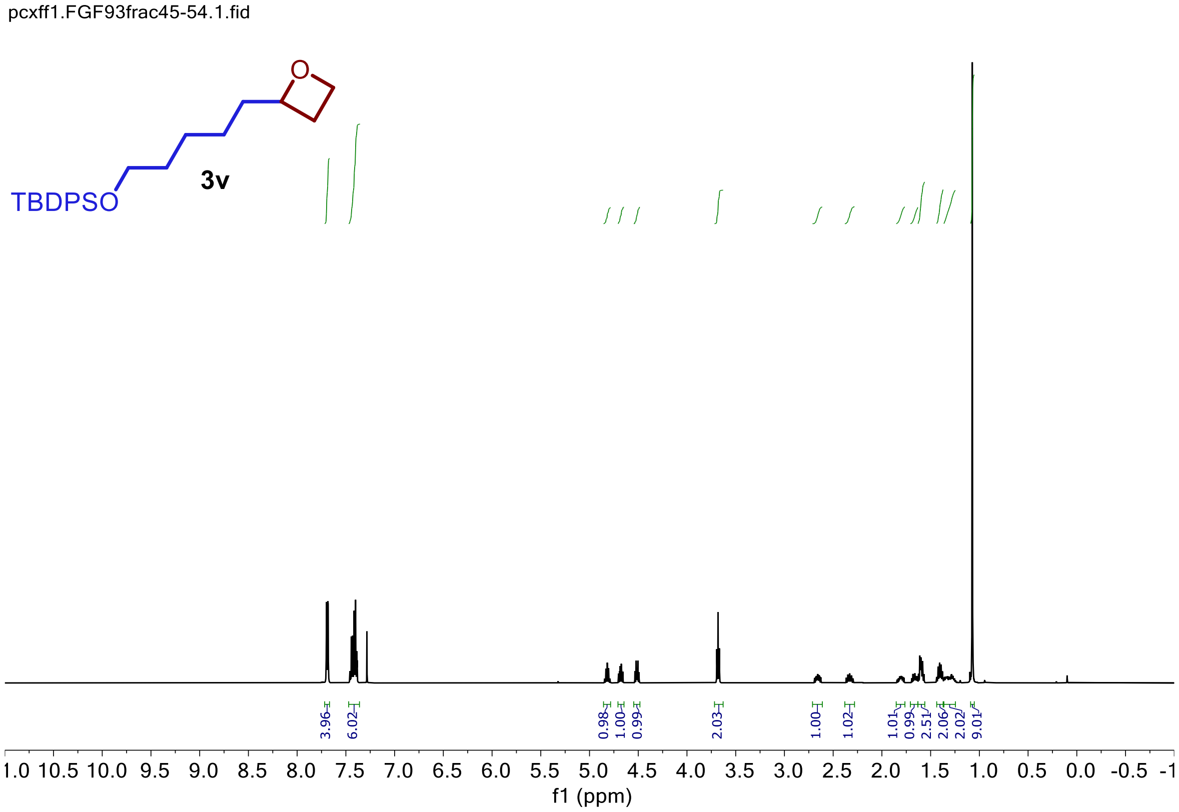

^{13}C-NMR ($CDCl_3$, 126 MHz)

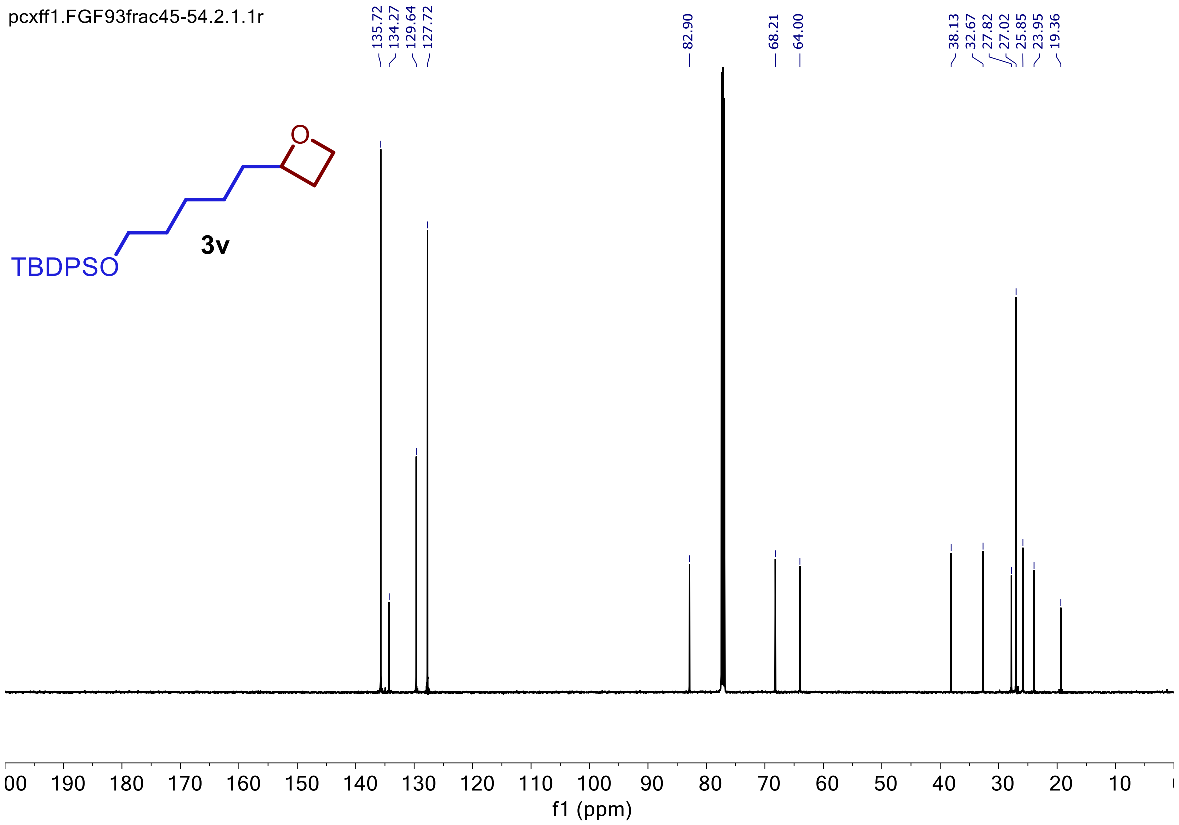

3-methyl-1-oxaspiro[3.5]nonane (3w)

^{1}H-NMR ($CDCl_3$, 400 MHz)

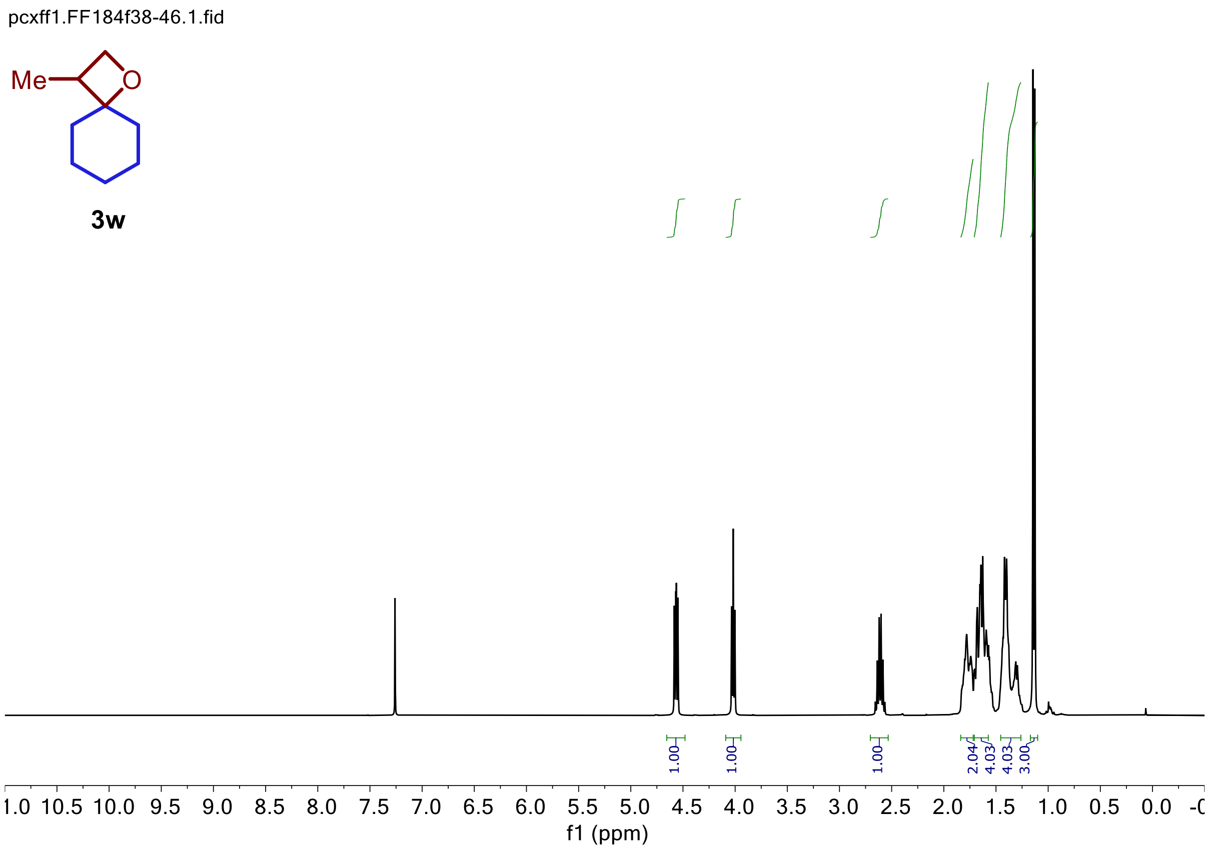

^{13}C-NMR ($CDCl_3$, 101 MHz)

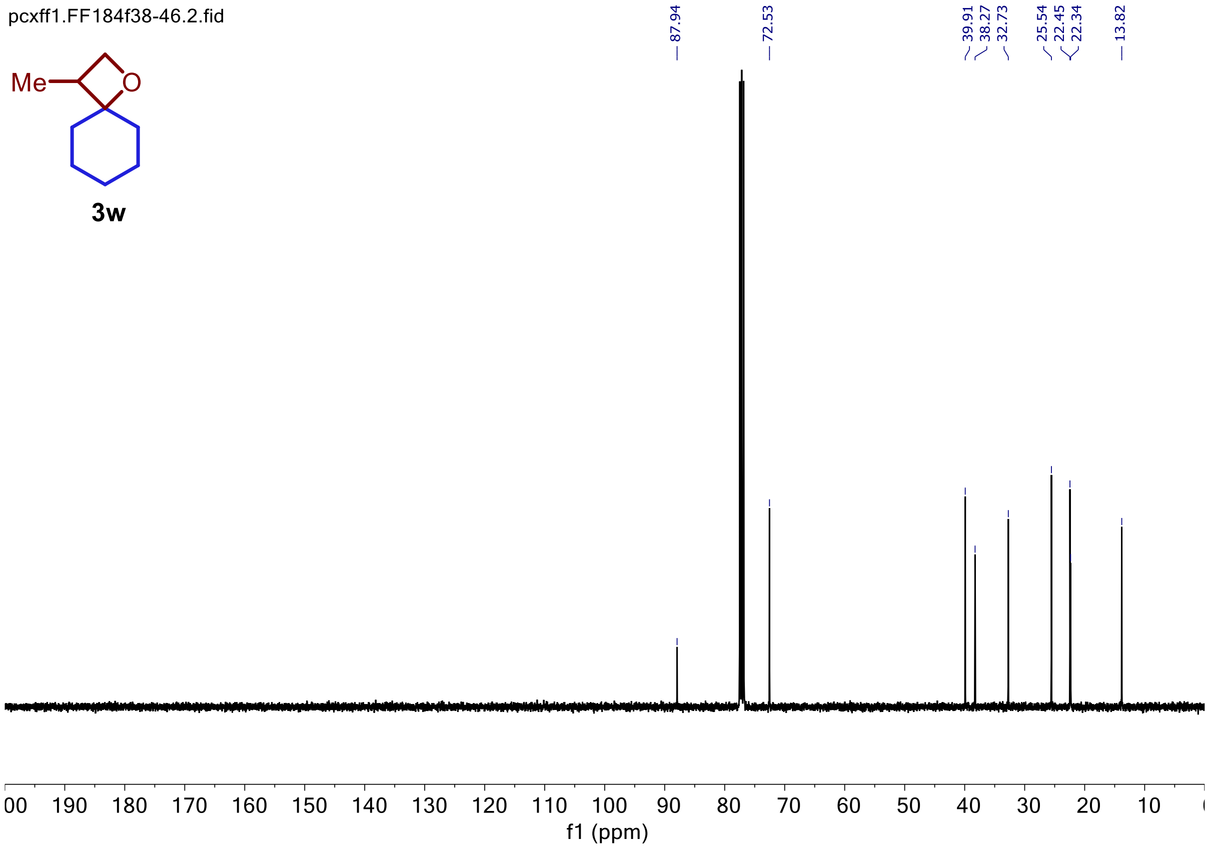

2-methyl-1-oxaspiro[3.5]nonane (3x)

^{1}H-NMR ($CDCl_3$, 500 MHz)

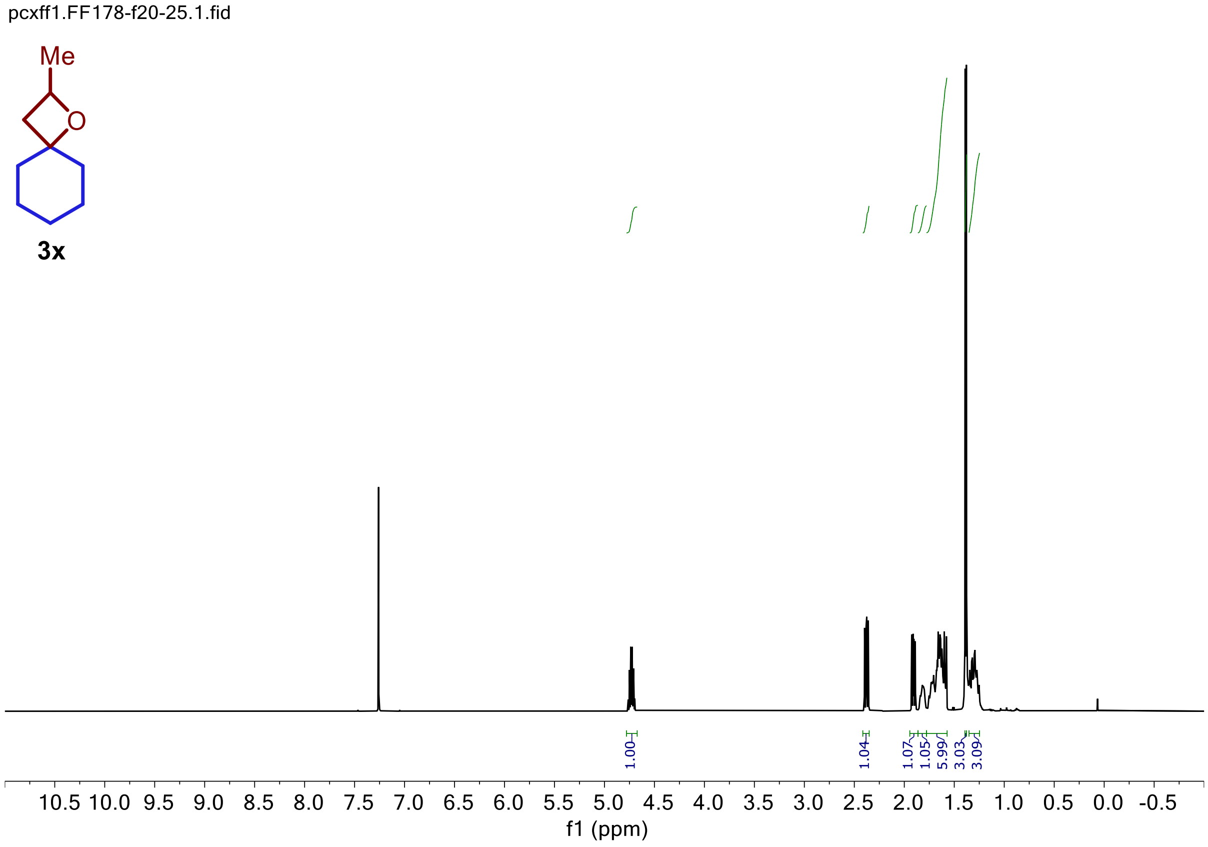

^{13}C-NMR ($CDCl_3$, 126 MHz)

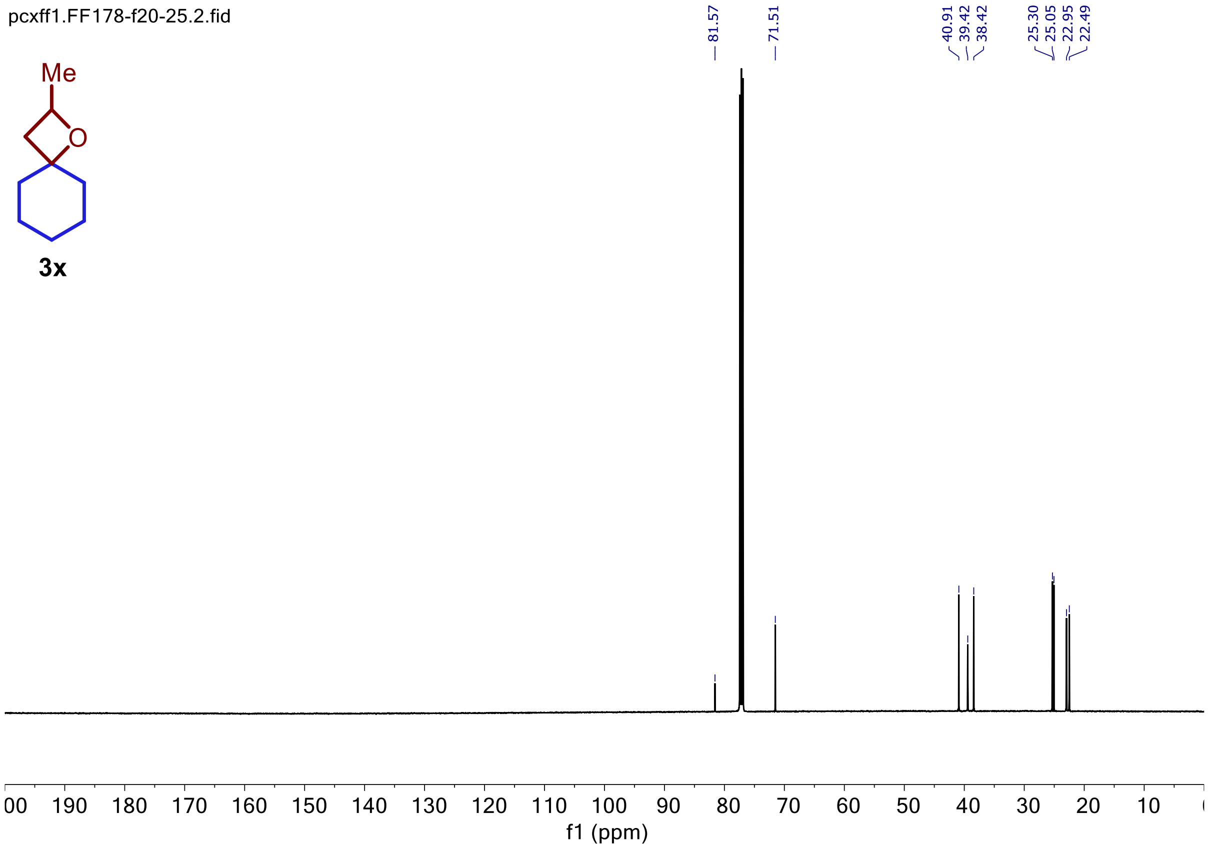

2-phenyl-1-oxaspiro[3.5]nonane (3y)

^{1}H-NMR ($CDCl_3$, 500 MHz)

pczsp3.DF1246A.1.fid

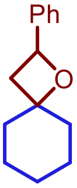

3y

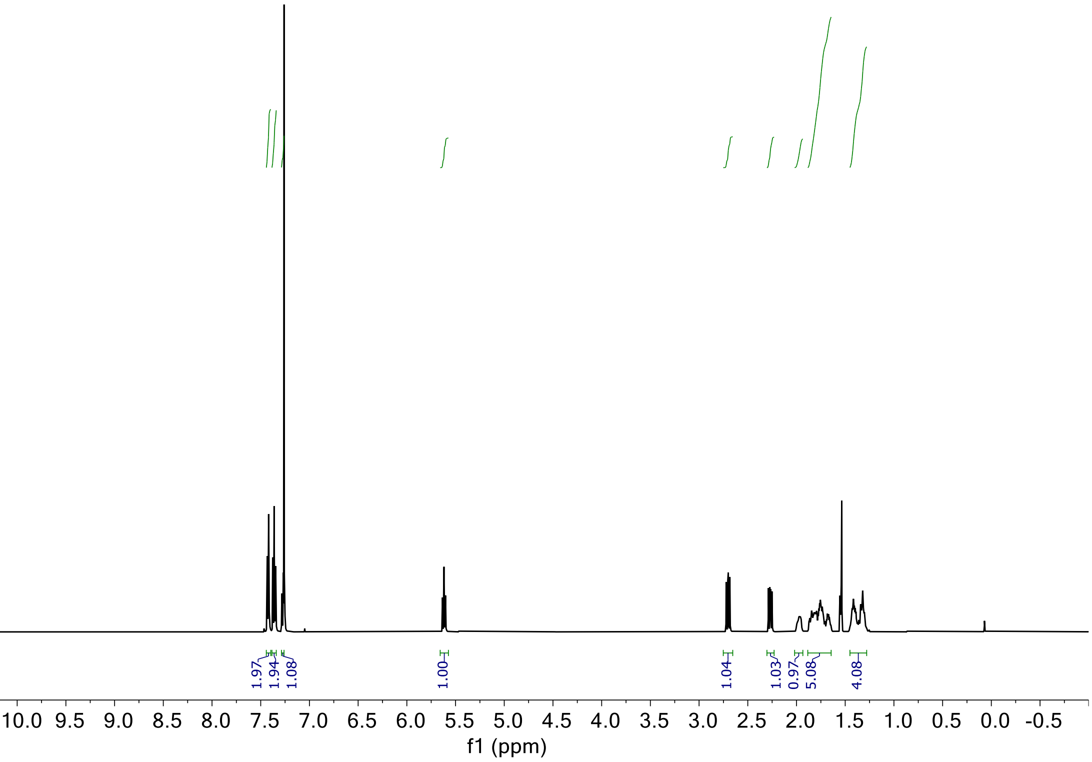

^{13}C-NMR ($CDCl_3$, 126 MHz)

pczsp3.DF1246A2.2.fid

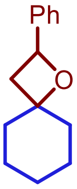

3y

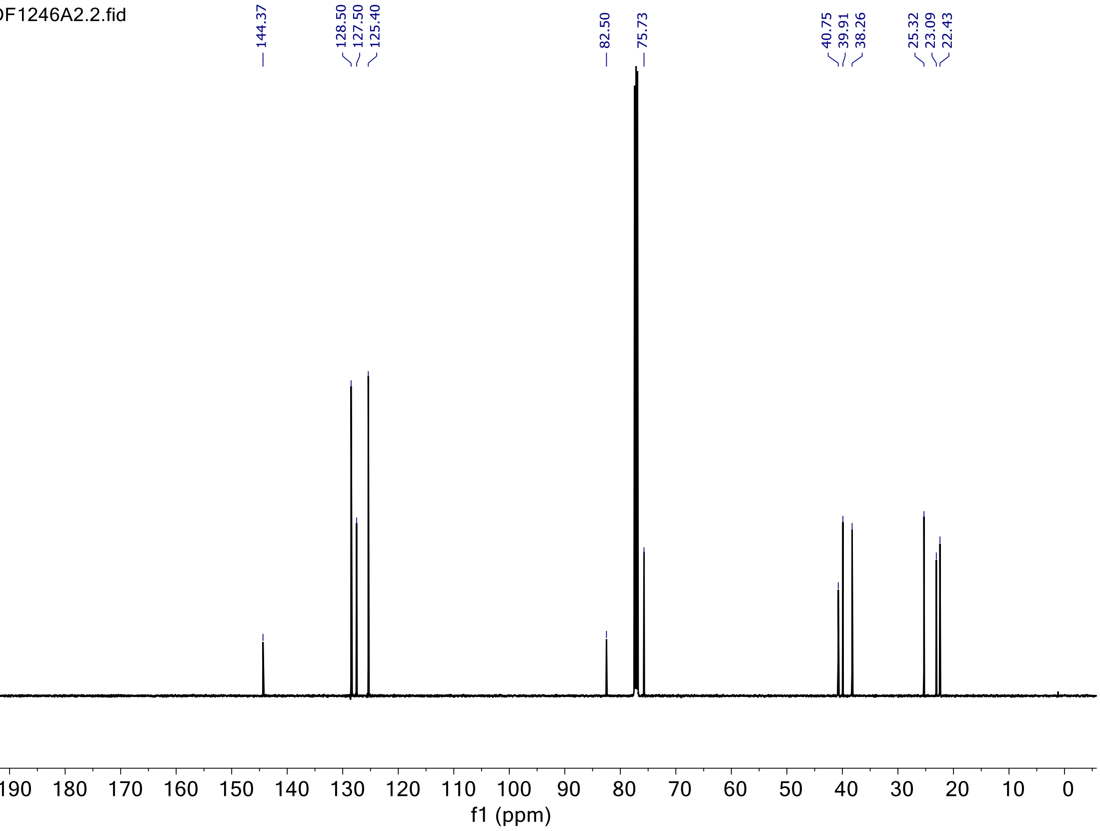

1-((3*S*,8*S*,9*S*,10*R*,13*S*,14*S*,17*S*)-10,13-dimethyl-1,2,4,7,8,9,10,11,12,13,14,15,16,17-tetradecahydrospiro[cyclopenta[*a*]phenanthrene-3,2'-oxetan]-17-yl)ethan-1-one (3z)

^{1}H-NMR (CDCl$_3$, 500 MHz)

pczsp3.SP643.6.fid

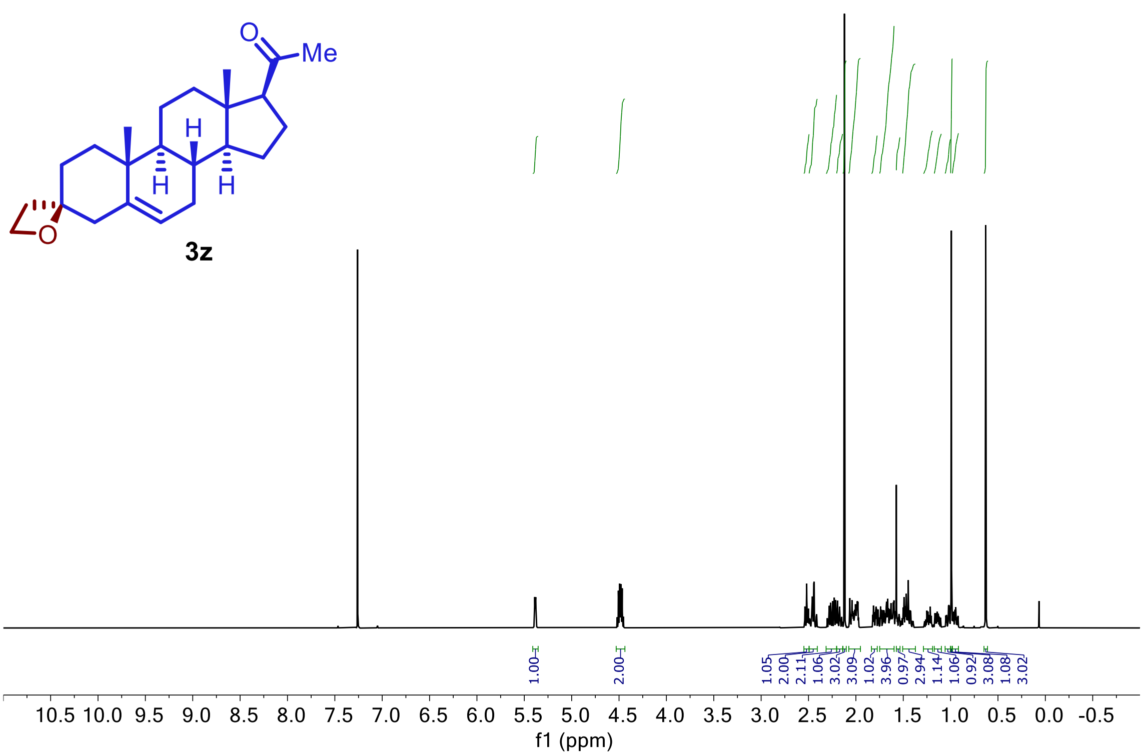

^{13}C-NMR (CDCl$_3$, 126 MHz)

pczsp3.SP643.11.fid

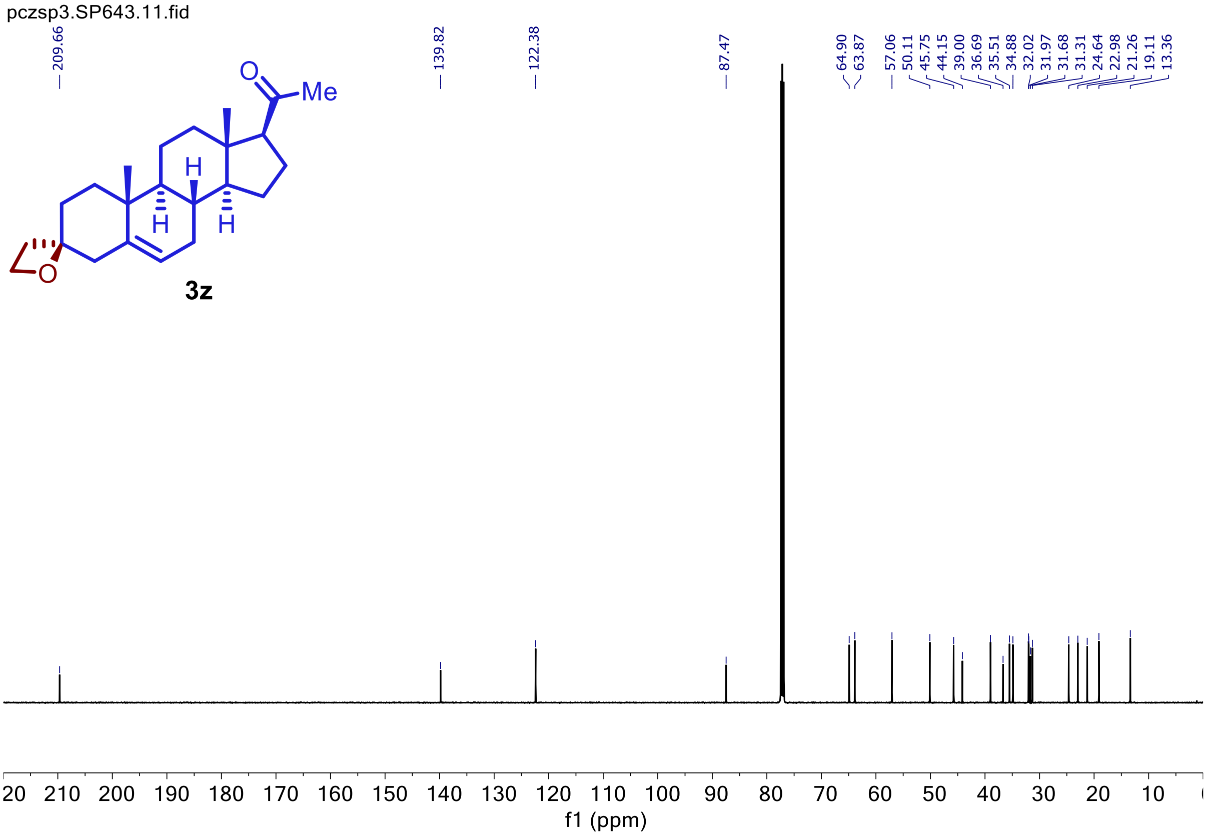

(3a*R*,5*R*,5a*S*,8a*S*,8b*R*)-2,2,7,7-tetramethyl-5-((*R*)-oxetan-2-yl)tetrahydro-5*H*-bis([1,3]dioxolo)[4,5-*b*:4',5'-d]pyran (3aa)

^{1}H-NMR ($CDCl_3$, 500 MHz)

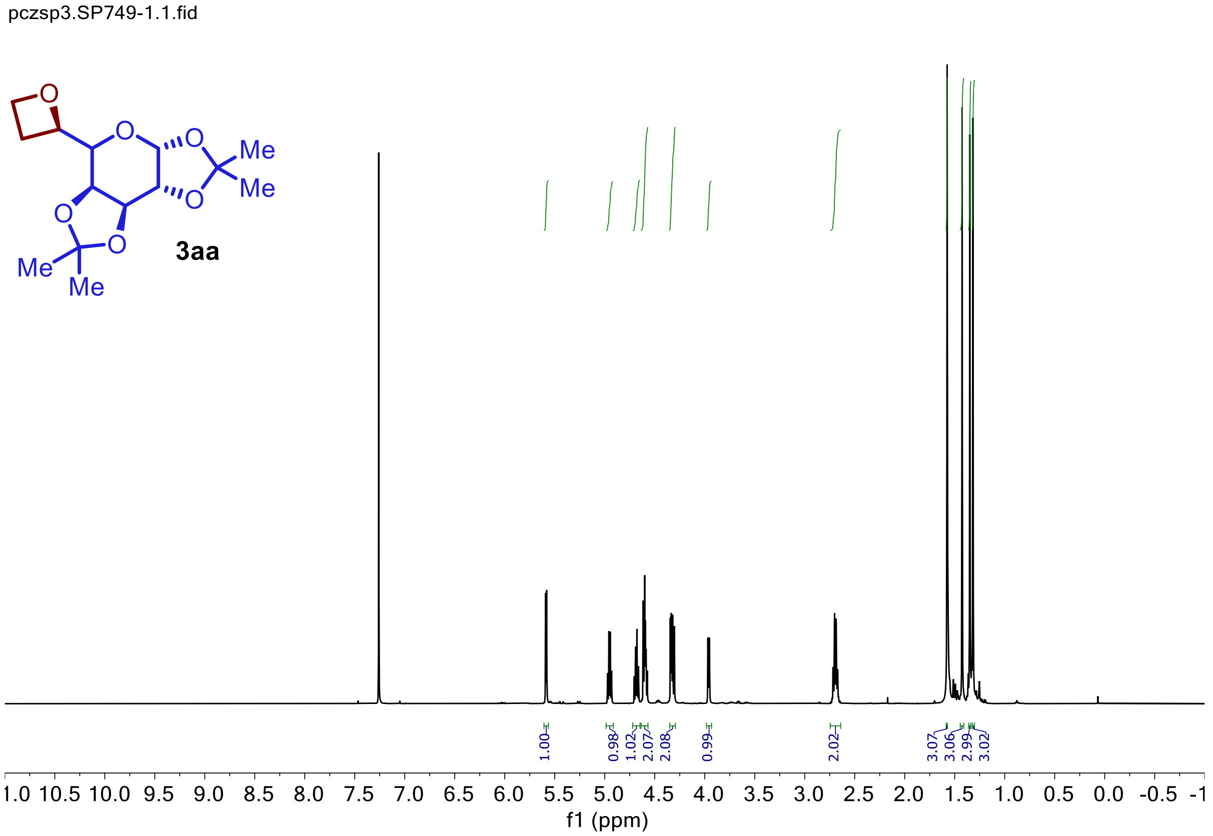

^{13}C-NMR ($CDCl_3$, 126 MHz)

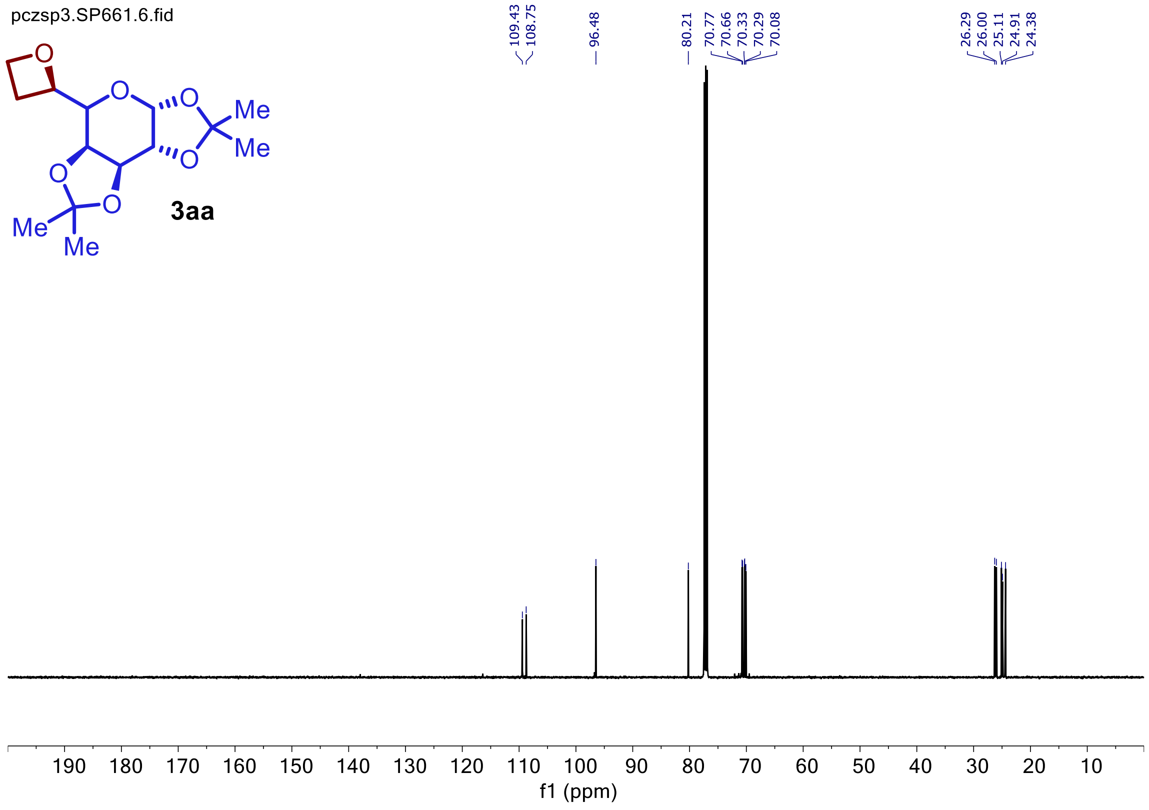

(8*R*,9*S*,10*R*,13*S*,14*S*,17*R*)-10,13-dimethyl-1,6,7,8,9,10,11,12,13,14,15,16-dodecahydrospiro[cyclopenta[*a*]phenanthrene-17,2'-oxetan]-3(2*H*)-one (3ab)

^{1}H-NMR ($CDCl_3$, 500 MHz)

pczsp3.SP651.20.fid

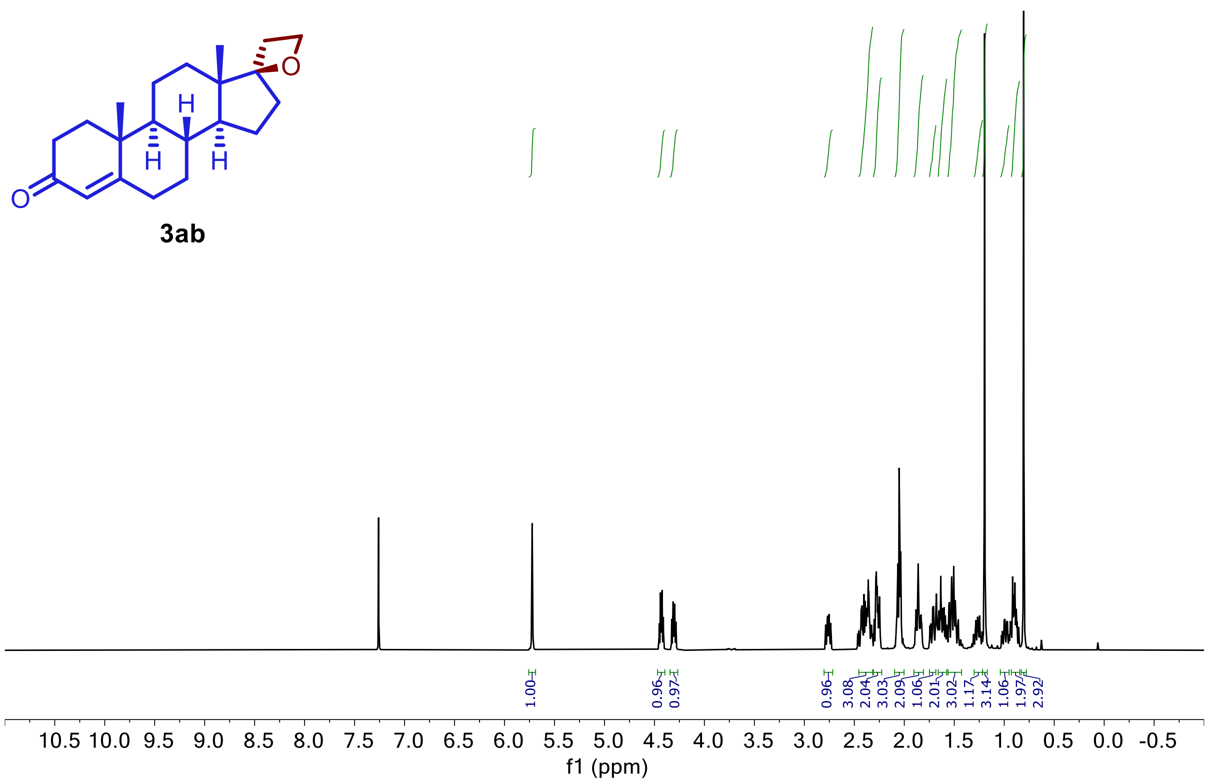

^{13}C-NMR ($CDCl_3$, 126 MHz)

pczsp3.SP651.13.fid

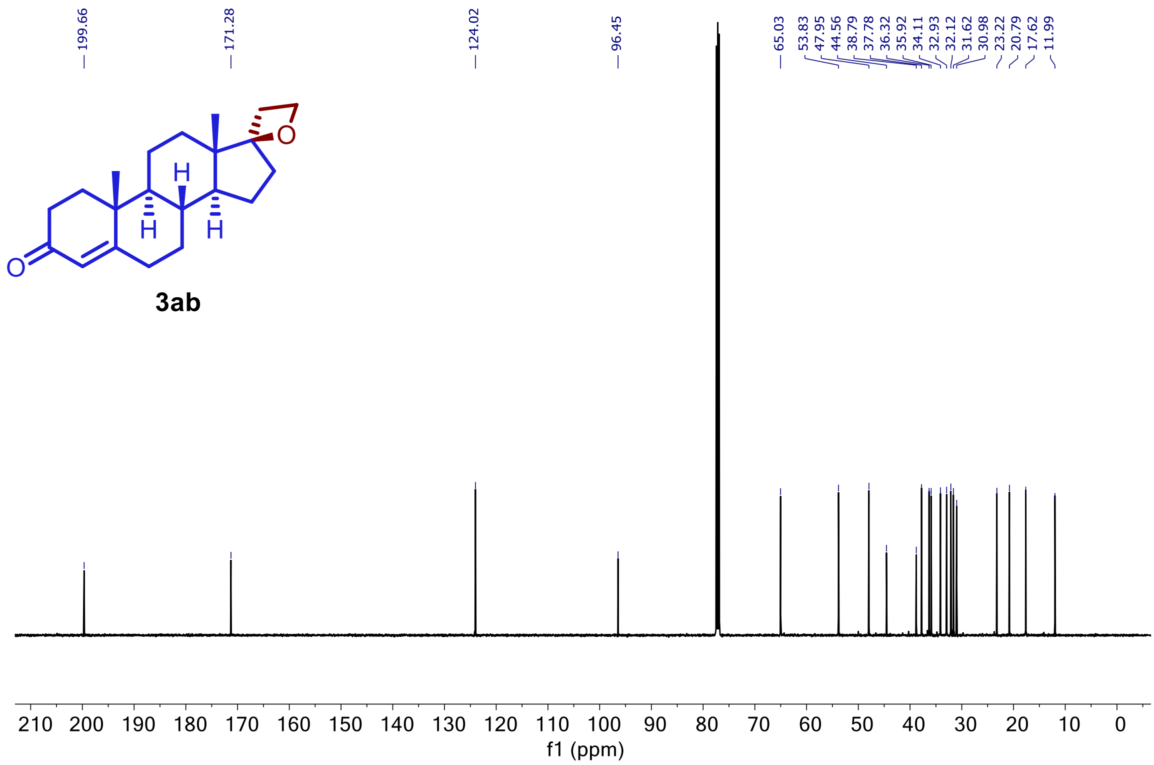

((*R*)-3-hydroxy-3-((3a*R*,5*R*,5a*S*,8a*S*,8b*R*)-2,2,7,7-tetramethyltetrahydro-5*H* bis([1,3]dioxolo)[4,5-*b*:4',5'-d]pyran-5-yl)propyl)dineopentylsulfonium triflate (S1)

^{1}H-NMR ($CDCl_3$, 500 MHz)

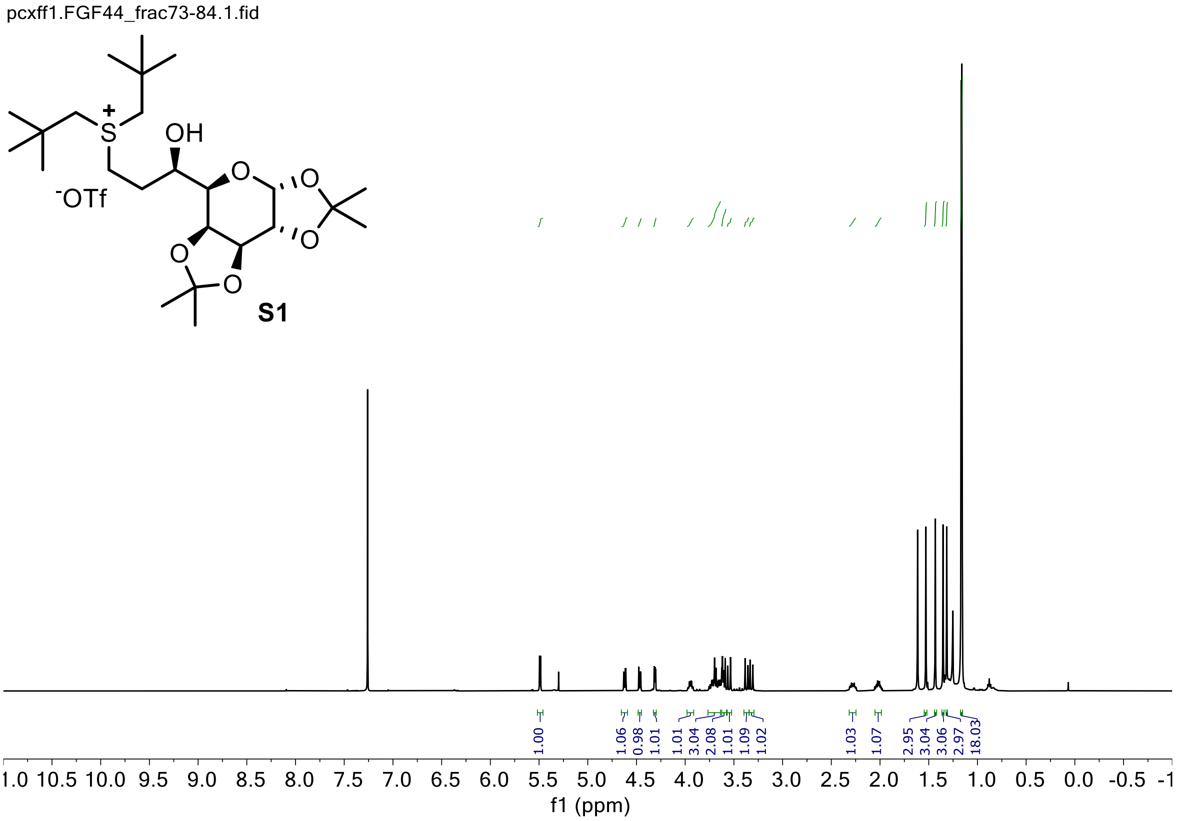

^{13}C-NMR ($CDCl_3$, 126 MHz)

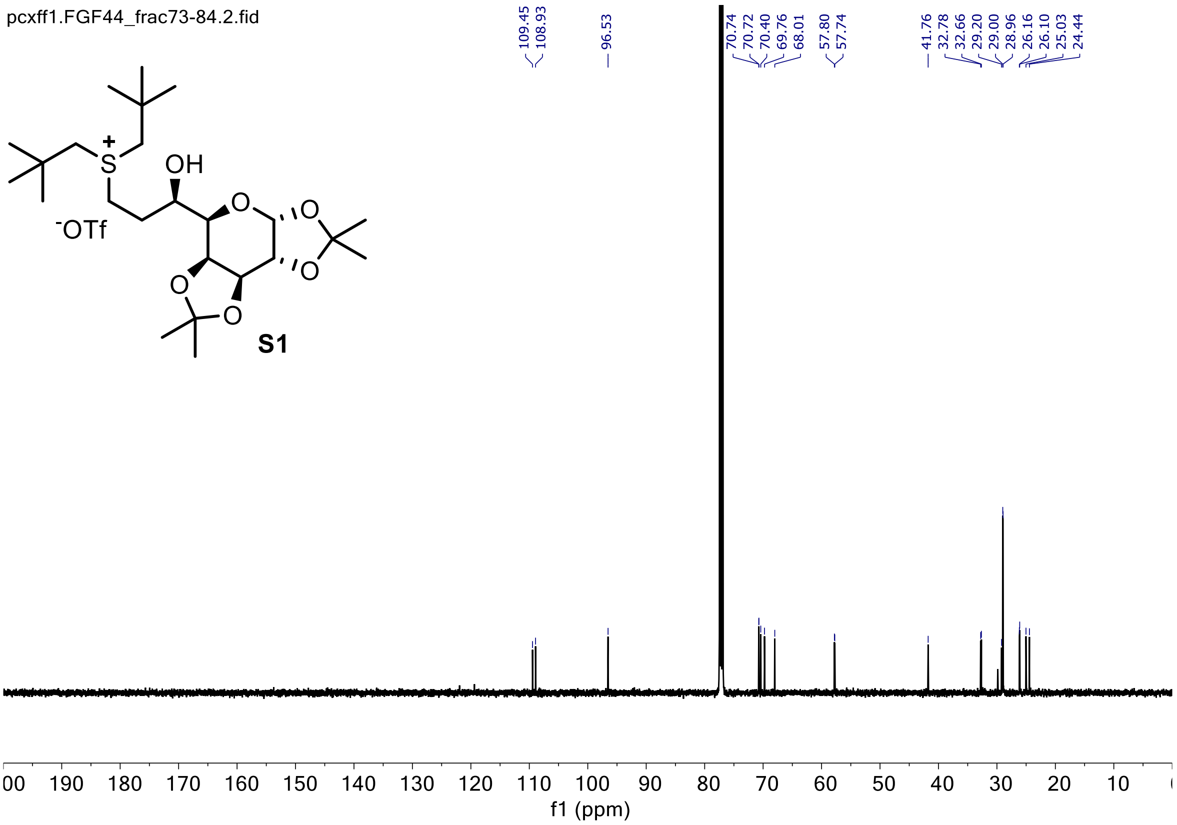

^{19}F-NMR ($CDCl_3$, 376 MHz)

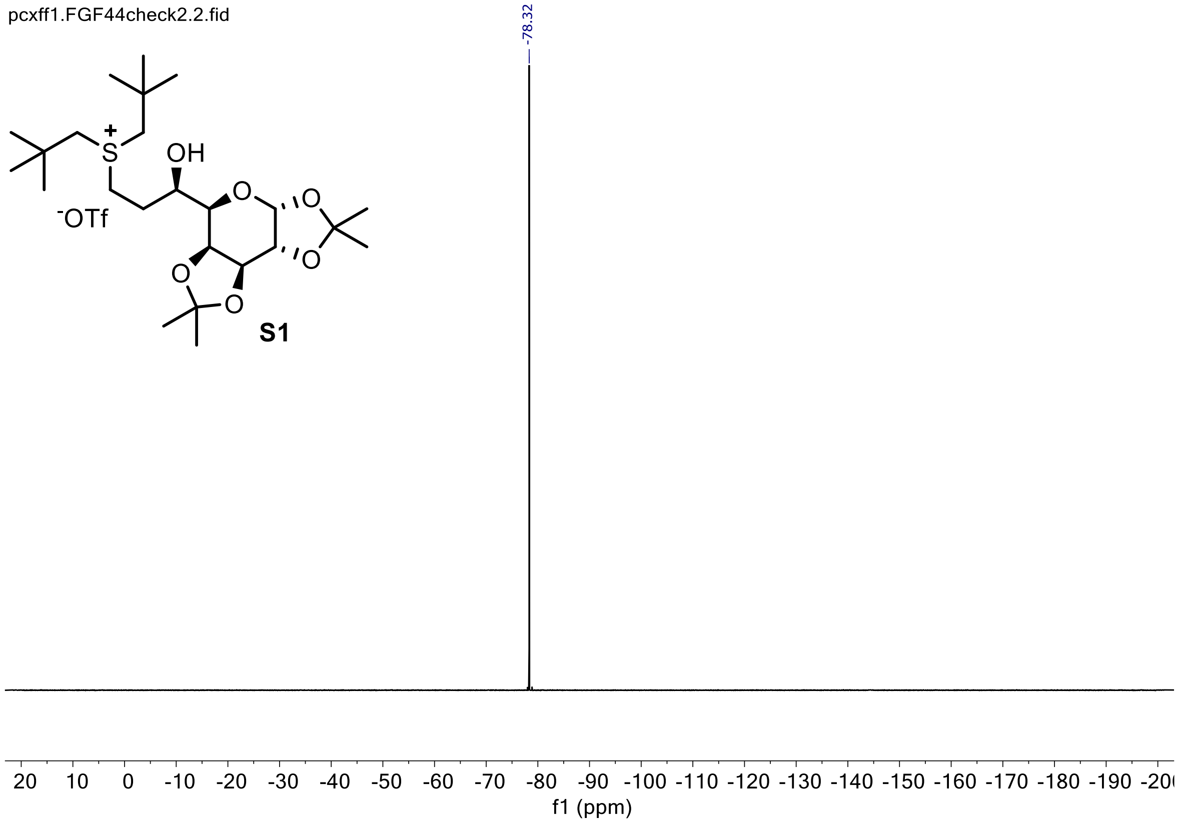

(*R*)-3-amino-1-((3a*R*,5*R*,5a*S*,8a*S*,8b*R*)-2,2,7,7-tetramethyltetrahydro-5*H*-bis([1,3]dioxolo)[4,5-*b*:4',5'-d]pyran-5-yl)propan-1-ol (S2)

^{1}H-NMR (CDCl$_3$, 500 MHz)

pcxff1.FGF117frac35-40.1.fid

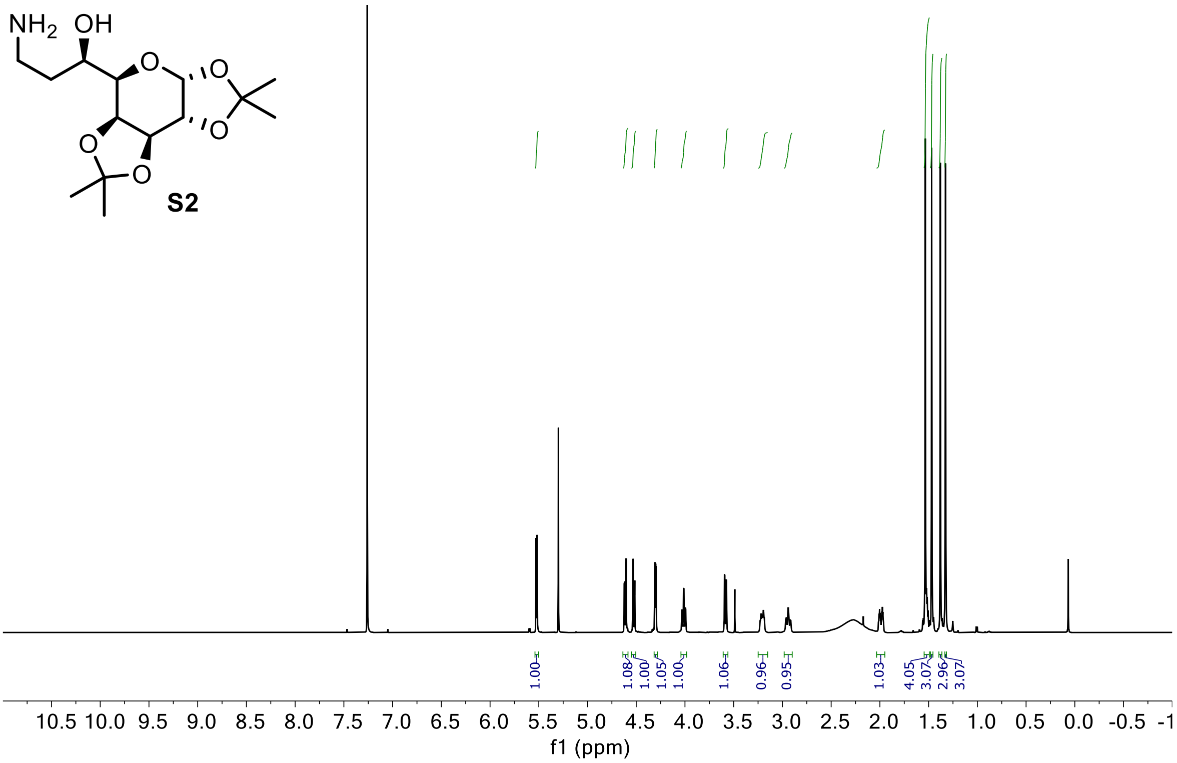

^{13}C-NMR (CDCl$_3$, 126 MHz)

pcxff1.FGF117frac35-40.2.fid

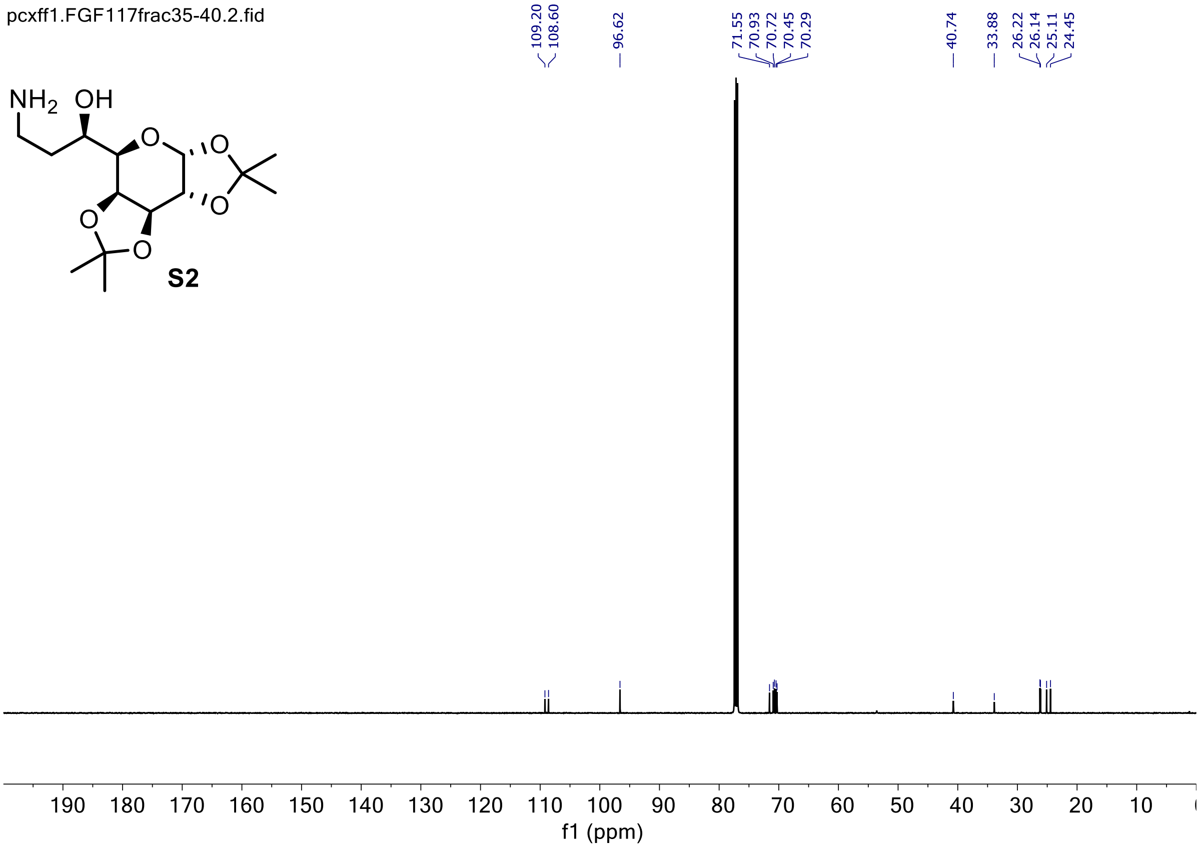

Supplement: Supplementary file 1 — ja3c04891_si_001.pdf [file ja3c04891_si_001.pdf]
